# Neural embedding of frailty in cognitively unimpaired aging and dementia across Latin America

**DOI:** 10.1002/alz.71232

**Published:** 2026-04-22

**Authors:** Joaquin Migeot, Olivia Wen, Raul Gonzalez‐Gomez, Hernan Hernandez, David Aguillon, Jose Alberto Avila‐Funes, Maria I. Behrens, Martin A. Bruno, Nilton Custodio, Carolina Delgado, Claudia Duran‐Aniotz, Dafne Duron‐Reyes, Adolfo M. García, Maria E. Godoy, Kun Hu, Brian Lawlor, Peng Li, Marcelo Adrian Maito, Diana L. Matallana, Bruce Miller, Maira Okada de Oliveira, Stefanie D. Pina‐Escudero, Katherine L. Possin, Elisa de Paula França Resende, Pablo Reyes, Hernando Santamaria‐Garcia, Andrea Slachevsky, Leonel T. Takada, Paulina Vergara, Kristine Yaffe, Jennifer S. Yokoyama, Roman Romero‐Ortuño, Agustin Ibanez

**Affiliations:** ^1^ Latin American Brain Health Institute (BrainLat) Universidad Adolfo Ibañez Santiago de Chile, Metropolitan Region of Santiago Chile; ^2^ Global Brain Health Institute (GBHI) Trinity College Dublin Dublin Ireland; ^3^ Grupo de Neurociencias de Antioquia Facultad de Medicina Universidad de Antioquia Medellín Colombia; ^4^ Dirección de Enseñanza Instituto Nacional de Ciencias Médicas y Nutrición Salvador Zubirán Mexico City Mexico; ^5^ Univ. Bordeaux Inserm, Bordeaux Population Health Research Center Bordeaux France; ^6^ Centro de Investigación Clínica Avanzada (CICA) – Hospital Clínico Universidad de Chile, Santiago Chile Independencia Santiago Chile; ^7^ Departamento de Neurología y Neurocirugía Hospital Clínico Universidad de Chile Independencia Santiago Chile; ^8^ Departamento de Neurociencia Facultad de Medicina Universidad de Chile Independencia Santiago Chile; ^9^ Departamento de Neurología y Psiquiatría Clínica Alemana de Santiago‐Universidad del Desarrollo Santiago Chile; ^10^ Consejo Nacional de Investigaciones Científicas y Técnicas (CONICET) Buenos Aires Argentina; ^11^ Instituto de Ciencias Biomédicas (ICBM) Facultad de Ciencias Médicas Universidad Católica de Cuyo San Juan Argentina; ^12^ Unidad de diagnóstico de deterioro cognitivo y prevención de demencia, Instituto Peruano de Neurociencias Lima Perú; ^13^ Unidad de investigación, Equilibria Lima Perú; ^14^ Departamento de Neurología y Neurocirugía. Hospital Clínico Universidad de Chile Santiago Chile; ^15^ Departamento de Neurociencia Facultad de medicina Universidad de Chile Santiago Chile; ^16^ Alzheimer Aguascalientes Family Foundation Aguascalientes Mexico; ^17^ Departamento de Lingüística y Literatura Facultad de Humanidades Universidad de Santiago de Chile Santiago Chile; ^18^ Global Brain Health Institute University of California San Francisco California USA; ^19^ Cognitive Neuroscience Center Universidad de San Andrés Buenos Aires Argentina; ^20^ Department of Anesthesia Critical Care and Pain Medicine Massachusetts General Hospital Harvard Medical School Boston Massachusetts USA; ^21^ Division of Sleep Medicine Harvard Medical School Boston Massachusetts USA; ^22^ The Broad Institute of MIT and Harvard Cambridge Massachusetts USA; ^23^ Departamento de Salud Mental Hospital Universitario Fundación Santa Fe Bogotá Colombia; ^24^ Centro de Memoria y Cognición Hospital Universitario San Ignacio Bogotá Colombia; ^25^ Department of Neurology Memory and Aging Center University of California San Francisco California USA; ^26^ Cognitive Neurology and Behavioral Unit (GNCC) University of São Paulo São Paulo Brazil; ^27^ Universidade Federal de Minas Gerais Belo Horizonte Minas Gerais Brazil; ^28^ Pontificia Universidad Javeriana Bogotá, D.C. Colombia; ^29^ Hospital Universitario San Ignacio Center for Memory and Cognition, Intellectus Bogotá, D.C Colombia; ^30^ Geroscience Center for Brain Health and Metabolism (GERO) Santiago de Chile, Metropolitan Region of Santiago Chile; ^31^ Memory and Neuropsychiatric Center (CMYN) Neurology Department Hospital del Salvador & Faculty of Medicine University of Chile Santiago de Chile Chile; ^32^ Neuropsychology and Clinical Neuroscience Laboratory (LANNEC) Physiopathology Program – Institute of Biomedical Sciences Neuroscience and East Neuroscience Departments Faculty of Medicine University of Chile Santiago de Chile Chile; ^33^ Departamento de Medicina Servicio de Neurología Clínica Alemana‐Universidad del Desarrollo Santiago de Chile, Metropolitan Region of Santiago Chile; ^34^ Department of Psychiatry Weill Institute for Neurosciences University of California San Francisco California USA; ^35^ Department of Epidemiology & Biostatistics University of California San Francisco California USA; ^36^ Discipline of Medical Gerontology School of Medicine Trinity College Dublin Ireland; ^37^ Department of Biophysics School of Medicine Istanbul Medipol University Istanbul Türkiye; ^38^ Barcelonaβeta Brain Research Center (BBRC) Pasqual Maragall Foundation Barcelona Spain

**Keywords:** Alzheimer's disease, brain frailty, cognitively unimpaired subjects, frailty, frailty network, frontotemporal lobar degeneration, functional connectivity, gray matter volume, Latin America, machine learning, neuroimaging, voxel‐based morphometry

## Abstract

**INTRODUCTION:**

Frailty influences dementia risk and severity. However, its role in differentiating dementia subtypes and associations with brain structural and functional alterations remain understudied, especially in Latin America.

**METHODS:**

Multi‐Partner Consortium to Expand Dementia Research in Latin America data included 3461 participants (cognitively unimpaired [CU], Alzheimer's disease [AD], and frontotemporal lobar degeneration [FTLD]) from Latin America using a frailty index constructed from 32 health‐related variables. XGBoost‐logistic regression models tested group discrimination, and voxel‐based morphometry plus functional connectivity analyses explored neural correlates.

**RESULTS:**

Frailty distinguished CU from AD (area under the curve [AUC] = 0.85) and CU from FTLD (AUC = 0.88) but not AD from FTLD (AUC = 0.59). Higher frailty was linked to widespread gray matter loss, with temporal involvement in CU and stronger frontotemporal effects in dementia, particularly FTLD. Connectivity analyses showed fronto‐temporo‐posterior reductions and increased connectivity across the frailty network.

**DISCUSSION:**

Findings position frailty as a promising marker for identifying AD and FTLD relative to CU individuals, linked with brain health alterations in Latin American populations.

## Highlights

- Frailty index distinguished CU from AD (AUC = 0.85) and FTLD (AUC = 0.88).
- Frailty failed to distinguish AD from FTLD (AUC = 0.59).
- Higher frailty was linked to widespread gray matter loss in CU and dementia.
- FTLD showed stronger frontotemporal gray matter effects than AD.
- Frailty is related to reduced fronto‐temporo‐posterior and increased network connectivity.

## 1 BACKGROUND

Frailty is characterized by decreased physiological reserve and heightened vulnerability to stressors, stemming from the cumulative burden of health deficits across multiple biopsychosocial domains.^1^, ^2^ Like other measures of accelerated aging, frailty is a marker of unhealthy aging. People living with frailty have a higher risk of dementia, and this is thought to be mediated by multiple molecular, cellular, and physiological deficits, expressed as increased comorbidities, disabilities, and acceleration of biological aging.^3^, ^4^, ^5^, ^6^, ^7^ Frailty increases the risk of conversion from mild cognitive impairment to dementia^8^ and may moderate the relationship between neuropathology and dementia in Alzheimer's disease (AD),^5^ suggesting a pathology‐accelerating or reduced resilience effect. Neuropathological studies have linked frailty to reduced gray matter volumes (GMVs) in dementia‐sensitive regions such as the frontal and parietal lobes, hippocampus, insula, and cerebellum, alongside functional connectivity disruptions spanning motor, sensorimotor, cingulate, and precuneus hubs.^9^ The global prevalence of frailty is 13.6%,^10^ reaching 21.7% in Latin America, ranging from 10.6% in Colombia to 31.3% in Chile.^11^ The number of older adults with frailty is expected to rise in this region, linked with a rapid increase in the aging population and a higher burden of chronic disease and disabilities compared to developed countries.^12^ Regionally grounded research is needed to characterize the links between frailty and its neural embedding in healthy aging and dementia in Latin America.

However, significant gaps in the evidence remain. Most available studies from Latin America narrowly focus on the physical aspects of frailty, often overlooking socioemotional, functional, cognitive, and broader health domains essential for a comprehensive assessment.^2^, ^13^, ^14^ Frailty research in the region also tends to examine cognitive decline^15^ or dementia diagnosis as a general umbrella,^16^, ^17^, ^18^, ^19^, ^20^ without addressing specific subtypes such as AD and frontotemporal lobar degeneration (FTLD) or conducting comparative analyses. The use of multimodal neuroimaging to characterize frailty‐related brain alterations in healthy aging and dementia remains understudied, limited to cerebrovascular measures, with inconsistent results.^21^, ^22^ These limitations preclude the development of comprehensive frailty assessments and multimodal neuroimaging approaches in Latin America, which would be useful for refining frailty phenotyping across the spectrum from cognitively unimpaired (CU) individuals to dementia stages and characterizing syndrome‐specific brain signatures in AD and FTLD.

We aimed to fill the aforementioned gaps by evaluating the role of frailty in dementia subtype characterizations and its association with structural and functional brain alterations among CU, AD, and FTLD from Latin American sites. We analyzed data from 3461 individuals from the Multi‐Partner Consortium to Expand Dementia Research in Latin America (ReDLat),^23^ constructing a deficit accumulation‐based frailty index,^1^, ^24^ including 32 health‐related variables encompassing cardiometabolic assessments, visual and auditory health, total number of diagnoses and medications, health behaviors, global clinical status, neuropsychiatric symptoms, depression, anxiety, functional ability, and cognition. This comprehensive approach enabled us to elucidate how frailty contributed to distinct neurodegenerative profiles and their underlying brain mechanisms.

**RESEARCH IN CONTEXT**

1. **Systematic review**: We reviewed prior studies on frailty and dementia through PubMed, Scopus, and regional Latin American databases. While frailty has been widely studied as a multidimensional risk factor for dementia, few investigations have examined its structural and functional brain correlates, and even fewer have done so across Latin American populations.
2. **Interpretation**: Our findings demonstrate that frailty robustly distinguishes dementia subtypes from CU individuals and is associated with shared and syndrome‐specific structural and functional brain alterations that map onto a brain frailty network. These results position frailty as an integrative marker of disease burden and clinical status in dementia, complementing traditional biomarker‐based approaches.
3. **Future directions**: Future research should clarify the mechanisms linking frailty to dementia pathophysiology, test frailty‐informed interventions, and expand analyses to diverse and underrepresented populations. Specific priorities include exploring frailty as a moderator of neuropathological processes in frontotemporal dementia variants and assessing its utility in clinical trials for prevention and care strategies.

## 2 METHODS

### 2.1 Participants

A total of 3461 participants were included in this study, with a mean age of 63.48 years (SD = 12.85), 65.78% of whom were female (Table 1). The dataset comprised three groups: CU individuals (*n* = 1924, mean age 58.50, SD = 13.58; 69.50% female), 1126 individuals with probable AD (mean age 70.91, SD = 8.24; 65.1% female), and 411 participants with probable FTLD (mean age 66.31, SD = 8.23; 50.10% female). All participants were recruited through ReDLat^23^, ^25^ across  countries: Argentina (*n* = 197), Brazil (*n* = 323), Chile (*n* = 340),[Table alz71232-tbl-0001] Colombia (*n* = 1418), Mexico (*n* = 418), Peru (*n* = 765). ReDLat gathered data from multiple locations across each country, employing a standardized data framework and consistent diagnostic protocols.^26^, ^27^, ^28^, ^29^, ^30^ Participants were enrolled through a multifaceted recruitment strategy encompassing (a) clinical settings such as memory clinics, neurology services, and partner hospitals; (b) academic networks established through collaborations with universities and research centers; (c) community‐based initiatives that involved outreach events and culturally sensitive materials aimed at engaging both urban and rural populations from varied socioeconomic contexts; and (d) alliances with public health programs and grassroots organizations to align participant recruitment with broader awareness and health promotion campaigns. These approaches enabled the inclusion of individuals across diverse settings, with a deliberate emphasis on populations historically underrepresented due to socioeconomic and educational disparities, as reflected in the ReDLat cohort to enhance participation and accessibility, recruitment methods included on‐site assessments, community‐centered outreach, and deployment of mobile units.

**TABLE 1 alz71232-tbl-0001:** Group differences in demographic and health variables employed to calculate frailty index.

Variable	CU	AD	FTLD	Statistics	Comparisons
Sample size	1924	1126	411		
Age	58.50 (13.58)	70.91 (8.24)	66.31 (8.23)	*F*(2, 3259) = 414.41, *p* < 0.001	AD > FTLD > CU
Sex (F:M)	1338:573	733:390	206:203	*χ* ^2^ (2, 3260) = 61.62, *p* < 0.001	
Years of education	15.71 (2.97)	13.63 (3.50)	14.14 (3.56)	*F*(2, 3150) = 38.99, *p* < 0.001	CU > FTLD > AD
Anxiety (GAD‐7)	3.34 (3.85)	3.34 (4.07)	5.11 (5.32)	*F*(2, 3212) = 28.977, *p* < 0.001	FTLD > AD & CU
Depression (GDS‐SF)	2.5 (2.62)	3.35 (2.86)	4.62 (3.69)	*F*(2, 3238) = 95.342, *p* < 0.001	FTLD > AD > CU
Neuropsychiatric symptoms (NPI‐Q)	1.36 (2.55)	6.72 (5.59)	10.44 (7.02)	*F*(2, 3436) = 977.277, *p* < 0.001	FTLD > AD > CU
Cognition (MMSE)	27.75 (2.69)	20.54 (4.59)	21.03 (6.63)	*F*(2, 3318) = 1276.473, *p* < 0.001	AD & FTLD > CU
Functionality (PFAQ)	0.19 (1.28)	12.85 (8.16)	13.87 (9.46)	*F*(2, 3230) = 2021.518, *p* < 0.001	FTLD > AD > CU
Basic functionality (T‐ADLQ)	0.06 (0.4)	1.61 (2.34)	3 (3.77)	*F*(2, 3442) = 526.049, *p* < 0.001	FTLD > AD > CU
Instrumental functionality (T‐ADLQ)	0.65 (2.07)	21.45 (14.09)	23.76 (17.55)	*F*(2, 3442) = 1891.789, *p* < 0.001	FTLD > AD > CU
Advanced functionality (T‐ADLQ)	0.54 (1.39)	6.42 (5.04)	7.4 (6.01)	*F*(2, 3442) = 1188.507, *p* < 0.001	FTLD > AD > CU
Body mass index	1.84 (0.74)	1.63 (0.7)	1.68 (0.69)	*F*(2, 3234) = 30.467, *p* < 0.001	AD & FTLD > CU
Resting heart rate	70.87 (17.27)	70.74 (11.41)	70.38 (9.85)	*F*(2, 2999) = 0.163, *p* = 0.850	
Systolic blood pressure	120.18 (16.36)	124.76 (16.98)	119.64 (15.54)	*F*(2, 2992) = 27.447, *p* < 0.001	AD > FTLD & CU
Diastolic blood pressure	74.02 (9.9)	73.99 (10.2)	72.19 (9.29)	*F*(2, 2992) = 5.226, *p* = 0.005	CU & AD > FTLD
Number of diagnoses	1.7 (1.67)	1.98 (2.11)	1.77 (1.77)	*F*(2, 3457) = 8.546, *p* < 0.001	AD > CU
Number of medications	1.71 (2.07)	4.05 (3)	3.66 (3.01)	*F*(2, 3457) = 338.001, *p* < 0.001	AD > FTLD > CU
History of hypertension	1463:460	636:490	301:110	*χ* ^2^ (2,3461) = 131.620, *p* < 0.001	AD vs. CU & FTLD
History of diabetes	1756:167	959:167	350:61	*χ* ^2^ (2,3461) = 31.946, *p* < 0.001	CU vs. AD & FTLD
History of dyslipidemia	1706:217	876:250	352:59	*χ* ^2^ (2,3461) = 65.928, *p* < 0.001	AD vs. CU & FTLD
Visual health	589:1212:19	324:741:21	120:255:8	*χ* ^2^ (2,3290) = 6.543, *p* = 0.162	
Auditory health	1727:69:23	995:64:26	342:24:16	*χ* ^2^ (2,3287) = 6.543, *p* < 0.001	CU vs. AD & FTLD
Ever smoked	1144:711	632:465	204:184	*χ* ^2^ (2,3341) = 12.880, *p* = 0.002	CU versus FTLD
Currently smoking	1687:162	1012:80	357:29	*χ* ^2^ (2,3328) = 2.124, *p* = 0.346	
Cigarettes per day	3.03 (7.35)	3.66 (7.92)	4.3 (9.29)	*F*(2, 3321) = 5.273, *p* = 0.005	FTLD > CU
Substance abuse	1779:39	1056:25	352:29	*χ* ^2^ (2,3281) = 35.763, *p* < 0.001	FTLD vs. AD & CU
Frailty index	0.14 (0.065)	0.24 (0.075)	0.27 (0.10)	*F*(2, 3458) = 948.9, *p* < 0.001	FTLD > AD > CU

Note: Means and standard deviations in parentheses are reported for (CU), AD, and (FTLD) groups. Group comparisons were assessed using one‐way ANOVA for continuous variables and chi‐squared test for categorical variables. Significant post hoc comparisons (Tukey's Honestly Significant Difference) are summarized using directionality to indicate higher or lower values between groups.

Abbreviations: AD, Alzheimer's disease; CDR, Clinical Dementia Rating; CU, cognitively unimpaired; FTLD, frontotemporal lobar degeneration; GAD‐7, anxiety via the Geriatric Depression Scale; GDS‐SF, Geriatric Depression Scale; PFAQ, Pfeffer Functional Activities Questionnaire; MMSE, Mini‐Mental State Examination; NPI‐Q, Neuropsychiatric Inventory Questionnaire; T‐ADLQ; Technology‐Activities of Daily Living Questionnaire.

Conditions other than AD or FTLD, scores below 14 on the Mini‐Mental State Examination (MMSE), and the presence of impairments hindering task completion (e.g., severe language, communication, visual or hearing impairment) were considered exclusion criteria for study participation. Clinical criteria for AD originated from the National Institute on Aging‐Alzheimer's Association workgroups on diagnostic guidelines for AD^31^ and for FTLD from revised clinical criteria.^32^ CU participants have no history of neurological or psychiatric disorders or impaired cognitive function. Diagnoses were defined by healthcare professional consensus, considering clinical interviews, cognitive and neurological assessments, and magnetic resonance imaging (MRI).^23^

Several steps were taken to ensure assessment comparability across sites.^23^ Clinician evaluations were acquired using a standardized assessment battery. Rigorous quality control protocol, training, and certification were provided to all clinicians. Clinical and cognitive evaluations were standardized across all ReDLat sites. The executive committee of the ReDLat consortium and the Institutional Review Boards at each recruiting site provided approval for this study. In accordance with the Declaration of Helsinki, informed consent was obtained from all participants.

### 2.2 Frailty index

Following a standard procedure,^24^ we developed a score indexing the accumulation of health deficits across multiple domains. The frailty index aggregates a wide range of health variables into a singular continuous score, ranging from 0 (no deficit) to 1 (deficit). We selected 32 measurable health variables suitable for inclusion in the frailty index.^24^ Selected health variables integrate multiple physiological systems^24^ in alignment with standard comprehensive assessment criteria for signs, symptoms, diseases, or disabilities.^33^

The identified health variables span six key domains: physical health (seven cardiometabolic items,^34^, ^35^ two auditory and visual health items,^36^ two items for number of medications^37^ and diagnoses),^33^ five health behavior items,^24^, ^37^ eight global clinical status items,^38^ and three clinical assessments (a neuropsychiatric,^38^, ^39^ depression,^37^, ^38^ and anxiety assessment,^40^ one cognition item,^34^ and four functional ability items^41^). Physical health includes cardiometabolic measures (blood pressure, heart rate, body mass index), sensory impairments (visual and auditory status), and number of diagnoses and medications, with data primarily extracted from clinical records or questionnaires. Health behaviors cover smoking and substance use, both assessed via self‐report and known to correlate with frailty outcomes. Global clinical status is indexed using the Clinical Dementia Rating (CDR) scale, while neuropsychiatric symptoms are evaluated via the Neuropsychiatric Inventory Questionnaire (NPI‐Q). Depressive symptoms are measured using the Geriatric Depression Scale‐Short Form (GDS‐SF), and anxiety via the Geriatric Depression Scale (GAD‐7), both validated tools in aging populations. Functional abilities are assessed with the Pfeffer Functional Activities Questionnaire (PFAQ) and the Technology‐Activities of Daily Living Questionnaire (T‐ADLQ), capturing instrumental and technology‐related activities. Cognitive function is measured using the MMSE. All health variables and instruments were previously employed to calculate the frailty index^24^, ^33^, ^34^, ^35^, ^36^, ^37^, ^38^, ^39^, ^40^, ^41^ and are validated and widely used in research across Latin American and Caribbean populations.^42^, ^43^, ^44^, ^45^, ^46^, ^47^, ^48^, ^49^, ^50^, ^51^, ^52^, ^53^, ^54^, ^55^, ^56^, ^57^ See Supporting Information 1 for a description of each assessment.

### 2.3 Frailty index scoring procedure

We selected 32 health variables for inclusion in the frailty index, in accordance with standard procedures recommending the inclusion of at least 30 health variables to maximize reliability.^24^, ^58^ All selected health variables were rescored on a binarized scale from 0 (no deficit) to 1 (deficit). Standard criteria^24^ were used to corroborate that the presence of deficits reported for each health variable was not too rare (<1% reporting a deficit) or too common (>80% reporting a deficit). We also corroborated that health variables had no more than 15% missing values and were not too highly correlated (*r* ≥ 0.8). See Figures S1–S3 for the correlation plot between all health variables per group. Variance inflation factor analysis was used to further avoid multicollinearity, with a threshold value of 10^59^, ^60^ (Supporting Information 2). Frailty index scores were computed by adding up the values of the observed health variables and dividing this sum by the number of variables observed. Following standard guidelines,^24^ this calculation was performed only for participants with at least 80% observed values across health variables, resulting in the final selection of 3461 participants included in the study from a total sample of 3549. The syntax developed for the frailty index calculation can be found in the Open Science Framework (OSF) database https://osf.io/natbh.

### 2.4 Neuroimaging acquisition and preprocessing

#### 2.4.1 MRI preprocessing

T1‐weighted structural MRI scans were available for 1050 individuals, including 506 CU, 383 AD, and 161 FTLD, and across six countries: Argentina (*n* = 118, CU:AD/FTLD = 52:66), Brazil (*n* = 154, CU:AD/FTLD = 91:63), Chile (*n* = 141, CU:AD/FTLD = 66:75), Colombia (*n* = 238, CU:AD/FTLD = 30:208), Mexico (*n* = 56, CU:AD/FTLD = 32:24), and Peru (*n* = 343, CU:AD/FTLD = 235:108). See Table S1 for recording parameters per site. Standard preprocessing procedures were performed through voxel‐based morphometry and Computational Anatomy (CAT12)^61^ and Statistical Parametric Mapping (SPM) software (version 12; Wellcome Centre for Human Neuroimaging in MATLAB R2021a). Bias‐field correction, noise reduction, segmentation, and brain extraction protocols were carried out. Images were normalized to standard space using the Montreal Neurological Institute template at 0.5 mm isotropic resolution, and data were normalized to the mean global intensity and harmonized across all subjects. Gray matter segmentations were then smoothed using a 6 × 6 × 6 mm Gaussian kernel. The homogeneity and orthogonality of all images were verified. Finally, regional GMVs were extracted for each participant based on the Automated Anatomical Labeling (AAL) atlas and adjusted for total intracranial volume (TIV) using the CAT12 region of interest (ROI) extraction pipeline, which computes volume estimates by summing modulated gray matter segments within each ROI and scaling them relative to individual TIV to account for head size variability.

#### 2.4.2 Resting‐state fMRI preprocessing

Resting‐state functional MRI (fMRI) recordings were available for 667 individuals, including 254 CU, 305 AD, and 108 FTLD, across five countries: Argentina (*n* = 116, CU:AD/FTLD = 51:58:7), Brazil (*n* = 134, CU:AD/FTLD = 90:22:22), Chile (*n* = 146, CU:AD/FTLD = 60:60:26), Colombia (*n* = 233, CU:AD/FTLD = 29:156:48), and Mexico (*n* = 38, CU:AD/FTLD = 24:9:5). See Table S2 for recording parameters per site. In accordance with the fMRIprep standard pipeline,^62^ image preprocessing was conducted for motion correction, slice timing and susceptibility distortion correction, co‐registration of anatomical and functional images, and normalization to standard space. To reduce noise and confounds, signal intensity was normalized, and spatial smoothing was performed with a 6 × 6 × 6 Gaussian kernel. A bandpass filter (0.008 to 0.09 Hz) was applied, and additional steps for this process were done in the CONN22.a toolbox. Through a protocol derived from the AAL atlas, Pearson correlation coefficients were calculated between the average BOLD time series of each ROI pair. This generated a 116 × 116 correlation matrix for each subject. The correlation matrices were transformed using Fisher's z‐transformation to approximate a standard normal distribution of the correlation coefficients.

## 3 STATISTICAL ANALYSIS

### 3.1 Logistic regression

An XGBoost binary logistic regression analysis was conducted, including the frailty index and frailty features as predictors in separate models. This gradient boosting algorithm enables parallel computation tree boosting, delivering precise predictions and avoiding overfitting.^63^ First, the training model and hyperparameters were defined using Bayesian optimization. A k‐fold cross‐validation (*k* = 10) was then conducted using 80% of our dataset to train the model, 10% to test, to enhance model performance.^64^ This process optimizes the XGBoost hyperparameters by running a Bayesian search (BayesSearchCV) on the training data with a 10‑fold stratified cross‐validation using the area under the receiver operating characteristic (ROC) curve (AUC) as the scoring metric, selects the best parameter combination, fits the model to the full training set, and then returns predictions and evaluation metrics on the held‑out test set. Given the imbalance between sample size across groups (Table 1), class imbalance was explicitly addressed during model training using class‐weighted optimization (scale_pos_weight), defined for each binary comparison as the ratio between the number of negative and positive samples in the training data. This approach increases the penalty for misclassification of the minority class within the loss function, reducing the tendency of the model to favor the majority class during optimization. To further characterize performance under class imbalance, row‐normalized confusion matrices, balanced accuracy, and probabilistic calibration using the Brier score were computed. We implemented SHapley Additive exPlanations (SHAP) analysis to interpret model outputs.^65^ After Bayesian tuning with BayesSearchCV, the XGBoost model was refit once on the full training partition (80% of the data). SHAP values were then calculated a single time on this training set. These values provide a unified framework to quantify the contribution of each feature to individual predictions, enhancing model transparency and facilitating the identification of key predictors. All procedures were implemented using the XGBoost package in Python. All models were run using XGBoost and SHAP libraries and modules in Python.

### 3.2 Gray matter associations with frailty

A series of linear regression models were performed to examine the association between frailty index and GMV across 116 AAL atlas^66^ brain regions. This parcellation has been widely utilized in brain network research on AD and FTLD across structural and functional imaging studies.^30^, ^67^, ^68^, ^69^, ^70^ As no single parcellation method consistently outperforms others across metrics,^71^ we selected the AAL atlas to ensure comparability with existing literature. This choice also allowed us to use the same atlas for structural and functional imaging analyses, aligning our study with previous works in dementia.^30^, ^67^, ^68^, ^69^, ^70^, ^72^, ^73^ For each region, a separate ordinary least‐squares (OLS) model was estimated using the frailty index as the main predictor and scanner type as covariate. For each model, we extracted the model adjusted *R* ^2^ and *p* value and the frailty index *t*‐value and *p* value. Associations were considered when both the *p* value of the model including the frailty index and covariates and the *p* value of the frailty index as a predictor were <0.05. Multiple comparisons were corrected using the false discovery rate (FDR) for the overall model. Brain imaging results were visualized with MRIcroGL (version 1.2.20220720).

To assess whether the association between frailty and GMV differed across groups, OLS regression models were fitted separately within each group with repeated subsampling (*n* = 1000), without replacement, to derive the empirical distributions of the *t*‐statistics for the frailty effect at each ROI.^74^, ^75^, ^76^ These group‐specific distributions were directly contrasted between groups using independent‐sample *t*‐tests, restricted to regions where significant associations between frailty and GMV were observed in at least one group subject to pairwise comparison (CU vs AD, CU vs FTLD, AD vs FTLD). Multiple comparisons across regions were controlled using FDR correction.

### 3.3 Resting‐state functional connectivity associations with frailty

Linear regression analyses were conducted to evaluate the relationship between frailty and whole‐brain functional connectivity, defined as pairwise connectivity strength between ROIs from the AAL atlas. Consistent with prior methodologies,^77^, ^78^ a separate OLS model was fitted for each ROI‐to‐ROI connection, with frailty as the primary independent variable and scanner type as covariate. The same extraction of model metrics and consideration of associations were employed as for the gray matter associations with frailty. The same subsampling‐based procedure and between‐group contrast framework described for gray matter analyses were applied to functional connectivity to formally test group differences in frailty–connectivity associations. Visualizations were generated with BrainNet Viewer.^79^

### 3.4 Control for scanner effects and data quality

Scanner type was included as a set of dummy‐coded covariates into gray matter and functional connectivity predictive models to account for acquisition‐related variability.^69^ For MRI recording quality assessment, we calculated the spatial signal‐to‐noise ratio (SNR) computed across image slices.^80^, ^81^ The SNR was defined as the mean signal intensity of brain voxels divided by the standard deviation of that signal, providing a voxel‐wise measure of signal consistency. This was derived using the overall data quality (ODQ) FUS metric,^82^, ^83^, ^84^ which enables standardized evaluation of image quality across individuals and sites. Quality assessment for resting‐state fMRI recordings was evaluated using both spatial and temporal SNR, as well as head motion parameters. Temporal SNR (tSNR) was calculated by dividing the fMRI time series into 20‐TR segments, then computing the mean signal within each segment and dividing it by its standard deviation.^85^ This approach captures signal stability over time and is sensitive to physiological and motion‐related noise. All fMRI quality metrics were extracted using the ODQ pipeline to ensure consistent processing.^82^, ^83^, ^84^

### 3.5 Sensitivity analysis

To control for potential circularity and confounding due to clinical variables inherently associated with dementia diagnosis, we recalculated three separate frailty indexes excluding (a) severity (CDR); (b) CDR and cognition (MMSE); and (c) CDR, MMSE, anxiety (GAD‐7), and depression (GDS‐SF). Then we reran the binary logistic regression analysis. All classification analyses based on frailty features excluded the CDR to avoid circularity. Additionally, analysis of covariance (ANCOVA) models with pairwise post hoc comparisons using Tukey's Honestly Significant Difference were conducted to examine group differences in frailty while controlling for age, sex, and years of education as covariates.

For neuroimaging analysis, all primary models evaluating associations between frailty and brain structure and functional connectivity were adjusted for inter‐scanner variability by including scanner type as a dummy‐coded covariate. To control for potential circularity with clinical severity, additional models were estimated, adjusting for CDR total score in addition to scanner type. Potential confounding by image quality was addressed by running additional models adjusting for structural and temporal SNR in addition to scanner type and correlating frailty with these quality metrics. Multiple comparisons were adjusted by FDR.

## 4 RESULTS

### 4.1 Frailty in dementia

**FIGURE 1:**
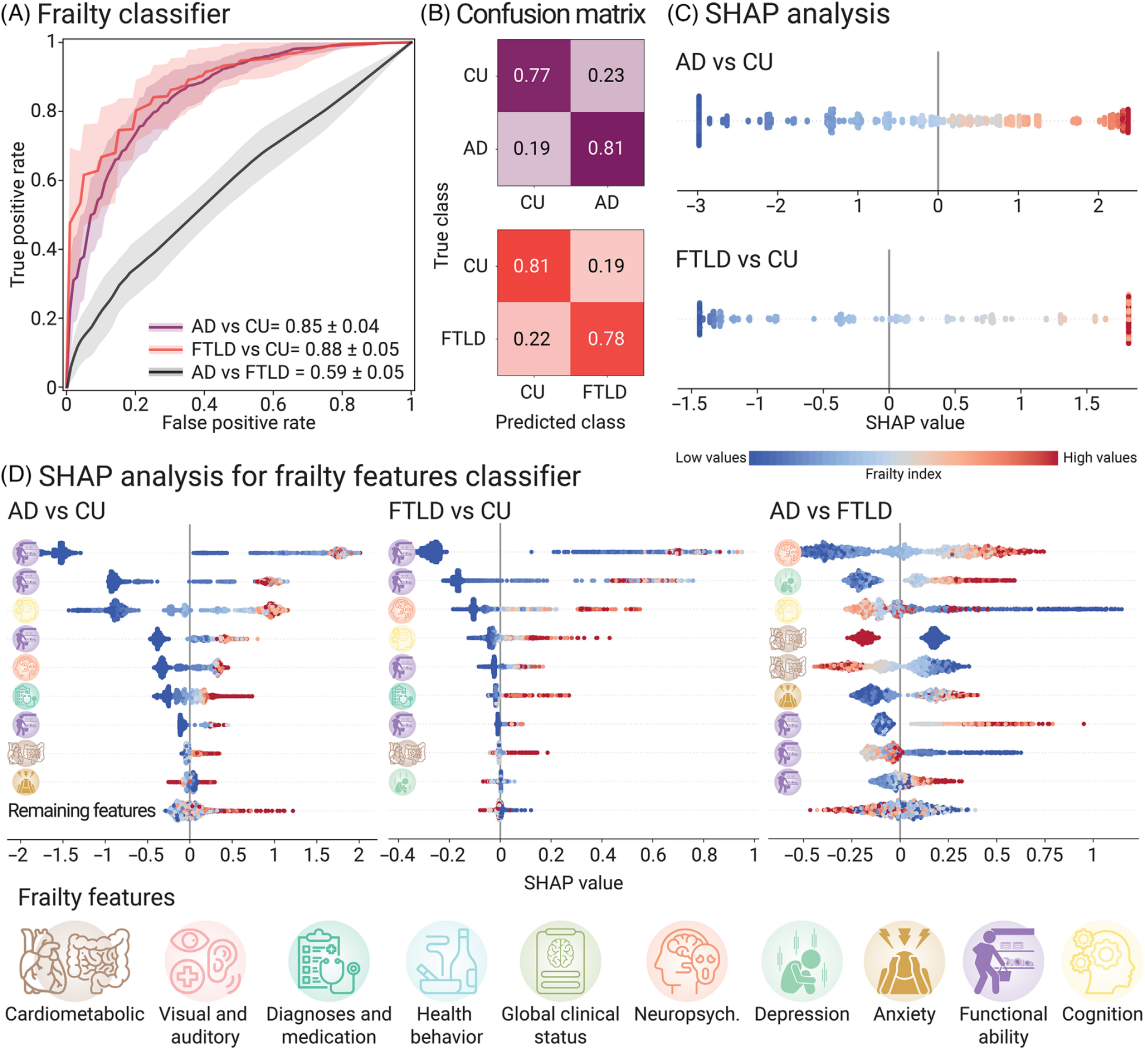
Frailty classification performance. (A) Receiver operating characteristic (ROC) curves showing performance of frailty‐based classifier in distinguishing Alzheimer's disease (AD) from cognitively unimpaired individuals (CU; purple), frontotemporal lobar degeneration (FTLD) from CU (red), and AD from FTLD (black). Shaded areas indicate the standard deviation of the area under the curve (AUC) across cross‐validation folds. (B) Row‐normalized confusion matrices summarizing classification accuracy for each pairwise comparison, averaged across cross‐validation folds. (C) SHapley Additive exPlanations (SHAP) summary plots illustrating contribution of global frailty index to model predictions for each binary classification task. Each point represents an individual observation, colored by the frailty index value (low to high), with SHAP values reflecting the direction and magnitude of each observation's contribution to the predicted class. (D) SHAP summary plots for individual frailty domains contributing to classification between groups (AD vs CU, FTLD vs CU, and AD vs FTLD). Features are ranked by mean absolute SHAP value, indicating their relative importance in the model. Positive SHAP values indicate a contribution to the disease class, whereas negative values indicate a contribution to the comparison group. Frailty domains are color‐coded by conceptual category, with the remaining features aggregated for clarity.

Frailty demonstrated robust performance in classifying AD versus CU, achieving a mean AUC of 0.85 ± 0.04. The AUC scores ranged from 0.77 to 0.94 across folds, indicating consistent prediction accuracy (Figure 1A). Accuracy, sensitivity, specificity, precision, and F1 metrics corroborated the model performance (Table 2). SHAP analysis showed that lower frailty (negative SHAP values) increased the likelihood of CU classification, while higher frailty favored AD classification (Figure 1B). At the frailty features level, the model achieved a mean AUC of 0.99 ± 0.01, with higher functional and cognitive impairment, more pronounced neuropsychiatric and anxiety symptoms, greater number of diagnoses and medications, and cardiometabolic burden as the strongest contributors to AD classification (Figure 1C).

**TABLE 2:** Performance metrics of frailty‐based models for diagnostic classification. Classification performance of frailty index‐based logistic models distinguishing Alzheimer's disease (AD) from cognitively unimpaired individuals (CU), frontotemporal lobar degeneration (FTLD) from CU, and AD from FTLD.

Performance metric	AD versus CU	FTLD versus CU	AD versus FTLD
Accuracy	0.77	0.81	0.70
Balanced accuracy	0.79	0.79	0.56
Calibration (Brier score)	0.16	0.14	0.24
Sensitivity (recall)	0.80	0.78	0.28
Specificity	0.74	0.84	0.84
Precision	0.76	0.83	0.38
F1 Score	0.78	0.80	0.32

When classifying FTLD and CU, the model achieved a mean AUC of 0.88 ± 0.05, with individual fold AUC scores ranging from 0.78 to 0.95, indicating reliable performance (Figure 1A). The metrics of accuracy, sensitivity, specificity, precision, and F1 confirmed the results (Table 2). SHAP analysis indicated that lower frailty increased the likelihood of being classified as CU, whereas higher frailty enhanced the likelihood of FTLD classification (Figure 1B). At the frailty features level, the model achieved a mean AUC of 0.98 ± 0.01, where the top contributing frailty features included greater functional impairment and neuropsychiatric symptoms, lower cognitive functioning, a higher number of diagnoses and medication, greater cardiometabolic burden, and increased depressive symptoms, which increased the probability of FTLD classification (Figure 1C).

Frailty levels between AD and FTLD yielded low discriminatory values (mean AUC of 0.59 ± 0.05; Figure 1A). AUC values ranged from 0.51 to 0.68, with lower values of accuracy, sensitivity, specificity, precision, and F1 (Table 2). At the frailty features level, the model achieved a mean AUC of 0.76 ± 0.04, with neuropsychiatric and depressive symptoms, cognition, cardiometabolic burden, anxiety symptoms, and functional impairment as the most discriminative features (Figure 1C).

### 4.2 Associations between frailty and brain volume

**FIGURE 2:**
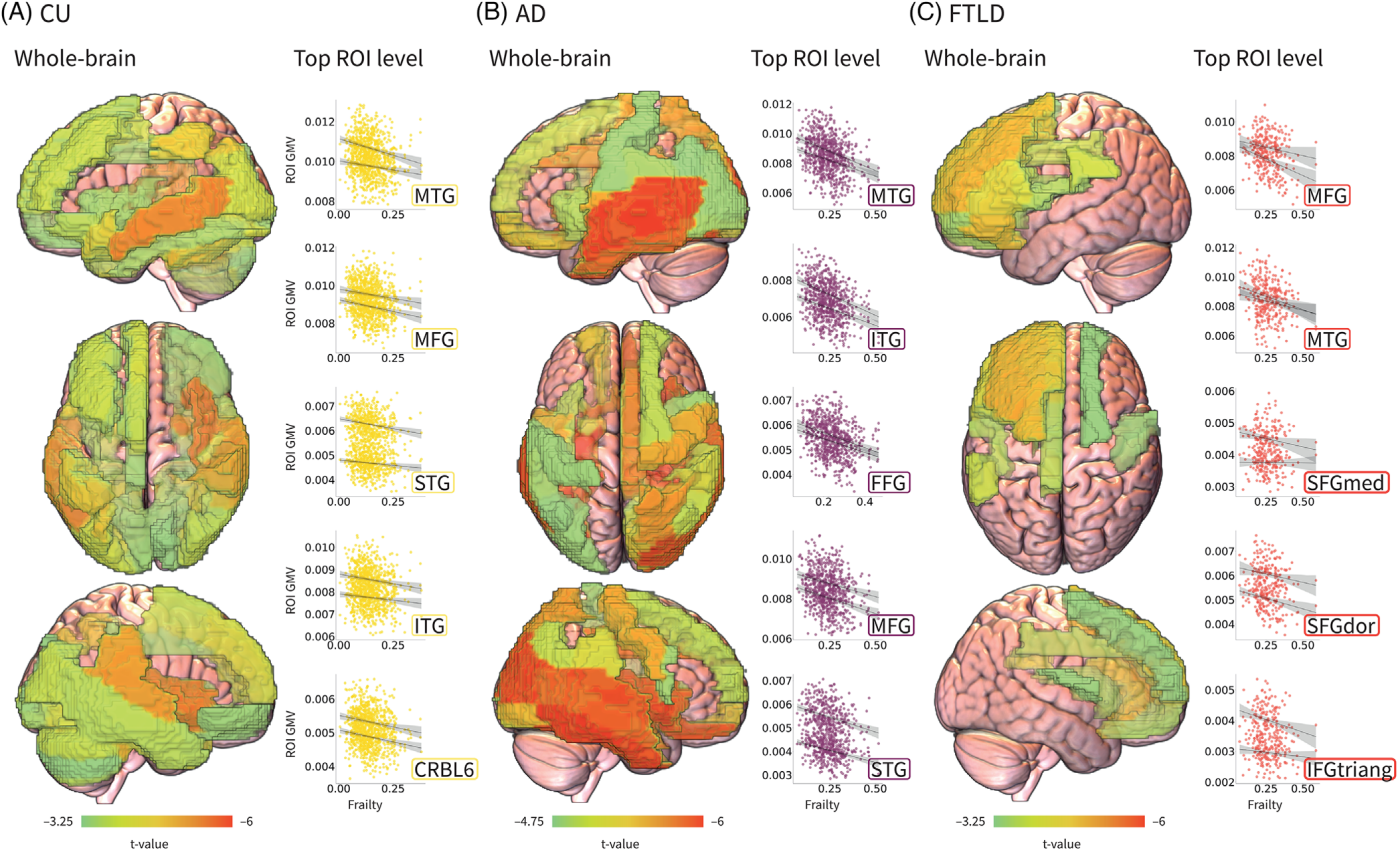
Association between frailty and brain structure. Whole‐brain gray matter volume (GMV) maps display *t*‐values from ordinary least‐squares models estimating association between frailty and regional GMV for cognitively unimpaired (CU; A), Alzheimer's disease (AD; B), and frontotemporal lobar degeneration (FTLD; C). For each group, the five regions of interest (ROIs) with the strongest associations are shown with scatterplots of frailty scores (*x*‐axis) against regional GMV (*y*‐axis), along with linear regression fits for both hemispheres and 95% confidence intervals. CRBL6, cerebellar lobule VI; FFG, fusiform gyrus; IFGtriang, inferior frontal gyrus, triangular part; ITG, inferior temporal gyrus; MFG, middle frontal gyrus; MTG, middle temporal gyrus; SFGdor, superior frontal gyrus, dorsolateral; SFGmed, superior frontal gyrus, medial; STG, superior temporal gyrus.

In CU, higher frailty scores were associated with localized reductions in brain volume, predominantly temporal, frontal, and cerebellar lobes. Top associations were observed in the insular cortex, where the strongest association was observed in the right insula (*t* = −5.477). Temporal lobe involvement included the left middle temporal gyrus (*t* = −5.35), the right superior temporal gyrus (*t* = −5.029), and the left superior temporal pole (*t* = −4.217). Limbic regions were also implicated, with significant associations in the right amygdala (*t* = −4.996) and the left amygdala (*t* = −4.009). Parietal lobe associations included the right supramarginal gyrus (*t* = −4.93) and the left inferior parietal lobule (*t* = −4.396). Cerebellar regions showed consistent associations, including the right cerebellar lobules 4–5 (*t* = −4.83) and cerebellar lobule 6 bilaterally, on the right (*t* = −4.693) and left (*t* = −4.634). In occipital and occipito‐temporal regions, significant associations were identified in the left lingual gyrus (*t* = −4.043), the right fusiform gyrus (*t* = −4.031), and the left middle occipital gyrus (*t* = −3.968). Frontal lobe involvement was observed in the left medial superior frontal gyrus (*t* = −3.999). Additional associations were found in the occipital lobe (medial, calcarine sulcus), parietal areas (inferior, postcentral and supramarginal gyrus), insula, and fusiform gyrus (Figure 2A, Table 3).

**TABLE 3 alz71232-tbl-0003:** Frailty on gray matter volume for CU. *P* value adjusted by the false discovery rate (*P*_FDR_) < 0.05, covariate: recording site. Regions are presented using Automated Anatomical Labeling atlas.

	Model		Frailty	
Region	***R* ^2^_adj_**	***P*_FDR_**	***t***	***P*_FDR_**
Precentral L	0.113	<0.001	−2.443	0.022
Frontal Sup L	0.211	<0.001	−3.241	0.004
Frontal Sup Orb L	0.146	<0.001	−3.140	0.005
Frontal Sup Orb R	0.141	<0.001	−3.325	0.003
Frontal Mid L	0.204	<0.001	−3.896	0.001
Frontal Mid Orb L	0.158	<0.001	−2.257	0.035
Frontal Mid Orb R	0.153	<0.001	−3.373	0.003
Frontal Inf Tri L	0.087	<0.001	−3.145	0.005
Frontal Inf Orb L	0.092	<0.001	−3.708	0.001
Frontal Inf Orb R	0.101	<0.001	−3.384	0.003
Supp Motor Area L	0.105	<0.001	−3.805	0.001
Olfactory L	0.123	<0.001	−2.765	0.011
Frontal Sup Med L	0.146	<0.001	−3.999	0.001
Frontal Med Orb L	0.093	<0.001	−3.085	0.005
Rectus L	0.093	<0.001	−2.744	0.012
Rectus R	0.090	<0.001	−2.433	0.023
Insula L	0.046	<0.001	−3.499	0.002
Insula R	0.104	<0.001	−5.477	<0.001
Cingulum Mid L	0.062	<0.001	−3.383	0.003
Hippocampus L	0.128	<0.001	−3.574	0.002
Hippocampus R	0.188	<0.001	−3.326	0.003
ParaHippocampal R	0.087	<0.001	−3.174	0.004
Amygdala L	0.110	<0.001	−4.009	0.001
Amygdala R	0.164	<0.001	−4.996	<0.001
Calcarine L	0.294	<0.001	−3.416	0.003
Calcarine R	0.166	<0.001	−3.363	0.003
Cuneus L	0.301	<0.001	−3.171	0.004
Cuneus R	0.231	<0.001	−3.273	0.003
Lingual L	0.179	<0.001	−4.043	0.001
Lingual R	0.188	<0.001	−3.524	0.002
Occipital Sup L	0.152	<0.001	−2.552	0.018
Occipital Sup R	0.142	<0.001	−2.864	0.009
Occipital Mid L	0.158	<0.001	−3.968	0.001
Occipital Mid R	0.183	<0.001	−3.850	0.001
Occipital Inf L	0.076	<0.001	−2.608	0.016
Occipital Inf R	0.141	<0.001	−2.190	0.041
Fusiform L	0.118	<0.001	−3.574	0.002
Fusiform R	0.167	<0.001	−4.031	0.001
Postcentral L	0.154	<0.001	−3.235	0.004
Parietal Inf L	0.105	<0.001	−4.396	<0.001
Parietal Inf R	0.156	<0.001	−2.879	0.008
SupraMarginal L	0.032	0.003	−2.485	0.020
SupraMarginal R	0.109	<0.001	−4.930	<0.001
Angular R	0.156	<0.001	−3.040	0.006
Caudate L	0.050	<0.001	−2.614	0.016
Putamen L	0.180	<0.001	−2.556	0.018
Putamen R	0.186	<0.001	−3.106	0.005
Pallidum L	0.098	<0.001	2.490	0.020
Thalamus L	0.268	<0.001	−2.485	0.020
Thalamus R	0.227	<0.001	−2.640	0.015
Heschl L	0.042	<0.001	−2.956	0.007
Heschl R	0.127	<0.001	−3.758	0.001
Temporal Sup L	0.059	<0.001	−3.026	0.006
Temporal Sup R	0.097	<0.001	−5.029	<0.001
Temporal Pole Sup L	0.053	<0.001	−4.217	<0.001
Temporal Pole Sup R	0.041	<0.001	−2.969	0.007
Temporal Mid L	0.142	<0.001	−5.350	<0.001
Temporal Mid R	0.181	<0.001	−3.727	0.001
Temporal Pole Mid L	0.026	0.008	−2.934	0.007
Temporal Pole Mid R	0.073	<0.001	−3.635	0.001
Temporal Inf L	0.125	<0.001	−2.735	0.012
Temporal Inf R	0.260	<0.001	−3.697	0.001
Cerebellum Crus1 L	0.071	<0.001	−3.129	0.005
Cerebellum Crus1 R	0.079	<0.001	−3.832	0.001
Cerebellum Crus2 L	0.040	0.001	−2.405	0.024
Cerebellum Crus2 R	0.037	0.001	−3.268	0.003
Cerebellum 3 L	0.089	<0.001	−2.616	0.016
Cerebellum 3 R	0.091	<0.001	−3.380	0.003
Cerebellum 4 5 L	0.103	<0.001	−3.876	0.001
Cerebellum 4 5 R	0.113	<0.001	−4.830	<0.001
Cerebellum 6 L	0.113	<0.001	−4.634	<0.001
Cerebellum 6 R	0.140	<0.001	−4.693	<0.001
Cerebellum 7b L	0.034	0.002	−2.618	0.016
Cerebellum 7b R	0.058	<0.001	−3.683	0.001
Cerebellum 8 L	0.064	<0.001	−2.545	0.018
Cerebellum 8 R	0.068	<0.001	−3.065	0.005
Cerebellum 10 L	0.033	0.003	−2.670	0.014
Cerebellum 10 R	0.026	0.008	−2.991	0.006
Vermis 3	0.326	<0.001	−2.110	0.049
Vermis 4 and 5	0.101	<0.001	−2.971	0.007
Vermis 6	0.066	<0.001	−2.743	0.012
Vermis 7	0.129	<0.001	−2.487	0.020
Vermis 8	0.166	<0.001	−2.228	0.038
Vermis 9	0.165	<0.001	−2.156	0.044

Abbreviations: Inf, inferior; L, left; Med, medial; Mid, middle; Orb, orbital; R, right; Sup, superior; Supp, supplementary; Tri, triangular (pars triangularis).

For AD, higher frailty scores were associated with widespread reductions in temporal, frontal, and occipital lobes, with additional involvement of parietal and limbic regions. The strongest effects were detected in the temporal lobe, specifically the right inferior temporal gyrus (*t* = −6.925), followed by the left superior temporal gyrus (*t* = −6.313), the left middle temporal gyrus (*t* = −6.19), the right superior temporal gyrus (*t* = −5.923), and the right middle temporal gyrus (*t* = −5.813). Limbic structures showed marked involvement, with robust bilateral associations in the hippocampus, stronger on the right (*t* = −6.885) than on the left (*t* = −6.307), followed by the right amygdala (*t* = −6.316) and the right parahippocampal region (*t* = −5.851). In the insular cortex, a strong association was observed in the right insula (*t* = −6.572). Occipital and occipito‐temporal regions also demonstrated pronounced effects, led by the right fusiform gyrus (*t* = −6.43), followed by the right superior occipital gyrus (*t* = −6.339), the right middle occipital gyrus (*t* = −6.125), and the left fusiform gyrus (*t* = −6.094). Frontal lobe involvement was observed in the right rectus gyrus (*t* = −6.096). Precentral and postcentral gyrus were also associated, as well as parietal regions including the inferior parietal gyrus and precuneus (Figure 2B, Table 4).

**TABLE 4 alz71232-tbl-0004:** Frailty on gray matter volume for Alzheimer's disease. *P* value adjusted by the false discovery rate (*P*_FDR_) < 0.05, covariate: recording site. Regions are presented using the Automated Anatomical Labeling atlas.

	Model		Frailty	
Region	***R* ^2^_adj_**	***P*_FDR_**	***t***	***P*_FDR_**
Precentral L	0.193	<0.001	−4.513	<0.001
Precentral R	0.297	<0.001	−5.445	<0.001
Frontal Sup L	0.096	<0.001	−3.707	<0.001
Frontal Sup R	0.141	<0.001	−4.984	<0.001
Frontal Sup Orb L	0.153	<0.001	−5.274	<0.001
Frontal Sup Orb R	0.117	<0.001	−3.321	0.001
Frontal Mid L	0.152	<0.001	−4.628	<0.001
Frontal Mid R	0.108	<0.001	−4.198	<0.001
Frontal Mid Orb L	0.086	<0.001	−3.916	<0.001
Frontal Mid Orb R	0.077	<0.001	−3.280	0.002
Frontal Inf Oper L	0.081	<0.001	−3.811	<0.001
Frontal Inf Oper R	0.083	<0.001	−4.841	<0.001
Frontal Inf Tri L	0.068	<0.001	−4.021	<0.001
Frontal Inf Tri R	0.083	<0.001	−4.238	<0.001
Frontal Inf Orb L	0.061	<0.001	−3.379	0.001
Frontal Inf Orb R	0.105	<0.001	−4.318	<0.001
Rolandic Oper L	0.111	<0.001	−5.432	<0.001
Rolandic Oper R	0.094	<0.001	−3.896	<0.001
Supp Motor Area L	0.130	<0.001	−3.466	0.001
Supp Motor Area R	0.147	<0.001	−4.070	<0.001
Olfactory L	0.197	<0.001	−5.708	<0.001
Olfactory R	0.198	<0.001	−3.767	<0.001
Frontal Sup Med L	0.143	<0.001	−4.489	<0.001
Frontal Sup Med R	0.097	<0.001	−3.317	0.001
Frontal Med Orb L	0.074	<0.001	−3.619	0.001
Frontal Med Orb R	0.083	<0.001	−3.577	0.001
Rectus L	0.192	<0.001	−4.324	<0.001
Rectus R	0.237	<0.001	−6.096	<0.001
Insula L	0.091	<0.001	−4.941	<0.001
Insula R	0.158	<0.001	−6.572	<0.001
Cingulum Ant L	0.074	<0.001	−3.651	0.001
Cingulum Mid L	0.154	<0.001	−4.736	<0.001
Cingulum Mid R	0.159	<0.001	−5.693	<0.001
Cingulum Post L	0.177	<0.001	−2.932	0.005
Cingulum Post R	0.079	<0.001	−3.149	0.002
Hippocampus L	0.189	<0.001	−6.307	<0.001
Hippocampus R	0.220	<0.001	−6.885	<0.001
ParaHippocampal L	0.224	<0.001	−5.362	<0.001
ParaHippocampal R	0.195	<0.001	−5.851	<0.001
Amygdala L	0.149	<0.001	−5.072	<0.001
Amygdala R	0.156	<0.001	−6.316	<0.001
Calcarine L	0.285	<0.001	−3.543	0.001
Calcarine R	0.158	<0.001	−4.907	<0.001
Cuneus L	0.258	<0.001	−3.034	0.004
Cuneus R	0.259	<0.001	−5.252	<0.001
Lingual L	0.254	<0.001	−4.090	<0.001
Lingual R	0.262	<0.001	−5.119	<0.001
Occipital Sup L	0.173	<0.001	−4.561	<0.001
Occipital Sup R	0.228	<0.001	−6.339	<0.001
Occipital Mid L	0.191	<0.001	−4.810	<0.001
Occipital Mid R	0.211	<0.001	−6.125	<0.001
Occipital Inf L	0.118	<0.001	−4.864	<0.001
Occipital Inf R	0.174	<0.001	−5.344	<0.001
Fusiform L	0.246	<0.001	−6.094	<0.001
Fusiform R	0.271	<0.001	−6.430	<0.001
Postcentral L	0.241	<0.001	−4.809	<0.001
Postcentral R	0.231	<0.001	−3.757	<0.001
Parietal Sup L	0.170	<0.001	−3.445	0.001
Parietal Sup R	0.224	<0.001	−5.535	<0.001
Parietal Inf L	0.164	<0.001	−4.731	<0.001
Parietal Inf R	0.172	<0.001	−3.858	<0.001
SupraMarginal L	0.073	<0.001	−4.875	<0.001
SupraMarginal R	0.144	<0.001	−5.119	<0.001
Angular L	0.111	<0.001	−4.772	<0.001
Angular R	0.160	<0.001	−5.020	<0.001
Precuneus L	0.216	<0.001	−3.819	<0.001
Precuneus R	0.216	<0.001	−4.298	<0.001
Caudate L	0.114	<0.001	−2.957	0.005
Putamen L	0.099	<0.001	−3.268	0.002
Putamen R	0.090	<0.001	−2.706	0.009
Thalamus L	0.226	<0.001	−3.863	<0.001
Thalamus R	0.278	<0.001	−4.469	<0.001
Heschl L	0.145	<0.001	−5.478	<0.001
Heschl R	0.148	<0.001	−3.773	<0.001
Temporal Sup L	0.144	<0.001	−6.313	<0.001
Temporal Sup R	0.153	<0.001	−5.923	<0.001
Temporal Pole Sup L	0.120	<0.001	−4.255	<0.001
Temporal Pole Sup R	0.095	<0.001	−3.890	<0.001
Temporal Mid L	0.157	<0.001	−6.190	<0.001
Temporal Mid R	0.161	<0.001	−5.813	<0.001
Temporal Pole Mid L	0.119	<0.001	−4.366	<0.001
Temporal Pole Mid R	0.137	<0.001	−4.587	<0.001
Temporal Inf L	0.227	<0.001	−5.805	<0.001
Temporal Inf R	0.249	<0.001	−6.925	<0.001
Cerebellum Crus2 L	0.064	<0.001	−3.557	0.001
Cerebellum Crus2 R	0.060	<0.001	−2.904	0.005
Cerebellum 6 L	0.135	<0.001	−2.145	0.041
Cerebellum 6 R	0.124	<0.001	−2.332	0.026
Cerebellum 7b L	0.056	<0.001	−3.512	0.001
Cerebellum 7b R	0.081	<0.001	−3.157	0.002
Cerebellum 8 L	0.100	<0.001	−2.421	0.021
Cerebellum 8 R	0.112	<0.001	−2.751	0.008

Abbreviations: Ant, anterior; Inf, inferior; L, left; Med, medial; Mid, middle; Oper, opercular (pars opercularis); Orb, orbital; Post, posterior; R, right; Sup, superior; Supp, supplementary; Tri, triangular (pars triangularis).

In FTLD, frailty was associated with extensive volume reductions in frontal and temporal lobes, with additional contributions from the occipital, parietal, and limbic regions. Top associations were predominantly localized within the frontal lobe, where the strongest association was observed in the left middle frontal gyrus (*t* = −4.757), followed by the left superior frontal gyrus (*t* = −4.579), left inferior frontal gyrus pars orbitalis (*t* = −4.551), left medial superior frontal gyrus (*t* = −4.326), left inferior frontal gyrus pars triangularis (*t* = −4.219), left inferior frontal gyrus pars opercularis (*t* = −4.074), left superior orbital frontal gyrus (*t* = −3.586), left middle orbital frontal gyrus (*t* = −3.482), and the right middle frontal gyrus (*t* = −2.873). Parietal involvement was observed in the left supramarginal gyrus (*t* = −3.928), while temporal lobe involvement was limited to the left superior temporal gyrus (*t* = −3.185). Cingulate regions also showed significant associations, including the left mid‐cingulate cortex (*t* = −3.723) and the left posterior cingulate cortex (*t* = −3.176). Subcortical involvement was evident in the left caudate nucleus (*t* = −3.555). Further effects extended to the superior temporal, occipital, and insular regions. Precentral and postcentral gyrus were also involved, along with the precuneus and inferior parietal gyrus (Figure 2C, [Table alz71232-tbl-0003], [Table alz71232-tbl-0004] Table 5).

**TABLE 5:** Frailty on gray matter volume for frontotemporal lobar degeneration. *P* value adjusted by the false discovery rate (*P*_FDR_) < 0.05, covariate: recording site. Regions are presented using the Automated Anatomical Labeling atlas.

	Model		Frailty	
Region	***R* ^2^_adj_**	***P*_FDR_**	***t***	***P*_FDR_**
Frontal Sup L	0.177	<0.001	−4.579	<0.001
Frontal Sup Orb L	0.095	0.008	−3.586	0.005
Frontal Mid L	0.142	0.001	−4.757	<0.001
Frontal Mid R	0.067	0.031	−2.873	0.025
Frontal Mid Orb L	0.082	0.016	−3.482	0.006
Frontal Inf Oper L	0.079	0.018	−4.074	0.001
Frontal Inf Tri L	0.081	0.016	−4.219	0.001
Frontal Inf Orb L	0.114	0.003	−4.551	<0.001
Rolandic Oper L	0.119	0.002	−3.607	0.005
Frontal Sup Med L	0.096	0.008	−4.326	0.001
Cingulum Mid L	0.062	0.038	−3.723	0.004
Cingulum Post L	0.146	0.001	−3.176	0.011
Occipital Inf R	0.197	<0.001	−2.605	0.034
SupraMarginal L	0.078	0.018	−3.928	0.002
Precuneus L	0.058	0.046	−2.480	0.045
Caudate L	0.104	0.005	−3.555	0.005
Heschl L	0.133	0.001	−2.782	0.026
Temporal Sup L	0.131	0.001	−3.185	0.011
Temporal Mid L	0.091	0.010	−2.861	0.025

Abbreviations: Inf, inferior; L, left; Med, medial; Mid, middle; Oper, opercular (pars opercularis); Orb, orbital; Post, posterior; R, right; Sup, superior; Tri, triangular (pars triangularis).

#### 4.2.1 Group differences in frailty–brain volume associations

Stronger frailty‐related effects in AD compared to CU were primarily observed in medial temporal, posterior cingulate–precuneus, parietal, lateral temporal, and occipital regions, with the largest differences in the right superior parietal lobule (*t* = 56.28), posterior cingulate cortex (*t* = 51.42), right hippocampus (*t* = 50.38), right gyrus rectus (*t* = 47.83), and left parahippocampal cortex (*t* = 46.78). In contrast, stronger associations in CU relative to AD were predominantly localized to cerebellar and vermian regions, including the left cerebellar lobule 4–5 (*t* = −33.50), right cerebellar lobule 4–5 (*t* = −31.32), right cerebellar lobule 3 (*t* = −31.15), left cerebellar lobule 6 (*t* = −24.96), and left cerebellar lobule 10 (*t* = −23.19) (Table S3).

Regarding FTLD versus CU, stronger effects in FTLD were predominantly localized to frontal and cingulo‐opercular regions, with the largest differences observed in the left inferior frontal operculum (*t* = 57.59), left superior frontal gyrus (*t* = 54.98), left middle frontal gyrus (*t* = 54.22), left inferior frontal triangularis (*t* = 54.16), and left Rolandic operculum (*t* = 54.37). Stronger associations in CU relative to FTLD were mainly observed in cerebellar and posterior midline regions, with the most pronounced effects in the right cerebellar lobule 10 (*t* = −70.51), right cerebellar lobule 4–5 (*t* = −55.22), left cerebellar lobule 6 (*t* = −51.40), left cerebellar lobule 4–5 (*t* = −48.72), and posterior cingulate cortex (*t* = −79.46) (Table S4).

When comparing the magnitude of frailty‐related effects in AD versus FTLD, stronger effects in AD were predominantly observed in medial temporal, limbic, and posterior occipito‐temporal regions, with the largest differences in the left parahippocampal cortex (*t* = −92.51), right calcarine cortex (*t* = −78.22), right lingual gyrus (*t* = −75.80), right fusiform gyrus (*t* = −75.15), and right inferior temporal gyrus (*t* = −72.17). In contrast, stronger associations in FTLD relative to AD were mainly localized to frontal and cingulo‐opercular regions, with the most pronounced effects in the left inferior frontal orbital gyrus (*t* = 56.81), left superior frontal gyrus (*t* = 50.59), left middle frontal gyrus (*t* = 44.79), left inferior frontal triangularis (*t* = 41.26), and left inferior frontal operculum (*t* = 38.50) (Table S5).

### 4.3 Frailty and functional connectivity in aging and dementia

**FIGURE 3:**
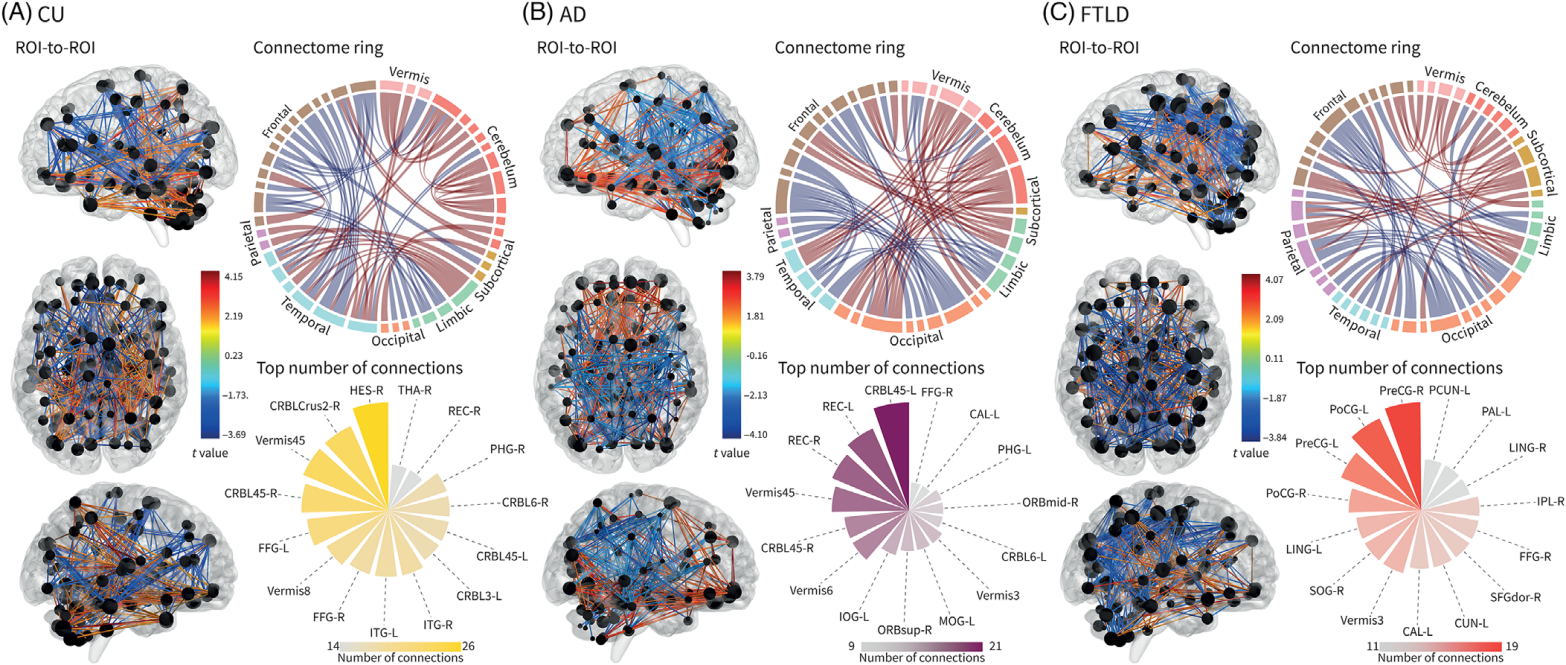
Association between frailty and brain connectivity. Region of interest (ROI)‐to‐ROI functional connectivity results are shown separately for cognitively unimpaired individuals (CU), Alzheimer's disease (AD), and frontotemporal lobar degeneration (FTLD), based on group‐specific linear regression models fitted independently for each ROI‐to‐ROI connection, with functional connectivity strength as the dependent variable, frailty as the predictor of interest, and scanner type included as a covariate. Only statistically significant associations after multiple‐comparison correction are displayed. Whole‐brain network visualizations depict the spatial distribution of frailty‐related connectivity effects, with edges color‐coded according to the direction and magnitude of the association, expressed as *t*‐values from the regression models. Circular connectome plots summarize the anatomical organization of significant connections by major lobar systems, while polar bar plots highlight the top 15 regions with the highest number of frailty‐associated connections within each group, providing a node‐level summary of network involvement. CAL‐L, calcarine gyrus, left; CRBL3‐L, cerebellar lobule 3, left; CRBL45‐L, cerebellar lobules 4 and 5, left; CRBL45‐R, cerebellar lobules 4 and 5, right; CRBL6‐L, cerebellar lobule 6, left; CRBLCrus2‐R, cerebellum crus 2, right; CUN‐L, cuneus, left; FFG‐L, fusiform gyrus, left; FFG‐R, fusiform gyrus, right; HES‐R, heschl gyrus, right; IOG‐L, inferior occipital gyrus, left; IPL‐R, inferior parietal lobule, right; ITG‐L, inferior temporal gyrus, left; ITG‐R, inferior temporal gyrus, right; LING‐L, lingual gyrus, left; LING‐R, lingual gyrus, right; MOG‐L, middle occipital gyrus, left; ORBmid‐R, middle orbital gyrus, right; ORBsup‐R, superior orbital gyrus, right; PAL‐L, pallidum, left; PCUN‐L, precuneus, left; PHG‐R, parahippocampal gyrus, right; PoCG‐L, postcentral gyrus, left; PoCG‐R, postcentral gyrus, right; PreCG‐L, precentral gyrus, left; PreCG‐R, precentral gyrus, right; REC‐L, gyrus rectus, left; REC‐R, gyrus rectus, right; SFGdor‐R, superior frontal gyrus, dorsolateral, right; SOG‐R, superior occipital gyrus, right; THA‐R, thalamus, right; Vermis3, vermis lobule 3; Vermis6, vermis lobule 6; Vermis8, vermis lobule 8; Vermis45, vermis lobules 4 and 5.

In CU, higher frailty was associated with reduced functional connectivity across frontal, temporal, occipital, and parietal regions, particularly connections between the inferior and superior frontal gyrus with temporal cortices, as well as between the cuneus, fusiform gyrus, and precentral areas. Top associations included the left inferior frontal gyrus pars triangularis–left inferior temporal gyrus (*t* = −3.685), followed by the left inferior frontal gyrus pars opercularis–left pallidum (*t* = −3.227), the right rectus gyrus–left amygdala (*t* = −3.125), the right supplementary motor area–right fusiform gyrus (*t* = −3.072), the right inferior frontal gyrus pars triangularis–right inferior temporal gyrus (*t* = −3.018), the right anterior cingulum–left amygdala (*t* = −2.983), the left superior orbital frontal gyrus–left anterior cingulum (*t* = −2.983), the left medial superior frontal gyrus–right rectus gyrus (*t* = −2.894), and the right rectus gyrus–right anterior cingulum connection (*t* = −2.854). In contrast, greater frailty was linked to increased connectivity among cerebellar, limbic, and temporal pole regions, including enhanced connectivity between crus 1/2, lobules 7b, 8, and 10, the vermis, parahippocampal gyrus, fusiform, and bilateral temporal poles. Top associations included the right rolandic operculum–right angular gyrus (*t* = 4.15), followed by the left superior orbital frontal gyrus–left cerebellar lobule 3 (*t* = 3.844), the right temporal pole (middle)–right cerebellar crus 2 (*t* = 3.84), the left cerebellar crus 2–vermis 8 (*t* = 3.774), the right Heschl gyrus–vermis 4–5 (*t* = 3.756), the left superior parietal lobule–left caudate nucleus (*t* = 3.683), the left cerebellar lobule 7b–left cerebellar lobule 10 (*t* = 3.53), the right parahippocampal gyrus–left inferior temporal gyrus (*t* = 3.523), the left cerebellar lobule 8–left cerebellar lobule 10 (*t* = 3.432), and the right cerebellar crus 2–left cerebellar lobule 8 (*t* = 3.421) (Figure 3A, Table S6). Regions with the highest number of connections were primarily located in the temporal, cerebellar, and subcortical regions. These included the Heschl gyrus, cerebellum crus 2, vermis lobules 4 and 5, cerebellar lobules 3, 4 and 5, and 6, fusiform gyrus, inferior temporal gyrus, parahippocampal gyrus, gyrus rectus, and thalamus (Figure 3A).

In AD, higher frailty was associated with reduced functional connectivity in occipital, parietal, sensorimotor, and limbic regions. Top effects were observed between the right angular gyrus and the left superior temporal pole (*t* = −4.104), followed by connections linking the left cuneus with the right superior parietal lobule (*t* = −3.459) and with the left superior parietal lobule (*t* = −3.098), the right superior occipital gyrus–right postcentral gyrus (*t* = −3.411), the right middle occipital gyrus–right postcentral gyrus (*t* = −2.972), the left mid‐cingulate cortex with the right Heschl gyrus (*t* = −3.376) and with vermis 8 (*t* = −3.073), the right precentral gyrus–right rolandic operculum (*t* = −3.259), the left rolandic operculum–left mid‐cingulate cortex (*t* = −2.888), and the left middle frontal gyrus–vermis 8 (*t* = −2.95). Greater frailty was linked to increased compensatory connectivity across cerebellar, occipital, limbic, and frontal regions. Top associations involved the left middle occipital gyrus with cerebellar lobules 4–5 on the left (*t* = 3.787), followed by the left middle occipital gyrus with vermis 4–5 (*t* = 3.667) and cerebellar lobules 4–5 on the right (*t* = 3.624), bilateral rectus–parahippocampal coupling on the left (*t* = 3.608), a frontal–frontal connection between the right middle orbital frontal gyrus and the right medial superior frontal gyrus (*t* = 3.596), the left middle occipital gyrus with vermis 3 (*t* = 3.56), the left rectus gyrus–left cerebellar lobules 4–5 (*t* = 3.45), the right fusiform gyrus with vermis 4–5 (*t* = 3.334), the right rectus gyrus–right parahippocampal gyrus (*t* = 3.271), and the right fusiform gyrus with vermis 3 (*t* = 3.216) (Figure 3B, Table S7). The most connected regions were mainly located in the occipital, cerebellar, and limbic lobes. These included cerebellare lobules 4 and 5 and 6, vermis lobules 3, 4 and 5, and 6, gyrus rectus, superior and middle orbital gyrus, parahippocampal gyrus, calcarine gyrus, middle and inferior occipital gyrus, and fusiform gyrus (Figure 3B).

In FTLD, higher frailty was associated with reduced functional connectivity primarily involving occipital, parietal, and sensorimotor regions. Decreased connectivity was most marked between the left middle occipital gyrus–right superior parietal lobule (*t* = −3.843), followed by the left middle occipital gyrus–left postcentral gyrus (*t* = −3.473), the left Rolandic operculum–right superior temporal pole (*t* = −3.427), the left precentral gyrus–right middle occipital gyrus (*t* = −3.403), the right lingual gyrus–left paracentral lobule (*t* = −3.287), the left cuneus–right cerebellar lobule 9 (*t* = −3.249), the left calcarine cortex–left precuneus (*t* = −3.212), the right superior occipital gyrus–left fusiform gyrus (*t* = −3.154), the right superior frontal gyrus–left posterior cingulum (*t* = −3.121), and the right precentral gyrus–left inferior occipital gyrus (*t* = −3.117). In contrast, greater frailty was associated with increased connectivity across subcortical, cerebellar, temporal, and limbic regions. Top associations involved the right cerebellar lobules 4–5–vermis 1–2 (*t* = 4.069), followed by the right rectus gyrus–left insula (*t* = 3.98) and the right rectus gyrus–right insula (*t* = 3.948), the right precuneus–left pallidum (*t* = 3.417), the left olfactory cortex–vermis 4–5 (*t* = 3.155), the left medial superior frontal gyrus–vermis 9 (*t* = 3.121), the right superior orbital frontal gyrus–vermis 10 (*t* = 3.037), the right supplementary motor area–left cerebellar crus 1 (*t* = 2.924), the left precuneus–left pallidum (*t* = 2.877), and the right inferior occipital gyrus–right caudate nucleus (*t* = 2.828) (Figure 3C, Table S8). The regions with the highest number of connections were predominantly located in the frontal, parietal, and occipital lobes. These included the precentral and postcentral gyrus, superior frontal gyrus, inferior parietal lobule, precuneus, pallidum, calcarine gyrus, cuneus, superior occipital gyrus, lingual gyrus, fusiform gyrus, and vermis lobule 3 (Figure 3C).

#### 4.3.1 Group differences in frailty–functional connectivity associations

Stronger frailty‐associated connectivity effects in AD were mainly observed across temporo‐parietal and parietal–limbic networks, with the largest differences linking the angular gyrus and temporal pole, opercular–parietal regions, cerebellar posterior lobules, and cingulate–auditory cortex connections. Top AD‐dominant effects were identified for connectivity between left angular gyrus and right superior temporal pole (*t* = 81.46), right angular gyrus and left superior temporal pole (*t* = 77.39), right Rolandic operculum and right angular gyrus (*t* = 76.00), right cerebellar crus 2 and right cerebellar lobule 4–5 (*t* = 71.79), and left mid‐cingulate cortex and right Heschl's gyrus (*t* = 70.94). In contrast, stronger frailty‐related connectivity effects in CU were predominantly localized to frontal–limbic, frontotemporal, and basal ganglia–cerebellum. The most pronounced CU‐dominant effects were observed for connectivity between right medial orbitofrontal cortex and left amygdala (*t* = −71.29), left gyrus rectus and left amygdala (*t* = −74.64), right Heschl's gyrus and right cerebellar lobule 10 (*t* = −78.51), right gyrus rectus and left amygdala (*t* = −85.27), and left putamen and left cerebellar lobule 4–5 (*t* = −87.19) (Table S9).

In FTLD versus CU, stronger connectivity effects in FTLD were predominantly observed across temporal pole, opercular, and occipital regions interacting with cerebellar and limbic structures, with the largest effects linking the left Rolandic operculum and right superior temporal pole (*t* = 150.01), left cuneus and right cerebellar lobule 9 (*t* = 137.66), left olfactory cortex and right superior temporal pole (*t* = 127.69), left fusiform gyrus and right middle temporal pole (*t* = 126.83), and left Heschl's gyrus and left cerebellar lobule 3 (*t* = 125.33). Stronger frailty‐related connectivity effects in CU were mainly localized to frontal–basal ganglia and limbic circuits. The most pronounced CU‐dominant effects were observed for connectivity between the left precentral gyrus and left pallidum (*t* = −123.64), left medial superior frontal cortex and right pallidum (*t* = −123.88), right postcentral gyrus and left pallidum (*t* = −127.72), left precuneus and left pallidum (*t* = −128.01), and left medial superior frontal cortex and vermis 9 (*t* = −141.87) (Table S10).

When comparing AD versus FTLD, stronger connectivity effects in FTLD were primarily distributed across occipital, opercular, and posterior cingulate regions. The most pronounced FTLD‐dominant effects were observed for connectivity between the left cuneus and right cerebellar lobule 9 (*t* = 140.91), left inferior frontal operculum and left inferior temporal gyrus (*t* = 119.87), right precentral gyrus and left inferior frontal operculum (*t* = 112.72), left Rolandic operculum and left Heschl's gyrus (*t* = 111.52), and left mid‐cingulate cortex and right superior occipital cortex (*t* = 110.68). In contrast, stronger frailty‐related connectivity effects in AD were predominantly localized to frontal–subcortical and limbic circuits. The largest AD‐dominant effects were identified for connectivity between the left precentral gyrus and vermis 8 (*t* = −134.68), left inferior parietal lobule and vermis 8 (*t* = −135.29), left medial superior frontal cortex and vermis 9 (*t* = −138.60), right gyrus rectus and left insula (*t* = −141.51), and right insula and right fusiform gyrus (*t* = −143.21 (Table S11).

### 4.4 Sensitivity analyses

Additional analyses were run to confirm the robustness of our findings. Excluding CDR from the frailty index calculation produced similar results that those of the full frailty index in classifying AD versus CU (frailty index: mean AUC = 0.84 ± 0.02), FTLD versus CU (frailty index: mean AUC = 0.84 ± 0.05), and AD versus FTLD (frailty index: mean AUC = 0.57 ± 0.03) (Table S12). Similar results were observed when also excluding the MMSE score in AD versus CU (frailty index: mean AUC = 0.81 ± 0.02; frailty features: mean AUC = 0.99 ± 0.01), FTLD versus CU (frailty index: mean AUC = 0.81 ± 0.04; frailty features: mean AUC = 0.98 ± 0.01), and AD versus FTLD (frailty index: mean AUC = 0.56 ± 0.04; frailty features: mean AUC = 0.74 ± 0.06) (Table S12). Results persisted when also excluding GAD‐7 and GDS‐SF scores in AD versus CU (frailty index: mean AUC = 0.81 ± 0.02; frailty features: mean AUC = 0.99 ± 0.01), FTLD versus CU (frailty index: mean AUC = 0.80 ± 0.05; frailty features: mean AUC = 0.99 ± 0.01), and AD versus FTLD (frailty index: mean AUC = 0.52 ± 0.03; frailty features: mean AUC = 0.71 ± 0.05) (Table S12). Differences in frailty between groups persisted after controlling for age, sex, and education (*F*[2, 3145] = 565.67, *p* < 0.001). Large effects were observed for individuals with AD compared to CU (Cohen's *d* = 1.37, *p* < 0.001), while those with FTLD presenting even larger effects (Cohen's *d* = 1.87, *p* < 0.001). The difference between AD and FTLD was also significant (Cohen's *d* = 0.52, *p* < 0.001), indicating higher frailty levels for FTLD.

Regarding neuroimaging analyses, additional models showed preserved associations after adjusting for structural and temporal data quality metrics in addition to scanner type (Tables S13–S18). Similarly, structural and temporal data quality metrics were not associated with the frailty index in all groups (Table S19). Associations with GMV and ROI‐to‐ROI functional connectivity were preserved after adjusting for global clinical status in addition to scanner type (Tables S20–S23).

## 5 DISCUSSION

We aimed to characterize the role of frailty in dementia subtypes and its structural and functional brain correlates in CU aging and dementia in Latin America. Frailty strongly differentiated AD and FTLD from CU but did not distinguish AD from FTLD. Top features for discriminating between CU and AD/FTLD were functional ability, cognition, neuropsychiatric symptoms, and the number of diagnoses and medications, while in AD versus FTLD the main distinguishing features were neuropsychiatric and depressive symptoms, cognition, and cardiometabolic factors. Higher frailty scores were associated with aging‐sensitive hubs in CU, involving frontotemporal atrophy, with additional extensions into posterior and cerebellar regions. Disease‐sensitive hubs were associated with fronto‐temporo‐occipital atrophy in AD, displaying a stronger frontotemporal component with a more pronounced temporal involvement that extended into additional posterior and occipital brain regions. In FTLD, a more prominent frontal component was observed, extending to cingulate and temporal regions. In terms of connectivity, CU showed reduced frontotemporal connections with limbic–cerebellar increases, AD exhibited marked posterior connectivity loss with occipital‐cerebellar increases, and FTLD displayed widespread connectivity disruption with partial compensatory limbic and subcortical increases. Group differences in frailty–brain associations differed across CU, AD, and FTLD, being aligned with the neurodegenerative profiles typically associated with each diagnostic group. Specifically, AD showed stronger frailty‐related associations in medial temporal, posterior cingulate–precuneus, and parietal‐occipital regions, whereas FTLD exhibited more pronounced effects in frontal and cingulo‐opercular regions. Overall, findings suggest that frailty captures a global health deterioration signal that overlaps with neurodegenerative vulnerability, providing a marker of the systemic burden of aging and dementia.

Our findings align with previous evidence indicating that frailty is associated with dementia burden.^3^, ^4^, ^5^, ^6^ The frailty index was 0.14 for CU, 0.24 for AD, and 0.27 for FTLD, in line with previous population studies where it typically falls within the 0.05 to 0.35 range.^86^, ^87^, ^88^, ^89^ While most evidence has focused on AD, here we provide the first multimodal characterization of the role of frailty in FTLD, aligned with evidence of multisystem alterations in this syndrome.^90^, ^91^, ^92^, ^93^ Similar discriminatory power and top frailty features between dementia subtypes suggest that similar frailty‐related pathophysiological processes may be present in AD and FTLD, mostly linked with shared multisystem consequences of neurodegeneration rather than disease‐specific mechanisms. Like the description of the role of frailty in AD‐specific neuropathology,^5^ findings invite future studies to characterize the role of frailty in FTLD‐specific neuropathological signatures, such as tau, TAR DNA‐binding protein 43, and fused in sarcoma (FUS).^94^ Frailty may serve as a comprehensive tool for assessing systemic decline and an integrative clinical indicator encompassing cognitive, functional, psychological, and physical health domains relevant to FTLD management and prognosis, aligned with current guidelines for assessment and management of frailty in individuals living with dementia.^95^

Discrimination findings outperformed those of previous frailty‐based studies on dementia and cognitive impairment,^38^, ^96^, ^97^, ^98^, ^99^ where AUC values of approximately 0.83 and 0.84 have been reported for discriminating between CU and AD^38^ and between CU and cognitive impairment,^99^ respectively. This may reflect the high prevalence of multimorbidity in Latin America^100^ and the relevance of clinical, cardiometabolic, and lifestyle factors in brain health.^26^, ^101^, ^102^, ^103^ The region faces a disproportionate burden of non‐communicable diseases and associated risk factors compared to other world regions. Between 2000 and 2020, Latin American countries registered the greatest increases in absolute numbers of cataract‐related blindness among adults aged ≥50 years.^104^ Cardiovascular diseases remain the leading cause of mortality, driven by population growth, aging, and a sharp rise in cardiometabolic risk factors (particularly obesity and diabetes mellitus) over the past four decades.^105^, ^106^ Recent epidemiological shifts show that cardiovascular disease incidence and mortality in Latin America is the highest in the Americas.^105^ The region also records the highest global prevalence of physical inactivity, affecting 34.3% of men and 43.7% of women, alongside the lowest overall diet quality, below the global average.^105^ Moreover, the burden of sugar‐sweetened beverage consumption is disproportionately high, with attributable fractions of 24.4% for type 2 diabetes and 11.3% for cardiovascular diseases in Latin American countries.^107^ The availability of a comprehensive frailty assessment that captures the clinical, cardiometabolic, and lifestyle dimensions described above is particularly relevant in Latin America, where rapid population aging, rising dementia prevalence, and limited access to standardized performance measures demand scalable and feasible tools for early risk detection and management. Leveraging routinely collected clinical data, such assessments can support large‐scale epidemiological studies and strengthen dementia risk stratification in resource‐limited settings.

Associations with GMV and functional connectivity were extensive, encompassing both disease‐specific regions and additional areas not typically emphasized in dementia research, suggesting a broader neural impact of frailty in Latin American older adults. In CU, frailty was associated with atrophy in brain regions primarily vulnerable to aging and dementia, such as frontal, parietal, and temporal regions.^108^, ^109^, ^110^ This was accompanied by associations across brain regions, such as the hippocampus, cerebellum, frontal gyrus, and precuneus, in line with findings employing mainly physically based frailty measures.^111^, ^112^ Our widespread pattern of associations across brain regions may be explained by the multidimensional nature of our frailty indicator, which integrates multiple health‐related domains beyond physical status, thereby capturing a broader spectrum of systemic vulnerability linked to brain structure in CU individuals. A pattern of association similar to CU was observed in AD and FTLD, primarily targeting dementia‐vulnerable regions specific to each syndrome, with predominant temporo‐parietal involvement in AD^113^ and frontal alterations in FTLD.^114^ Findings suggest a common pathway of frailty from CU to dementia that may subsequently diverge into syndrome‐specific atrophy patterns in AD and FTLD, consistent with current evidence linking frailty with dementia risk,^3^ incidence,^4^ brain atrophy,^39^, ^115^, ^116^ and neuropathology.^5^

Functional connectivity findings revealed main associations with the frailty network,^117^ characterized by increased connectivity in the visual system with enhanced caudate‐centered projections to visual and prefrontal regions that show a positive association with frailty. Like atrophy associations, a more widespread pattern with stronger caudate involvement was observed in CU, and occipital and frontal predominance in AD and FTLD, reflecting disruption of the frailty network aligned with dementia‐vulnerable regions specific to each syndrome. In line with previous functional connectivity‐based brain aging models in AD and FTLD,^70^ fronto‐temporo‐posterior disruptions were observed across groups, with a more marked aging‐related frontotemporal component in CU. This may suggest that frailty‐specific and aging‐related connectivity changes follow partially distinct but converging trajectories that interact in pathological aging, characterizing the compounded impact of frailty on neural connectivity integrity. Overall, these findings contribute to the global characterization of brain frailty^118^, ^119^ by revealing both shared and syndrome‐specific neural alterations across CU, AD, and FTLD, underscoring the relevance of frailty as a multidimensional construct that intersects with disease processes in dementia. In our Latin American sample, the widespread aging‐ and dementia‐sensitive patterns observed in our results may be partly explained by the extended multidomain nature of our frailty index – which integrates clinical, cognitive, psychological, functional, health behavior, and cardiometabolic dimensions – together with the pronounced multimorbidity and cardiometabolic profile that characterizes people with dementia in the region. These insights may help delineate the brain frailty phenotype across CU and dementia in Latin America, providing a framework to understand how multimorbidity and systemic risk factors shape neural vulnerability in this context.

Our study has several strengths. It provides the largest assessment of frailty and its multimodal neural correlates in dementia across Latin American countries, spanning CU, AD, and FTLD. This allowed us to examine frailty across the continuum from CU to major dementia syndromes and compare shared and syndrome‐specific neural signatures of frailty. It is also the first study to characterize frailty discrimination in FTLD and its multimodal neuroimaging profile, extending beyond findings from local or single‐center studies.^120^ A key strength lies in the use of a comprehensive frailty assessment derived from multidomain health data, which enhances ecological validity and supports applicability in real‐world clinical settings and is better suited to incorporate the multimorbidity profile highly relevant in Latin American populations. Methodologically, the study employed a robust approach that incorporated machine learning–based techniques to improve generalizability, alongside sensitivity analyses to account for potential circularity with clinical variables, as well as rigorous adjustments for scanner variability and image quality.

The study has important limitations. The health variables selected to calculate the frailty index were more oriented toward brain health than other physiological systems, which may have artificially inflated associations due to circularity and partial construct overlap with clinical dementia severity. We partially addressed this concern through sensitivity analyses excluding variables more closely linked to the dementia phenotype and adjusting neuroimaging analysis by global clinical status, which yielded consistent discriminability between aging and dementia and associations with brain volume and functional connectivity. However, because frailty is an aggregate construct, potential redundancy may arise from the overall architecture of the index rather than from individual components, and item‐level exclusions may therefore underestimate residual overlap with core aspects of dementia severity. Future studies should incorporate broader multisystem clinical measures to better disentangle global frailty from disease‐specific severity. The cross‐sectional design precludes causal inferences regarding the temporal relationship between frailty and brain changes. The lack of external comparisons with non‐Latin American cohorts constrain the generalizability of the findings. Laboratory‐based biomarkers of frailty, such as inflammatory or metabolic indicators, were not included, limiting the biological characterization of health deficits. Performance‐based measures such as grip strength and gait speed, widely used to complement frailty assessments with objective physical metrics, were absent. Finally, the omission of white matter analyses restricted direct comparison with previous studies and limited insights into the microstructural underpinnings of frailty‐related brain vulnerability.

In conclusion, this study provides a large‐scale, multimodal characterization of frailty across CU, AD, and FTLD in Latin America, demonstrating that frailty shows robust discriminative capacity between CU and dementia groups and captures a multidimensional signal of systemic vulnerability that aligns with both aging‐ and dementia‐sensitive neural alterations. The shared and syndrome‐specific structural and functional brain correlates contribute to the delineation of the global brain frailty phenotype and underscore the integrative role of frailty in dementia risk and progression. Regionally tailored frailty‐based strategies are required to support dementia prevention and care in Latin America, where multimorbidity and health disparities are highly prevalent. Many of the domains encompassed in our frailty index (hypertension, diabetes, cardiometabolic conditions, and lifestyle behaviors) overlap with leading modifiable risk factors for dementia,^121^ which accounts for a disproportionately high number of cases in the region compared to global estimates.^122^ Incorporating frailty assessment into dementia prevention and management agendas in the region may therefore provide a scalable, clinically feasible tool to identify at‐risk individuals, capture systemic contributors to brain vulnerability, and improve strategies to mitigate the growing burden of dementia in the region.

## CONFLICT OF INTEREST STATEMENT

The authors declare no conflicts of interest. Author disclosures are available in the Supporting Information.

## CONSENT STATEMENT

All human subjects provided written informed consent.

## Supporting information



Supporting Information

Supporting Information
