# Supplementary material for: Neural embedding of frailty in cognitively unimpaired aging and dementia across Latin America
Source: Alzheimers Dement. 2026 Apr 22;22(4):e71232. doi: 10.1002/alz.71232 (PMC13100973; doi:10.1002/alz.71232)
Supplement: Supplementary file 2 — Supporting Information [file ALZ-22-e71232-s002.docx]

**Supplementary information**

**Supplementary Material**

**Supplementary Material 1. Assessments employed for frailty index calculation**

*Cardiometabolic Assessments*

Participant heart health data was collected through blood pressure and resting heart rate measures. Systolic and diastolic blood pressure (mmHg) was submitted as a numerical value (systolic: min 30, max 210) (diastolic: min 20, max 120). Resting heart rate was measured in beats per minute, entered as a numerical value.

The Body Mass Index (BMI) is a scale widely used to measure obesity obtained by dividing weight (kg) by height ($m^{2}$). Participant data about the height and weight of each participant was collected to make these calculations. Height data was entered numerically from 0 to 250 cm, while weight was entered numerically from 0 to 250 kg.

*Visual and auditory health*

Visual and auditory health information was both listed directly from participant medical records and scored in visual and auditory health questionnaires. The visual assessment item comprised the following question: “without corrective lenses, is the subject’s vision functionally normal?” rated on a scale 0 to 2 (0 = yes, 1 = no but functionally normal with corrective lenses, 2 = no, and it is not improved by corrective lenses). The auditory assessment item comprised the following question: “without a hearing aid(s), is the subject’s hearing functionally normal? Rated on a scale from 0 to 2 (0 = yes, 1 = no but functionally normal with hearing aid(s), 2 = no, and it is not improved by hearing aid(s).

*Number of diagnoses and medications*

Information about the total number of diagnoses was collected by listing directly from participant medical records. History of hypertension, diabetes and dyslipidemia were considered independently[1, 2]. All medications were listed directly from participant medical records, including those pertaining to auditory and visual loss or correction.

*Health behavior*

Participant data concerning health behaviors was collected for smoking and substance abuse history. Smoking behavior including status, historical and habitual use were collected through self-report questionnaires. Smoking status items comprised 4 yes/no questions scaled 0 (no) to 1 (yes). These questions included “has the participant ever smoked?” and “does he/she still smoke?”, and “how many cigarettes per day on average did the participant smoke?”. Habitual questions concerning the frequency of daily smoking were entered as a free numerical response: “for how many years did the participant smoke or has the participant smoked?”, “how many cigarettes per day on average did the participant smoke over that time?”.

A fourth item comprised a binary score: yes = 1, no = 0 for the presence of alcohol or other substance abuse. Smoking behavior and substance abuse history are highly correlated with frailty index predictive outcomes[3, 4].

*Global clinical status*

The Clinical Dementia Rating Scale (CDR) is a widely established tool designed to assess cognitive functioning and determine the presence of dementia in aging adults[5]. The CDR evaluates scores across 6 domains: memory, orientation, judgment and problem solving, community affairs, home and hobbies, and personal care. These scores are rated on a five point scale from 0.0 to 3.0[6]. The CDR‐FTLD scale‐modified was employed in FTLD to evaluate the language and behavior as additional domains[7]. The CDR has exhibited high construct validity and reliability and has been widely used in Latin American participants[8-10].

*Neuropsychiatric symptoms*

The Neuropsychiatric Inventory (NPI-Q) was a tool developed to assess a wide array of behaviors and neuropsychiatric symptoms commonly present in dementia patients[11]. The NPI-Q evaluates 12 items: delusions, hallucinations, agitation/aggression, anxiety, depression/dysphoria, euphoria/elation, apathy/indifference, disinhibition, irritability/lability, aberrant motor behavior, nighttime behavioral disturbances, and appetite/eating abnormalities.

Each item captures both the severity and frequency of symptoms; severity is rated 1 (mild) through 3 (severe); frequency is rated 1 (occasionally) through 4 (very frequently) The sum of all rated items produces a single neuropsychiatric score, ranging from 0 to 36, with higher scores reflecting more severe and frequent symptom presentation. The NPI is validated, demonstrates adequate test-retest and interpreter reliability, and is widely used in Latin American participants[12, 13].

*Depression* The Geriatric Depression Scale Short Form (GDS‑SF) is a 15‑item self‑report screening tool derived from the original 30‑item GDS to assess depressive symptoms in older adults. Of the 15 yes/no questions, ten indicate the presence of depression when answered affirmatively, while the remaining five indicate depression when answered negatively. Designed to minimize fatigue and maintain simplicity for individuals with cognitive or physical limitations, the GDS‑SF can be completed in approximately five to seven minutes. Each item is scored 0 or 1 point, yielding a total score from 0 to 15; scores of 0–4 are considered within normal limits, 5–8 suggest mild depression, 9–11 indicate moderate depression, and 12–15 signify severe depressive symptoms. The GDS-SF demonstrates adequate test-retest and convergent reliability and is widely used in Latin American participants[14, 15].

*Functional ability*

The Pfeffer Functional Activities Questionnaire (PFAQ) assesses the functional abilities of older adults, focused on activities instrumental to everyday routines[16]. This tool measures the ability to perform tasks such as managing finances and medications, using the telephone, and meal preparation. Performance of each activity is scored on a scale from 0 (independent) to 3 (dependent), with the total score ranging from 0 (independent, no functional impairment) to 30 (dependent, functional impairment). This singular score provides an overall assessment of the individual's functional status. The PFAQ has demonstrated high reliability and is widely utilized in research with Latin American populations[17-19].

The Technology-Activities of Daily Living Questionnaire (T-ADLQ) is an informant-rated questionnaire that measures the ability to perform 33 activities of daily living encompassing the following areas of activities: self-care, household care, employment and recreation, shopping and money, travel, communication, and technology[20]. These are categorized into three domains stratified by hierarchical levels of complexity: basic, instrumental and advanced[21, 22]. Each activity was scored from 0 (no functional deficit) to 3 (functional deficit) summed in a final score. In addition, two non-numerical response options are included: 'Never Did', for activities not performed prior to dementia onset (e.g., employment), and 'Don’t Know', for situations in which the informant lacks sufficient information. These options allow for adjustment based on premorbid functioning and help mitigate potential cultural or gender-related biases. The T-ADLQ instrument was developed in Spanish and used widely in Latin American populations, demonstrating high validity and reliability[20, 23].

*Cognition*

The MMSE is a cognitive assessment tool for cognitive functions such as memory, arithmetic function, and orientation[24]. These functions are examined across five key domains: Orientation, registration, attention and calculation, recall, and language. The MMSE scores these functions from 0 to 30, with lower scores signifying greater cognitive impairment. Specifically, a score of 24 or higher reflects normative cognitive functioning, while scores below 24 indicate cognitive impairment. Scores of 19-23 suggest mild cognitive impairment, 10-18 indicate moderate impairment, and scores below 10 are consistent with severe impairment. The MMSE is a cognitive assessment tool used frequently in Latin American participants, exhibiting moderate to high reliability[17, 25, 26].

*Anxiety*

The GAD-7 is a widely utilized assessment tool developed to measure symptoms of generalized anxiety disorder (GAD) through a brief, 7 item self-report questionnaire[27]. Assessment items ask participants to recall the frequency of GAD symptoms experienced within the last two weeks. Responses to each item are rated 0 to 3 for frequency, 0 = “not at all”, 1 = “several days”, 2 = “over half the days” and 3 = “nearly every day”, out of a total maximum score of 21. Scores are interpreted as 0 – 4 “no anxiety appreciated” to 15 – 21 “severe anxiety symptoms appreciated”). The GAD-7 has been used frequently in research in Latin American populations with high criterion validity and test-retest reliability[27, 28].

**Supplementary Material 2. Variance inflation factor for estimation of collinearity between health variables.**

Variance inflation factors (VIFs) were calculated separately within each group to assess multicollinearity among the features included in the frailty index. VIFs were obtained by iteratively regressing each feature on the remaining predictors within group, allowing identification of domains exhibiting structural redundancy. In CU, VIFs were uniformly low, with the highest values observed for smoking-related variables (years smoking: VIF = 2.60; ever smoked: VIF = 2.29), followed by systolic blood pressure (VIF = 1.83), diastolic blood pressure (VIF = 1.65), depression (GDS-SF; VIF = 1.60), number of medications (VIF = 1.59), cigarettes per day (VIF = 1.56), anxiety (GAD-7; VIF = 1.56), and current smoking status (VIF = 1.49). In AD, higher VIFs were concentrated among functional and clinical severity measures, with the top values corresponding to instrumental activities of daily living (T-ADLQ instrumental; VIF = 4.13), global functionality (PFAQ; VIF = 2.79), CDR community affairs (VIF = 2.69), CDR home and hobbies (VIF = 2.36), CDR judgment and problem solving (VIF = 2.35), CDR orientation (VIF = 2.16), basic activities of daily living (T-ADLQ basic; VIF = 1.92), CDR memory (VIF = 1.80), CDR personal care (VIF = 1.78), and systolic blood pressure (VIF = 1.74), reflecting moderate collinearity among overlapping indicators of functional decline, but considerably below typical thresholds[29, 30]. In FTLD, the strongest multicollinearity was observed, again primarily within functional and severity-related domains, with the highest VIFs for instrumental functionality (T-ADLQ instrumental; VIF = 4.55), CDR community affairs (VIF = 4.04), global functionality (PFAQ; VIF = 3.66), CDR judgment and problem solving (VIF = 3.58), CDR home and hobbies (VIF = 3.21), CDR orientation (VIF = 2.88), CDR memory (VIF = 2.73), basic activities of daily living (T-ADLQ basic; VIF = 2.68), cognition (MMSE; VIF = 2.58), and CDR personal care (VIF = 2.42).

**Supplementary Figures**


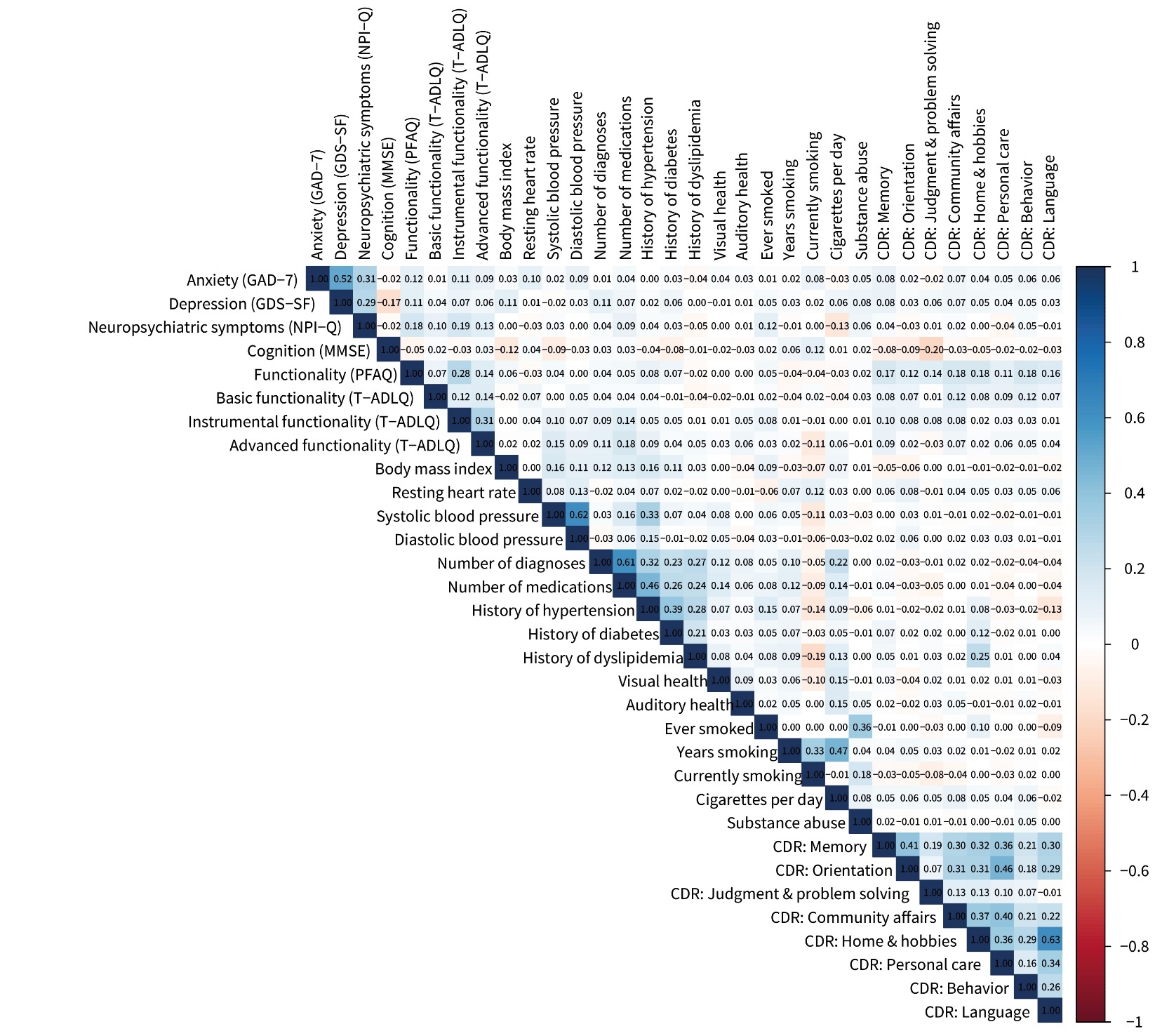


**Supplementary Figure 1**. Pairwise correlations among the variables included in the frailty index for CU. Continuous–continuous and continuous–binary variable pairs were assessed using Spearman correlations, while binary–binary pairs used tetrachoric correlations. Coefficients inside each cell and a color scale reflects correlation magnitude and direction.


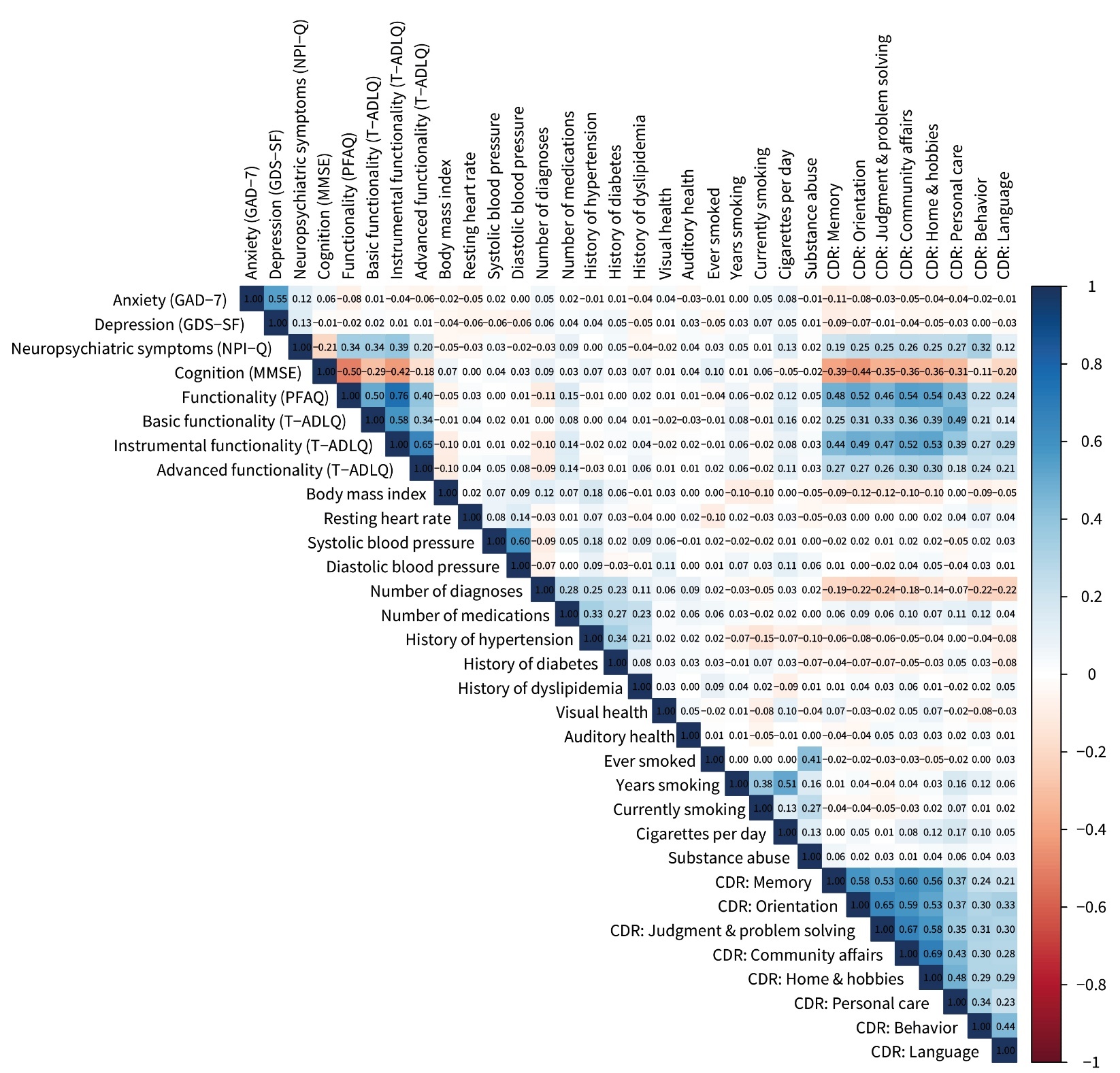


**Supplementary Figure 2**. Pairwise correlations among the variables included in the frailty index for AD. Continuous–continuous and continuous–binary variable pairs were assessed using Spearman correlations, while binary–binary pairs used tetrachoric correlations. Coefficients inside each cell and a color scale reflects correlation magnitude and direction.


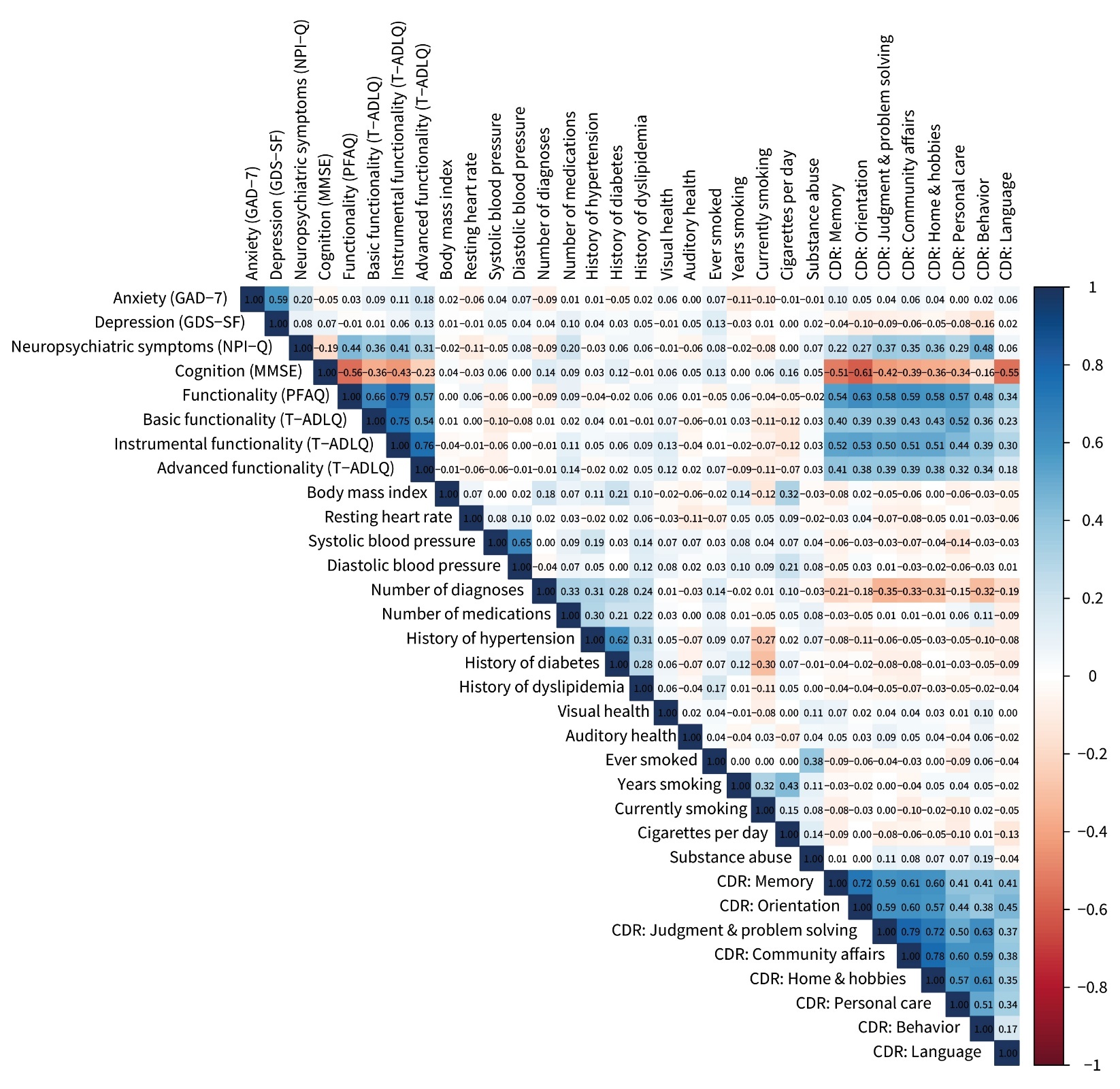


**Supplementary Figure 3**. Pairwise correlations among the variables included in the frailty index for FTLD. Continuous–continuous and continuous–binary variable pairs were assessed using Spearman correlations, while binary–binary pairs used tetrachoric correlations. Coefficients inside each cell and a color scale reflects correlation magnitude and direction.

| **Location** | **Scanner Model Tesla** | **Seq name** | **TR (ms)** | **TE (ms)** | **Flip angle (°)** | **Matrix dim** | **Voxel size (mm)** |
| --- | --- | --- | --- | --- | --- | --- | --- |
| Argentina site | 1.5T GE Signa HDxt | T1 3D FFE | 0.008824 | 0.003548 | 13 | 256x256x156 | 0.95x0.95x1 |
| Brazil site 1 | 3T Siemens Verio | SAG T1 3D | 1.8 | 0.00244 | 9 | 224x512x512 | 0.5x0.5x0.5 |
| Brazil site 2 | 3T Philips Achieva | T1 3D FFE | 0.0064768 | 0.002949 | 9 | 192x256x256 | 1x1x1 |
| Chile site 1 | 3T Philips Ingenia | T1 3D FFE | 0.0077732 | 0.003553 | 8 | 355x576x576 | 0.5x0.5x0.5 |
| Chile site 2 | 3T Siemens MAGNETOM Lumina | MP RAGE | 1.8 | 0.00213 | 8 | 192x256x256 | 1x1x1 |
| Chile site 3 | 3T Philips Ingenia | T1 3D FFE | 0.0078661 | 0.003601 | 8 | 355x576x576 | 0.5x0.5x0.5 |
| Chile site 4 | 3T Siemens MAGNETOM Lumina | T1 3D FFE | 1.8 | 0.00213 | 8 | 192x256x256 | 1x1x1 |
| Colombia site 1 | 3T Siemens Skyra | MP RAGE | 2.3 | 0.00225 | 8 | 192x256x256 | 0.95x0.95x0.95 |
| Colombia site 2 | 3T Philips Ingenia Elition X | TFE | 0.0080664 | 0.003694 | 8 | 200x256x256 | 1x1x1 |
| Colombia site 3 | 3T Philips Achieva | SAG T1 3D | 0.0076217 | 0.003747 | 8 | 180x256x256 | 1x1x1 |
| Mexico site | 3T Siemens Biograph mMR | MP RAGE | 2.3 | 0.00289 | 9 | 160x256x256 | 1x1x1 |
| Peru site 1 | 3T Siemens Skyra | MP RAGE | 2.3 | 0.00227 | 8 | 176x256x256 | 1x.977x.977 |
| Peru site 2 | 3T Siemens Spectra | MP RAGE | 1.9 | 0.00242 | 9 | 160x256x256 | 1x.977x.977 |

**Supplementary Table 1.** Structural MRI acquisition parameters per site.

| **Location** | **Scanner Model Tesla** | **TR (ms)** | **TE (ms)** | **Voxel size (mm)** | **N° vol** | **Matrix dimension** | **Flip angle (º)** | **N° slices** |
| --- | --- | --- | --- | --- | --- | --- | --- | --- |
| Argentina site | 1.5T GE Signa HDxt | 2.5 | 0.03 | 3.8x3.8x5 | 120 | 64x33 | 50 | 33 |
| Brazil site 1 | 3T Siemens Verio | 3 | 0.03 | 3.4x3.4x5 | 80 | 64x30 | 90 | 36 |
| Brazil site 2 | 3T Philips Achieva | 2.2 | 0.028001 | 3.3x3.3x3.3 | 275 | 64x44 | 80 | 44 |
| Chile site 1 | 3T Philips Ingenia | 4.9715 | 0.030001 | 2.5x2.5x2.8 | 121 | 96x45 | 82 | 45 |
| Chile site 2 | 3T Siemens MAGNETOM Lumina | 2.5 | 0.03 | 2.5x2.5x2.5 | 235 | 94x66 | 90 | 66 |
| Chile site 3 | 3T Philips Ingenia | 4.97133 | 0.03 | 2.5x2.5x2.8 | 121 | 96x45 | 82 | 45 |
| Chile site 4 | 3T Siemens MAGNETOM Lumina | 2.5 | 0.03 | 2.5x2.5x2.5 | 235 | 94x66 | 90 | 66 |
| Colombia site 1 | 3T Siemens Skyra | 3 | 0.03 | 3x3x3 | 200 | 70x45 | 80 | 39 |
| Colombia site 2 | 3T Philips Ingenia Elition X | 2.5 | 0.03 | 3x3x3 | 240 | 80x60 | 90 | 60 |
| Colombia site 3 | 3T Philips Achieva | 3 | 0.03 | 3x3x3 | 197 | 64x48 | 90 | 50 |
| Mexico site | 3T Siemens Biograph mMR | 3.5 | 0.032 | 2.2x2.2x2.7 | 120 | 94x66 | 45 | 66 |

**Supplementary Table 2.** Resting-state fMRI acquisition parameters per site.

| **Region** | **CU** | **AD** | ***t*** | ***P*_FDR_** |
| --- | --- | --- | --- | --- |
| Precentral L | -1.205 (1.023) | -2.265 (0.839) | 25.324 | < 0.001 |
| Precentral R | -1.159 (1.244) | -2.744 (0.789) | 34.037 | < 0.001 |
| Frontal Sup L | -1.485 (0.99) | -1.81 (0.866) | 7.833 | < 0.001 |
| Frontal Sup Orb L | -1.414 (0.927) | -2.58 (0.89) | 28.694 | < 0.001 |
| Frontal Mid L | -1.77 (0.901) | -2.225 (0.798) | 11.957 | < 0.001 |
| Frontal Mid Orb L | -1.098 (0.943) | -1.892 (0.859) | 19.685 | < 0.001 |
| Frontal Inf Tri L | -1.437 (0.864) | -1.936 (0.833) | 13.135 | < 0.001 |
| Frontal Inf Orb L | -1.767 (0.951) | -1.603 (0.851) | -4.056 | < 0.001 |
| Frontal Inf Orb R | -1.624 (1.062) | -2.083 (0.834) | 10.750 | < 0.001 |
| Rolandic Oper L | -0.903 (0.894) | -2.638 (0.843) | 44.668 | < 0.001 |
| Olfactory L | -1.251 (0.874) | -2.812 (0.861) | 40.219 | < 0.001 |
| Frontal Sup Med L | -1.809 (1.066) | -2.182 (0.799) | 8.870 | < 0.001 |
| Frontal Med Orb L | -1.376 (0.922) | -1.722 (0.772) | 9.103 | < 0.001 |
| Rectus L | -1.234 (0.905) | -2.108 (0.816) | 22.677 | < 0.001 |
| Rectus R | -1.126 (0.915) | -3.041 (0.874) | 47.828 | < 0.001 |
| Insula L | -1.578 (0.861) | -2.394 (0.807) | 21.883 | < 0.001 |
| Insula R | -2.443 (0.881) | -3.191 (0.839) | 19.436 | < 0.001 |
| Cingulum Mid L | -1.589 (1.025) | -2.396 (0.846) | 19.201 | < 0.001 |
| Hippocampus L | -1.604 (0.898) | -3.157 (0.926) | 38.073 | < 0.001 |
| Hippocampus R | -1.494 (0.83) | -3.479 (0.928) | 50.381 | < 0.001 |
| ParaHippocampal R | -1.409 (0.92) | -2.918 (0.913) | 36.839 | < 0.001 |
| Amygdala L | -1.766 (0.882) | -2.535 (0.898) | 19.310 | < 0.001 |
| Amygdala R | -2.209 (0.88) | -3.144 (0.918) | 23.231 | < 0.001 |
| Calcarine L | -1.518 (0.98) | -1.749 (0.831) | 5.682 | < 0.001 |
| Calcarine R | -1.517 (0.981) | -2.457 (0.864) | 22.736 | < 0.001 |
| Cuneus R | -1.456 (0.89) | -2.637 (0.91) | 29.347 | < 0.001 |
| Lingual L | -1.756 (0.949) | -2.016 (0.872) | 6.383 | < 0.001 |
| Lingual R | -1.573 (0.92) | -2.549 (0.893) | 24.067 | < 0.001 |
| Occipital Sup L | -1.195 (0.964) | -2.252 (0.849) | 26.028 | < 0.001 |
| Occipital Sup R | -1.316 (0.96) | -3.217 (0.898) | 45.721 | < 0.001 |
| Occipital Mid L | -1.717 (0.936) | -2.374 (0.862) | 16.327 | < 0.001 |
| Occipital Mid R | -1.697 (0.999) | -3.065 (0.934) | 31.627 | < 0.001 |
| Occipital Inf L | -1.111 (0.925) | -2.402 (0.841) | 32.635 | < 0.001 |
| Occipital Inf R | -0.992 (0.885) | -2.675 (0.928) | 41.503 | < 0.001 |
| Fusiform L | -1.564 (1.005) | -2.984 (0.836) | 34.363 | < 0.001 |
| Fusiform R | -1.717 (1.022) | -3.19 (0.84) | 35.212 | < 0.001 |
| Postcentral L | -1.426 (0.881) | -2.416 (0.858) | 25.461 | < 0.001 |
| Parietal Inf L | -1.972 (0.898) | -2.37 (0.849) | 10.178 | < 0.001 |
| Parietal Inf R | -1.237 (0.87) | -1.946 (0.938) | 17.509 | < 0.001 |
| SupraMarginal L | -1.128 (0.854) | -2.43 (0.88) | 33.588 | < 0.001 |
| SupraMarginal R | -2.191 (0.87) | -2.544 (0.939) | 8.710 | < 0.001 |
| Angular R | -1.35 (0.924) | -2.52 (0.922) | 28.331 | < 0.001 |
| Precuneus L | -0.909 (0.954) | -1.924 (0.914) | 24.280 | < 0.001 |
| Caudate L | -1.222 (1.038) | -1.427 (1.047) | 4.401 | < 0.001 |
| Putamen L | -1.096 (0.957) | -1.605 (0.857) | 12.523 | < 0.001 |
| Pallidum L | 1.128 (1.041) | 0.173 (0.888) | 22.088 | < 0.001 |
| Thalamus L | -1.095 (0.953) | -1.871 (0.845) | 19.257 | < 0.001 |
| Thalamus R | -1.16 (0.94) | -2.176 (0.784) | 26.241 | < 0.001 |
| Heschl L | -1.236 (0.834) | -2.694 (0.895) | 37.699 | < 0.001 |
| Heschl R | -1.636 (0.922) | -1.869 (0.884) | 5.773 | < 0.001 |
| Temporal Sup L | -1.355 (0.916) | -3.124 (0.848) | 44.824 | < 0.001 |
| Temporal Sup R | -2.238 (0.975) | -2.958 (0.827) | 17.812 | < 0.001 |
| Temporal Pole Sup L | -1.9 (0.919) | -2.086 (0.855) | 4.679 | < 0.001 |
| Temporal Pole Sup R | -1.344 (0.941) | -1.898 (0.83) | 13.963 | < 0.001 |
| Temporal Mid L | -2.4 (1.029) | -3.053 (0.904) | 15.073 | < 0.001 |
| Temporal Mid R | -1.681 (0.99) | -2.908 (0.833) | 29.984 | < 0.001 |
| Temporal Pol Mid L | -1.345 (0.983) | -2.163 (0.841) | 20.011 | < 0.001 |
| Temporal Pol Mid R | -1.68 (0.934) | -2.256 (0.849) | 14.439 | < 0.001 |
| Temporal Inf L | -1.272 (0.965) | -2.863 (0.894) | 38.221 | < 0.001 |
| Temporal Inf R | -1.719 (0.91) | -3.443 (0.836) | 44.115 | < 0.001 |
| Cerebelum Crus1 L | -1.373 (0.915) | -0.707 (0.929) | -16.141 | < 0.001 |
| Cerebelum Crus1 R | -1.672 (0.963) | -0.798 (0.874) | -21.254 | < 0.001 |
| Cerebelum Crus2 L | -1.073 (0.87) | -1.79 (0.855) | 18.587 | < 0.001 |
| Cerebelum 3 L | -1.12 (0.886) | -0.895 (0.848) | -5.818 | < 0.001 |
| Cerebelum 3 R | -1.444 (0.827) | -0.305 (0.807) | -31.151 | < 0.001 |
| Cerebelum 4 5 L | -1.637 (0.906) | -0.343 (0.82) | -33.499 | < 0.001 |
| Cerebelum 4 5 R | -2.068 (0.938) | -0.837 (0.815) | -31.317 | < 0.001 |
| Cerebelum 6 L | -2.015 (0.935) | -1.043 (0.801) | -24.955 | < 0.001 |
| Cerebelum 6 R | -2.048 (0.987) | -1.129 (0.866) | -22.116 | < 0.001 |
| Cerebelum 7b L | -1.136 (0.852) | -1.744 (0.797) | 16.484 | < 0.001 |
| Cerebelum 8 L | -1.119 (0.856) | -1.207 (0.824) | 2.338 | 0.021 |
| Cerebelum 10 L | -1.209 (0.896) | -0.279 (0.898) | -23.186 | < 0.001 |
| Cerebelum 10 R | -1.33 (0.829) | -0.579 (0.877) | -19.689 | < 0.001 |
| Vermis 3 | -0.912 (0.828) | -0.375 (0.791) | -14.844 | < 0.001 |
| Vermis 4 5 | -1.275 (0.836) | -0.049 (0.869) | -32.151 | < 0.001 |
| Vermis 6 | -1.156 (0.873) | -0.374 (0.865) | -20.118 | < 0.001 |
| Vermis 7 | -1.077 (0.891) | -0.342 (0.816) | -19.243 | < 0.001 |
| Vermis 8 | -1.005 (0.836) | -0.533 (0.793) | -12.953 | < 0.001 |
| Vermis 9 | -0.964 (0.861) | -0.552 (0.78) | -11.212 | < 0.001 |
| Frontal Sup R | -0.914 (1.363) | -2.468 (0.786) | 31.230 | < 0.001 |
| Frontal Mid R | -0.851 (1.317) | -2.03 (0.748) | 24.605 | < 0.001 |
| Frontal Inf Oper L | -0.908 (1.018) | -1.839 (0.86) | 22.069 | < 0.001 |
| Frontal Inf Oper R | -0.492 (1.078) | -2.396 (0.897) | 42.940 | < 0.001 |
| Frontal Inf Tri R | -0.547 (0.944) | -2.044 (0.849) | 37.300 | < 0.001 |
| Rolandic Oper R | -0.683 (0.959) | -1.888 (0.756) | 31.213 | < 0.001 |
| Supp Motor Area R | -0.758 (1.31) | -2.035 (0.859) | 25.759 | < 0.001 |
| Olfactory R | -0.739 (0.927) | -1.82 (0.854) | 27.137 | < 0.001 |
| Frontal Sup Med R | -0.69 (1.218) | -1.579 (0.78) | 19.430 | < 0.001 |
| Frontal Med Orb R | -0.921 (0.962) | -1.733 (0.866) | 19.848 | < 0.001 |
| Cingulum Ant L | -1.051 (1.106) | -1.78 (0.761) | 17.182 | < 0.001 |
| Cingulum Ant R | -0.73 (1.199) | -0.982 (0.723) | 5.701 | < 0.001 |
| Cingulum Mid R | -1.315 (1.418) | -2.871 (0.867) | 29.603 | < 0.001 |
| Cingulum Post L | 0.153 (0.887) | -1.453 (0.878) | 40.673 | < 0.001 |
| Cingulum Post R | 0.52 (0.983) | -1.572 (0.83) | 51.421 | < 0.001 |
| ParaHippocampal L | -0.729 (0.965) | -2.665 (0.883) | 46.779 | < 0.001 |
| Postcentral R | -0.788 (0.987) | -1.893 (0.827) | 27.126 | < 0.001 |
| Parietal Sup L | -0.775 (0.913) | -1.729 (0.844) | 24.273 | < 0.001 |
| Parietal Sup R | -0.46 (0.901) | -2.753 (0.921) | 56.284 | < 0.001 |
| Angular L | -0.879 (0.937) | -2.371 (0.903) | 36.247 | < 0.001 |
| Precuneus R | -0.425 (1.023) | -2.188 (0.883) | 41.237 | < 0.001 |

**Supplementary Table 3**. Between-group comparison on regional frailty-related GMV effects between CU and AD. Mean (SD) t-values of the frailty–GMV association estimated separately in cognitively unimpaired (CU) and Alzheimer’s disease (AD) groups using repeated subsampling (n = 1000) and models adjusted for scanner effects.

| **Region** | **CU** | **AD** | ***t*** | ***P*_FDR_** |
| --- | --- | --- | --- | --- |
| Precentral L | -1.205 (1.023) | -2.43 (0.685) | 31.471 | < 0.001 |
| Precentral R | -1.159 (1.244) | -0.985 (0.623) | -3.944 | < 0.001 |
| Frontal Sup L | -1.485 (0.99) | -3.609 (0.716) | 54.984 | < 0.001 |
| Frontal Sup Orb L | -1.414 (0.927) | -2.891 (0.729) | 39.601 | < 0.001 |
| Frontal Sup Orb R | -1.587 (0.927) | -0.857 (0.834) | -18.503 | < 0.001 |
| Frontal Mid L | -1.77 (0.901) | -3.749 (0.722) | 54.222 | < 0.001 |
| Frontal Mid Orb L | -1.098 (0.943) | -2.811 (0.756) | 44.814 | < 0.001 |
| Frontal Mid Orb R | -1.548 (0.997) | -1.821 (0.812) | 6.721 | < 0.001 |
| Frontal Inf Tri L | -1.437 (0.864) | -3.403 (0.756) | 54.155 | < 0.001 |
| Frontal Inf Orb L | -1.767 (0.951) | -3.626 (0.738) | 48.832 | < 0.001 |
| Frontal Inf Orb R | -1.624 (1.062) | -2.273 (0.75) | 15.783 | < 0.001 |
| Rolandic Oper L | -0.903 (0.894) | -2.832 (0.678) | 54.374 | < 0.001 |
| Supp Motor Area L | -1.694 (0.969) | -2.519 (0.699) | 21.836 | < 0.001 |
| Olfactory L | -1.251 (0.874) | -1.879 (0.661) | 18.107 | < 0.001 |
| Frontal Sup Med L | -1.809 (1.066) | -3.43 (0.742) | 39.467 | < 0.001 |
| Frontal Med Orb L | -1.376 (0.922) | -2.065 (0.69) | 18.917 | < 0.001 |
| Rectus L | -1.234 (0.905) | -2.171 (0.615) | 27.054 | < 0.001 |
| Rectus R | -1.126 (0.915) | -2.207 (0.761) | 28.707 | < 0.001 |
| Insula L | -1.578 (0.861) | -2.091 (0.707) | 14.569 | < 0.001 |
| Insula R | -2.443 (0.881) | -2.563 (0.755) | 3.268 | 0.001 |
| Cingulum Mid L | -1.589 (1.025) | -2.937 (0.61) | 35.726 | < 0.001 |
| Hippocampus L | -1.604 (0.898) | -1.321 (0.676) | -7.983 | < 0.001 |
| Hippocampus R | -1.494 (0.83) | -1.056 (0.736) | -12.501 | < 0.001 |
| ParaHippocampal R | -1.409 (0.92) | -0.448 (0.707) | -26.203 | < 0.001 |
| Amygdala L | -1.766 (0.882) | 0.007 (0.691) | -50.015 | < 0.001 |
| Amygdala R | -2.209 (0.88) | -0.557 (0.667) | -47.302 | < 0.001 |
| Calcarine L | -1.518 (0.98) | -0.509 (0.57) | -28.126 | < 0.001 |
| Calcarine R | -1.517 (0.981) | 0.132 (0.591) | -45.545 | < 0.001 |
| Cuneus L | -1.461 (0.939) | -1.191 (0.62) | -7.602 | < 0.001 |
| Cuneus R | -1.456 (0.89) | -0.399 (0.63) | -30.647 | < 0.001 |
| Lingual L | -1.756 (0.949) | -0.34 (0.564) | -40.559 | < 0.001 |
| Lingual R | -1.573 (0.92) | -0.02 (0.562) | -45.534 | < 0.001 |
| Occipital Sup R | -1.316 (0.96) | -0.894 (0.698) | -11.237 | < 0.001 |
| Occipital Mid L | -1.717 (0.936) | -1.372 (0.805) | -8.826 | < 0.001 |
| Occipital Inf L | -1.111 (0.925) | -1.439 (0.726) | 8.815 | < 0.001 |
| Occipital Inf R | -0.992 (0.885) | -1.996 (0.621) | 29.372 | < 0.001 |
| Fusiform L | -1.564 (1.005) | -1.072 (0.652) | -12.999 | < 0.001 |
| Fusiform R | -1.717 (1.022) | -0.627 (0.677) | -28.124 | < 0.001 |
| Postcentral L | -1.426 (0.881) | -1.551 (0.601) | 3.711 | < 0.001 |
| Parietal Inf R | -1.237 (0.87) | -1.138 (0.652) | -2.878 | 0.004 |
| SupraMarginal L | -1.128 (0.854) | -3.014 (0.688) | 54.363 | < 0.001 |
| SupraMarginal R | -2.191 (0.87) | -1.505 (0.632) | -20.166 | < 0.001 |
| Angular R | -1.35 (0.924) | -0.962 (0.653) | -10.850 | < 0.001 |
| Precuneus L | -0.909 (0.954) | -1.946 (0.62) | 28.819 | < 0.001 |
| Caudate L | -1.222 (1.038) | -2.857 (0.802) | 39.417 | < 0.001 |
| Putamen L | -1.096 (0.957) | -1.906 (0.748) | 21.063 | < 0.001 |
| Putamen R | -1.305 (0.949) | -1.144 (0.802) | -4.109 | < 0.001 |
| Pallidum L | 1.128 (1.041) | -0.362 (0.657) | 38.307 | < 0.001 |
| Thalamus L | -1.095 (0.953) | -1.256 (0.634) | 4.445 | < 0.001 |
| Thalamus R | -1.16 (0.94) | -0.666 (0.616) | -13.893 | < 0.001 |
| Heschl L | -1.236 (0.834) | -2.222 (0.708) | 28.507 | < 0.001 |
| Heschl R | -1.636 (0.922) | -1.803 (0.665) | 4.662 | < 0.001 |
| Temporal Sup L | -1.355 (0.916) | -2.479 (0.72) | 30.524 | < 0.001 |
| Temporal Sup R | -2.238 (0.975) | -1.827 (0.665) | -11.024 | < 0.001 |
| Temporal Pole Sup L | -1.9 (0.919) | -0.703 (0.653) | -33.586 | < 0.001 |
| Temporal Pole Sup R | -1.344 (0.941) | -0.3 (0.66) | -28.726 | < 0.001 |
| Temporal Mid L | -2.4 (1.029) | -2.238 (0.622) | -4.278 | < 0.001 |
| Temporal Pol Mid L | -1.345 (0.983) | 0.016 (0.719) | -35.338 | < 0.001 |
| Temporal Pol Mid R | -1.68 (0.934) | -0.282 (0.697) | -37.927 | < 0.001 |
| Temporal Inf L | -1.272 (0.965) | -1.138 (0.627) | -3.704 | < 0.001 |
| Temporal Inf R | -1.719 (0.91) | -0.961 (0.696) | -20.921 | < 0.001 |
| Cerebelum Crus1 L | -1.373 (0.915) | 0.13 (0.615) | -43.120 | < 0.001 |
| Cerebelum Crus1 R | -1.672 (0.963) | -0.145 (0.615) | -42.279 | < 0.001 |
| Cerebelum Crus2 L | -1.073 (0.87) | 0.128 (0.717) | -33.710 | < 0.001 |
| Cerebelum Crus2 R | -1.422 (0.882) | 0.007 (0.684) | -40.459 | < 0.001 |
| Cerebelum 3 L | -1.12 (0.886) | -0.062 (0.553) | -32.062 | < 0.001 |
| Cerebelum 3 R | -1.444 (0.827) | 0.08 (0.68) | -44.999 | < 0.001 |
| Cerebelum 4 5 L | -1.637 (0.906) | 0.036 (0.599) | -48.723 | < 0.001 |
| Cerebelum 4 5 R | -2.068 (0.938) | -0.134 (0.589) | -55.224 | < 0.001 |
| Cerebelum 6 L | -2.015 (0.935) | -0.195 (0.616) | -51.402 | < 0.001 |
| Cerebelum 6 R | -2.048 (0.987) | -0.469 (0.613) | -42.955 | < 0.001 |
| Cerebelum 7b L | -1.136 (0.852) | -0.063 (0.721) | -30.418 | < 0.001 |
| Cerebelum 7b R | -1.577 (0.822) | -0.246 (0.712) | -38.704 | < 0.001 |
| Cerebelum 8 L | -1.119 (0.856) | -0.496 (0.658) | -18.236 | < 0.001 |
| Cerebelum 8 R | -1.309 (0.902) | -0.383 (0.69) | -25.799 | < 0.001 |
| Cerebelum 10 L | -1.209 (0.896) | 0.439 (0.598) | -48.361 | < 0.001 |
| Cerebelum 10 R | -1.33 (0.829) | 1.039 (0.665) | -70.508 | < 0.001 |
| Vermis 3 | -0.912 (0.828) | 0.528 (0.582) | -45.008 | < 0.001 |
| Vermis 4 5 | -1.275 (0.836) | -0.228 (0.636) | -31.510 | < 0.001 |
| Vermis 6 | -1.156 (0.873) | 0.35 (0.651) | -43.732 | < 0.001 |
| Vermis 7 | -1.077 (0.891) | 0.6 (0.686) | -47.165 | < 0.001 |
| Vermis 8 | -1.005 (0.836) | -0.308 (0.675) | -20.534 | < 0.001 |
| Vermis 9 | -0.964 (0.861) | 0.02 (0.62) | -29.329 | < 0.001 |
| Frontal Sup R | -0.914 (1.363) | -2.595 (0.685) | 34.842 | < 0.001 |
| Frontal Mid R | -0.851 (1.317) | -2.303 (0.752) | 30.261 | < 0.001 |
| Frontal Inf Oper L | -0.908 (1.018) | -3.275 (0.807) | 57.590 | < 0.001 |
| Frontal Inf Tri R | -0.547 (0.944) | -2.193 (0.724) | 43.774 | < 0.001 |
| Rolandic Oper R | -0.683 (0.959) | -2.587 (0.641) | 52.213 | < 0.001 |
| Supp Motor Area R | -0.758 (1.31) | -2.157 (0.704) | 29.733 | < 0.001 |
| Frontal Sup Med R | -0.69 (1.218) | -2.001 (0.75) | 28.985 | < 0.001 |
| Frontal Med Orb R | -0.921 (0.962) | -1.841 (0.772) | 23.574 | < 0.001 |
| Cingulum Ant L | -1.051 (1.106) | -2.532 (0.685) | 36.004 | < 0.001 |
| Cingulum Mid R | -1.315 (1.418) | -2.254 (0.68) | 18.881 | < 0.001 |
| Cingulum Post L | 0.153 (0.887) | -2.48 (0.558) | 79.460 | < 0.001 |
| Parietal Sup R | -0.46 (0.901) | -1.609 (0.748) | 31.034 | < 0.001 |
| Angular L | -0.879 (0.937) | -2.113 (0.584) | 35.319 | < 0.001 |
| Precuneus R | -0.425 (1.023) | -1.838 (0.607) | 37.556 | < 0.001 |
| Caudate R | -0.895 (1.228) | -2.243 (0.825) | 28.811 | < 0.001 |

**Supplementary Table 4**. Between-group comparison on regional frailty-related GMV effects between CU and FTLD. Mean (SD) t-values of the frailty–GMV association estimated separately in cognitively unimpaired (CU) and frontotemporal lobar degeneration (FTLD) groups using repeated subsampling (n = 1000) and models adjusted for scanner effects.

| **Region** | **AD** | **FTLD** | ***t*** | ***P*_FDR_** |
| --- | --- | --- | --- | --- |
| Precentral L | -2.265 (0.839) | -2.43 (0.685) | 4.829 | < 0.001 |
| Precentral R | -2.744 (0.789) | -0.985 (0.623) | -55.314 | < 0.001 |
| Frontal Sup L | -1.81 (0.866) | -3.609 (0.716) | 50.585 | < 0.001 |
| Frontal Sup Orb L | -2.58 (0.89) | -2.891 (0.729) | 8.553 | < 0.001 |
| Frontal Sup Orb R | -1.635 (0.793) | -0.857 (0.834) | -21.389 | < 0.001 |
| Frontal Mid L | -2.225 (0.798) | -3.749 (0.722) | 44.793 | < 0.001 |
| Frontal Mid Orb L | -1.892 (0.859) | -2.811 (0.756) | 25.403 | < 0.001 |
| Frontal Mid Orb R | -1.597 (0.84) | -1.821 (0.812) | 6.067 | < 0.001 |
| Frontal Inf Tri L | -1.936 (0.833) | -3.403 (0.756) | 41.259 | < 0.001 |
| Frontal Inf Orb L | -1.603 (0.851) | -3.626 (0.738) | 56.808 | < 0.001 |
| Frontal Inf Orb R | -2.083 (0.834) | -2.273 (0.75) | 5.354 | < 0.001 |
| Rolandic Oper L | -2.638 (0.843) | -2.832 (0.678) | 5.678 | < 0.001 |
| Supp Motor Area L | -1.735 (0.748) | -2.519 (0.699) | 24.184 | < 0.001 |
| Olfactory L | -2.812 (0.861) | -1.879 (0.661) | -27.179 | < 0.001 |
| Frontal Sup Med L | -2.182 (0.799) | -3.43 (0.742) | 36.178 | < 0.001 |
| Frontal Med Orb L | -1.722 (0.772) | -2.065 (0.69) | 10.460 | < 0.001 |
| Rectus R | -3.041 (0.874) | -2.207 (0.761) | -22.761 | < 0.001 |
| Insula L | -2.394 (0.807) | -2.091 (0.707) | -8.942 | < 0.001 |
| Insula R | -3.191 (0.839) | -2.563 (0.755) | -17.588 | < 0.001 |
| Cingulum Mid L | -2.396 (0.846) | -2.937 (0.61) | 16.399 | < 0.001 |
| Hippocampus L | -3.157 (0.926) | -1.321 (0.676) | -50.660 | < 0.001 |
| Hippocampus R | -3.479 (0.928) | -1.056 (0.736) | -64.663 | < 0.001 |
| ParaHippocampal R | -2.918 (0.913) | -0.448 (0.707) | -67.680 | < 0.001 |
| Amygdala L | -2.535 (0.898) | 0.007 (0.691) | -70.927 | < 0.001 |
| Amygdala R | -3.144 (0.918) | -0.557 (0.667) | -72.086 | < 0.001 |
| Calcarine L | -1.749 (0.831) | -0.509 (0.57) | -38.897 | < 0.001 |
| Calcarine R | -2.457 (0.864) | 0.132 (0.591) | -78.221 | < 0.001 |
| Cuneus L | -1.513 (0.867) | -1.191 (0.62) | -9.549 | < 0.001 |
| Cuneus R | -2.637 (0.91) | -0.399 (0.63) | -63.960 | < 0.001 |
| Lingual L | -2.016 (0.872) | -0.34 (0.564) | -51.024 | < 0.001 |
| Lingual R | -2.549 (0.893) | -0.02 (0.562) | -75.796 | < 0.001 |
| Occipital Sup L | -2.252 (0.849) | -1.118 (0.758) | -31.526 | < 0.001 |
| Occipital Sup R | -3.217 (0.898) | -0.894 (0.698) | -64.562 | < 0.001 |
| Occipital Mid L | -2.374 (0.862) | -1.372 (0.805) | -26.860 | < 0.001 |
| Occipital Mid R | -3.065 (0.934) | -1.736 (0.741) | -35.243 | < 0.001 |
| Occipital Inf L | -2.402 (0.841) | -1.439 (0.726) | -27.405 | < 0.001 |
| Occipital Inf R | -2.675 (0.928) | -1.996 (0.621) | -19.215 | < 0.001 |
| Fusiform L | -2.984 (0.836) | -1.072 (0.652) | -57.044 | < 0.001 |
| Fusiform R | -3.19 (0.84) | -0.627 (0.677) | -75.153 | < 0.001 |
| Postcentral L | -2.416 (0.858) | -1.551 (0.601) | -26.103 | < 0.001 |
| Parietal Inf L | -2.37 (0.849) | -2.011 (0.712) | -10.244 | < 0.001 |
| Parietal Inf R | -1.946 (0.938) | -1.138 (0.652) | -22.355 | < 0.001 |
| SupraMarginal L | -2.43 (0.88) | -3.014 (0.688) | 16.520 | < 0.001 |
| SupraMarginal R | -2.544 (0.939) | -1.505 (0.632) | -28.994 | < 0.001 |
| Angular R | -2.52 (0.922) | -0.962 (0.653) | -43.595 | < 0.001 |
| Caudate L | -1.427 (1.047) | -2.857 (0.802) | 34.288 | < 0.001 |
| Putamen L | -1.605 (0.857) | -1.906 (0.748) | 8.348 | < 0.001 |
| Putamen R | -1.317 (0.817) | -1.144 (0.802) | -4.781 | < 0.001 |
| Thalamus L | -1.871 (0.845) | -1.256 (0.634) | -18.406 | < 0.001 |
| Thalamus R | -2.176 (0.784) | -0.666 (0.616) | -47.879 | < 0.001 |
| Heschl L | -2.694 (0.895) | -2.222 (0.708) | -13.076 | < 0.001 |
| Temporal Sup L | -3.124 (0.848) | -2.479 (0.72) | -18.338 | < 0.001 |
| Temporal Sup R | -2.958 (0.827) | -1.827 (0.665) | -33.728 | < 0.001 |
| Temporal Pole Sup L | -2.086 (0.855) | -0.703 (0.653) | -40.658 | < 0.001 |
| Temporal Pole Sup R | -1.898 (0.83) | -0.3 (0.66) | -47.639 | < 0.001 |
| Temporal Mid L | -3.053 (0.904) | -2.238 (0.622) | -23.497 | < 0.001 |
| Temporal Mid R | -2.908 (0.833) | -1.694 (0.684) | -35.652 | < 0.001 |
| Temporal Pol Mid L | -2.163 (0.841) | 0.016 (0.719) | -62.284 | < 0.001 |
| Temporal Pol Mid R | -2.256 (0.849) | -0.282 (0.697) | -56.837 | < 0.001 |
| Temporal Inf L | -2.863 (0.894) | -1.138 (0.627) | -49.940 | < 0.001 |
| Temporal Inf R | -3.443 (0.836) | -0.961 (0.696) | -72.170 | < 0.001 |
| Cerebelum Crus2 L | -1.79 (0.855) | 0.128 (0.717) | -54.383 | < 0.001 |
| Cerebelum Crus2 R | -1.437 (0.906) | 0.007 (0.684) | -40.204 | < 0.001 |
| Cerebelum 6 L | -1.043 (0.801) | -0.195 (0.616) | -26.531 | < 0.001 |
| Cerebelum 6 R | -1.129 (0.866) | -0.469 (0.613) | -19.675 | < 0.001 |
| Cerebelum 7b L | -1.744 (0.797) | -0.063 (0.721) | -49.471 | < 0.001 |
| Cerebelum 7b R | -1.581 (0.86) | -0.246 (0.712) | -37.804 | < 0.001 |
| Cerebelum 8 L | -1.207 (0.824) | -0.496 (0.658) | -21.305 | < 0.001 |
| Cerebelum 8 R | -1.36 (0.829) | -0.383 (0.69) | -28.657 | < 0.001 |
| Frontal Sup R | -2.468 (0.786) | -2.595 (0.685) | 3.853 | < 0.001 |
| Frontal Mid R | -2.03 (0.748) | -2.303 (0.752) | 8.142 | < 0.001 |
| Frontal Inf Oper L | -1.839 (0.86) | -3.275 (0.807) | 38.496 | < 0.001 |
| Frontal Inf Oper R | -2.396 (0.897) | -1.604 (0.765) | -21.234 | < 0.001 |
| Frontal Inf Tri R | -2.044 (0.849) | -2.193 (0.724) | 4.219 | < 0.001 |
| Rolandic Oper R | -1.888 (0.756) | -2.587 (0.641) | 22.297 | < 0.001 |
| Supp Motor Area R | -2.035 (0.859) | -2.157 (0.704) | 3.469 | 0.001 |
| Olfactory R | -1.82 (0.854) | -1.201 (0.696) | -17.768 | < 0.001 |
| Frontal Sup Med R | -1.579 (0.78) | -2.001 (0.75) | 12.346 | < 0.001 |
| Frontal Med Orb R | -1.733 (0.866) | -1.841 (0.772) | 2.923 | 0.004 |
| Cingulum Ant L | -1.78 (0.761) | -2.532 (0.685) | 23.218 | < 0.001 |
| Cingulum Ant R | -0.982 (0.723) | -1.2 (0.707) | 6.801 | < 0.001 |
| Cingulum Mid R | -2.871 (0.867) | -2.254 (0.68) | -17.706 | < 0.001 |
| Cingulum Post L | -1.453 (0.878) | -2.48 (0.558) | 31.216 | < 0.001 |
| Cingulum Post R | -1.572 (0.83) | -1.117 (0.708) | -13.207 | < 0.001 |
| ParaHippocampal L | -2.665 (0.883) | 0.574 (0.668) | -92.512 | < 0.001 |
| Postcentral R | -1.893 (0.827) | -1.304 (0.627) | -17.950 | < 0.001 |
| Parietal Sup L | -1.729 (0.844) | -1.088 (0.794) | -17.513 | < 0.001 |
| Parietal Sup R | -2.753 (0.921) | -1.609 (0.748) | -30.469 | < 0.001 |
| Angular L | -2.371 (0.903) | -2.113 (0.584) | -7.586 | < 0.001 |
| Precuneus R | -2.188 (0.883) | -1.838 (0.607) | -10.314 | < 0.001 |
| Caudate R | -0.96 (0.896) | -2.243 (0.825) | 33.312 | < 0.001 |

**Supplementary Table 5**. Between-group comparison on regional frailty-related GMV effects between AD and FTLD. Mean (SD) t-values of the frailty–GMV association estimated separately in Alzheimer’s disease (AD) and frontotemporal lobar degeneration (FTLD) groups using repeated subsampling (n = 1000) and models adjusted for scanner effects.

| **Regions** | **Model** | | **Frailty** | |
| --- | --- | --- | --- | --- |
|  | **R^2^_adj_** | ***P*_FDR_** | ***t*** | ***P*_FDR_** |
| Precentral L - Cingulum Mid R | 0.149 | < 0.001 | 2.273 | 0.024 |
| Precentral L - Lingual L | 0.180 | < 0.001 | -2.693 | 0.008 |
| Precentral L - Lingual R | 0.202 | < 0.001 | -2.080 | 0.039 |
| Precentral L - Fusiform L | 0.246 | < 0.001 | -2.726 | 0.007 |
| Precentral L - Temporal Pole Mid R | 0.220 | < 0.001 | -1.979 | 0.049 |
| Precentral L - Temporal Inf L | 0.290 | < 0.001 | -2.273 | 0.024 |
| Frontal Mid Orb R - Cerebellum 10 L | 0.105 | < 0.001 | 2.010 | 0.045 |
| Frontal Mid Orb R - Occipital Mid L | 0.050 | 0.008 | 2.086 | 0.038 |
| Frontal Mid Orb R - Occipital Inf L | 0.068 | 0.001 | 2.446 | 0.015 |
| Frontal Mid Orb R - Thalamus R | 0.116 | < 0.001 | 2.017 | 0.045 |
| Frontal Mid Orb R - Cerebellum 3 L | 0.035 | 0.029 | 2.202 | 0.029 |
| Cerebellum 6 R - Vermis 8 | 0.226 | < 0.001 | 2.432 | 0.016 |
| Cerebellum 6 R - Vermis 9 | 0.170 | < 0.001 | 2.271 | 0.024 |
| Cerebellum 7b L - Cerebellum 7b R | 0.268 | < 0.001 | 2.217 | 0.028 |
| Cerebellum 7b L - Cerebellum 8 L | 0.473 | < 0.001 | 3.208 | 0.002 |
| Cerebellum 7b L - Cerebellum 8 R | 0.379 | < 0.001 | 2.424 | 0.016 |
| Cerebellum 7b L - Cerebellum 10 L | 0.068 | 0.002 | 3.530 | < 0.001 |
| Cerebellum 7b L - Cerebellum 10 R | 0.106 | < 0.001 | 2.227 | 0.027 |
| Cerebellum 7b L - Vermis 8 | 0.201 | < 0.001 | 2.731 | 0.007 |
| Cerebellum 7b R - Cerebellum 8 L | 0.242 | < 0.001 | 2.409 | 0.017 |
| Cerebellum 7b R - Cerebellum 8 R | 0.367 | < 0.001 | 2.783 | 0.006 |
| Cerebellum 7b R - Cerebellum 10 L | 0.068 | 0.001 | 3.000 | 0.003 |
| Cerebellum 7b R - Cerebellum 10 R | 0.102 | < 0.001 | 1.983 | 0.048 |
| Cerebellum 7b R - Vermis 8 | 0.155 | < 0.001 | 2.745 | 0.007 |
| Cerebellum 8 L - Cerebellum 8 R | 0.462 | < 0.001 | 2.216 | 0.028 |
| Cerebellum 8 L - Cerebellum 9 L | 0.139 | < 0.001 | 2.020 | 0.044 |
| Cerebellum 8 L - Cerebellum 9 R | 0.203 | < 0.001 | 2.383 | 0.018 |
| Cerebellum 8 L - Cerebellum 10 L | 0.115 | < 0.001 | 3.432 | 0.001 |
| Cerebellum 8 L - Vermis 8 | 0.275 | < 0.001 | 2.549 | 0.011 |
| Cerebellum 8 R - Cerebellum 9 L | 0.119 | < 0.001 | 2.063 | 0.040 |
| Cerebellum 8 R - Cerebellum 9 R | 0.193 | < 0.001 | 2.161 | 0.032 |
| Cerebellum 8 R - Cerebellum 10 L | 0.065 | 0.002 | 2.590 | 0.010 |
| Cerebellum 8 R - Vermis 8 | 0.228 | < 0.001 | 2.276 | 0.024 |
| Cerebellum 9 R - Vermis 8 | 0.136 | < 0.001 | 2.261 | 0.025 |
| Cerebellum 10 R - Vermis 4 5 | 0.049 | 0.008 | 2.240 | 0.026 |
| Frontal Inf Oper L - Cerebellum 6 R | 0.096 | < 0.001 | -1.975 | 0.049 |
| Frontal Inf Oper L - Rectus R | 0.086 | < 0.001 | 2.021 | 0.044 |
| Frontal Inf Oper L - Pallidum L | 0.068 | 0.001 | -3.227 | 0.001 |
| Frontal Inf Oper L - Temporal Inf L | 0.125 | < 0.001 | -2.663 | 0.008 |
| Vermis 7 Vermis 8 | 0.239 | < 0.001 | 3.019 | 0.003 |
| Vermis 7 Vermis 10 | 0.284 | < 0.001 | 2.037 | 0.043 |
| Frontal Inf Oper R - Heschl R | 0.088 | < 0.001 | 2.001 | 0.047 |
| Frontal Inf Oper R - Temporal Inf R | 0.184 | < 0.001 | -2.070 | 0.039 |
| Frontal Inf Oper R - Cerebellum Crus1 R | 0.093 | < 0.001 | 2.306 | 0.022 |
| Frontal Inf Oper R - Cerebellum Crus2 R | 0.065 | 0.002 | 2.564 | 0.011 |
| Frontal Inf Tri L - Cerebellum 6 R | 0.056 | 0.004 | -2.192 | 0.029 |
| Frontal Inf Tri L - Frontal Inf Orb L | 0.276 | < 0.001 | -2.526 | 0.012 |
| Frontal Inf Tri L - Occipital Inf L | 0.062 | 0.002 | -2.140 | 0.033 |
| Frontal Inf Tri L - Occipital Inf R | 0.053 | 0.006 | -2.112 | 0.036 |
| Frontal Inf Tri L - Fusiform L | 0.060 | 0.003 | -2.256 | 0.025 |
| Frontal Inf Tri L - Fusiform R | 0.113 | < 0.001 | -2.520 | 0.012 |
| Frontal Inf Tri L - Temporal Mid L | 0.193 | < 0.001 | -2.088 | 0.038 |
| Frontal Inf Tri L - Temporal Inf L | 0.136 | < 0.001 | -3.685 | < 0.001 |
| Frontal Inf Tri L - Temporal Inf R | 0.143 | < 0.001 | -2.766 | 0.006 |
| Frontal Inf Tri R - Cerebellum 6 R | 0.071 | 0.001 | -2.003 | 0.046 |
| Frontal Inf Tri R - Vermis 4 5 | 0.046 | 0.011 | 2.020 | 0.044 |
| Frontal Inf Tri R - Fusiform L | 0.048 | 0.009 | -2.175 | 0.031 |
| Frontal Inf Tri R - Temporal Mid L | 0.084 | < 0.001 | -2.055 | 0.041 |
| Frontal Inf Tri R - Temporal Inf R | 0.162 | < 0.001 | -3.018 | 0.003 |
| Frontal Inf Tri R - Cerebellum 6 L | 0.097 | < 0.001 | -2.140 | 0.033 |
| Frontal Inf Orb L - Supp Motor Area R | 0.102 | < 0.001 | -2.349 | 0.020 |
| Frontal Inf Orb L - Pallidum L | 0.131 | < 0.001 | 2.556 | 0.011 |
| Frontal Inf Orb L - Heschl L | 0.076 | 0.001 | 2.346 | 0.020 |
| Frontal Inf Orb L - Heschl R | 0.147 | < 0.001 | 2.300 | 0.022 |
| Frontal Inf Orb L - Temporal Sup L | 0.145 | < 0.001 | 2.054 | 0.041 |
| Frontal Inf Orb R - Vermis 3 | 0.072 | 0.001 | 2.783 | 0.006 |
| Frontal Inf Orb R - Vermis 4 5 | 0.137 | < 0.001 | 2.025 | 0.044 |
| Frontal Inf Orb R - Frontal Sup Medial R | 0.131 | < 0.001 | -2.317 | 0.021 |
| Frontal Inf Orb R - Fusiform R | 0.168 | < 0.001 | 2.535 | 0.012 |
| Frontal Inf Orb R - Heschl L | 0.085 | < 0.001 | 2.517 | 0.012 |
| Frontal Inf Orb R - Heschl R | 0.126 | < 0.001 | 2.402 | 0.017 |
| Frontal Inf Orb R - Temporal Pole Mid L | 0.130 | < 0.001 | 2.042 | 0.042 |
| Frontal Inf Orb R - Cerebellum 3 L | 0.045 | 0.012 | 2.059 | 0.041 |
| Frontal Inf Orb R - Cerebellum 4 5 R | 0.116 | < 0.001 | 2.335 | 0.020 |
| Rolandic Oper L - Cerebellum 8 L | 0.097 | < 0.001 | 2.582 | 0.010 |
| Rolandic Oper L - Cerebellum 9 R | 0.047 | 0.010 | 2.177 | 0.030 |
| Rolandic Oper L - Temporal Pole Mid L | 0.086 | < 0.001 | -2.035 | 0.043 |
| Rolandic Oper R - Parietal Inf R | 0.181 | < 0.001 | 2.826 | 0.005 |
| Rolandic Oper R - Angular L | 0.108 | < 0.001 | 2.268 | 0.024 |
| Rolandic Oper R - Angular R | 0.093 | < 0.001 | 4.150 | < 0.001 |
| Rolandic Oper R - Thalamus R | 0.129 | < 0.001 | 2.102 | 0.037 |
| Supp Motor Area L - Cerebellum 6 R | 0.130 | < 0.001 | -2.337 | 0.020 |
| Supp Motor Area L - Vermis 3 | 0.058 | 0.004 | -2.793 | 0.006 |
| Supp Motor Area L - Vermis 6 | 0.096 | < 0.001 | -2.517 | 0.012 |
| Supp Motor Area L - Postcentral L | 0.262 | < 0.001 | -2.053 | 0.041 |
| Supp Motor Area L - Pallidum L | 0.039 | 0.021 | -2.271 | 0.024 |
| Supp Motor Area L - Cerebellum 3 L | 0.054 | 0.005 | -2.433 | 0.016 |
| Supp Motor Area L - Cerebellum 3 R | 0.098 | < 0.001 | -2.474 | 0.014 |
| Supp Motor Area L - Cerebellum 4 5 L | 0.076 | 0.001 | -2.141 | 0.033 |
| Precentral R - Occipital Inf R | 0.139 | < 0.001 | -2.128 | 0.034 |
| Precentral R - Parietal Sup R | 0.135 | < 0.001 | 2.420 | 0.016 |
| Precentral R - Parietal Inf R | 0.112 | < 0.001 | 1.988 | 0.048 |
| Precentral R - Cerebellum Crus2 L | 0.058 | 0.004 | 2.453 | 0.015 |
| Precentral R - Cerebellum Crus2 R | 0.073 | 0.001 | 2.429 | 0.016 |
| Supp Motor Area R - Cerebellum 10 R | 0.066 | 0.002 | -2.054 | 0.041 |
| Supp Motor Area R - Vermis 3 | 0.109 | < 0.001 | -2.834 | 0.005 |
| Supp Motor Area R - Cingulum Ant L | 0.083 | < 0.001 | -2.106 | 0.036 |
| Supp Motor Area R - Lingual L | 0.196 | < 0.001 | -2.103 | 0.037 |
| Supp Motor Area R - Lingual R | 0.186 | < 0.001 | -2.042 | 0.042 |
| Supp Motor Area R - Fusiform L | 0.248 | < 0.001 | -2.013 | 0.045 |
| Supp Motor Area R - Fusiform R | 0.254 | < 0.001 | -3.072 | 0.002 |
| Supp Motor Area R - Temporal Pole Sup L | 0.165 | < 0.001 | -2.035 | 0.043 |
| Supp Motor Area R - Temporal Inf R | 0.267 | < 0.001 | -2.434 | 0.016 |
| Olfactory L - Cerebellum 7b L | 0.073 | 0.001 | 2.254 | 0.025 |
| Olfactory L - Parietal Inf R | 0.147 | < 0.001 | 2.391 | 0.018 |
| Olfactory L - Caudate R | 0.093 | < 0.001 | 2.364 | 0.019 |
| Olfactory R - Cerebellum 10 R | 0.085 | < 0.001 | -2.030 | 0.043 |
| Olfactory R - Frontal Sup Medial R | 0.114 | < 0.001 | -2.500 | 0.013 |
| Olfactory R - Cingulum Ant L | 0.119 | < 0.001 | -2.319 | 0.021 |
| Olfactory R - Cingulum Mid L | 0.090 | < 0.001 | -2.017 | 0.045 |
| Frontal Sup Medial L - Rectus L | 0.179 | < 0.001 | -2.826 | 0.005 |
| Frontal Sup Medial L - Rectus R | 0.150 | < 0.001 | -2.894 | 0.004 |
| Frontal Sup Medial L - Occipital Inf R | 0.042 | 0.016 | -1.970 | 0.050 |
| Frontal Sup Medial L - Precuneus L | 0.168 | < 0.001 | -2.514 | 0.013 |
| Frontal Sup Medial L - Precuneus R | 0.077 | 0.001 | -2.566 | 0.011 |
| Frontal Sup Medial L - Temporal Mid L | 0.180 | < 0.001 | -2.440 | 0.015 |
| Frontal Sup Medial L - Temporal Mid R | 0.121 | < 0.001 | -2.701 | 0.007 |
| Frontal Sup Medial L - Temporal Inf L | 0.186 | < 0.001 | -2.794 | 0.006 |
| Frontal Sup Medial L - Temporal Inf R | 0.157 | < 0.001 | -2.451 | 0.015 |
| Frontal Sup Medial L - Cerebellum Crus2 R | 0.152 | < 0.001 | -2.851 | 0.005 |
| Frontal Sup Medial R - Cerebellum 7b L | 0.077 | 0.001 | 2.381 | 0.018 |
| Frontal Sup Medial R - Rectus R | 0.218 | < 0.001 | -2.096 | 0.037 |
| Frontal Sup Medial R - Amygdala L | 0.129 | < 0.001 | -2.106 | 0.036 |
| Frontal Med Orb L - ParaHippocampal L | 0.113 | < 0.001 | -2.279 | 0.024 |
| Frontal Med Orb L - Amygdala L | 0.075 | 0.001 | -2.417 | 0.016 |
| Frontal Med Orb L - Parietal Inf R | 0.034 | 0.032 | 2.025 | 0.044 |
| Frontal Med Orb R - Cingulum Ant L | 0.276 | < 0.001 | -2.657 | 0.008 |
| Frontal Med Orb R - Amygdala L | 0.084 | < 0.001 | -2.137 | 0.034 |
| Frontal Med Orb R - Parietal Inf R | 0.030 | 0.046 | 2.000 | 0.047 |
| Rectus L - Vermis 4 5 | 0.101 | < 0.001 | 2.623 | 0.009 |
| Rectus L - Cingulum Ant L | 0.172 | < 0.001 | -2.063 | 0.040 |
| Rectus L - Cingulum Ant R | 0.201 | < 0.001 | -1.977 | 0.049 |
| Rectus L - Amygdala L | 0.205 | < 0.001 | -2.290 | 0.023 |
| Rectus L - Cuneus L | 0.087 | < 0.001 | -2.503 | 0.013 |
| Rectus L - Occipital Sup L | 0.057 | 0.004 | -2.234 | 0.026 |
| Rectus L - Occipital Sup R | 0.070 | 0.001 | -2.023 | 0.044 |
| Rectus L - Parietal Inf R | 0.194 | < 0.001 | 1.978 | 0.049 |
| Rectus L - Temporal Sup R | 0.125 | < 0.001 | -2.605 | 0.010 |
| Rectus L - Cerebellum 3 L | 0.034 | 0.031 | 2.075 | 0.039 |
| Rectus L - Cerebellum 4 5 L | 0.056 | 0.005 | 2.456 | 0.015 |
| Rectus L - Cerebellum 4 5 R | 0.067 | 0.002 | 3.043 | 0.003 |
| Rectus R - Cerebellum 6 R | 0.070 | 0.001 | 2.067 | 0.040 |
| Rectus R - Vermis 4 5 | 0.116 | < 0.001 | 2.374 | 0.018 |
| Rectus R - Cingulum Ant L | 0.180 | < 0.001 | -2.137 | 0.034 |
| Rectus R - Cingulum Ant R | 0.275 | < 0.001 | -2.854 | 0.005 |
| Rectus R - Amygdala L | 0.211 | < 0.001 | -3.125 | 0.002 |
| Rectus R - Occipital Inf L | 0.156 | < 0.001 | 3.137 | 0.002 |
| Rectus R - Temporal Sup R | 0.093 | < 0.001 | -2.235 | 0.026 |
| Rectus R - Cerebellum 3 R | 0.063 | 0.002 | 2.301 | 0.022 |
| Rectus R - Cerebellum 4 5 L | 0.054 | 0.005 | 2.477 | 0.014 |
| Rectus R - Cerebellum 4 5 R | 0.040 | 0.018 | 2.634 | 0.009 |
| Rectus R - Cerebellum 6 L | 0.057 | 0.004 | 2.417 | 0.016 |
| Frontal Sup L - Cerebellum 7b R | 0.079 | 0.001 | -2.048 | 0.042 |
| Frontal Sup L - Frontal Sup R | 0.378 | < 0.001 | 2.124 | 0.035 |
| Frontal Sup L - Temporal Inf R | 0.249 | < 0.001 | -2.328 | 0.021 |
| Insula R - Pallidum R | 0.105 | < 0.001 | 2.325 | 0.021 |
| Insula R - Thalamus R | 0.121 | < 0.001 | 2.152 | 0.032 |
| Cingulum Ant L - Vermis 6 | 0.048 | 0.009 | -2.128 | 0.034 |
| Cingulum Ant L - ParaHippocampal L | 0.041 | 0.017 | -2.592 | 0.010 |
| Cingulum Ant L - Cerebellum 3 L | 0.035 | 0.029 | -2.165 | 0.031 |
| Cingulum Ant R - Amygdala L | 0.087 | < 0.001 | -2.983 | 0.003 |
| Cingulum Ant R - Temporal Pole Mid R | 0.094 | < 0.001 | -2.502 | 0.013 |
| Cingulum Mid L - Vermis 3 | 0.040 | 0.019 | -2.023 | 0.044 |
| Cingulum Mid L - Vermis 6 | 0.045 | 0.012 | -2.302 | 0.022 |
| Cingulum Mid L - Vermis 7 | 0.102 | < 0.001 | -2.037 | 0.043 |
| Cingulum Mid R - Cerebellum 10 R | 0.073 | 0.001 | -1.984 | 0.048 |
| Cingulum Mid R - Vermis 3 | 0.071 | 0.001 | -2.263 | 0.025 |
| Cingulum Mid R - Vermis 6 | 0.053 | 0.006 | -2.293 | 0.023 |
| Cingulum Mid R - Fusiform L | 0.180 | < 0.001 | -2.231 | 0.027 |
| Cingulum Mid R - Fusiform R | 0.217 | < 0.001 | -2.338 | 0.020 |
| Cingulum Mid R - Cerebellum Crus2 R | 0.152 | < 0.001 | 2.387 | 0.018 |
| Cingulum Post L - Vermis 4 5 | 0.178 | < 0.001 | 2.225 | 0.027 |
| Cingulum Post L - Hippocampus L | 0.156 | < 0.001 | 2.098 | 0.037 |
| Cingulum Post L - Heschl R | 0.166 | < 0.001 | 2.061 | 0.040 |
| Cingulum Post R - ParaHippocampal R | 0.082 | < 0.001 | 2.221 | 0.027 |
| Cingulum Post R - Heschl R | 0.114 | < 0.001 | 2.498 | 0.013 |
| Hippocampus L - Cerebellum 6 R | 0.064 | 0.002 | 2.193 | 0.029 |
| Hippocampus L - Postcentral L | 0.091 | < 0.001 | -2.470 | 0.014 |
| Hippocampus L - Angular L | 0.150 | < 0.001 | 2.102 | 0.037 |
| Hippocampus L - Cerebellum 4 5 L | 0.125 | < 0.001 | 2.630 | 0.009 |
| Hippocampus R - Cerebellum 6 R | 0.064 | 0.002 | 1.998 | 0.047 |
| Hippocampus R - Vermis 4 5 | 0.197 | < 0.001 | 2.031 | 0.043 |
| Hippocampus R - Thalamus L | 0.082 | < 0.001 | 2.019 | 0.045 |
| Hippocampus R - Temporal Mid L | 0.190 | < 0.001 | 2.783 | 0.006 |
| Hippocampus R - Temporal Mid R | 0.202 | < 0.001 | 2.763 | 0.006 |
| ParaHippocampal L - Cerebellum 6 R | 0.061 | 0.003 | 2.477 | 0.014 |
| ParaHippocampal L - ParaHippocampal R | 0.352 | < 0.001 | 2.479 | 0.014 |
| ParaHippocampal L - Fusiform R | 0.173 | < 0.001 | 3.039 | 0.003 |
| ParaHippocampal L - Parietal Inf R | 0.066 | 0.002 | 2.468 | 0.014 |
| ParaHippocampal L - Temporal Pole Mid L | 0.187 | < 0.001 | 2.180 | 0.030 |
| ParaHippocampal L - Temporal Pole Mid R | 0.137 | < 0.001 | 2.381 | 0.018 |
| ParaHippocampal L - Temporal Inf L | 0.205 | < 0.001 | 2.161 | 0.032 |
| ParaHippocampal L - Temporal Inf R | 0.171 | < 0.001 | 2.355 | 0.019 |
| ParaHippocampal L - Cerebellum Crus2 R | 0.050 | 0.008 | 2.397 | 0.017 |
| Frontal Sup R - Olfactory R | 0.130 | < 0.001 | -2.272 | 0.024 |
| Frontal Sup R - Frontal Sup Orb L | 0.157 | < 0.001 | -2.598 | 0.010 |
| Frontal Sup R - Temporal Inf R | 0.312 | < 0.001 | -2.793 | 0.006 |
| ParaHippocampal R - Cerebellum 6 R | 0.086 | < 0.001 | 2.202 | 0.029 |
| ParaHippocampal R - Occipital Inf L | 0.237 | < 0.001 | 2.018 | 0.045 |
| ParaHippocampal R - Fusiform L | 0.315 | < 0.001 | 3.415 | 0.001 |
| ParaHippocampal R - Fusiform R | 0.276 | < 0.001 | 3.225 | 0.001 |
| ParaHippocampal R - Parietal Sup R | 0.158 | < 0.001 | 1.986 | 0.048 |
| ParaHippocampal R - Thalamus R | 0.107 | < 0.001 | 2.252 | 0.025 |
| ParaHippocampal R - Temporal Mid L | 0.081 | < 0.001 | 2.567 | 0.011 |
| ParaHippocampal R - Temporal Mid R | 0.164 | < 0.001 | 2.524 | 0.012 |
| ParaHippocampal R - Temporal Pole Mid L | 0.220 | < 0.001 | 2.592 | 0.010 |
| ParaHippocampal R - Temporal Pole Mid R | 0.193 | < 0.001 | 1.986 | 0.048 |
| ParaHippocampal R - Temporal Inf L | 0.277 | < 0.001 | 3.523 | 0.001 |
| ParaHippocampal R - Temporal Inf R | 0.256 | < 0.001 | 3.206 | 0.002 |
| ParaHippocampal R - Cerebellum 3 L | 0.145 | < 0.001 | 2.314 | 0.021 |
| ParaHippocampal R - Cerebellum 4 5 L | 0.081 | < 0.001 | 2.158 | 0.032 |
| ParaHippocampal R - Cerebellum 4 5 R | 0.103 | < 0.001 | 2.637 | 0.009 |
| Amygdala L - Vermis 6 | 0.031 | 0.041 | -2.125 | 0.035 |
| Amygdala L - Vermis 10 | 0.087 | < 0.001 | 2.129 | 0.034 |
| Calcarine L - Vermis 4 5 | 0.177 | < 0.001 | 2.145 | 0.033 |
| Calcarine L - Thalamus R | 0.120 | < 0.001 | 2.120 | 0.035 |
| Calcarine L - Heschl R | 0.107 | < 0.001 | 2.533 | 0.012 |
| Calcarine R - Vermis 4 5 | 0.230 | < 0.001 | 2.724 | 0.007 |
| Calcarine R - Pallidum L | 0.098 | < 0.001 | 2.827 | 0.005 |
| Calcarine R - Thalamus R | 0.124 | < 0.001 | 2.454 | 0.015 |
| Calcarine R - Heschl R | 0.141 | < 0.001 | 3.012 | 0.003 |
| Cuneus L - Vermis 7 | 0.125 | < 0.001 | -2.497 | 0.013 |
| Cuneus L - Occipital Inf R | 0.106 | < 0.001 | -2.805 | 0.005 |
| Cuneus L - Fusiform L | 0.144 | < 0.001 | -2.459 | 0.015 |
| Cuneus L - Fusiform R | 0.181 | < 0.001 | -2.212 | 0.028 |
| Cuneus L - Temporal Inf L | 0.089 | < 0.001 | -2.592 | 0.010 |
| Cuneus L - Temporal Inf R | 0.125 | < 0.001 | -2.491 | 0.013 |
| Cuneus L - Cerebellum 3 L | 0.046 | 0.011 | -2.085 | 0.038 |
| Cuneus R - Fusiform L | 0.194 | < 0.001 | -2.457 | 0.015 |
| Cuneus R - Fusiform R | 0.216 | < 0.001 | -2.002 | 0.046 |
| Cuneus R - Pallidum R | 0.095 | < 0.001 | 2.328 | 0.021 |
| Cuneus R - Thalamus R | 0.174 | < 0.001 | 3.130 | 0.002 |
| Cuneus R - Temporal Inf L | 0.087 | < 0.001 | -2.121 | 0.035 |
| Cuneus R - Temporal Inf R | 0.117 | < 0.001 | -2.429 | 0.016 |
| Lingual L - Vermis 4 5 | 0.151 | < 0.001 | 2.225 | 0.027 |
| Lingual L - Vermis 8 | 0.097 | < 0.001 | 2.492 | 0.013 |
| Lingual L - Paracentral Lobule L | 0.235 | < 0.001 | -2.009 | 0.046 |
| Lingual L - Heschl R | 0.061 | 0.003 | 2.279 | 0.024 |
| Lingual L - Cerebellum 3 L | 0.061 | 0.003 | 2.015 | 0.045 |
| Lingual L - Cerebellum 4 5 L | 0.175 | < 0.001 | 2.773 | 0.006 |
| Lingual L - Cerebellum 4 5 R | 0.190 | < 0.001 | 2.441 | 0.015 |
| Lingual R - Vermis 8 | 0.104 | < 0.001 | 2.527 | 0.012 |
| Lingual R - Paracentral Lobule L | 0.250 | < 0.001 | -2.162 | 0.032 |
| Lingual R - Paracentral Lobule R | 0.238 | < 0.001 | -2.029 | 0.044 |
| Lingual R - Heschl R | 0.063 | 0.002 | 2.739 | 0.007 |
| Lingual R - Temporal Pole Sup R | 0.135 | < 0.001 | 2.176 | 0.031 |
| Lingual R - Cerebellum 4 5 R | 0.219 | < 0.001 | 2.016 | 0.045 |
| Occipital Sup L - Vermis 4 5 | 0.126 | < 0.001 | 2.069 | 0.040 |
| Occipital Sup L - Fusiform L | 0.208 | < 0.001 | -2.055 | 0.041 |
| Occipital Sup L - Fusiform R | 0.244 | < 0.001 | -2.048 | 0.042 |
| Occipital Sup L - Angular R | 0.071 | 0.001 | 2.423 | 0.016 |
| Occipital Sup L - Pallidum R | 0.107 | < 0.001 | 2.017 | 0.045 |
| Occipital Sup L - Thalamus R | 0.095 | < 0.001 | 2.605 | 0.010 |
| Occipital Sup L - Temporal Inf R | 0.094 | < 0.001 | -2.214 | 0.028 |
| Frontal Sup Orb L - Vermis 3 | 0.058 | 0.004 | 2.006 | 0.046 |
| Frontal Sup Orb L - Frontal Sup Medial L | 0.112 | < 0.001 | -1.975 | 0.049 |
| Frontal Sup Orb L - Cingulum Ant L | 0.153 | < 0.001 | -2.983 | 0.003 |
| Frontal Sup Orb L - Cingulum Ant R | 0.198 | < 0.001 | -2.168 | 0.031 |
| Frontal Sup Orb L - Cuneus L | 0.070 | 0.001 | -2.582 | 0.010 |
| Frontal Sup Orb L - Angular L | 0.118 | < 0.001 | -2.076 | 0.039 |
| Frontal Sup Orb L - Cerebellum 3 L | 0.089 | < 0.001 | 3.844 | < 0.001 |
| Occipital Sup R - Pallidum R | 0.138 | < 0.001 | 2.108 | 0.036 |
| Occipital Sup R - Thalamus R | 0.146 | < 0.001 | 2.461 | 0.015 |
| Occipital Mid L - Vermis 4 5 | 0.172 | < 0.001 | 2.253 | 0.025 |
| Occipital Mid L - Caudate L | 0.079 | < 0.001 | 2.324 | 0.021 |
| Occipital Mid L - Pallidum L | 0.037 | 0.025 | 2.295 | 0.023 |
| Occipital Mid L - Thalamus R | 0.116 | < 0.001 | 2.386 | 0.018 |
| Occipital Mid L - Heschl R | 0.068 | 0.001 | 2.164 | 0.031 |
| Occipital Mid L - Temporal Inf R | 0.194 | < 0.001 | -2.361 | 0.019 |
| Occipital Mid R - Vermis 9 | 0.109 | < 0.001 | 2.015 | 0.045 |
| Occipital Mid R - Thalamus R | 0.149 | < 0.001 | 2.478 | 0.014 |
| Occipital Mid R - Heschl R | 0.063 | 0.002 | 2.149 | 0.033 |
| Occipital Mid R - Temporal Inf R | 0.127 | < 0.001 | -2.116 | 0.035 |
| Occipital Inf L - Vermis 4 5 | 0.114 | < 0.001 | 2.242 | 0.026 |
| Occipital Inf L - Vermis 6 | 0.087 | < 0.001 | 2.715 | 0.007 |
| Occipital Inf L - Vermis 8 | 0.102 | < 0.001 | 2.541 | 0.012 |
| Occipital Inf L - Cerebellum Crus2 R | 0.131 | < 0.001 | 2.391 | 0.018 |
| Occipital Inf L - Cerebellum 4 5 L | 0.186 | < 0.001 | 3.336 | 0.001 |
| Occipital Inf L - Cerebellum 4 5 R | 0.104 | < 0.001 | 2.348 | 0.020 |
| Occipital Inf R - Postcentral L | 0.151 | < 0.001 | -2.103 | 0.036 |
| Occipital Inf R - Paracentral Lobule R | 0.174 | < 0.001 | -2.368 | 0.019 |
| Fusiform L - Cerebellum 6 R | 0.240 | < 0.001 | 2.090 | 0.038 |
| Fusiform L - Vermis 4 5 | 0.168 | < 0.001 | 2.943 | 0.004 |
| Fusiform L - Vermis 6 | 0.116 | < 0.001 | 2.901 | 0.004 |
| Fusiform L - Vermis 8 | 0.139 | < 0.001 | 2.577 | 0.011 |
| Fusiform L - Paracentral Lobule L | 0.202 | < 0.001 | -2.325 | 0.021 |
| Fusiform L - Cerebellum Crus2 L | 0.239 | < 0.001 | 2.323 | 0.021 |
| Fusiform L - Cerebellum Crus2 R | 0.152 | < 0.001 | 2.764 | 0.006 |
| Fusiform L - Cerebellum 3 L | 0.077 | 0.001 | 2.042 | 0.042 |
| Fusiform L - Cerebellum 4 5 L | 0.170 | < 0.001 | 2.237 | 0.026 |
| Fusiform L - Cerebellum 4 5 R | 0.181 | < 0.001 | 2.851 | 0.005 |
| Fusiform L - Cerebellum 6 L | 0.279 | < 0.001 | 2.300 | 0.022 |
| Fusiform R - Vermis 6 | 0.134 | < 0.001 | 2.317 | 0.021 |
| Fusiform R - Vermis 8 | 0.132 | < 0.001 | 2.764 | 0.006 |
| Fusiform R - Vermis 9 | 0.091 | < 0.001 | 2.141 | 0.033 |
| Fusiform R - Paracentral Lobule L | 0.236 | < 0.001 | -2.475 | 0.014 |
| Fusiform R - Heschl R | 0.075 | 0.001 | 1.970 | 0.050 |
| Fusiform R - Temporal Pole Sup R | 0.168 | < 0.001 | 2.481 | 0.014 |
| Fusiform R - Temporal Pole Mid R | 0.160 | < 0.001 | 2.130 | 0.034 |
| Fusiform R - Cerebellum Crus2 R | 0.269 | < 0.001 | 2.401 | 0.017 |
| Fusiform R - Cerebellum 4 5 R | 0.205 | < 0.001 | 3.090 | 0.002 |
| Postcentral L - Cerebellum 9 L | 0.060 | 0.003 | 2.348 | 0.020 |
| Postcentral L - Parietal Sup L | 0.198 | < 0.001 | 2.125 | 0.035 |
| Postcentral L - Pallidum L | 0.077 | 0.001 | -2.188 | 0.030 |
| Postcentral L - Temporal Pole Mid L | 0.141 | < 0.001 | -2.017 | 0.045 |
| Postcentral L - Cerebellum Crus2 R | 0.091 | < 0.001 | 2.126 | 0.035 |
| Postcentral R - Parietal Sup R | 0.196 | < 0.001 | 2.409 | 0.017 |
| Parietal Sup L - Precuneus L | 0.096 | < 0.001 | 1.981 | 0.049 |
| Parietal Sup L - Caudate L | 0.127 | < 0.001 | 3.683 | < 0.001 |
| Parietal Sup L - Caudate R | 0.114 | < 0.001 | 3.005 | 0.003 |
| Parietal Sup L - Heschl R | 0.104 | < 0.001 | 3.070 | 0.002 |
| Parietal Sup L - Temporal Sup L | 0.094 | < 0.001 | 2.235 | 0.026 |
| Frontal Sup Orb R - Frontal Inf Oper R | 0.131 | < 0.001 | 1.971 | 0.050 |
| Frontal Sup Orb R - Cingulum Ant L | 0.093 | < 0.001 | -2.478 | 0.014 |
| Frontal Sup Orb R - Cingulum Ant R | 0.167 | < 0.001 | -2.054 | 0.041 |
| Frontal Sup Orb R - Occipital Inf L | 0.082 | < 0.001 | 2.045 | 0.042 |
| Frontal Sup Orb R - Cerebellum 3 L | 0.063 | 0.002 | 2.512 | 0.013 |
| Parietal Sup R - Vermis 8 | 0.067 | 0.002 | 2.836 | 0.005 |
| Parietal Sup R - Caudate L | 0.133 | < 0.001 | 2.980 | 0.003 |
| Parietal Sup R - Caudate R | 0.090 | < 0.001 | 2.415 | 0.016 |
| Parietal Sup R - Heschl R | 0.092 | < 0.001 | 2.361 | 0.019 |
| Parietal Inf L - Cerebellum 6 R | 0.161 | < 0.001 | -2.663 | 0.008 |
| Parietal Inf L - Cerebellum 7b R | 0.135 | < 0.001 | -1.984 | 0.048 |
| Parietal Inf L - Vermis 1 2 | 0.030 | 0.043 | -2.137 | 0.034 |
| Parietal Inf L - Heschl R | 0.053 | 0.006 | 2.187 | 0.030 |
| Parietal Inf R - Vermis 1 2 | 0.108 | < 0.001 | -2.105 | 0.036 |
| Parietal Inf R - Heschl R | 0.092 | < 0.001 | 3.244 | 0.001 |
| Angular L - Temporal Pole Sup R | 0.053 | 0.006 | 2.326 | 0.021 |
| Angular R - Vermis 7 | 0.056 | 0.005 | -2.096 | 0.037 |
| Angular R - Heschl R | 0.031 | 0.040 | 2.246 | 0.026 |
| Angular R - Temporal Sup R | 0.075 | 0.001 | 2.030 | 0.043 |
| Precuneus L - Cerebellum 7b R | 0.074 | 0.001 | -1.983 | 0.049 |
| Precuneus R - Vermis 8 | 0.077 | 0.001 | 2.053 | 0.041 |
| Paracentral Lobule L - Cerebellum 10 L | 0.112 | < 0.001 | 2.089 | 0.038 |
| Paracentral Lobule L - Temporal Inf R | 0.151 | < 0.001 | -2.388 | 0.018 |
| Frontal Mid L - Precuneus L | 0.178 | < 0.001 | -1.971 | 0.050 |
| Frontal Mid L - Temporal Mid R | 0.086 | < 0.001 | -2.047 | 0.042 |
| Frontal Mid L - Temporal Inf L | 0.327 | < 0.001 | -2.188 | 0.030 |
| Frontal Mid L - Temporal Inf R | 0.266 | < 0.001 | -2.240 | 0.026 |
| Caudate L - Heschl R | 0.112 | < 0.001 | 2.823 | 0.005 |
| Caudate L - Temporal Sup L | 0.119 | < 0.001 | 2.499 | 0.013 |
| Caudate L - Cerebellum 4 5 R | 0.041 | 0.018 | 2.432 | 0.016 |
| Caudate R - Cerebellum 10 R | 0.072 | 0.001 | -2.223 | 0.027 |
| Caudate R - Heschl R | 0.107 | < 0.001 | 2.722 | 0.007 |
| Caudate R - Temporal Sup L | 0.132 | < 0.001 | 2.042 | 0.042 |
| Putamen L - Cerebellum 7b R | 0.205 | < 0.001 | -1.979 | 0.049 |
| Putamen L - Cerebellum 8 L | 0.073 | 0.001 | -2.360 | 0.019 |
| Putamen L - Thalamus L | 0.210 | < 0.001 | 2.263 | 0.025 |
| Putamen L - Heschl R | 0.154 | < 0.001 | 1.995 | 0.047 |
| Putamen L - Cerebellum 4 5 L | 0.089 | < 0.001 | -2.666 | 0.008 |
| Putamen R - Vermis 6 | 0.111 | < 0.001 | -2.119 | 0.035 |
| Putamen R - Thalamus L | 0.261 | < 0.001 | 1.972 | 0.050 |
| Putamen R - Thalamus R | 0.256 | < 0.001 | 1.988 | 0.048 |
| Putamen R - Heschl R | 0.197 | < 0.001 | 2.164 | 0.031 |
| Putamen R - Cerebellum 3 R | 0.082 | < 0.001 | -2.230 | 0.027 |
| Pallidum L - Heschl R | 0.096 | < 0.001 | 2.660 | 0.008 |
| Pallidum L - Cerebellum 3 L | 0.060 | 0.003 | 2.005 | 0.046 |
| Pallidum R - Vermis 10 | 0.064 | 0.002 | -2.371 | 0.018 |
| Pallidum R - Cerebellum 3 R | 0.063 | 0.002 | -2.087 | 0.038 |
| Pallidum R - Cerebellum 4 5 L | 0.083 | < 0.001 | -2.158 | 0.032 |
| Thalamus L - Cerebellum 10 R | 0.041 | 0.018 | -2.093 | 0.037 |
| Thalamus L - Heschl R | 0.149 | < 0.001 | 3.281 | 0.001 |
| Thalamus R - Heschl R | 0.143 | < 0.001 | 3.151 | 0.002 |
| Thalamus R - Temporal Pole Mid R | 0.217 | < 0.001 | 2.215 | 0.028 |
| Heschl L - Vermis 4 5 | 0.070 | 0.001 | 2.970 | 0.003 |
| Frontal Mid R - Cerebellum 6 R | 0.085 | < 0.001 | -1.991 | 0.048 |
| Frontal Mid R - Lingual L | 0.054 | 0.005 | -2.049 | 0.042 |
| Frontal Mid R - Fusiform L | 0.112 | < 0.001 | -2.539 | 0.012 |
| Frontal Mid R - Temporal Inf R | 0.308 | < 0.001 | -2.750 | 0.006 |
| Heschl R - Cerebellum 10 R | 0.051 | 0.007 | -2.708 | 0.007 |
| Heschl R - Vermis 4 5 | 0.102 | < 0.001 | 3.756 | < 0.001 |
| Temporal Sup L - Vermis 4 5 | 0.102 | < 0.001 | 2.774 | 0.006 |
| Temporal Sup R - Vermis 4 5 | 0.172 | < 0.001 | 2.187 | 0.030 |
| Temporal Sup R - Cerebellum 3 R | 0.114 | < 0.001 | -2.361 | 0.019 |
| Temporal Pole Sup L - Cerebellum 3 L | 0.140 | < 0.001 | 2.271 | 0.024 |
| Temporal Pole Sup L - Cerebellum 4 5 R | 0.082 | < 0.001 | 2.430 | 0.016 |
| Temporal Pole Sup R - Vermis 4 5 | 0.122 | < 0.001 | 2.405 | 0.017 |
| Temporal Pole Sup R - Temporal Pole Mid L | 0.197 | < 0.001 | 1.996 | 0.047 |
| Temporal Pole Sup R - Cerebellum Crus1 R | 0.091 | < 0.001 | 2.405 | 0.017 |
| Temporal Pole Sup R - Cerebellum 4 5 R | 0.099 | < 0.001 | 2.226 | 0.027 |
| Temporal Mid L - Vermis 4 5 | 0.187 | < 0.001 | 2.825 | 0.005 |
| Temporal Mid L - Vermis 10 | 0.094 | < 0.001 | -2.084 | 0.038 |
| Temporal Mid L - Cerebellum 3 L | 0.134 | < 0.001 | 2.423 | 0.016 |
| Temporal Mid L - Cerebellum 4 5 L | 0.152 | < 0.001 | 2.427 | 0.016 |
| Temporal Mid L - Cerebellum 4 5 R | 0.064 | 0.002 | 2.485 | 0.014 |
| Temporal Mid R - Vermis 4 5 | 0.255 | < 0.001 | 2.263 | 0.025 |
| Temporal Mid R - Cerebellum Crus1 R | 0.214 | < 0.001 | 2.066 | 0.040 |
| Temporal Mid R - Cerebellum 4 5 R | 0.112 | < 0.001 | 3.092 | 0.002 |
| Temporal Pole Mid L - Cerebellum 6 R | 0.111 | < 0.001 | 1.972 | 0.050 |
| Temporal Pole Mid L - Cerebellum 7b L | 0.150 | < 0.001 | 2.032 | 0.043 |
| Temporal Pole Mid L - Temporal Pole Mid R | 0.432 | < 0.001 | 2.907 | 0.004 |
| Temporal Pole Mid L - Cerebellum Crus1 L | 0.150 | < 0.001 | 2.677 | 0.008 |
| Temporal Pole Mid L - Cerebellum Crus2 L | 0.094 | < 0.001 | 2.133 | 0.034 |
| Temporal Pole Mid L - Cerebellum Crus2 R | 0.118 | < 0.001 | 2.856 | 0.005 |
| Temporal Pole Mid R - Cerebellum 6 R | 0.083 | < 0.001 | 2.125 | 0.035 |
| Temporal Pole Mid R - Cerebellum 7b R | 0.090 | < 0.001 | 2.057 | 0.041 |
| Temporal Pole Mid R - Cerebellum 10 L | 0.045 | 0.012 | 2.278 | 0.024 |
| Temporal Pole Mid R - Cerebellum 10 R | 0.093 | < 0.001 | 2.016 | 0.045 |
| Temporal Pole Mid R - Temporal Inf L | 0.270 | < 0.001 | 2.196 | 0.029 |
| Temporal Pole Mid R - Cerebellum Crus2 R | 0.150 | < 0.001 | 3.840 | < 0.001 |
| Temporal Inf L - Cerebellum 10 R | 0.152 | < 0.001 | 2.168 | 0.031 |
| Temporal Inf L - Vermis 3 | 0.092 | < 0.001 | 2.113 | 0.036 |
| Temporal Inf L - Vermis 4 5 | 0.154 | < 0.001 | 2.736 | 0.007 |
| Temporal Inf L - Vermis 6 | 0.081 | < 0.001 | 2.524 | 0.012 |
| Temporal Inf L - Cerebellum Crus2 R | 0.227 | < 0.001 | 2.098 | 0.037 |
| Temporal Inf L - Cerebellum 3 L | 0.086 | < 0.001 | 2.963 | 0.003 |
| Temporal Inf L - Cerebellum 4 5 L | 0.167 | < 0.001 | 2.530 | 0.012 |
| Temporal Inf L - Cerebellum 4 5 R | 0.124 | < 0.001 | 2.653 | 0.009 |
| Frontal Mid Orb L - Vermis 3 | 0.103 | < 0.001 | 2.533 | 0.012 |
| Frontal Mid Orb L - Vermis 4 5 | 0.092 | < 0.001 | 2.156 | 0.032 |
| Frontal Mid Orb L - Cerebellum 3 L | 0.077 | 0.001 | 3.058 | 0.002 |
| Temporal Inf R - Cerebellum Crus2 R | 0.192 | < 0.001 | 2.106 | 0.036 |
| Cerebellum Crus1 L - Vermis 8 | 0.128 | < 0.001 | 2.980 | 0.003 |
| Cerebellum Crus1 L - Cerebellum 4 5 L | 0.177 | < 0.001 | 3.145 | 0.002 |
| Cerebellum Crus1 L - Cerebellum 4 5 R | 0.113 | < 0.001 | 3.296 | 0.001 |
| Cerebellum Crus1 R - Vermis 8 | 0.135 | < 0.001 | 3.209 | 0.002 |
| Cerebellum Crus1 R - Cerebellum 4 5 R | 0.107 | < 0.001 | 2.940 | 0.004 |
| Cerebellum Crus2 L - Cerebellum 7b L | 0.261 | < 0.001 | 2.116 | 0.035 |
| Cerebellum Crus2 L - Cerebellum 8 L | 0.252 | < 0.001 | 3.151 | 0.002 |
| Cerebellum Crus2 L - Cerebellum 9 L | 0.139 | < 0.001 | 2.080 | 0.039 |
| Cerebellum Crus2 L - Cerebellum 10 L | 0.141 | < 0.001 | 2.296 | 0.023 |
| Cerebellum Crus2 L - Vermis 8 | 0.209 | < 0.001 | 3.774 | < 0.001 |
| Cerebellum Crus2 L - Cerebellum 4 5 L | 0.161 | < 0.001 | 2.716 | 0.007 |
| Cerebellum Crus2 L - Cerebellum 4 5 R | 0.146 | < 0.001 | 2.137 | 0.034 |
| Cerebellum Crus2 R - Cerebellum 7b L | 0.110 | < 0.001 | 2.907 | 0.004 |
| Cerebellum Crus2 R - Cerebellum 7b R | 0.358 | < 0.001 | 2.842 | 0.005 |
| Cerebellum Crus2 R - Cerebellum 8 L | 0.164 | < 0.001 | 3.421 | 0.001 |
| Cerebellum Crus2 R - Cerebellum 8 R | 0.187 | < 0.001 | 3.113 | 0.002 |
| Cerebellum Crus2 R - Cerebellum 9 R | 0.197 | < 0.001 | 2.206 | 0.028 |
| Cerebellum Crus2 R - Cerebellum 10 L | 0.084 | < 0.001 | 2.737 | 0.007 |
| Cerebellum Crus2 R - Cerebellum 10 R | 0.101 | < 0.001 | 2.111 | 0.036 |
| Cerebellum Crus2 R - Vermis 8 | 0.213 | < 0.001 | 3.260 | 0.001 |
| Cerebellum Crus2 R - Cerebellum 4 5 L | 0.136 | < 0.001 | 2.842 | 0.005 |
| Cerebellum Crus2 R - Cerebellum 4 5 R | 0.195 | < 0.001 | 3.193 | 0.002 |
| Cerebellum 3 L - Cerebellum 9 L | 0.145 | < 0.001 | -1.988 | 0.048 |
| Cerebellum 4 5 L - Cerebellum 10 L | 0.072 | 0.001 | 2.046 | 0.042 |
| Cerebellum 4 5 L - Cerebellum 10 R | 0.072 | 0.001 | 2.586 | 0.010 |
| Cerebellum 4 5 R - Cerebellum 6 R | 0.250 | < 0.001 | 2.641 | 0.009 |
| Cerebellum 4 5 R - Vermis 1 2 | 0.072 | 0.001 | 2.071 | 0.039 |
| Cerebellum 4 5 R - Cerebellum 6 L | 0.303 | < 0.001 | 1.987 | 0.048 |
| Cerebellum 6 L - Vermis 8 | 0.248 | < 0.001 | 2.350 | 0.020 |
| Cerebellum 6 L - Vermis 9 | 0.183 | < 0.001 | 2.357 | 0.019 |

**Supplementary Table 6**. Frailty on ROI-to-ROI functional connectivity for CU. *P*_FDR_<0.05, covariate: recording site. Regions are presented using the AAL atlas.

| **Regions** | **Model** | | **Frailty** | |
| --- | --- | --- | --- | --- |
|  | **R^2^_adj_** | ***P*_FDR_** | ***t*** | ***P*_FDR_** |
| Precentral L - Frontal Sup Medial R | 0.135 | < 0.001 | 2.059 | 0.040 |
| Precentral L - Cuneus L | 0.147 | < 0.001 | -2.228 | 0.027 |
| Precentral L - Lingual L | 0.070 | < 0.001 | -2.138 | 0.033 |
| Precentral L - Occipital Sup L | 0.057 | 0.001 | -2.159 | 0.032 |
| Precentral L - Occipital Mid L | 0.036 | 0.016 | -2.416 | 0.016 |
| Precentral L - Fusiform L | 0.160 | < 0.001 | -2.650 | 0.008 |
| Precentral L - Fusiform R | 0.171 | < 0.001 | -2.202 | 0.028 |
| Precentral L - Cerebellum 8 L | 0.077 | < 0.001 | -2.272 | 0.024 |
| Precentral L - Vermis 8 | 0.115 | < 0.001 | -2.126 | 0.034 |
| Precentral R - Rolandic Oper R | 0.092 | < 0.001 | -3.259 | 0.001 |
| Precentral R - Cuneus L | 0.068 | < 0.001 | -2.193 | 0.029 |
| Precentral R - Cuneus R | 0.045 | 0.006 | -2.256 | 0.025 |
| Precentral R - Occipital Sup L | 0.040 | 0.010 | -2.409 | 0.017 |
| Precentral R - Occipital Sup R | 0.057 | 0.001 | -2.795 | 0.006 |
| Precentral R - Occipital Mid L | 0.029 | 0.033 | -2.434 | 0.016 |
| Precentral R - Occipital Mid R | 0.036 | 0.017 | -2.506 | 0.013 |
| Precentral R - Occipital Inf R | 0.075 | < 0.001 | -2.036 | 0.043 |
| Precentral R - Fusiform L | 0.111 | < 0.001 | -2.163 | 0.031 |
| Precentral R - SupraMarginal L | 0.047 | 0.005 | -2.059 | 0.040 |
| Precentral R - Temporal Sup R | 0.089 | < 0.001 | -2.023 | 0.044 |
| Precentral R - Vermis 10 | 0.162 | < 0.001 | -2.177 | 0.030 |
| Frontal Sup L - Frontal Mid L | 0.227 | < 0.001 | 2.102 | 0.036 |
| Frontal Sup L - Insula R | 0.092 | < 0.001 | -2.013 | 0.045 |
| Frontal Sup R - Frontal Mid R | 0.208 | < 0.001 | 2.151 | 0.032 |
| Frontal Sup R - Vermis 10 | 0.183 | < 0.001 | -2.208 | 0.028 |
| Frontal Sup Orb L - Supp Motor Area L | 0.164 | < 0.001 | 2.260 | 0.025 |
| Frontal Sup Orb L - Supp Motor Area R | 0.171 | < 0.001 | 2.047 | 0.042 |
| Frontal Sup Orb L - ParaHippocampal L | 0.116 | < 0.001 | 2.273 | 0.024 |
| Frontal Sup Orb L - Cerebellum 3 L | 0.154 | < 0.001 | 1.989 | 0.048 |
| Frontal Sup Orb L - Cerebellum 4 5 L | 0.211 | < 0.001 | 2.162 | 0.031 |
| Frontal Sup Orb L - Vermis 6 | 0.089 | < 0.001 | 2.041 | 0.042 |
| Frontal Sup Orb L - Vermis 9 | 0.090 | < 0.001 | 2.687 | 0.008 |
| Frontal Sup Orb R - Frontal Inf Oper L | 0.107 | < 0.001 | 2.227 | 0.027 |
| Frontal Sup Orb R - Frontal Inf Oper R | 0.115 | < 0.001 | 2.052 | 0.041 |
| Frontal Sup Orb R - Supp Motor Area L | 0.205 | < 0.001 | 2.227 | 0.027 |
| Frontal Sup Orb R - Hippocampus L | 0.254 | < 0.001 | 2.372 | 0.018 |
| Frontal Sup Orb R - Amygdala L | 0.177 | < 0.001 | 2.328 | 0.021 |
| Frontal Sup Orb R - Lingual L | 0.213 | < 0.001 | 1.984 | 0.048 |
| Frontal Sup Orb R - Caudate R | 0.128 | < 0.001 | 2.289 | 0.023 |
| Frontal Sup Orb R - Thalamus R | 0.127 | < 0.001 | 2.030 | 0.043 |
| Frontal Sup Orb R - Heschl L | 0.107 | < 0.001 | 2.584 | 0.010 |
| Frontal Sup Orb R - Cerebellum 4 5 L | 0.242 | < 0.001 | 2.238 | 0.026 |
| Frontal Sup Orb R - Cerebellum 4 5 R | 0.215 | < 0.001 | 2.107 | 0.036 |
| Frontal Mid L - Frontal Sup Medial L | 0.154 | < 0.001 | 2.483 | 0.014 |
| Frontal Mid L - Frontal Sup Medial R | 0.145 | < 0.001 | 2.001 | 0.046 |
| Frontal Mid L - Cerebellum Crus1 L | 0.267 | < 0.001 | -2.364 | 0.019 |
| Frontal Mid L - Cerebellum Crus2 L | 0.289 | < 0.001 | -2.311 | 0.022 |
| Frontal Mid L - Cerebellum 8 L | 0.124 | < 0.001 | -2.321 | 0.021 |
| Frontal Mid L - Cerebellum 10 R | 0.165 | < 0.001 | -2.040 | 0.042 |
| Frontal Mid L - Vermis 7 | 0.090 | < 0.001 | -2.285 | 0.023 |
| Frontal Mid L - Vermis 8 | 0.137 | < 0.001 | -2.950 | 0.003 |
| Frontal Mid L - Vermis 9 | 0.149 | < 0.001 | -2.397 | 0.017 |
| Frontal Mid R - Supp Motor Area R | 0.101 | < 0.001 | 2.442 | 0.015 |
| Frontal Mid R - Frontal Sup Medial L | 0.081 | < 0.001 | 2.802 | 0.005 |
| Frontal Mid R - Frontal Sup Medial R | 0.135 | < 0.001 | 2.676 | 0.008 |
| Frontal Mid R - Cingulum Ant L | 0.044 | 0.007 | 1.970 | 0.050 |
| Frontal Mid R - Cingulum Ant R | 0.073 | < 0.001 | 2.558 | 0.011 |
| Frontal Mid Orb L - Olfactory L | 0.086 | < 0.001 | 2.099 | 0.037 |
| Frontal Mid Orb L - Frontal Sup Medial R | 0.072 | < 0.001 | 2.604 | 0.010 |
| Frontal Mid Orb L - ParaHippocampal L | 0.123 | < 0.001 | 2.796 | 0.006 |
| Frontal Mid Orb L - ParaHippocampal R | 0.125 | < 0.001 | 2.532 | 0.012 |
| Frontal Mid Orb L - Lingual L | 0.174 | < 0.001 | 2.173 | 0.031 |
| Frontal Mid Orb L - Cerebellum 4 5 L | 0.206 | < 0.001 | 2.492 | 0.013 |
| Frontal Mid Orb L - Vermis 1 2 | 0.210 | < 0.001 | 2.682 | 0.008 |
| Frontal Mid Orb L - Vermis 3 | 0.140 | < 0.001 | 2.292 | 0.023 |
| Frontal Mid Orb R - Supp Motor Area R | 0.153 | < 0.001 | 2.121 | 0.035 |
| Frontal Mid Orb R - Frontal Sup Medial R | 0.119 | < 0.001 | 3.596 | < 0.001 |
| Frontal Mid Orb R - ParaHippocampal L | 0.091 | < 0.001 | 3.146 | 0.002 |
| Frontal Mid Orb R - ParaHippocampal R | 0.088 | < 0.001 | 2.108 | 0.036 |
| Frontal Mid Orb R - Amygdala L | 0.227 | < 0.001 | 2.253 | 0.025 |
| Frontal Mid Orb R - Heschl L | 0.064 | 0.001 | 2.280 | 0.023 |
| Frontal Mid Orb R - Cerebellum 3 L | 0.188 | < 0.001 | 2.485 | 0.014 |
| Frontal Mid Orb R - Cerebellum 4 5 L | 0.250 | < 0.001 | 2.776 | 0.006 |
| Frontal Mid Orb R - Cerebellum 6 L | 0.105 | < 0.001 | 2.604 | 0.010 |
| Frontal Mid Orb R - Vermis 1 2 | 0.261 | < 0.001 | 2.075 | 0.039 |
| Frontal Inf Oper L - Postcentral L | 0.055 | 0.002 | 2.195 | 0.029 |
| Frontal Inf Oper L - Cerebellum 4 5 R | 0.107 | < 0.001 | -2.109 | 0.036 |
| Frontal Inf Oper L - Vermis 7 | 0.041 | 0.009 | -2.121 | 0.035 |
| Frontal Inf Oper R - Frontal Sup Medial R | 0.147 | < 0.001 | 2.063 | 0.040 |
| Frontal Inf Oper R - Pallidum L | 0.089 | < 0.001 | -2.039 | 0.042 |
| Frontal Inf Tri L - Cingulum Post R | 0.145 | < 0.001 | 2.260 | 0.025 |
| Frontal Inf Tri L - Cerebellum 4 5 R | 0.110 | < 0.001 | -2.492 | 0.013 |
| Frontal Inf Tri L - Vermis 8 | 0.039 | 0.012 | -1.983 | 0.048 |
| Frontal Inf Tri R - Fusiform R | 0.059 | 0.001 | -2.121 | 0.035 |
| Frontal Inf Tri R - Pallidum L | 0.077 | < 0.001 | -2.076 | 0.039 |
| Frontal Inf Tri R - Temporal Pole Mid L | 0.148 | < 0.001 | 2.547 | 0.011 |
| Frontal Inf Tri R - Cerebellum 4 5 R | 0.045 | 0.006 | -2.106 | 0.036 |
| Frontal Inf Tri R - Cerebellum 10 L | 0.118 | < 0.001 | -2.753 | 0.006 |
| Frontal Inf Tri R - Cerebellum 10 R | 0.174 | < 0.001 | -2.250 | 0.025 |
| Frontal Inf Tri R - Vermis 3 | 0.120 | < 0.001 | -2.138 | 0.033 |
| Frontal Inf Tri R - Vermis 10 | 0.202 | < 0.001 | -2.009 | 0.045 |
| Frontal Inf Orb L - ParaHippocampal L | 0.112 | < 0.001 | 2.139 | 0.033 |
| Frontal Inf Orb L - Vermis 3 | 0.090 | < 0.001 | 2.687 | 0.008 |
| Frontal Inf Orb R - Supp Motor Area R | 0.136 | < 0.001 | 2.279 | 0.023 |
| Frontal Inf Orb R - Hippocampus R | 0.334 | < 0.001 | 2.228 | 0.027 |
| Frontal Inf Orb R - ParaHippocampal L | 0.074 | < 0.001 | 2.471 | 0.014 |
| Frontal Inf Orb R - Cerebellum 6 L | 0.110 | < 0.001 | 2.473 | 0.014 |
| Frontal Inf Orb R - Cerebellum 6 R | 0.091 | < 0.001 | 2.034 | 0.043 |
| Frontal Inf Orb R - Vermis 6 | 0.147 | < 0.001 | 2.236 | 0.026 |
| Rolandic Oper L - Rolandic Oper R | 0.228 | < 0.001 | -2.094 | 0.037 |
| Rolandic Oper L - Cingulum Mid L | 0.048 | 0.004 | -2.888 | 0.004 |
| Rolandic Oper L - Cingulum Mid R | 0.058 | 0.001 | -2.121 | 0.035 |
| Rolandic Oper L - Lingual R | 0.054 | 0.002 | -2.074 | 0.039 |
| Rolandic Oper L - Fusiform L | 0.061 | 0.001 | -2.023 | 0.044 |
| Rolandic Oper L - Precuneus L | 0.111 | < 0.001 | -2.078 | 0.039 |
| Rolandic Oper L - Precuneus R | 0.112 | < 0.001 | -2.194 | 0.029 |
| Rolandic Oper L - Vermis 1 2 | 0.121 | < 0.001 | 2.370 | 0.018 |
| Rolandic Oper R - Insula L | 0.111 | < 0.001 | -2.497 | 0.013 |
| Rolandic Oper R - Insula R | 0.045 | 0.006 | -2.257 | 0.025 |
| Rolandic Oper R - Cingulum Mid L | 0.046 | 0.005 | -2.082 | 0.038 |
| Rolandic Oper R - Lingual R | 0.064 | 0.001 | -2.065 | 0.040 |
| Rolandic Oper R - Postcentral L | 0.035 | 0.018 | -2.003 | 0.046 |
| Rolandic Oper R - SupraMarginal R | 0.157 | < 0.001 | -2.321 | 0.021 |
| Rolandic Oper R - Paracentral Lobule R | 0.065 | 0.001 | -2.532 | 0.012 |
| Rolandic Oper R - Temporal Sup L | 0.090 | < 0.001 | -2.288 | 0.023 |
| Rolandic Oper R - Temporal Sup R | 0.181 | < 0.001 | -2.519 | 0.012 |
| Rolandic Oper R - Cerebellum 4 5 R | 0.031 | 0.027 | -2.254 | 0.025 |
| Rolandic Oper R - Cerebellum 8 L | 0.060 | 0.001 | -2.378 | 0.018 |
| Rolandic Oper R - Cerebellum 10 R | 0.156 | < 0.001 | -2.030 | 0.043 |
| Supp Motor Area L - Calcarine R | 0.047 | 0.005 | -2.121 | 0.035 |
| Supp Motor Area L - Cuneus L | 0.234 | < 0.001 | -2.016 | 0.045 |
| Supp Motor Area L - Lingual R | 0.089 | < 0.001 | -2.076 | 0.039 |
| Supp Motor Area L - Fusiform L | 0.189 | < 0.001 | -2.167 | 0.031 |
| Supp Motor Area R - Frontal Sup Medial L | 0.096 | < 0.001 | 2.104 | 0.036 |
| Supp Motor Area R - Rectus L | 0.232 | < 0.001 | 2.015 | 0.045 |
| Supp Motor Area R - Rectus R | 0.215 | < 0.001 | 2.376 | 0.018 |
| Supp Motor Area R - Insula L | 0.039 | 0.011 | -2.394 | 0.017 |
| Supp Motor Area R - SupraMarginal L | 0.062 | 0.001 | -2.009 | 0.045 |
| Supp Motor Area R - Paracentral Lobule L | 0.212 | < 0.001 | 2.041 | 0.042 |
| Olfactory L - Cerebellum 10 L | 0.098 | < 0.001 | 2.188 | 0.029 |
| Olfactory R - Heschl R | 0.052 | 0.003 | 2.370 | 0.018 |
| Frontal Sup Medial L - Caudate L | 0.183 | < 0.001 | -2.014 | 0.045 |
| Frontal Med Orb L - Vermis 1 2 | 0.154 | < 0.001 | 2.434 | 0.016 |
| Frontal Med Orb R - ParaHippocampal L | 0.053 | 0.002 | 2.071 | 0.039 |
| Frontal Med Orb R - Amygdala L | 0.109 | < 0.001 | 2.593 | 0.010 |
| Frontal Med Orb R - Parietal Sup L | 0.203 | < 0.001 | 2.491 | 0.013 |
| Frontal Med Orb R - Parietal Inf L | 0.195 | < 0.001 | 2.102 | 0.036 |
| Frontal Med Orb R - Caudate L | 0.090 | < 0.001 | -2.131 | 0.034 |
| Frontal Med Orb R - Cerebellum 6 L | 0.119 | < 0.001 | 2.283 | 0.023 |
| Frontal Med Orb R - Cerebellum 8 R | 0.199 | < 0.001 | 2.659 | 0.008 |
| Frontal Med Orb R - Vermis 6 | 0.166 | < 0.001 | 2.804 | 0.005 |
| Rectus L - ParaHippocampal L | 0.135 | < 0.001 | 3.608 | < 0.001 |
| Rectus L - ParaHippocampal R | 0.179 | < 0.001 | 2.251 | 0.025 |
| Rectus L - Amygdala L | 0.143 | < 0.001 | 2.486 | 0.013 |
| Rectus L - Lingual L | 0.194 | < 0.001 | 2.559 | 0.011 |
| Rectus L - Occipital Mid L | 0.164 | < 0.001 | 2.310 | 0.022 |
| Rectus L - Occipital Inf L | 0.230 | < 0.001 | 2.689 | 0.008 |
| Rectus L - Occipital Inf R | 0.189 | < 0.001 | 2.301 | 0.022 |
| Rectus L - Heschl L | 0.076 | < 0.001 | 2.118 | 0.035 |
| Rectus L - Temporal Inf R | 0.141 | < 0.001 | 2.257 | 0.025 |
| Rectus L - Cerebellum 3 L | 0.226 | < 0.001 | 2.827 | 0.005 |
| Rectus L - Cerebellum 4 5 L | 0.222 | < 0.001 | 3.450 | 0.001 |
| Rectus L - Cerebellum 6 L | 0.205 | < 0.001 | 2.454 | 0.015 |
| Rectus L - Cerebellum 6 R | 0.196 | < 0.001 | 2.311 | 0.022 |
| Rectus L - Cerebellum 8 L | 0.235 | < 0.001 | 2.050 | 0.041 |
| Rectus L - Cerebellum 8 R | 0.168 | < 0.001 | 2.128 | 0.034 |
| Rectus L - Cerebellum 10 L | 0.140 | < 0.001 | 2.373 | 0.018 |
| Rectus L - Vermis 6 | 0.175 | < 0.001 | 2.370 | 0.018 |
| Rectus L - Vermis 9 | 0.131 | < 0.001 | 2.010 | 0.045 |
| Rectus R - ParaHippocampal L | 0.109 | < 0.001 | 2.553 | 0.011 |
| Rectus R - ParaHippocampal R | 0.180 | < 0.001 | 3.271 | 0.001 |
| Rectus R - Amygdala L | 0.099 | < 0.001 | 2.351 | 0.019 |
| Rectus R - Occipital Inf L | 0.175 | < 0.001 | 2.253 | 0.025 |
| Rectus R - Occipital Inf R | 0.185 | < 0.001 | 2.505 | 0.013 |
| Rectus R - Fusiform R | 0.164 | < 0.001 | 2.112 | 0.036 |
| Rectus R - Heschl L | 0.088 | < 0.001 | 2.378 | 0.018 |
| Rectus R - Temporal Pole Sup L | 0.218 | < 0.001 | 2.091 | 0.037 |
| Rectus R - Temporal Inf L | 0.104 | < 0.001 | 2.468 | 0.014 |
| Rectus R - Temporal Inf R | 0.141 | < 0.001 | 2.305 | 0.022 |
| Rectus R - Cerebellum Crus1 L | 0.098 | < 0.001 | 2.005 | 0.046 |
| Rectus R - Cerebellum 3 L | 0.171 | < 0.001 | 2.290 | 0.023 |
| Rectus R - Cerebellum 4 5 L | 0.210 | < 0.001 | 2.534 | 0.012 |
| Rectus R - Cerebellum 4 5 R | 0.213 | < 0.001 | 2.492 | 0.013 |
| Rectus R - Cerebellum 6 L | 0.219 | < 0.001 | 2.822 | 0.005 |
| Rectus R - Vermis 8 | 0.071 | < 0.001 | 2.603 | 0.010 |
| Rectus R - Vermis 9 | 0.160 | < 0.001 | 2.070 | 0.039 |
| Insula L - Cingulum Ant L | 0.068 | < 0.001 | -2.016 | 0.045 |
| Insula L - Cingulum Mid L | 0.036 | 0.016 | -2.121 | 0.035 |
| Insula L - Cingulum Mid R | 0.056 | 0.002 | -2.406 | 0.017 |
| Insula L - Cerebellum Crus1 R | 0.243 | < 0.001 | -2.235 | 0.026 |
| Insula R - Cingulum Ant R | 0.096 | < 0.001 | -2.528 | 0.012 |
| Insula R - Cingulum Mid L | 0.045 | 0.006 | -2.525 | 0.012 |
| Insula R - Calcarine L | 0.109 | < 0.001 | -2.157 | 0.032 |
| Insula R - Occipital Inf L | 0.103 | < 0.001 | -2.166 | 0.031 |
| Insula R - Fusiform L | 0.129 | < 0.001 | -2.618 | 0.009 |
| Insula R - Fusiform R | 0.165 | < 0.001 | -2.280 | 0.023 |
| Insula R - Pallidum L | 0.164 | < 0.001 | -2.076 | 0.039 |
| Cingulum Ant R - Hippocampus L | 0.187 | < 0.001 | 2.069 | 0.039 |
| Cingulum Ant R - Cerebellum 8 L | 0.168 | < 0.001 | 2.228 | 0.027 |
| Cingulum Mid L - Cingulum Mid R | 0.419 | < 0.001 | -2.048 | 0.041 |
| Cingulum Mid L - Fusiform L | 0.073 | < 0.001 | -2.131 | 0.034 |
| Cingulum Mid L - Caudate L | 0.147 | < 0.001 | -2.178 | 0.030 |
| Cingulum Mid L - Thalamus L | 0.104 | < 0.001 | -2.185 | 0.030 |
| Cingulum Mid L - Heschl L | 0.089 | < 0.001 | -2.522 | 0.012 |
| Cingulum Mid L - Heschl R | 0.071 | < 0.001 | -3.376 | 0.001 |
| Cingulum Mid L - Cerebellum 8 L | 0.122 | < 0.001 | -2.439 | 0.015 |
| Cingulum Mid L - Vermis 8 | 0.145 | < 0.001 | -3.073 | 0.002 |
| Cingulum Mid R - Heschl R | 0.058 | 0.001 | -2.824 | 0.005 |
| Cingulum Mid R - Temporal Sup R | 0.078 | < 0.001 | -2.076 | 0.039 |
| Cingulum Mid R - Cerebellum 4 5 L | 0.034 | 0.020 | -2.159 | 0.032 |
| Cingulum Mid R - Cerebellum 8 L | 0.137 | < 0.001 | -2.885 | 0.004 |
| Cingulum Mid R - Vermis 8 | 0.137 | < 0.001 | -2.672 | 0.008 |
| Cingulum Post L - Hippocampus L | 0.101 | < 0.001 | -2.421 | 0.016 |
| Cingulum Post L - Cuneus R | 0.157 | < 0.001 | 2.112 | 0.036 |
| Cingulum Post L - SupraMarginal R | 0.093 | < 0.001 | 2.273 | 0.024 |
| Cingulum Post L - Cerebellum 10 R | 0.199 | < 0.001 | -2.045 | 0.042 |
| Cingulum Post R - Putamen R | 0.226 | < 0.001 | 2.153 | 0.032 |
| Cingulum Post R - Cerebellum Crus2 R | 0.088 | < 0.001 | -2.345 | 0.020 |
| Hippocampus L - Precuneus L | 0.128 | < 0.001 | -2.241 | 0.026 |
| Hippocampus R - Precuneus L | 0.131 | < 0.001 | -2.269 | 0.024 |
| Hippocampus R - Cerebellum 4 5 R | 0.134 | < 0.001 | 2.723 | 0.007 |
| Hippocampus R - Vermis 1 2 | 0.086 | < 0.001 | 2.120 | 0.035 |
| Hippocampus R - Vermis 4 5 | 0.112 | < 0.001 | 2.133 | 0.034 |
| ParaHippocampal L - Pallidum L | 0.116 | < 0.001 | 2.053 | 0.041 |
| ParaHippocampal L - Temporal Sup L | 0.126 | < 0.001 | 2.249 | 0.025 |
| ParaHippocampal R - Cerebellum 4 5 L | 0.097 | < 0.001 | 2.491 | 0.013 |
| ParaHippocampal R - Cerebellum 4 5 R | 0.090 | < 0.001 | 2.493 | 0.013 |
| ParaHippocampal R - Cerebellum 10 L | 0.065 | 0.001 | 3.054 | 0.002 |
| ParaHippocampal R - Vermis 4 5 | 0.126 | < 0.001 | 2.398 | 0.017 |
| ParaHippocampal R - Vermis 6 | 0.084 | < 0.001 | 2.301 | 0.022 |
| Amygdala L - Angular L | 0.185 | < 0.001 | 2.465 | 0.014 |
| Amygdala L - Vermis 1 2 | 0.097 | < 0.001 | 2.117 | 0.035 |
| Amygdala R - Cuneus L | 0.057 | 0.001 | -2.056 | 0.041 |
| Amygdala R - Cuneus R | 0.047 | 0.005 | -1.989 | 0.048 |
| Amygdala R - Occipital Sup L | 0.062 | 0.001 | -2.445 | 0.015 |
| Amygdala R - Pallidum L | 0.227 | < 0.001 | 2.020 | 0.044 |
| Amygdala R - Heschl L | 0.093 | < 0.001 | 2.271 | 0.024 |
| Amygdala R - Temporal Sup L | 0.049 | 0.004 | 2.026 | 0.044 |
| Amygdala R - Temporal Inf R | 0.097 | < 0.001 | -2.412 | 0.016 |
| Calcarine L - Occipital Mid L | 0.159 | < 0.001 | 2.491 | 0.013 |
| Calcarine L - Occipital Mid R | 0.121 | < 0.001 | 2.291 | 0.023 |
| Calcarine L - Occipital Inf L | 0.099 | < 0.001 | 2.260 | 0.025 |
| Calcarine L - Postcentral L | 0.066 | 0.001 | -2.099 | 0.037 |
| Calcarine L - Cerebellum 4 5 L | 0.111 | < 0.001 | 2.627 | 0.009 |
| Calcarine L - Cerebellum 6 L | 0.205 | < 0.001 | 2.151 | 0.032 |
| Calcarine L - Vermis 3 | 0.028 | 0.036 | 2.702 | 0.007 |
| Calcarine L - Vermis 4 5 | 0.155 | < 0.001 | 2.253 | 0.025 |
| Calcarine L - Vermis 6 | 0.116 | < 0.001 | 2.248 | 0.025 |
| Calcarine L - Vermis 7 | 0.154 | < 0.001 | 2.070 | 0.039 |
| Calcarine R - Postcentral L | 0.029 | 0.033 | -2.646 | 0.009 |
| Calcarine R - Postcentral R | 0.041 | 0.009 | -2.113 | 0.035 |
| Calcarine R - Parietal Sup L | 0.037 | 0.014 | -2.130 | 0.034 |
| Calcarine R - Cerebellum 4 5 L | 0.141 | < 0.001 | 2.281 | 0.023 |
| Calcarine R - Cerebellum 4 5 R | 0.132 | < 0.001 | 2.068 | 0.040 |
| Calcarine R - Vermis 3 | 0.052 | 0.003 | 2.408 | 0.017 |
| Calcarine R - Vermis 4 5 | 0.099 | < 0.001 | 2.534 | 0.012 |
| Cuneus L - Postcentral L | 0.103 | < 0.001 | -2.787 | 0.006 |
| Cuneus L - Postcentral R | 0.073 | < 0.001 | -2.819 | 0.005 |
| Cuneus L - Parietal Sup L | 0.101 | < 0.001 | -3.098 | 0.002 |
| Cuneus L - Parietal Sup R | 0.108 | < 0.001 | -3.459 | 0.001 |
| Cuneus L - Angular L | 0.108 | < 0.001 | 2.500 | 0.013 |
| Cuneus L - Paracentral Lobule L | 0.183 | < 0.001 | -2.234 | 0.026 |
| Cuneus L - Paracentral Lobule R | 0.171 | < 0.001 | -2.417 | 0.016 |
| Cuneus L - Temporal Pole Sup R | 0.104 | < 0.001 | -2.442 | 0.015 |
| Cuneus L - Vermis 10 | 0.122 | < 0.001 | 2.713 | 0.007 |
| Cuneus R - Occipital Sup L | 0.273 | < 0.001 | -2.049 | 0.041 |
| Cuneus R - Postcentral R | 0.030 | 0.030 | -2.397 | 0.017 |
| Cuneus R - Parietal Sup L | 0.043 | 0.008 | -2.271 | 0.024 |
| Cuneus R - Parietal Sup R | 0.027 | 0.040 | -2.600 | 0.010 |
| Cuneus R - Cerebellum 3 R | 0.128 | < 0.001 | 2.562 | 0.011 |
| Cuneus R - Cerebellum 4 5 L | 0.117 | < 0.001 | 2.059 | 0.040 |
| Cuneus R - Vermis 3 | 0.117 | < 0.001 | 2.662 | 0.008 |
| Cuneus R - Vermis 4 5 | 0.114 | < 0.001 | 2.228 | 0.027 |
| Cuneus R - Vermis 10 | 0.163 | < 0.001 | 2.905 | 0.004 |
| Lingual L - Cerebellum 4 5 L | 0.128 | < 0.001 | 2.305 | 0.022 |
| Lingual L - Cerebellum 4 5 R | 0.107 | < 0.001 | 2.007 | 0.046 |
| Lingual L - Vermis 4 5 | 0.158 | < 0.001 | 2.210 | 0.028 |
| Lingual L - Vermis 6 | 0.185 | < 0.001 | 2.732 | 0.007 |
| Lingual L - Vermis 9 | 0.066 | 0.001 | 2.025 | 0.044 |
| Lingual R - Occipital Sup R | 0.160 | < 0.001 | -2.197 | 0.029 |
| Lingual R - Occipital Inf L | 0.145 | < 0.001 | 1.972 | 0.050 |
| Lingual R - Postcentral L | 0.040 | 0.010 | -2.626 | 0.009 |
| Lingual R - Cerebellum Crus1 R | 0.306 | < 0.001 | 2.173 | 0.031 |
| Lingual R - Cerebellum 4 5 L | 0.094 | < 0.001 | 2.214 | 0.028 |
| Lingual R - Cerebellum 4 5 R | 0.101 | < 0.001 | 2.066 | 0.040 |
| Lingual R - Cerebellum 6 R | 0.335 | < 0.001 | 2.524 | 0.012 |
| Lingual R - Vermis 4 5 | 0.159 | < 0.001 | 2.265 | 0.024 |
| Lingual R - Vermis 6 | 0.166 | < 0.001 | 2.928 | 0.004 |
| Lingual R - Vermis 9 | 0.085 | < 0.001 | 2.306 | 0.022 |
| Lingual R - Vermis 10 | 0.175 | < 0.001 | 2.034 | 0.043 |
| Occipital Sup L - Postcentral R | 0.034 | 0.019 | -2.210 | 0.028 |
| Occipital Sup L - Temporal Pole Sup L | 0.091 | < 0.001 | -2.852 | 0.005 |
| Occipital Sup L - Temporal Pole Mid R | 0.089 | < 0.001 | -2.594 | 0.010 |
| Occipital Sup L - Temporal Inf R | 0.102 | < 0.001 | -2.158 | 0.032 |
| Occipital Sup L - Vermis 10 | 0.185 | < 0.001 | 2.078 | 0.039 |
| Occipital Sup R - Postcentral R | 0.054 | 0.002 | -3.411 | 0.001 |
| Occipital Sup R - Parietal Sup L | 0.073 | < 0.001 | -2.149 | 0.032 |
| Occipital Sup R - Parietal Sup R | 0.063 | 0.001 | -1.979 | 0.049 |
| Occipital Sup R - Paracentral Lobule R | 0.094 | < 0.001 | -2.322 | 0.021 |
| Occipital Sup R - Temporal Pole Sup L | 0.091 | < 0.001 | -2.123 | 0.035 |
| Occipital Sup R - Vermis 1 2 | 0.236 | < 0.001 | 2.069 | 0.039 |
| Occipital Sup R - Vermis 3 | 0.182 | < 0.001 | 2.256 | 0.025 |
| Occipital Sup R - Vermis 4 5 | 0.132 | < 0.001 | 2.030 | 0.043 |
| Occipital Mid L - Postcentral R | 0.025 | 0.048 | -2.719 | 0.007 |
| Occipital Mid L - Parietal Sup L | 0.066 | 0.001 | -2.408 | 0.017 |
| Occipital Mid L - Parietal Sup R | 0.068 | < 0.001 | -2.428 | 0.016 |
| Occipital Mid L - Angular L | 0.063 | 0.001 | 2.008 | 0.046 |
| Occipital Mid L - Temporal Inf R | 0.124 | < 0.001 | -2.029 | 0.043 |
| Occipital Mid L - Cerebellum 3 R | 0.189 | < 0.001 | 2.573 | 0.011 |
| Occipital Mid L - Cerebellum 4 5 L | 0.248 | < 0.001 | 3.787 | < 0.001 |
| Occipital Mid L - Cerebellum 4 5 R | 0.171 | < 0.001 | 3.624 | < 0.001 |
| Occipital Mid L - Cerebellum 6 L | 0.221 | < 0.001 | 2.515 | 0.012 |
| Occipital Mid L - Vermis 3 | 0.113 | < 0.001 | 3.560 | < 0.001 |
| Occipital Mid L - Vermis 4 5 | 0.176 | < 0.001 | 3.667 | < 0.001 |
| Occipital Mid L - Vermis 6 | 0.170 | < 0.001 | 2.090 | 0.037 |
| Occipital Mid L - Vermis 10 | 0.171 | < 0.001 | 2.287 | 0.023 |
| Occipital Mid R - Postcentral R | 0.037 | 0.014 | -2.972 | 0.003 |
| Occipital Mid R - Parietal Sup R | 0.094 | < 0.001 | -2.373 | 0.018 |
| Occipital Mid R - Heschl L | 0.101 | < 0.001 | 1.979 | 0.049 |
| Occipital Mid R - Cerebellum Crus1 R | 0.144 | < 0.001 | 1.979 | 0.049 |
| Occipital Mid R - Cerebellum 4 5 L | 0.145 | < 0.001 | 2.898 | 0.004 |
| Occipital Mid R - Cerebellum 4 5 R | 0.108 | < 0.001 | 2.742 | 0.006 |
| Occipital Mid R - Vermis 3 | 0.087 | < 0.001 | 2.358 | 0.019 |
| Occipital Mid R - Vermis 4 5 | 0.100 | < 0.001 | 2.662 | 0.008 |
| Occipital Inf L - Cerebellum Crus1 L | 0.155 | < 0.001 | 2.157 | 0.032 |
| Occipital Inf L - Cerebellum 4 5 L | 0.192 | < 0.001 | 2.978 | 0.003 |
| Occipital Inf L - Cerebellum 4 5 R | 0.132 | < 0.001 | 2.906 | 0.004 |
| Occipital Inf L - Cerebellum 6 L | 0.212 | < 0.001 | 2.473 | 0.014 |
| Occipital Inf L - Cerebellum 6 R | 0.218 | < 0.001 | 2.419 | 0.016 |
| Occipital Inf L - Vermis 3 | 0.078 | < 0.001 | 2.353 | 0.019 |
| Occipital Inf L - Vermis 4 5 | 0.168 | < 0.001 | 2.100 | 0.037 |
| Occipital Inf L - Vermis 6 | 0.102 | < 0.001 | 2.257 | 0.025 |
| Occipital Inf R - Postcentral L | 0.053 | 0.003 | -2.016 | 0.045 |
| Occipital Inf R - Temporal Mid R | 0.085 | < 0.001 | 2.106 | 0.036 |
| Occipital Inf R - Cerebellum 4 5 L | 0.141 | < 0.001 | 3.047 | 0.003 |
| Occipital Inf R - Cerebellum 4 5 R | 0.169 | < 0.001 | 2.607 | 0.010 |
| Occipital Inf R - Cerebellum 6 R | 0.268 | < 0.001 | 2.043 | 0.042 |
| Occipital Inf R - Vermis 4 5 | 0.195 | < 0.001 | 1.999 | 0.046 |
| Occipital Inf R - Vermis 10 | 0.166 | < 0.001 | 2.299 | 0.022 |
| Fusiform L - Postcentral L | 0.102 | < 0.001 | -2.562 | 0.011 |
| Fusiform L - Postcentral R | 0.097 | < 0.001 | -2.746 | 0.006 |
| Fusiform L - Parietal Sup L | 0.106 | < 0.001 | -2.322 | 0.021 |
| Fusiform L - Parietal Sup R | 0.095 | < 0.001 | -2.389 | 0.018 |
| Fusiform L - Cerebellum 8 L | 0.228 | < 0.001 | -2.326 | 0.021 |
| Fusiform L - Cerebellum 10 L | 0.176 | < 0.001 | 2.048 | 0.041 |
| Fusiform R - Postcentral R | 0.112 | < 0.001 | -2.234 | 0.026 |
| Fusiform R - Paracentral Lobule R | 0.173 | < 0.001 | -2.061 | 0.040 |
| Fusiform R - Pallidum R | 0.079 | < 0.001 | -2.000 | 0.046 |
| Fusiform R - Cerebellum 3 L | 0.109 | < 0.001 | 2.571 | 0.011 |
| Fusiform R - Cerebellum 4 5 L | 0.155 | < 0.001 | 2.699 | 0.007 |
| Fusiform R - Cerebellum 4 5 R | 0.123 | < 0.001 | 2.924 | 0.004 |
| Fusiform R - Cerebellum 6 R | 0.291 | < 0.001 | 2.103 | 0.036 |
| Fusiform R - Cerebellum 10 L | 0.197 | < 0.001 | 2.172 | 0.031 |
| Fusiform R - Vermis 3 | 0.109 | < 0.001 | 3.216 | 0.001 |
| Fusiform R - Vermis 4 5 | 0.163 | < 0.001 | 3.334 | 0.001 |
| Fusiform R - Vermis 6 | 0.119 | < 0.001 | 2.260 | 0.025 |
| Postcentral L - Pallidum R | 0.044 | 0.007 | 2.239 | 0.026 |
| Postcentral L - Temporal Inf R | 0.172 | < 0.001 | -2.116 | 0.035 |
| Postcentral R - Parietal Inf R | 0.147 | < 0.001 | 2.642 | 0.009 |
| Parietal Sup L - Temporal Mid L | 0.079 | < 0.001 | -2.119 | 0.035 |
| Parietal Sup L - Vermis 10 | 0.183 | < 0.001 | -2.100 | 0.037 |
| Parietal Sup R - Caudate L | 0.150 | < 0.001 | 2.430 | 0.016 |
| Parietal Sup R - Putamen R | 0.074 | < 0.001 | 1.971 | 0.050 |
| Parietal Sup R - Temporal Pole Sup L | 0.107 | < 0.001 | -2.361 | 0.019 |
| Parietal Sup R - Temporal Inf R | 0.147 | < 0.001 | -2.000 | 0.046 |
| Parietal Sup R - Cerebellum 6 L | 0.061 | 0.001 | -2.180 | 0.030 |
| Parietal Inf L - Angular L | 0.070 | < 0.001 | 2.013 | 0.045 |
| Parietal Inf L - Temporal Inf R | 0.076 | < 0.001 | -2.018 | 0.044 |
| Parietal Inf L - Vermis 8 | 0.091 | < 0.001 | -2.349 | 0.019 |
| Parietal Inf R - Pallidum L | 0.088 | < 0.001 | -2.199 | 0.029 |
| SupraMarginal L - Temporal Mid R | 0.037 | 0.014 | -2.037 | 0.042 |
| SupraMarginal L - Temporal Inf R | 0.086 | < 0.001 | -2.222 | 0.027 |
| SupraMarginal R - Heschl R | 0.058 | 0.001 | -2.249 | 0.025 |
| SupraMarginal R - Temporal Sup L | 0.074 | < 0.001 | -1.971 | 0.050 |
| SupraMarginal R - Temporal Sup R | 0.218 | < 0.001 | -2.590 | 0.010 |
| SupraMarginal R - Temporal Mid L | 0.054 | 0.002 | -2.000 | 0.046 |
| SupraMarginal R - Temporal Mid R | 0.144 | < 0.001 | -2.008 | 0.046 |
| Angular L - Precuneus R | 0.119 | < 0.001 | 2.000 | 0.046 |
| Angular L - Temporal Pole Sup R | 0.132 | < 0.001 | -2.567 | 0.011 |
| Angular L - Cerebellum Crus2 R | 0.066 | < 0.001 | -2.294 | 0.022 |
| Angular R - Temporal Pole Sup L | 0.129 | < 0.001 | -4.104 | < 0.001 |
| Angular R - Temporal Pole Sup R | 0.127 | < 0.001 | -2.751 | 0.006 |
| Precuneus L - Putamen R | 0.175 | < 0.001 | 2.011 | 0.045 |
| Precuneus L - Vermis 10 | 0.143 | < 0.001 | -2.169 | 0.031 |
| Precuneus R - Temporal Pole Sup L | 0.108 | < 0.001 | -1.989 | 0.048 |
| Caudate R - Temporal Pole Mid L | 0.155 | < 0.001 | 2.081 | 0.038 |
| Caudate R - Cerebellum 8 L | 0.178 | < 0.001 | 2.022 | 0.044 |
| Caudate R - Cerebellum 8 R | 0.262 | < 0.001 | 2.892 | 0.004 |
| Putamen L - Putamen R | 0.363 | < 0.001 | -2.430 | 0.016 |
| Putamen L - Cerebellum 4 5 L | 0.176 | < 0.001 | 2.932 | 0.004 |
| Pallidum L - Cerebellum 3 L | 0.113 | < 0.001 | 2.648 | 0.009 |
| Thalamus L - Cerebellum 4 5 L | 0.152 | < 0.001 | 2.307 | 0.022 |
| Heschl L - Temporal Sup L | 0.134 | < 0.001 | 2.185 | 0.030 |
| Heschl R - Cerebellum 10 R | 0.118 | < 0.001 | 2.684 | 0.008 |
| Temporal Sup L - Cerebellum 4 5 L | 0.108 | < 0.001 | 2.209 | 0.028 |
| Temporal Sup L - Cerebellum 6 L | 0.108 | < 0.001 | 2.372 | 0.018 |
| Temporal Sup R - Vermis 9 | 0.059 | 0.001 | 2.120 | 0.035 |
| Temporal Pole Sup R - Temporal Mid L | 0.143 | < 0.001 | -2.162 | 0.031 |
| Temporal Pole Sup R - Cerebellum 3 R | 0.078 | < 0.001 | 2.016 | 0.045 |
| Temporal Mid L - Cerebellum 4 5 L | 0.149 | < 0.001 | 2.276 | 0.024 |
| Temporal Mid R - Cerebellum Crus1 R | 0.124 | < 0.001 | 2.229 | 0.027 |
| Temporal Mid R - Cerebellum 4 5 L | 0.132 | < 0.001 | 2.500 | 0.013 |
| Temporal Mid R - Cerebellum 4 5 R | 0.083 | < 0.001 | 2.495 | 0.013 |
| Temporal Mid R - Vermis 4 5 | 0.126 | < 0.001 | 2.430 | 0.016 |
| Temporal Mid R - Vermis 6 | 0.102 | < 0.001 | 2.799 | 0.005 |
| Cerebellum Crus1 L - Cerebellum 6 L | 0.218 | < 0.001 | 2.182 | 0.030 |
| Cerebellum Crus1 R - Cerebellum 7b R | 0.180 | < 0.001 | -2.055 | 0.041 |
| Cerebellum 4 5 R - Vermis 4 5 | 0.117 | < 0.001 | 2.960 | 0.003 |
| Cerebellum 4 5 R - Vermis 7 | 0.088 | < 0.001 | 2.470 | 0.014 |
| Vermis 1 2 - Vermis 7 | 0.166 | < 0.001 | -2.144 | 0.033 |
| Vermis 3 - Vermis 6 | 0.062 | 0.001 | 2.003 | 0.046 |
| Vermis 4 5 - Vermis 6 | 0.125 | < 0.001 | 2.025 | 0.044 |
| Vermis 4 5 - Vermis 7 | 0.165 | < 0.001 | 2.317 | 0.021 |
| Vermis 6 - Vermis 7 | 0.134 | < 0.001 | 2.654 | 0.008 |

**Supplementary Table 7**. Frailty on ROI-to-ROI functional connectivity for AD. *P*_FDR_<0.05, covariate: recording site. Regions are presented using the AAL atlas.

| **Regions** | **Model** | | **Frailty** | |
| --- | --- | --- | --- | --- |
|  | **R^2^_adj_** | ***P*_FDR_** | ***t*** | ***P*_FDR_** |
| Precentral L - Vermis 8 | 0.096 | 0.024 | 2.505 | 0.014 |
| Precentral L - Calcarine L | 0.120 | 0.011 | -2.399 | 0.018 |
| Precentral L - Calcarine R | 0.082 | 0.039 | -2.265 | 0.026 |
| Precentral L - Cuneus R | 0.172 | 0.002 | -2.715 | 0.008 |
| Precentral L - Lingual L | 0.160 | 0.002 | -2.791 | 0.006 |
| Precentral L - Lingual R | 0.141 | 0.005 | -2.959 | 0.004 |
| Precentral L - Occipital Sup L | 0.112 | 0.014 | -2.150 | 0.034 |
| Precentral L - Occipital Sup R | 0.162 | 0.002 | -2.733 | 0.007 |
| Precentral L - Occipital Mid L | 0.171 | 0.002 | -3.098 | 0.003 |
| Precentral L - Occipital Mid R | 0.123 | 0.010 | -3.403 | 0.001 |
| Precentral L - Occipital Inf L | 0.140 | 0.005 | -3.043 | 0.003 |
| Precentral L - Occipital Inf R | 0.083 | 0.038 | -2.105 | 0.038 |
| Precentral L - Fusiform L | 0.162 | 0.002 | -2.571 | 0.012 |
| Precentral L - Fusiform R | 0.184 | 0.001 | -2.037 | 0.044 |
| Precentral L - Parietal Sup R | 0.096 | 0.024 | -2.259 | 0.026 |
| Precentral L - Pallidum L | 0.254 | < 0.001 | 2.335 | 0.022 |
| Frontal Mid Orb R - Vermis 3 | 0.201 | 0.001 | 2.480 | 0.015 |
| Frontal Mid Orb R - Occipital Sup R | 0.227 | < 0.001 | 2.199 | 0.030 |
| Frontal Mid Orb R - Postcentral L | 0.202 | 0.001 | 2.610 | 0.010 |
| Frontal Mid Orb R - Thalamus L | 0.231 | < 0.001 | 2.579 | 0.011 |
| Cerebellum 6 R - Cerebellum 8 R | 0.273 | < 0.001 | -2.073 | 0.041 |
| Cerebellum 7b R - Vermis 7 | 0.196 | 0.001 | -2.160 | 0.033 |
| Cerebellum 8 R - Vermis 6 | 0.205 | < 0.001 | -2.302 | 0.023 |
| Cerebellum 8 R - Vermis 7 | 0.230 | < 0.001 | -2.131 | 0.036 |
| Cerebellum 9 L - Vermis 3 | 0.148 | 0.004 | -2.034 | 0.045 |
| Cerebellum 9 R - Vermis 3 | 0.167 | 0.002 | -2.198 | 0.030 |
| Frontal Inf Oper L - Frontal Inf Orb R | 0.235 | < 0.001 | -2.144 | 0.034 |
| Frontal Inf Oper L - Parietal Inf L | 0.095 | 0.025 | -2.419 | 0.017 |
| Frontal Inf Oper L - Temporal Mid L | 0.245 | < 0.001 | -2.485 | 0.015 |
| Frontal Inf Oper L - Temporal Inf L | 0.223 | < 0.001 | -2.965 | 0.004 |
| Frontal Inf Oper L - Temporal Inf R | 0.147 | 0.004 | -2.036 | 0.044 |
| Frontal Inf Oper L - Cerebellum Crus1 R | 0.164 | 0.002 | -2.002 | 0.048 |
| Frontal Inf Oper L - Cerebellum 6 L | 0.150 | 0.004 | -2.215 | 0.029 |
| Frontal Inf Tri L - Frontal Inf Orb R | 0.196 | 0.001 | -2.294 | 0.024 |
| Frontal Inf Tri L - Frontal Med Orb R | 0.161 | 0.002 | -2.229 | 0.028 |
| Frontal Inf Tri L - Parietal Inf L | 0.123 | 0.009 | -2.198 | 0.030 |
| Frontal Inf Tri L - Putamen R | 0.122 | 0.010 | -2.020 | 0.046 |
| Frontal Inf Tri L - Temporal Inf L | 0.178 | 0.001 | -2.391 | 0.019 |
| Frontal Inf Tri L - Cerebellum 6 L | 0.094 | 0.026 | -2.058 | 0.042 |
| Frontal Inf Orb L - Vermis 4 5 | 0.184 | 0.001 | 2.215 | 0.029 |
| Frontal Inf Orb R - Lingual R | 0.193 | 0.001 | 2.289 | 0.024 |
| Frontal Inf Orb R - Thalamus L | 0.176 | 0.001 | 1.999 | 0.048 |
| Frontal Inf Orb R - Thalamus R | 0.188 | 0.001 | 2.155 | 0.034 |
| Rolandic Oper L - Supp Motor Area R | 0.088 | 0.032 | -2.259 | 0.026 |
| Rolandic Oper L - Amygdala R | 0.158 | 0.003 | -2.049 | 0.043 |
| Rolandic Oper L - Heschl L | 0.155 | 0.003 | -2.362 | 0.020 |
| Rolandic Oper L - Temporal Pole Sup R | 0.107 | 0.017 | -3.427 | 0.001 |
| Rolandic Oper R - Vermis 3 | 0.117 | 0.012 | 2.005 | 0.048 |
| Rolandic Oper R - Rectus R | 0.345 | < 0.001 | 2.200 | 0.030 |
| Rolandic Oper R - Cingulum Ant L | 0.081 | 0.041 | -2.402 | 0.018 |
| Rolandic Oper R - Cingulum Mid L | 0.168 | 0.002 | -2.899 | 0.005 |
| Rolandic Oper R - Temporal Pole Sup R | 0.168 | 0.002 | -2.467 | 0.015 |
| Supp Motor Area L - Occipital Sup R | 0.097 | 0.023 | -2.163 | 0.033 |
| Supp Motor Area L - Temporal Sup R | 0.097 | 0.024 | -2.094 | 0.039 |
| Precentral R - Cerebellum 9 L | 0.171 | 0.002 | 2.375 | 0.019 |
| Precentral R - Cerebellum 10 R | 0.270 | < 0.001 | 1.994 | 0.049 |
| Precentral R - Frontal Inf Oper L | 0.086 | 0.034 | -2.969 | 0.004 |
| Precentral R - Frontal Inf Oper R | 0.159 | 0.003 | -2.051 | 0.043 |
| Precentral R - Frontal Inf Tri L | 0.105 | 0.018 | -2.848 | 0.005 |
| Precentral R - Calcarine L | 0.080 | 0.042 | -2.547 | 0.012 |
| Precentral R - Calcarine R | 0.083 | 0.038 | -2.217 | 0.029 |
| Precentral R - Cuneus L | 0.152 | 0.003 | -2.343 | 0.021 |
| Precentral R - Cuneus R | 0.135 | 0.006 | -2.512 | 0.014 |
| Precentral R - Lingual L | 0.084 | 0.037 | -2.259 | 0.026 |
| Precentral R - Lingual R | 0.090 | 0.030 | -2.649 | 0.009 |
| Precentral R - Occipital Sup L | 0.139 | 0.005 | -2.331 | 0.022 |
| Precentral R - Occipital Sup R | 0.112 | 0.014 | -2.521 | 0.013 |
| Precentral R - Occipital Mid L | 0.129 | 0.008 | -2.664 | 0.009 |
| Precentral R - Occipital Inf L | 0.121 | 0.010 | -3.117 | 0.002 |
| Precentral R - Fusiform L | 0.076 | 0.049 | -2.553 | 0.012 |
| Precentral R - Fusiform R | 0.077 | 0.047 | -2.174 | 0.032 |
| Precentral R - Temporal Sup R | 0.090 | 0.030 | -2.103 | 0.038 |
| Precentral R - Cerebellum Crus1 L | 0.141 | 0.005 | 1.996 | 0.049 |
| Supp Motor Area R - Vermis 7 | 0.241 | < 0.001 | 2.317 | 0.023 |
| Supp Motor Area R - Occipital Sup L | 0.114 | 0.013 | -2.154 | 0.034 |
| Supp Motor Area R - SupraMarginal R | 0.100 | 0.021 | -2.087 | 0.039 |
| Supp Motor Area R - Cerebellum Crus1 L | 0.254 | < 0.001 | 2.924 | 0.004 |
| Olfactory L - Vermis 4 5 | 0.225 | < 0.001 | 3.155 | 0.002 |
| Olfactory L - Temporal Pole Sup L | 0.197 | 0.001 | -1.998 | 0.048 |
| Olfactory L - Temporal Pole Sup R | 0.086 | 0.034 | -2.687 | 0.008 |
| Frontal Sup Medial L - Vermis 9 | 0.217 | < 0.001 | 3.121 | 0.002 |
| Frontal Sup Medial L - Cingulum Post L | 0.151 | 0.003 | -2.696 | 0.008 |
| Frontal Sup Medial L - Cingulum Post R | 0.140 | 0.005 | -2.244 | 0.027 |
| Frontal Sup Medial L - Pallidum R | 0.195 | 0.001 | 2.649 | 0.009 |
| Frontal Sup Medial R - Cerebellum 10 L | 0.240 | < 0.001 | 2.169 | 0.032 |
| Frontal Sup Medial R - Vermis 3 | 0.189 | 0.001 | 2.180 | 0.032 |
| Frontal Sup Medial R - Vermis 9 | 0.284 | < 0.001 | 2.309 | 0.023 |
| Frontal Sup Medial R - Temporal Mid R | 0.143 | 0.005 | -2.521 | 0.013 |
| Frontal Med Orb L - Vermis 1 2 | 0.301 | < 0.001 | 2.400 | 0.018 |
| Frontal Med Orb L - Fusiform R | 0.252 | < 0.001 | -2.188 | 0.031 |
| Frontal Med Orb L - Temporal Pole Sup R | 0.132 | 0.007 | -2.027 | 0.045 |
| Frontal Med Orb L - Cerebellum Crus2 L | 0.180 | 0.001 | -2.355 | 0.020 |
| Frontal Med Orb R - Cingulum Post L | 0.128 | 0.008 | -2.177 | 0.032 |
| Frontal Med Orb R - Cerebellum Crus2 L | 0.173 | 0.002 | -2.095 | 0.039 |
| Rectus L - Cerebellum 9 R | 0.354 | < 0.001 | 1.991 | 0.049 |
| Rectus L - Vermis 10 | 0.219 | < 0.001 | 2.480 | 0.015 |
| Rectus L - SupraMarginal L | 0.210 | < 0.001 | 2.011 | 0.047 |
| Rectus L - Temporal Pole Mid R | 0.172 | 0.002 | -2.091 | 0.039 |
| Rectus R - Insula L | 0.380 | < 0.001 | 3.980 | < 0.001 |
| Rectus R - Insula R | 0.330 | < 0.001 | 3.948 | < 0.001 |
| Rectus R - Lingual R | 0.389 | < 0.001 | 2.103 | 0.038 |
| Rectus R - Parietal Inf R | 0.297 | < 0.001 | 2.203 | 0.030 |
| Rectus R - SupraMarginal L | 0.246 | < 0.001 | 2.423 | 0.017 |
| Insula L - Cerebellum 6 R | 0.177 | 0.001 | 2.794 | 0.006 |
| Insula L - Cuneus L | 0.309 | < 0.001 | 2.171 | 0.032 |
| Insula L - Occipital Sup R | 0.316 | < 0.001 | 2.202 | 0.030 |
| Insula L - Fusiform R | 0.293 | < 0.001 | 2.213 | 0.029 |
| Insula L - Parietal Sup L | 0.239 | < 0.001 | 2.376 | 0.019 |
| Insula L - Parietal Sup R | 0.179 | 0.001 | 2.443 | 0.016 |
| Insula L - Parietal Inf R | 0.348 | < 0.001 | 2.039 | 0.044 |
| Frontal Sup L - Vermis 1 2 | 0.237 | < 0.001 | -2.148 | 0.034 |
| Frontal Sup L - Vermis 9 | 0.136 | 0.006 | 1.998 | 0.048 |
| Frontal Sup L - Cingulum Post L | 0.133 | 0.007 | -2.539 | 0.013 |
| Frontal Sup L - Angular L | 0.136 | 0.006 | -2.040 | 0.044 |
| Insula R - Lingual R | 0.245 | < 0.001 | 2.581 | 0.011 |
| Insula R - Fusiform R | 0.239 | < 0.001 | 2.790 | 0.006 |
| Cingulum Ant R - Temporal Sup L | 0.183 | 0.001 | -2.197 | 0.030 |
| Cingulum Mid L - Calcarine L | 0.222 | < 0.001 | -2.706 | 0.008 |
| Cingulum Mid L - Cuneus R | 0.143 | 0.005 | -2.407 | 0.018 |
| Cingulum Mid L - Lingual R | 0.162 | 0.002 | -2.125 | 0.036 |
| Cingulum Mid L - Occipital Sup L | 0.124 | 0.009 | -2.357 | 0.020 |
| Cingulum Mid L - Occipital Sup R | 0.197 | 0.001 | -2.950 | 0.004 |
| Cingulum Mid L - Occipital Mid L | 0.095 | 0.025 | -2.415 | 0.018 |
| Cingulum Mid L - Temporal Sup R | 0.226 | < 0.001 | -2.731 | 0.007 |
| Cingulum Mid L - Temporal Pole Sup R | 0.193 | 0.001 | -2.246 | 0.027 |
| Cingulum Mid L - Cerebellum 4 5 R | 0.086 | 0.034 | -2.073 | 0.041 |
| Cingulum Mid R - Hippocampus L | 0.203 | < 0.001 | 2.157 | 0.033 |
| Cingulum Mid R - Occipital Mid L | 0.133 | 0.007 | -2.310 | 0.023 |
| Cingulum Mid R - Fusiform L | 0.161 | 0.002 | -2.345 | 0.021 |
| Cingulum Mid R - SupraMarginal L | 0.174 | 0.001 | -2.778 | 0.007 |
| Cingulum Mid R - Temporal Sup L | 0.187 | 0.001 | -2.058 | 0.042 |
| Cingulum Mid R - Temporal Sup R | 0.211 | < 0.001 | -2.869 | 0.005 |
| Cingulum Mid R - Temporal Pole Sup L | 0.150 | 0.004 | -2.088 | 0.039 |
| Cingulum Post L - Cerebellum 8 R | 0.304 | < 0.001 | -2.436 | 0.017 |
| Cingulum Post L - Cerebellum 9 R | 0.268 | < 0.001 | -2.791 | 0.006 |
| Cingulum Post L - Fusiform R | 0.140 | 0.005 | -2.059 | 0.042 |
| Cingulum Post L - Cerebellum Crus1 R | 0.311 | < 0.001 | -2.846 | 0.005 |
| Cingulum Post L - Cerebellum Crus2 L | 0.227 | < 0.001 | -2.965 | 0.004 |
| Cingulum Post R - Temporal Pole Mid L | 0.165 | 0.002 | 2.206 | 0.030 |
| Cingulum Post R - Cerebellum Crus1 R | 0.283 | < 0.001 | -2.041 | 0.044 |
| Hippocampus L - Vermis 1 2 | 0.225 | < 0.001 | 2.100 | 0.038 |
| Hippocampus L - SupraMarginal L | 0.183 | 0.001 | 2.458 | 0.016 |
| Hippocampus R - Amygdala L | 0.112 | 0.014 | -2.411 | 0.018 |
| Hippocampus R - Parietal Inf R | 0.181 | 0.001 | 2.108 | 0.038 |
| Hippocampus R - SupraMarginal L | 0.252 | < 0.001 | 2.403 | 0.018 |
| Hippocampus R - Angular R | 0.195 | 0.001 | 2.486 | 0.015 |
| Frontal Sup R - Cingulum Post L | 0.146 | 0.004 | -3.121 | 0.002 |
| Frontal Sup R - Cingulum Post R | 0.154 | 0.003 | -2.910 | 0.004 |
| Frontal Sup R - Hippocampus L | 0.286 | < 0.001 | 2.448 | 0.016 |
| Frontal Sup R - Calcarine L | 0.075 | 0.050 | -2.001 | 0.048 |
| Frontal Sup R - Lingual L | 0.093 | 0.027 | -2.010 | 0.047 |
| Frontal Sup R - Parietal Inf R | 0.090 | 0.030 | -2.001 | 0.048 |
| Frontal Sup R - Angular L | 0.119 | 0.011 | -2.052 | 0.043 |
| Frontal Sup R - Angular R | 0.098 | 0.023 | -2.426 | 0.017 |
| Frontal Sup R - Caudate L | 0.130 | 0.008 | 2.362 | 0.020 |
| Frontal Sup R - Caudate R | 0.107 | 0.017 | 2.171 | 0.032 |
| Frontal Sup R - Pallidum R | 0.208 | < 0.001 | 2.075 | 0.041 |
| Frontal Sup R - Cerebellum 4 5 R | 0.107 | 0.017 | -2.217 | 0.029 |
| ParaHippocampal R - Amygdala R | 0.111 | 0.015 | -2.240 | 0.027 |
| ParaHippocampal R - Caudate R | 0.254 | < 0.001 | 2.159 | 0.033 |
| Amygdala L - Cerebellum 7b R | 0.187 | 0.001 | 2.139 | 0.035 |
| Amygdala L - Angular L | 0.100 | 0.021 | 2.059 | 0.042 |
| Amygdala R - Vermis 9 | 0.120 | 0.011 | 2.016 | 0.047 |
| Amygdala R - Temporal Pole Mid R | 0.156 | 0.003 | -3.052 | 0.003 |
| Calcarine L - Cerebellum 9 R | 0.111 | 0.015 | -2.448 | 0.016 |
| Calcarine L - Postcentral L | 0.166 | 0.002 | -2.259 | 0.026 |
| Calcarine L - Postcentral R | 0.110 | 0.015 | -2.335 | 0.022 |
| Calcarine L - Parietal Sup R | 0.089 | 0.031 | -2.008 | 0.047 |
| Calcarine L - Precuneus L | 0.208 | < 0.001 | -3.212 | 0.002 |
| Calcarine L - Precuneus R | 0.184 | 0.001 | -2.858 | 0.005 |
| Calcarine L - Paracentral Lobule L | 0.124 | 0.009 | -2.524 | 0.013 |
| Calcarine R - Postcentral L | 0.155 | 0.003 | -2.657 | 0.009 |
| Calcarine R - Postcentral R | 0.137 | 0.006 | -2.503 | 0.014 |
| Calcarine R - Precuneus L | 0.132 | 0.007 | -2.112 | 0.037 |
| Calcarine R - Paracentral Lobule L | 0.094 | 0.026 | -2.509 | 0.014 |
| Calcarine R - Paracentral Lobule R | 0.078 | 0.045 | -2.180 | 0.032 |
| Cuneus L - Cerebellum 9 R | 0.178 | 0.001 | -3.249 | 0.002 |
| Cuneus L - Fusiform R | 0.120 | 0.011 | -2.258 | 0.026 |
| Cuneus L - Postcentral L | 0.141 | 0.005 | -2.226 | 0.028 |
| Cuneus L - Postcentral R | 0.100 | 0.022 | -2.229 | 0.028 |
| Cuneus L - Parietal Sup R | 0.100 | 0.022 | -2.292 | 0.024 |
| Cuneus L - Precuneus R | 0.251 | < 0.001 | -2.314 | 0.023 |
| Cuneus L - Paracentral Lobule R | 0.124 | 0.009 | -2.015 | 0.047 |
| Cuneus L - Pallidum L | 0.213 | < 0.001 | 2.482 | 0.015 |
| Cuneus L - Temporal Inf R | 0.093 | 0.027 | -2.261 | 0.026 |
| Cuneus L - Cerebellum Crus1 R | 0.174 | 0.001 | -2.385 | 0.019 |
| Cuneus R - Lingual L | 0.209 | < 0.001 | -2.230 | 0.028 |
| Cuneus R - Fusiform L | 0.135 | 0.006 | -2.059 | 0.042 |
| Cuneus R - Postcentral L | 0.178 | 0.001 | -3.010 | 0.003 |
| Cuneus R - Postcentral R | 0.114 | 0.013 | -2.475 | 0.015 |
| Cuneus R - Paracentral Lobule R | 0.156 | 0.003 | -2.249 | 0.027 |
| Cuneus R - Pallidum R | 0.275 | < 0.001 | 2.810 | 0.006 |
| Lingual L - Cerebellum 8 R | 0.195 | 0.001 | -2.183 | 0.031 |
| Lingual L - Postcentral L | 0.138 | 0.006 | -2.846 | 0.005 |
| Lingual L - Postcentral R | 0.150 | 0.004 | -2.282 | 0.025 |
| Lingual L - Parietal Sup L | 0.165 | 0.002 | -2.013 | 0.047 |
| Lingual L - Parietal Sup R | 0.134 | 0.006 | -2.261 | 0.026 |
| Lingual L - Precuneus L | 0.183 | 0.001 | -2.384 | 0.019 |
| Lingual L - Precuneus R | 0.153 | 0.003 | -2.893 | 0.005 |
| Lingual L - Paracentral Lobule L | 0.119 | 0.011 | -2.247 | 0.027 |
| Lingual L - Thalamus R | 0.164 | 0.002 | -2.054 | 0.043 |
| Lingual R - Postcentral L | 0.130 | 0.007 | -2.987 | 0.004 |
| Lingual R - Precuneus L | 0.141 | 0.005 | -2.037 | 0.044 |
| Lingual R - Precuneus R | 0.153 | 0.003 | -2.336 | 0.021 |
| Lingual R - Paracentral Lobule L | 0.151 | 0.003 | -3.287 | 0.001 |
| Lingual R - Paracentral Lobule R | 0.077 | 0.046 | -2.003 | 0.048 |
| Occipital Sup L - Fusiform R | 0.116 | 0.012 | -2.282 | 0.025 |
| Occipital Sup L - Postcentral L | 0.201 | 0.001 | -2.747 | 0.007 |
| Occipital Sup L - Postcentral R | 0.163 | 0.002 | -2.452 | 0.016 |
| Occipital Sup L - Parietal Sup R | 0.145 | 0.004 | -2.122 | 0.036 |
| Frontal Sup Orb L - Frontal Mid Orb R | 0.184 | 0.001 | 2.146 | 0.034 |
| Frontal Sup Orb L - Parietal Inf R | 0.204 | < 0.001 | 2.035 | 0.044 |
| Frontal Sup Orb L - Thalamus L | 0.232 | < 0.001 | 2.305 | 0.023 |
| Occipital Sup R - Occipital Inf L | 0.166 | 0.002 | -2.034 | 0.045 |
| Occipital Sup R - Fusiform L | 0.177 | 0.001 | -3.154 | 0.002 |
| Occipital Sup R - Fusiform R | 0.138 | 0.006 | -2.318 | 0.022 |
| Occipital Sup R - Postcentral L | 0.163 | 0.002 | -2.160 | 0.033 |
| Occipital Sup R - Postcentral R | 0.102 | 0.020 | -2.084 | 0.040 |
| Occipital Sup R - Angular L | 0.097 | 0.024 | 2.004 | 0.048 |
| Occipital Sup R - Temporal Inf L | 0.107 | 0.017 | -2.617 | 0.010 |
| Occipital Mid L - Occipital Inf R | 0.093 | 0.027 | -2.109 | 0.037 |
| Occipital Mid L - Postcentral L | 0.244 | < 0.001 | -3.473 | 0.001 |
| Occipital Mid L - Postcentral R | 0.183 | 0.001 | -3.085 | 0.003 |
| Occipital Mid L - Parietal Sup L | 0.252 | < 0.001 | -2.675 | 0.009 |
| Occipital Mid L - Parietal Sup R | 0.237 | < 0.001 | -3.843 | < 0.001 |
| Occipital Mid L - Paracentral Lobule L | 0.111 | 0.015 | -2.309 | 0.023 |
| Occipital Mid R - Fusiform L | 0.155 | 0.003 | -2.318 | 0.022 |
| Occipital Mid R - Postcentral L | 0.136 | 0.006 | -2.223 | 0.028 |
| Occipital Mid R - Postcentral R | 0.142 | 0.005 | -2.638 | 0.010 |
| Occipital Mid R - Parietal Sup R | 0.219 | < 0.001 | -2.297 | 0.024 |
| Occipital Mid R - Putamen R | 0.127 | 0.008 | 2.030 | 0.045 |
| Occipital Mid R - Temporal Inf L | 0.123 | 0.009 | -2.510 | 0.014 |
| Occipital Mid R - Temporal Inf R | 0.114 | 0.013 | -2.307 | 0.023 |
| Occipital Inf L - Cerebellum 8 R | 0.183 | 0.001 | -2.094 | 0.039 |
| Occipital Inf L - Postcentral L | 0.138 | 0.006 | -2.447 | 0.016 |
| Occipital Inf L - Postcentral R | 0.089 | 0.031 | -2.247 | 0.027 |
| Occipital Inf L - Paracentral Lobule L | 0.110 | 0.015 | -2.024 | 0.046 |
| Occipital Inf R - Postcentral L | 0.126 | 0.008 | -2.233 | 0.028 |
| Occipital Inf R - Caudate R | 0.174 | 0.001 | 2.828 | 0.006 |
| Occipital Inf R - Cerebellum 3 L | 0.090 | 0.030 | 2.023 | 0.046 |
| Occipital Inf R - Cerebellum 6 L | 0.133 | 0.007 | 2.047 | 0.043 |
| Fusiform L - Fusiform R | 0.465 | < 0.001 | -2.211 | 0.029 |
| Fusiform L - Postcentral L | 0.127 | 0.008 | -2.323 | 0.022 |
| Fusiform L - Thalamus R | 0.251 | < 0.001 | -2.733 | 0.007 |
| Fusiform L - Temporal Pole Mid R | 0.083 | 0.038 | -2.173 | 0.032 |
| Fusiform R - Cerebellum 8 L | 0.141 | 0.005 | -2.167 | 0.033 |
| Fusiform R - Postcentral L | 0.108 | 0.016 | -2.215 | 0.029 |
| Postcentral L - Temporal Sup R | 0.175 | 0.001 | -2.065 | 0.041 |
| Postcentral L - Cerebellum Crus2 L | 0.230 | < 0.001 | 2.390 | 0.019 |
| Postcentral L - Cerebellum Crus2 R | 0.121 | 0.010 | 2.346 | 0.021 |
| Postcentral R - Parietal Inf R | 0.097 | 0.024 | 2.050 | 0.043 |
| Postcentral R - Pallidum L | 0.165 | 0.002 | 2.193 | 0.031 |
| Postcentral R - Temporal Sup R | 0.084 | 0.037 | -2.411 | 0.018 |
| Postcentral R - Temporal Pole Sup L | 0.084 | 0.037 | -2.387 | 0.019 |
| Parietal Sup L - Pallidum R | 0.189 | 0.001 | 1.988 | 0.050 |
| Frontal Sup Orb R - Vermis 10 | 0.413 | < 0.001 | 3.037 | 0.003 |
| Frontal Sup Orb R - Frontal Inf Tri L | 0.238 | < 0.001 | -2.261 | 0.026 |
| Parietal Inf L - Vermis 3 | 0.148 | 0.004 | 2.347 | 0.021 |
| Parietal Inf L - Vermis 8 | 0.108 | 0.016 | 2.497 | 0.014 |
| Parietal Inf L - Temporal Inf R | 0.099 | 0.023 | -2.033 | 0.045 |
| Parietal Inf R - SupraMarginal R | 0.161 | 0.002 | 2.073 | 0.041 |
| Parietal Inf R - Precuneus L | 0.117 | 0.012 | 2.682 | 0.009 |
| Parietal Inf R - Pallidum L | 0.277 | < 0.001 | 2.427 | 0.017 |
| Parietal Inf R - Thalamus R | 0.152 | 0.003 | 2.032 | 0.045 |
| Parietal Inf R - Temporal Mid L | 0.256 | < 0.001 | 2.159 | 0.033 |
| Parietal Inf R - Cerebellum 4 5 L | 0.186 | 0.001 | 2.013 | 0.047 |
| SupraMarginal L - Precuneus L | 0.118 | 0.012 | 2.188 | 0.031 |
| SupraMarginal L - Cerebellum 4 5 L | 0.191 | 0.001 | 2.587 | 0.011 |
| SupraMarginal R - Pallidum L | 0.112 | 0.014 | 2.256 | 0.026 |
| Angular L - Vermis 3 | 0.123 | 0.010 | 2.528 | 0.013 |
| Angular L - Pallidum R | 0.234 | < 0.001 | 2.143 | 0.035 |
| Angular R - Cerebellum 10 L | 0.309 | < 0.001 | 2.372 | 0.020 |
| Angular R - Temporal Inf R | 0.156 | 0.003 | -2.213 | 0.029 |
| Angular R - Cerebellum Crus1 L | 0.225 | < 0.001 | -2.317 | 0.023 |
| Angular R - Cerebellum Crus1 R | 0.199 | 0.001 | -2.326 | 0.022 |
| Precuneus L - Cerebellum 7b R | 0.117 | 0.012 | -1.988 | 0.050 |
| Precuneus L - Cerebellum 9 R | 0.260 | < 0.001 | -2.294 | 0.024 |
| Precuneus L - Pallidum L | 0.163 | 0.002 | 2.877 | 0.005 |
| Precuneus L - Temporal Inf R | 0.077 | 0.047 | -2.177 | 0.032 |
| Precuneus L - Cerebellum Crus1 R | 0.222 | < 0.001 | -1.996 | 0.049 |
| Precuneus R - Cerebellum 8 R | 0.093 | 0.027 | -2.350 | 0.021 |
| Precuneus R - Pallidum L | 0.221 | < 0.001 | 3.417 | 0.001 |
| Precuneus R - Pallidum R | 0.424 | < 0.001 | 2.616 | 0.010 |
| Precuneus R - Cerebellum Crus1 R | 0.176 | 0.001 | -2.438 | 0.017 |
| Frontal Mid L - Cerebellum 7b R | 0.092 | 0.028 | -2.331 | 0.022 |
| Frontal Mid L - Cingulum Post L | 0.198 | 0.001 | -2.888 | 0.005 |
| Frontal Mid L - Cingulum Post R | 0.127 | 0.008 | -2.368 | 0.020 |
| Frontal Mid L - Calcarine L | 0.169 | 0.002 | -2.023 | 0.046 |
| Frontal Mid L - Angular R | 0.086 | 0.035 | -2.291 | 0.024 |
| Frontal Mid L - Heschl R | 0.094 | 0.026 | 2.112 | 0.037 |
| Frontal Mid L - Cerebellum Crus1 R | 0.186 | 0.001 | -2.183 | 0.031 |
| Paracentral Lobule R - Pallidum L | 0.171 | 0.002 | 2.278 | 0.025 |
| Caudate L - Cerebellum 7b R | 0.279 | < 0.001 | 2.200 | 0.030 |
| Caudate L - Cerebellum 8 L | 0.181 | 0.001 | 2.027 | 0.045 |
| Caudate L - Vermis 10 | 0.183 | 0.001 | 2.481 | 0.015 |
| Caudate L - Temporal Pole Mid R | 0.192 | 0.001 | 2.346 | 0.021 |
| Caudate L - Temporal Inf R | 0.183 | 0.001 | 2.157 | 0.033 |
| Caudate R - Vermis 10 | 0.170 | 0.002 | 2.095 | 0.039 |
| Caudate R - Putamen L | 0.223 | < 0.001 | -2.140 | 0.035 |
| Caudate R - Temporal Pole Mid L | 0.174 | 0.001 | 2.173 | 0.032 |
| Caudate R - Temporal Pole Mid R | 0.265 | < 0.001 | 2.460 | 0.016 |
| Putamen L - Cerebellum 10 L | 0.320 | < 0.001 | 2.290 | 0.024 |
| Putamen L - Temporal Pole Sup R | 0.216 | < 0.001 | -2.170 | 0.032 |
| Pallidum L - Thalamus R | 0.239 | < 0.001 | 2.588 | 0.011 |
| Pallidum L - Temporal Sup R | 0.228 | < 0.001 | 2.223 | 0.028 |
| Pallidum L - Temporal Mid R | 0.109 | 0.016 | 2.265 | 0.026 |
| Pallidum R - Temporal Sup R | 0.139 | 0.005 | 2.237 | 0.028 |
| Thalamus R - Vermis 10 | 0.277 | < 0.001 | 2.172 | 0.032 |
| Thalamus R - Heschl R | 0.190 | 0.001 | 2.231 | 0.028 |
| Thalamus R - Temporal Sup R | 0.130 | 0.007 | 2.228 | 0.028 |
| Thalamus R - Cerebellum 6 L | 0.224 | < 0.001 | -2.280 | 0.025 |
| Heschl L - Cerebellum 3 L | 0.107 | 0.017 | -2.555 | 0.012 |
| Frontal Mid R - Cingulum Post R | 0.146 | 0.004 | -2.276 | 0.025 |
| Frontal Mid R - Hippocampus L | 0.165 | 0.002 | 1.999 | 0.048 |
| Frontal Mid R - Thalamus R | 0.094 | 0.026 | 2.272 | 0.025 |
| Temporal Sup L - Cerebellum 6 R | 0.142 | 0.005 | 2.034 | 0.045 |
| Temporal Sup L - Vermis 3 | 0.115 | 0.013 | 1.991 | 0.049 |
| Temporal Sup L - Cerebellum Crus1 L | 0.092 | 0.028 | 2.133 | 0.035 |
| Temporal Sup R - Vermis 3 | 0.229 | < 0.001 | 2.258 | 0.026 |
| Temporal Sup R - Vermis 4 5 | 0.124 | 0.009 | 1.984 | 0.050 |
| Temporal Pole Sup L - Cerebellum 8 L | 0.106 | 0.017 | -2.233 | 0.028 |
| Temporal Pole Sup R - Cerebellum 7b R | 0.144 | 0.004 | 2.065 | 0.041 |
| Temporal Pole Sup R - Vermis 3 | 0.178 | 0.001 | 2.385 | 0.019 |
| Temporal Mid L - Vermis 3 | 0.123 | 0.010 | 2.379 | 0.019 |
| Temporal Mid L - Vermis 4 5 | 0.173 | 0.002 | 2.433 | 0.017 |
| Temporal Mid R - Vermis 3 | 0.213 | < 0.001 | 2.365 | 0.020 |
| Temporal Mid R - Vermis 4 5 | 0.206 | < 0.001 | 2.766 | 0.007 |
| Temporal Inf L - Cerebellum 7b R | 0.082 | 0.040 | -2.122 | 0.036 |
| Temporal Inf L - Cerebellum 8 R | 0.136 | 0.006 | -2.039 | 0.044 |
| Frontal Mid Orb L - Frontal Sup Medial R | 0.231 | < 0.001 | 2.121 | 0.036 |
| Frontal Mid Orb L - Thalamus L | 0.325 | < 0.001 | 2.269 | 0.025 |
| Frontal Mid Orb L - Temporal Pole Sup R | 0.220 | < 0.001 | 2.101 | 0.038 |
| Temporal Inf R - Cerebellum 8 L | 0.203 | < 0.001 | -2.433 | 0.017 |
| Temporal Inf R - Vermis 9 | 0.241 | < 0.001 | 1.998 | 0.048 |
| Cerebellum Crus1 L - Cerebellum 7b R | 0.135 | 0.006 | -2.375 | 0.019 |
| Cerebellum Crus1 L - Cerebellum 10 L | 0.146 | 0.004 | 2.024 | 0.046 |
| Cerebellum Crus1 R - Cerebellum Crus2 L | 0.201 | 0.001 | -2.277 | 0.025 |
| Cerebellum 3 R - Vermis 3 | 0.106 | 0.018 | 2.291 | 0.024 |
| Cerebellum 3 R - Vermis 4 5 | 0.146 | 0.004 | 2.353 | 0.021 |
| Cerebellum 4 5 L - Cerebellum 9 R | 0.122 | 0.010 | -2.229 | 0.028 |
| Cerebellum 4 5 R - Vermis 1 2 | 0.232 | < 0.001 | 4.069 | < 0.001 |
| Cerebellum 6 L - Cerebellum 6 R | 0.449 | < 0.001 | -2.138 | 0.035 |
| Cerebellum 6 L - Cerebellum 7b R | 0.096 | 0.024 | -2.304 | 0.023 |
| Cerebellum 6 L - Cerebellum 8 R | 0.256 | < 0.001 | -2.492 | 0.014 |

**Supplementary Table 8**. Frailty on ROI-to-ROI functional connectivity for FTLD. *P*_FDR_<0.05, covariate: recording site. Regions are presented using the AAL atlas.

| **Regions** | **CU** | **AD** | ***t*** | ***P*_FDR_** |
| --- | --- | --- | --- | --- |
| Precentral L - Cingulum Mid R | 1.435 (0.791) | 0.021 (0.814) | 39.360 | < 0.001 |
| Precentral L - Lingual L | -1.645 (0.815) | -1.215 (0.875) | -11.378 | < 0.001 |
| Precentral L - Lingual R | -1.275 (0.849) | -1.032 (0.844) | -6.431 | < 0.001 |
| Precentral L - Fusiform L | -1.658 (0.835) | -1.499 (0.858) | -4.204 | < 0.001 |
| Precentral L - Temporal Pole Mid R | -1.194 (0.76) | 0.482 (0.872) | -45.823 | < 0.001 |
| Precentral L - Temporal Inf L | -1.334 (0.763) | -0.374 (0.841) | -26.718 | < 0.001 |
| Precentral R - Occipital Inf R | -1.287 (0.784) | -1.139 (0.788) | -4.218 | < 0.001 |
| Precentral R - Parietal Sup R | 1.522 (0.772) | -0.217 (0.779) | 50.162 | < 0.001 |
| Precentral R - Parietal Inf R | 1.189 (0.847) | 0.453 (0.8) | 19.969 | < 0.001 |
| Precentral R - Cerebelum Crus2 L | 1.487 (0.767) | -0.215 (0.9) | 45.518 | < 0.001 |
| Precentral R - Cerebelum Crus2 R | 1.478 (0.756) | -0.408 (0.784) | 54.772 | < 0.001 |
| Frontal Sup L - Frontal Sup R | 1.356 (0.829) | 0.757 (0.849) | 15.970 | < 0.001 |
| Frontal Sup L - Temporal Inf R | -1.432 (0.768) | 0.11 (0.842) | -42.800 | < 0.001 |
| Frontal Sup L - Cerebelum 7b R | -1.275 (0.736) | -0.35 (0.798) | -26.918 | < 0.001 |
| Frontal Sup R - Frontal Sup Orb L | -1.659 (0.746) | 0.297 (0.838) | -55.127 | < 0.001 |
| Frontal Sup R - Olfactory R | -1.415 (0.799) | -0.273 (0.981) | -28.540 | < 0.001 |
| Frontal Sup R - Temporal Inf R | -1.738 (0.785) | 0.083 (0.853) | -49.690 | < 0.001 |
| Frontal Sup Orb L - Frontal Sup Medial L | -1.287 (0.708) | -0.204 (0.868) | -30.600 | < 0.001 |
| Frontal Sup Orb L - Cingulum Ant L | -1.838 (0.719) | 0.078 (0.882) | -53.221 | < 0.001 |
| Frontal Sup Orb L - Cingulum Ant R | -1.334 (0.708) | -0.282 (0.833) | -30.432 | < 0.001 |
| Frontal Sup Orb L - Cuneus L | -1.646 (0.749) | -0.427 (0.85) | -34.005 | < 0.001 |
| Frontal Sup Orb L - Angular L | -1.329 (0.757) | -0.368 (0.809) | -27.428 | < 0.001 |
| Frontal Sup Orb L - Cerebelum 3 L | 2.334 (0.847) | 1.122 (0.898) | 31.023 | < 0.001 |
| Frontal Sup Orb L - Vermis 3 | 1.259 (0.831) | 0.892 (0.854) | 9.718 | < 0.001 |
| Frontal Sup Orb R - Cingulum Ant L | -1.521 (0.751) | 0.335 (0.97) | -47.830 | < 0.001 |
| Frontal Sup Orb R - Cingulum Ant R | -1.301 (0.788) | 0.351 (0.921) | -43.099 | < 0.001 |
| Frontal Sup Orb R - Occipital Inf L | 1.277 (0.861) | 1.075 (0.901) | 5.123 | < 0.001 |
| Frontal Sup Orb R - Cerebelum 3 L | 1.554 (0.811) | 0.714 (0.857) | 22.534 | < 0.001 |
| Frontal Mid L - Precuneus L | -1.256 (0.792) | -0.331 (0.907) | -24.284 | < 0.001 |
| Frontal Mid L - Temporal Mid R | -1.316 (0.822) | -0.429 (0.823) | -24.106 | < 0.001 |
| Frontal Mid L - Temporal Inf L | -1.352 (0.688) | -0.804 (0.945) | -14.819 | < 0.001 |
| Frontal Mid L - Temporal Inf R | -1.364 (0.736) | -0.809 (0.886) | -15.238 | < 0.001 |
| Frontal Mid R - Lingual L | -1.286 (0.76) | 0.073 (0.922) | -35.975 | < 0.001 |
| Frontal Mid R - Fusiform L | -1.601 (0.757) | -0.27 (0.849) | -37.012 | < 0.001 |
| Frontal Mid R - Temporal Inf R | -1.714 (0.718) | -0.29 (0.917) | -38.684 | < 0.001 |
| Frontal Mid R - Cerebelum 6 R | -1.2 (0.783) | 0.179 (0.889) | -36.830 | < 0.001 |
| Frontal Mid Orb L - Cerebelum 3 L | 1.905 (0.813) | 1.036 (0.898) | 22.704 | < 0.001 |
| Frontal Mid Orb L - Vermis 3 | 1.57 (0.872) | 1.269 (0.843) | 7.834 | < 0.001 |
| Frontal Mid Orb L - Vermis 4 5 | 1.383 (0.919) | 1.108 (0.821) | 7.050 | < 0.001 |
| Frontal Mid Orb R - Occipital Mid L | 1.255 (0.808) | 0.121 (0.826) | 31.067 | < 0.001 |
| Frontal Mid Orb R - Occipital Inf L | 1.499 (0.825) | 0.777 (0.956) | 18.068 | < 0.001 |
| Frontal Mid Orb R - Thalamus R | 1.238 (0.733) | 0.687 (0.867) | 15.362 | < 0.001 |
| Frontal Mid Orb R - Cerebelum 10 L | 1.312 (0.887) | 0.165 (0.932) | 28.193 | < 0.001 |
| Frontal Inf Oper L - Rectus R | 1.246 (0.913) | 0.884 (0.814) | 9.364 | < 0.001 |
| Frontal Inf Oper L - Pallidum L | -1.926 (0.708) | -1.003 (0.809) | -27.164 | < 0.001 |
| Frontal Inf Oper L - Temporal Inf L | -1.592 (0.786) | 0.445 (0.833) | -56.233 | < 0.001 |
| Frontal Inf Oper L - Cerebelum 6 R | -1.296 (0.746) | -0.214 (0.821) | -30.861 | < 0.001 |
| Frontal Inf Oper R - Heschl R | 1.219 (0.76) | 0.071 (0.882) | 31.173 | < 0.001 |
| Frontal Inf Oper R - Temporal Inf R | -1.294 (0.744) | -0.401 (0.858) | -24.873 | < 0.001 |
| Frontal Inf Oper R - Cerebelum Crus1 R | 1.465 (0.98) | 0.773 (0.861) | 16.780 | < 0.001 |
| Frontal Inf Oper R - Cerebelum Crus2 R | 1.612 (0.815) | 0.73 (0.845) | 23.761 | < 0.001 |
| Frontal Inf Tri L - Frontal Inf Orb L | -1.574 (0.817) | 0.028 (0.876) | -42.290 | < 0.001 |
| Frontal Inf Tri L - Occipital Inf L | -1.323 (0.743) | -0.607 (0.881) | -19.644 | < 0.001 |
| Frontal Inf Tri L - Occipital Inf R | -1.284 (0.775) | 0.057 (0.972) | -34.109 | < 0.001 |
| Frontal Inf Tri L - Fusiform L | -1.409 (0.747) | -0.119 (0.877) | -35.389 | < 0.001 |
| Frontal Inf Tri L - Fusiform R | -1.556 (0.76) | -0.745 (0.856) | -22.369 | < 0.001 |
| Frontal Inf Tri L - Temporal Mid L | -1.274 (0.767) | 0.013 (0.864) | -35.237 | < 0.001 |
| Frontal Inf Tri L - Temporal Inf L | -2.244 (0.751) | 0.352 (0.884) | -70.782 | < 0.001 |
| Frontal Inf Tri L - Temporal Inf R | -1.704 (0.792) | -0.511 (0.863) | -32.211 | < 0.001 |
| Frontal Inf Tri L - Cerebelum 6 R | -1.37 (0.733) | -0.048 (0.886) | -36.342 | < 0.001 |
| Frontal Inf Tri R - Fusiform L | -1.348 (0.706) | -0.604 (0.916) | -20.329 | < 0.001 |
| Frontal Inf Tri R - Temporal Mid L | -1.26 (0.733) | -0.834 (0.771) | -12.664 | < 0.001 |
| Frontal Inf Tri R - Temporal Inf R | -1.858 (0.729) | 0.033 (0.902) | -51.548 | < 0.001 |
| Frontal Inf Tri R - Cerebelum 6 L | -1.318 (0.828) | -0.01 (0.881) | -34.217 | < 0.001 |
| Frontal Inf Tri R - Cerebelum 6 R | -1.208 (0.85) | -0.489 (0.85) | -18.915 | < 0.001 |
| Frontal Inf Tri R - Vermis 4 5 | 1.294 (0.928) | -0.708 (0.941) | 47.906 | < 0.001 |
| Frontal Inf Orb L - Supp Motor Area R | -1.512 (0.738) | 0.171 (0.803) | -48.833 | < 0.001 |
| Frontal Inf Orb L - Pallidum L | 1.63 (0.903) | -0.419 (0.906) | 50.673 | < 0.001 |
| Frontal Inf Orb L - Heschl L | 1.487 (0.844) | 0.123 (0.826) | 36.533 | < 0.001 |
| Frontal Inf Orb L - Heschl R | 1.445 (0.708) | 0.017 (0.835) | 41.276 | < 0.001 |
| Frontal Inf Orb L - Temporal Sup L | 1.291 (0.791) | 0.054 (0.844) | 33.816 | < 0.001 |
| Frontal Inf Orb R - Frontal Sup Medial R | -1.424 (0.702) | 0.33 (0.877) | -49.349 | < 0.001 |
| Frontal Inf Orb R - Fusiform R | 1.54 (0.848) | 0.503 (0.853) | 27.274 | < 0.001 |
| Frontal Inf Orb R - Heschl L | 1.544 (0.944) | 0.179 (0.757) | 35.675 | < 0.001 |
| Frontal Inf Orb R - Heschl R | 1.464 (0.737) | -0.088 (0.856) | 43.423 | < 0.001 |
| Frontal Inf Orb R - Temporal Pole Mid L | 1.245 (0.818) | 0.967 (0.935) | 7.088 | < 0.001 |
| Frontal Inf Orb R - Cerebelum 3 L | 1.265 (0.793) | 1.045 (0.85) | 5.976 | < 0.001 |
| Frontal Inf Orb R - Cerebelum 4 5 R | 1.485 (0.824) | 0.612 (0.87) | 23.049 | < 0.001 |
| Frontal Inf Orb R - Vermis 3 | 1.782 (0.784) | 0.888 (0.893) | 23.798 | < 0.001 |
| Frontal Inf Orb R - Vermis 4 5 | 1.296 (0.886) | 0.498 (0.871) | 20.318 | < 0.001 |
| Rolandic Oper L - Temporal Pole Mid L | -1.214 (0.777) | 0.223 (0.874) | -38.870 | < 0.001 |
| Rolandic Oper L - Cerebelum 8 L | 1.6 (0.771) | -0.557 (0.902) | 57.503 | < 0.001 |
| Rolandic Oper L - Cerebelum 9 R | 1.385 (0.742) | 0.219 (0.726) | 35.520 | < 0.001 |
| Rolandic Oper R - Parietal Inf R | 1.726 (0.82) | -0.298 (0.781) | 56.524 | < 0.001 |
| Rolandic Oper R - Angular L | 1.415 (0.768) | -0.488 (0.866) | 51.988 | < 0.001 |
| Rolandic Oper R - Angular R | 2.525 (0.847) | -0.251 (0.785) | 76.001 | < 0.001 |
| Rolandic Oper R - Thalamus R | 1.345 (0.856) | -0.574 (0.782) | 52.373 | < 0.001 |
| Supp Motor Area L - Postcentral L | -1.288 (0.771) | -0.104 (0.902) | -31.563 | < 0.001 |
| Supp Motor Area L - Pallidum L | -1.361 (0.725) | -0.752 (0.835) | -17.408 | < 0.001 |
| Supp Motor Area L - Cerebelum 3 L | -1.425 (0.723) | -1.049 (0.889) | -10.365 | < 0.001 |
| Supp Motor Area L - Cerebelum 3 R | -1.516 (0.717) | 0.045 (0.878) | -43.533 | < 0.001 |
| Supp Motor Area L - Cerebelum 4 5 L | -1.312 (0.786) | -0.954 (0.875) | -9.612 | < 0.001 |
| Supp Motor Area L - Cerebelum 6 R | -1.452 (0.791) | -1.033 (0.855) | -11.375 | < 0.001 |
| Supp Motor Area L - Vermis 3 | -1.75 (0.767) | -0.367 (0.902) | -36.925 | < 0.001 |
| Supp Motor Area L - Vermis 6 | -1.558 (0.831) | -0.214 (0.844) | -35.900 | < 0.001 |
| Supp Motor Area R - Cingulum Ant L | -1.311 (0.753) | 0.537 (0.821) | -52.452 | < 0.001 |
| Supp Motor Area R - Lingual L | -1.307 (0.706) | -0.755 (0.881) | -15.479 | < 0.001 |
| Supp Motor Area R - Lingual R | -1.271 (0.743) | -0.506 (0.936) | -20.272 | < 0.001 |
| Supp Motor Area R - Fusiform L | -1.257 (0.737) | -0.84 (0.821) | -11.949 | < 0.001 |
| Supp Motor Area R - Fusiform R | -1.897 (0.73) | -0.545 (0.828) | -38.725 | < 0.001 |
| Supp Motor Area R - Temporal Pole Sup L | -1.299 (0.81) | 0.1 (0.763) | -39.767 | < 0.001 |
| Supp Motor Area R - Temporal Inf R | -1.511 (0.731) | 0.312 (0.834) | -52.010 | < 0.001 |
| Supp Motor Area R - Cerebelum 10 R | -1.258 (0.878) | -0.428 (0.775) | -22.407 | < 0.001 |
| Supp Motor Area R - Vermis 3 | -1.778 (0.761) | 0.471 (0.88) | -61.116 | < 0.001 |
| Olfactory L - Parietal Inf R | 1.511 (0.775) | 0.314 (0.842) | 33.069 | < 0.001 |
| Olfactory L - Caudate R | 1.488 (0.939) | -0.256 (0.891) | 42.589 | < 0.001 |
| Olfactory L - Cerebelum 7b L | 1.44 (0.763) | -0.031 (0.871) | 40.166 | < 0.001 |
| Olfactory R - Frontal Sup Medial R | -1.568 (0.792) | 0.432 (0.931) | -51.745 | < 0.001 |
| Olfactory R - Cingulum Ant L | -1.435 (0.772) | -0.44 (0.875) | -26.951 | < 0.001 |
| Olfactory R - Cingulum Mid L | -1.303 (0.736) | -0.842 (0.863) | -12.859 | < 0.001 |
| Olfactory R - Cerebelum 10 R | -1.26 (0.833) | -0.272 (0.855) | -26.146 | < 0.001 |
| Frontal Sup Medial L - Rectus L | -1.803 (0.689) | -0.239 (0.85) | -45.170 | < 0.001 |
| Frontal Sup Medial L - Rectus R | -1.83 (0.648) | -0.072 (0.878) | -50.962 | < 0.001 |
| Frontal Sup Medial L - Occipital Inf R | -1.201 (0.764) | 0.092 (0.874) | -35.260 | < 0.001 |
| Frontal Sup Medial L - Precuneus L | -1.612 (0.791) | -0.203 (0.885) | -37.561 | < 0.001 |
| Frontal Sup Medial L - Precuneus R | -1.592 (0.706) | 0.57 (0.87) | -61.030 | < 0.001 |
| Frontal Sup Medial L - Temporal Mid L | -1.576 (0.838) | -0.026 (0.774) | -42.978 | < 0.001 |
| Frontal Sup Medial L - Temporal Mid R | -1.686 (0.734) | 0.083 (0.816) | -50.981 | < 0.001 |
| Frontal Sup Medial L - Temporal Inf L | -1.734 (0.699) | -0.127 (0.817) | -47.245 | < 0.001 |
| Frontal Sup Medial L - Temporal Inf R | -1.497 (0.716) | 0.244 (0.826) | -50.344 | < 0.001 |
| Frontal Sup Medial L - Cerebelum Crus2 R | -1.731 (0.833) | -0.486 (0.902) | -32.071 | < 0.001 |
| Frontal Sup Medial R - Rectus R | -1.366 (0.684) | 0.351 (0.91) | -47.693 | < 0.001 |
| Frontal Sup Medial R - Amygdala L | -1.319 (0.787) | 0.975 (0.942) | -59.115 | < 0.001 |
| Frontal Sup Medial R - Cerebelum 7b L | 1.481 (0.673) | 0.465 (0.84) | 29.832 | < 0.001 |
| Frontal Med Orb L - ParaHippocampal L | -1.463 (0.787) | 0.588 (0.897) | -54.369 | < 0.001 |
| Frontal Med Orb L - Amygdala L | -1.497 (0.711) | 0.629 (0.884) | -59.272 | < 0.001 |
| Frontal Med Orb L - Parietal Inf R | 1.193 (0.796) | -0.384 (0.866) | 42.385 | < 0.001 |
| Frontal Med Orb R - Cingulum Ant L | -1.587 (0.817) | -0.936 (0.871) | -17.238 | < 0.001 |
| Frontal Med Orb R - Amygdala L | -1.295 (0.784) | 1.446 (0.929) | -71.290 | < 0.001 |
| Frontal Med Orb R - Parietal Inf R | 1.22 (0.805) | 0.279 (0.879) | 24.977 | < 0.001 |
| Rectus L - Cingulum Ant L | -1.277 (0.773) | -0.174 (1.036) | -26.985 | < 0.001 |
| Rectus L - Cingulum Ant R | -1.279 (0.805) | -0.388 (0.871) | -23.745 | < 0.001 |
| Rectus L - Amygdala L | -1.398 (0.708) | 1.432 (0.968) | -74.644 | < 0.001 |
| Rectus L - Cuneus L | -1.548 (0.787) | -0.122 (0.87) | -38.435 | < 0.001 |
| Rectus L - Occipital Sup L | -1.36 (0.805) | 0.411 (0.845) | -47.993 | < 0.001 |
| Rectus L - Occipital Sup R | -1.257 (0.78) | -0.041 (0.781) | -34.861 | < 0.001 |
| Rectus L - Parietal Inf R | 1.278 (0.747) | 0.284 (0.839) | 28.005 | < 0.001 |
| Rectus L - Temporal Sup R | -1.587 (0.81) | -0.029 (0.867) | -41.497 | < 0.001 |
| Rectus L - Cerebelum 3 L | 1.293 (0.847) | 1.622 (0.866) | -8.600 | < 0.001 |
| Rectus L - Cerebelum 4 5 L | 1.464 (0.791) | 1.892 (0.858) | -11.599 | < 0.001 |
| Rectus L - Cerebelum 4 5 R | 1.895 (0.78) | 0.934 (0.829) | 26.707 | < 0.001 |
| Rectus L - Vermis 4 5 | 1.626 (0.851) | 0.468 (0.82) | 30.988 | < 0.001 |
| Rectus R - Cingulum Ant L | -1.31 (0.7) | 0.095 (1.04) | -35.454 | < 0.001 |
| Rectus R - Cingulum Ant R | -1.83 (0.762) | -0.023 (0.932) | -47.441 | < 0.001 |
| Rectus R - Amygdala L | -1.906 (0.802) | 1.309 (0.882) | -85.273 | < 0.001 |
| Rectus R - Occipital Inf L | 1.947 (0.831) | 1.265 (0.877) | 17.859 | < 0.001 |
| Rectus R - Temporal Sup R | -1.366 (0.785) | 0.304 (0.829) | -46.240 | < 0.001 |
| Rectus R - Cerebelum 3 R | 1.433 (0.777) | 0.892 (0.86) | 14.755 | < 0.001 |
| Rectus R - Cerebelum 4 5 L | 1.493 (0.769) | 1.402 (0.8) | 2.606 | 0.013 |
| Rectus R - Cerebelum 4 5 R | 1.62 (0.805) | 1.433 (0.829) | 5.105 | < 0.001 |
| Rectus R - Cerebelum 6 L | 1.494 (0.745) | 1.658 (0.895) | -4.454 | < 0.001 |
| Rectus R - Cerebelum 6 R | 1.23 (0.752) | 0.991 (0.833) | 6.731 | < 0.001 |
| Rectus R - Vermis 4 5 | 1.517 (0.929) | 0.218 (0.786) | 33.762 | < 0.001 |
| Insula R - Pallidum R | 1.437 (0.797) | 0.236 (0.801) | 33.620 | < 0.001 |
| Insula R - Thalamus R | 1.376 (0.819) | -0.594 (0.803) | 54.336 | < 0.001 |
| Cingulum Ant L - ParaHippocampal L | -1.642 (0.654) | 0.604 (0.833) | -67.068 | < 0.001 |
| Cingulum Ant L - Cerebelum 3 L | -1.323 (0.707) | -0.077 (0.938) | -33.546 | < 0.001 |
| Cingulum Ant L - Vermis 6 | -1.278 (0.719) | 0.505 (0.825) | -51.522 | < 0.001 |
| Cingulum Ant R - Amygdala L | -1.852 (0.708) | 0.637 (0.975) | -65.328 | < 0.001 |
| Cingulum Ant R - Temporal Pole Mid R | -1.545 (0.677) | 0.534 (0.856) | -60.263 | < 0.001 |
| Cingulum Mid L - Vermis 3 | -1.291 (0.666) | -0.469 (0.882) | -23.545 | < 0.001 |
| Cingulum Mid L - Vermis 6 | -1.449 (0.693) | -0.043 (0.804) | -41.888 | < 0.001 |
| Cingulum Mid L - Vermis 7 | -1.23 (0.763) | -0.255 (0.834) | -27.287 | < 0.001 |
| Cingulum Mid R - Fusiform L | -1.403 (0.708) | -1.111 (0.789) | -8.694 | < 0.001 |
| Cingulum Mid R - Fusiform R | -1.471 (0.696) | -0.723 (0.804) | -22.242 | < 0.001 |
| Cingulum Mid R - Cerebelum Crus2 R | 1.537 (0.771) | -0.335 (0.879) | 50.601 | < 0.001 |
| Cingulum Mid R - Cerebelum 10 R | -1.24 (0.692) | 0.238 (0.835) | -43.079 | < 0.001 |
| Cingulum Mid R - Vermis 3 | -1.396 (0.673) | -0.993 (0.897) | -11.373 | < 0.001 |
| Cingulum Mid R - Vermis 6 | -1.429 (0.705) | -0.556 (0.79) | -26.064 | < 0.001 |
| Cingulum Post L - Hippocampus L | 1.332 (0.761) | -1.339 (0.951) | 69.328 | < 0.001 |
| Cingulum Post L - Heschl R | 1.285 (0.839) | -0.012 (0.792) | 35.555 | < 0.001 |
| Cingulum Post L - Vermis 4 5 | 1.394 (0.839) | -0.738 (0.905) | 54.648 | < 0.001 |
| Cingulum Post R - ParaHippocampal R | 1.376 (0.832) | -0.767 (0.887) | 55.704 | < 0.001 |
| Cingulum Post R - Heschl R | 1.533 (0.819) | 0.092 (0.792) | 40.008 | < 0.001 |
| Hippocampus L - Postcentral L | -1.526 (0.645) | -0.215 (0.914) | -37.053 | < 0.001 |
| Hippocampus L - Angular L | 1.273 (0.815) | 0.418 (0.972) | 21.321 | < 0.001 |
| Hippocampus L - Cerebelum 4 5 L | 1.66 (0.739) | 0.442 (0.971) | 31.558 | < 0.001 |
| Hippocampus L - Cerebelum 6 R | 1.395 (0.737) | 0.254 (0.867) | 31.688 | < 0.001 |
| Hippocampus R - Thalamus L | 1.255 (0.775) | 0.156 (0.946) | 28.430 | < 0.001 |
| Hippocampus R - Temporal Mid L | 1.76 (0.799) | 0.147 (0.84) | 43.994 | < 0.001 |
| Hippocampus R - Temporal Mid R | 1.787 (0.875) | -0.153 (0.898) | 48.905 | < 0.001 |
| Hippocampus R - Cerebelum 6 R | 1.257 (0.775) | 0.317 (0.858) | 25.723 | < 0.001 |
| Hippocampus R - Vermis 4 5 | 1.315 (0.858) | 1.158 (0.887) | 4.022 | < 0.001 |
| ParaHippocampal L - ParaHippocampal R | 1.524 (0.714) | 0.17 (0.939) | 36.294 | < 0.001 |
| ParaHippocampal L - Fusiform R | 1.835 (0.766) | 0.211 (0.854) | 44.752 | < 0.001 |
| ParaHippocampal L - Parietal Inf R | 1.536 (0.857) | -0.136 (0.737) | 46.767 | < 0.001 |
| ParaHippocampal L - Temporal Pole Mid L | 1.308 (0.802) | 0.408 (0.89) | 23.738 | < 0.001 |
| ParaHippocampal L - Temporal Pole Mid R | 1.417 (0.79) | 0.176 (0.901) | 32.749 | < 0.001 |
| ParaHippocampal L - Temporal Inf L | 1.324 (0.743) | 0.091 (0.885) | 33.741 | < 0.001 |
| ParaHippocampal L - Temporal Inf R | 1.416 (0.733) | 0.068 (0.852) | 37.920 | < 0.001 |
| ParaHippocampal L - Cerebelum Crus2 R | 1.462 (0.87) | 0.379 (0.907) | 27.270 | < 0.001 |
| ParaHippocampal L - Cerebelum 6 R | 1.528 (0.733) | 0.245 (0.793) | 37.580 | < 0.001 |
| ParaHippocampal R - Occipital Inf L | 1.287 (0.793) | 0.209 (0.828) | 29.746 | < 0.001 |
| ParaHippocampal R - Fusiform L | 2.084 (0.733) | -0.009 (0.933) | 55.776 | < 0.001 |
| ParaHippocampal R - Fusiform R | 2 (0.773) | 0.945 (0.836) | 29.306 | < 0.001 |
| ParaHippocampal R - Parietal Sup R | 1.228 (0.784) | 0.251 (0.862) | 26.534 | < 0.001 |
| ParaHippocampal R - Thalamus R | 1.395 (0.807) | -0.372 (1.051) | 42.178 | < 0.001 |
| ParaHippocampal R - Temporal Mid L | 1.587 (0.791) | 0.44 (0.845) | 31.328 | < 0.001 |
| ParaHippocampal R - Temporal Mid R | 1.609 (0.754) | 0.405 (0.845) | 33.583 | < 0.001 |
| ParaHippocampal R - Temporal Pole Mid L | 1.615 (0.746) | 0.279 (0.822) | 38.071 | < 0.001 |
| ParaHippocampal R - Temporal Pole Mid R | 1.286 (0.74) | 1.056 (0.85) | 6.452 | < 0.001 |
| ParaHippocampal R - Temporal Inf L | 2.18 (0.725) | 0.544 (0.867) | 45.767 | < 0.001 |
| ParaHippocampal R - Temporal Inf R | 2.01 (0.746) | 0.468 (0.886) | 42.087 | < 0.001 |
| ParaHippocampal R - Cerebelum 3 L | 1.402 (0.893) | 1.049 (0.904) | 8.791 | < 0.001 |
| ParaHippocampal R - Cerebelum 4 5 R | 1.661 (0.737) | 1.371 (0.905) | 7.863 | < 0.001 |
| ParaHippocampal R - Cerebelum 6 R | 1.405 (0.776) | 0.498 (0.855) | 24.859 | < 0.001 |
| Amygdala L - Vermis 6 | -1.28 (0.845) | 0.368 (0.831) | -43.955 | < 0.001 |
| Amygdala L - Vermis 10 | 1.375 (0.992) | 0.293 (0.831) | 26.441 | < 0.001 |
| Calcarine L - Thalamus R | 1.322 (0.837) | 0.361 (0.859) | 25.307 | < 0.001 |
| Calcarine L - Heschl R | 1.59 (0.779) | -0.376 (0.762) | 57.026 | < 0.001 |
| Calcarine R - Pallidum L | 1.746 (0.778) | -0.25 (0.829) | 55.551 | < 0.001 |
| Calcarine R - Thalamus R | 1.518 (0.82) | -0.001 (0.847) | 40.777 | < 0.001 |
| Calcarine R - Heschl R | 1.905 (0.817) | -0.217 (0.806) | 58.475 | < 0.001 |
| Calcarine R - Vermis 4 5 | 1.708 (0.786) | 1.405 (0.891) | 8.069 | < 0.001 |
| Cuneus L - Occipital Inf R | -1.719 (0.757) | -1.079 (0.778) | -18.665 | < 0.001 |
| Cuneus L - Fusiform L | -1.537 (0.81) | -0.812 (0.851) | -19.520 | < 0.001 |
| Cuneus L - Fusiform R | -1.36 (0.772) | -0.229 (0.842) | -31.327 | < 0.001 |
| Cuneus L - Temporal Inf L | -1.643 (0.832) | -0.39 (0.85) | -33.290 | < 0.001 |
| Cuneus L - Temporal Inf R | -1.554 (0.794) | -1.019 (0.907) | -14.035 | < 0.001 |
| Cuneus L - Cerebelum 3 L | -1.273 (0.659) | -0.229 (0.956) | -28.406 | < 0.001 |
| Cuneus L - Vermis 7 | -1.555 (0.832) | -0.001 (0.811) | -42.265 | < 0.001 |
| Cuneus R - Fusiform L | -1.526 (0.856) | -0.681 (0.828) | -22.448 | < 0.001 |
| Cuneus R - Fusiform R | -1.2 (0.807) | -0.462 (0.819) | -20.303 | < 0.001 |
| Cuneus R - Pallidum R | 1.472 (0.805) | -0.133 (0.813) | 44.383 | < 0.001 |
| Cuneus R - Thalamus R | 1.981 (0.818) | 0.312 (0.84) | 45.001 | < 0.001 |
| Cuneus R - Temporal Inf L | -1.347 (0.779) | -0.138 (0.841) | -33.344 | < 0.001 |
| Cuneus R - Temporal Inf R | -1.518 (0.745) | -0.826 (0.874) | -19.053 | < 0.001 |
| Lingual L - Paracentral Lobule L | -1.247 (0.687) | -0.491 (0.829) | -22.199 | < 0.001 |
| Lingual L - Heschl R | 1.441 (0.853) | 0.171 (0.801) | 34.315 | < 0.001 |
| Lingual L - Cerebelum 3 L | 1.25 (0.802) | 0.406 (0.962) | 21.300 | < 0.001 |
| Lingual L - Cerebelum 4 5 L | 1.704 (0.783) | 1.234 (0.972) | 11.918 | < 0.001 |
| Lingual L - Cerebelum 4 5 R | 1.494 (0.825) | 1.102 (0.925) | 10.023 | < 0.001 |
| Lingual L - Vermis 4 5 | 1.378 (0.835) | 1.213 (0.96) | 4.081 | < 0.001 |
| Lingual L - Vermis 8 | 1.52 (0.734) | 0.018 (0.878) | 41.514 | < 0.001 |
| Lingual R - Paracentral Lobule L | -1.358 (0.756) | -0.503 (0.934) | -22.509 | < 0.001 |
| Lingual R - Paracentral Lobule R | -1.271 (0.77) | -0.62 (0.812) | -18.385 | < 0.001 |
| Lingual R - Heschl R | 1.712 (0.825) | 0.372 (0.79) | 37.089 | < 0.001 |
| Lingual R - Temporal Pole Sup R | 1.323 (0.843) | -0.512 (0.898) | 47.115 | < 0.001 |
| Lingual R - Cerebelum 4 5 R | 1.245 (0.83) | 1.141 (0.897) | 2.667 | 0.011 |
| Lingual R - Vermis 8 | 1.545 (0.734) | 0.162 (0.899) | 37.703 | < 0.001 |
| Occipital Sup L - Fusiform L | -1.283 (0.821) | -0.555 (0.85) | -19.488 | < 0.001 |
| Occipital Sup L - Fusiform R | -1.259 (0.792) | -0.802 (0.852) | -12.434 | < 0.001 |
| Occipital Sup L - Angular R | 1.484 (0.773) | 0.736 (0.78) | 21.523 | < 0.001 |
| Occipital Sup L - Pallidum R | 1.304 (0.69) | -0.412 (0.858) | 49.257 | < 0.001 |
| Occipital Sup L - Thalamus R | 1.643 (0.904) | 0.262 (0.905) | 34.144 | < 0.001 |
| Occipital Sup L - Temporal Inf R | -1.402 (0.792) | -1.233 (0.906) | -4.454 | < 0.001 |
| Occipital Sup L - Vermis 4 5 | 1.252 (0.867) | 0.776 (0.963) | 11.607 | < 0.001 |
| Occipital Sup R - Pallidum R | 1.35 (0.692) | 0.418 (0.881) | 26.322 | < 0.001 |
| Occipital Sup R - Thalamus R | 1.52 (0.806) | 0.411 (0.846) | 30.027 | < 0.001 |
| Occipital Mid L - Caudate L | 1.473 (0.83) | 0.78 (0.818) | 18.828 | < 0.001 |
| Occipital Mid L - Pallidum L | 1.409 (0.757) | -0.003 (0.81) | 40.289 | < 0.001 |
| Occipital Mid L - Thalamus R | 1.515 (0.861) | 0.597 (0.896) | 23.369 | < 0.001 |
| Occipital Mid L - Heschl R | 1.372 (0.843) | 0.384 (0.731) | 28.008 | < 0.001 |
| Occipital Mid L - Temporal Inf R | -1.471 (0.785) | -1.164 (0.825) | -8.535 | < 0.001 |
| Occipital Mid L - Vermis 4 5 | 1.369 (0.829) | 2.015 (0.966) | -16.057 | < 0.001 |
| Occipital Mid R - Thalamus R | 1.577 (0.887) | 0.205 (0.886) | 34.621 | < 0.001 |
| Occipital Mid R - Heschl R | 1.358 (0.842) | 0.311 (0.883) | 27.131 | < 0.001 |
| Occipital Mid R - Temporal Inf R | -1.304 (0.72) | -0.859 (0.855) | -12.582 | < 0.001 |
| Occipital Mid R - Vermis 9 | 1.235 (0.839) | 0.771 (0.845) | 12.314 | < 0.001 |
| Occipital Inf L - Cerebelum Crus2 R | 1.459 (0.756) | 0.57 (0.896) | 23.970 | < 0.001 |
| Occipital Inf L - Cerebelum 4 5 L | 2.035 (0.788) | 1.589 (0.972) | 11.269 | < 0.001 |
| Occipital Inf L - Cerebelum 4 5 R | 1.47 (0.776) | 1.589 (0.926) | -3.114 | 0.003 |
| Occipital Inf L - Vermis 4 5 | 1.386 (0.75) | 1.141 (0.857) | 6.819 | < 0.001 |
| Occipital Inf L - Vermis 6 | 1.747 (0.747) | 1.273 (0.903) | 12.782 | < 0.001 |
| Occipital Inf L - Vermis 8 | 1.554 (0.728) | 0.074 (0.87) | 41.242 | < 0.001 |
| Occipital Inf R - Postcentral L | -1.251 (0.807) | -1.113 (0.786) | -3.866 | < 0.001 |
| Occipital Inf R - Paracentral Lobule R | -1.458 (0.783) | -0.408 (0.769) | -30.265 | < 0.001 |
| Fusiform L - Paracentral Lobule L | -1.439 (0.732) | -0.815 (0.833) | -17.806 | < 0.001 |
| Fusiform L - Cerebelum Crus2 L | 1.379 (0.685) | -0.067 (0.923) | 39.780 | < 0.001 |
| Fusiform L - Cerebelum Crus2 R | 1.647 (0.779) | -0.665 (0.875) | 62.422 | < 0.001 |
| Fusiform L - Cerebelum 3 L | 1.292 (0.783) | 0.268 (0.92) | 26.817 | < 0.001 |
| Fusiform L - Cerebelum 4 5 L | 1.365 (0.763) | 0.61 (0.949) | 19.599 | < 0.001 |
| Fusiform L - Cerebelum 4 5 R | 1.759 (0.783) | 0.34 (0.895) | 37.737 | < 0.001 |
| Fusiform L - Cerebelum 6 L | 1.381 (0.813) | 0.717 (0.881) | 17.519 | < 0.001 |
| Fusiform L - Cerebelum 6 R | 1.22 (0.793) | 0.127 (0.894) | 28.934 | < 0.001 |
| Fusiform L - Vermis 4 5 | 1.82 (0.805) | 0.87 (0.917) | 24.629 | < 0.001 |
| Fusiform L - Vermis 6 | 1.782 (0.775) | 0.713 (0.902) | 28.416 | < 0.001 |
| Fusiform L - Vermis 8 | 1.531 (0.71) | -0.201 (0.836) | 49.938 | < 0.001 |
| Fusiform R - Paracentral Lobule L | -1.53 (0.784) | -0.431 (0.794) | -31.122 | < 0.001 |
| Fusiform R - Heschl R | 1.231 (0.685) | 0.051 (1.024) | 30.277 | < 0.001 |
| Fusiform R - Temporal Pole Sup R | 1.534 (0.803) | 0.001 (0.806) | 42.605 | < 0.001 |
| Fusiform R - Temporal Pole Mid R | 1.351 (0.792) | 0.112 (0.835) | 34.041 | < 0.001 |
| Fusiform R - Cerebelum Crus2 R | 1.507 (0.761) | 0.736 (0.8) | 22.077 | < 0.001 |
| Fusiform R - Cerebelum 4 5 R | 1.92 (0.834) | 1.643 (0.844) | 7.386 | < 0.001 |
| Fusiform R - Vermis 6 | 1.441 (0.784) | 1.29 (0.861) | 4.108 | < 0.001 |
| Fusiform R - Vermis 8 | 1.681 (0.737) | 0.281 (0.829) | 39.950 | < 0.001 |
| Fusiform R - Vermis 9 | 1.328 (0.776) | 0.732 (0.856) | 16.324 | < 0.001 |
| Postcentral L - Parietal Sup L | 1.357 (0.757) | -0.27 (0.881) | 44.279 | < 0.001 |
| Postcentral L - Pallidum L | -1.329 (0.708) | 0.408 (0.772) | -52.442 | < 0.001 |
| Postcentral L - Temporal Pole Mid L | -1.202 (0.73) | -0.919 (0.744) | -8.584 | < 0.001 |
| Postcentral L - Cerebelum Crus2 R | 1.314 (0.797) | -0.191 (0.768) | 43.006 | < 0.001 |
| Postcentral L - Cerebelum 9 L | 1.5 (0.759) | 0.065 (0.883) | 38.975 | < 0.001 |
| Postcentral R - Parietal Sup R | 1.535 (0.781) | 0.185 (0.789) | 38.447 | < 0.001 |
| Parietal Sup L - Precuneus L | 1.254 (0.748) | 0.807 (0.807) | 12.856 | < 0.001 |
| Parietal Sup L - Caudate L | 2.314 (0.78) | 0.745 (0.941) | 40.601 | < 0.001 |
| Parietal Sup L - Caudate R | 1.892 (0.785) | 0.757 (0.932) | 29.454 | < 0.001 |
| Parietal Sup L - Heschl R | 1.941 (0.852) | -0.185 (0.844) | 56.060 | < 0.001 |
| Parietal Sup L - Temporal Sup L | 1.411 (0.753) | -0.131 (0.806) | 44.231 | < 0.001 |
| Parietal Sup R - Caudate L | 1.878 (0.844) | 1.393 (0.863) | 12.697 | < 0.001 |
| Parietal Sup R - Caudate R | 1.533 (0.866) | 0.973 (0.918) | 14.036 | < 0.001 |
| Parietal Sup R - Heschl R | 1.489 (0.787) | 0.135 (0.903) | 35.745 | < 0.001 |
| Parietal Sup R - Vermis 8 | 1.702 (0.786) | -0.878 (0.856) | 70.222 | < 0.001 |
| Parietal Inf L - Heschl R | 1.377 (0.755) | -0.744 (0.743) | 63.322 | < 0.001 |
| Parietal Inf L - Cerebelum 6 R | -1.662 (0.791) | -0.287 (0.762) | -39.578 | < 0.001 |
| Parietal Inf L - Cerebelum 7b R | -1.168 (0.834) | 0.371 (0.856) | -40.712 | < 0.001 |
| Parietal Inf L - Vermis 1 2 | -1.401 (0.805) | 0.089 (0.877) | -39.571 | < 0.001 |
| Parietal Inf R - Heschl R | 2.006 (0.827) | -0.332 (0.782) | 64.951 | < 0.001 |
| Parietal Inf R - Vermis 1 2 | -1.308 (0.766) | 0.105 (0.917) | -37.401 | < 0.001 |
| Angular L - Temporal Pole Sup R | 1.452 (0.791) | -1.485 (0.821) | 81.463 | < 0.001 |
| Angular R - Heschl R | 1.345 (0.862) | -0.224 (0.78) | 42.691 | < 0.001 |
| Angular R - Temporal Sup R | 1.199 (0.806) | -0.104 (0.885) | 34.398 | < 0.001 |
| Angular R - Vermis 7 | -1.359 (0.82) | -0.016 (0.843) | -36.111 | < 0.001 |
| Precuneus L - Cerebelum 7b R | -1.249 (0.816) | -0.039 (0.809) | -33.299 | < 0.001 |
| Precuneus R - Vermis 8 | 1.207 (0.786) | -0.735 (0.798) | 54.830 | < 0.001 |
| Paracentral Lobule L - Temporal Inf R | -1.485 (0.722) | -0.325 (0.832) | -33.317 | < 0.001 |
| Paracentral Lobule L - Cerebelum 10 L | 1.323 (0.82) | -0.128 (0.798) | 40.124 | < 0.001 |
| Caudate L - Heschl R | 1.752 (0.925) | -0.482 (0.892) | 54.960 | < 0.001 |
| Caudate L - Temporal Sup L | 1.555 (0.824) | -0.841 (0.876) | 63.018 | < 0.001 |
| Caudate L - Cerebelum 4 5 R | 1.557 (0.815) | 0.144 (0.985) | 34.965 | < 0.001 |
| Caudate R - Heschl R | 1.708 (0.848) | -0.018 (0.873) | 44.855 | < 0.001 |
| Caudate R - Temporal Sup L | 1.313 (0.798) | 0.027 (0.854) | 34.781 | < 0.001 |
| Caudate R - Cerebelum 10 R | -1.41 (0.817) | 0.073 (0.79) | -41.259 | < 0.001 |
| Putamen L - Thalamus L | 1.431 (0.77) | 0.017 (0.834) | 39.427 | < 0.001 |
| Putamen L - Heschl R | 1.234 (0.805) | 0.272 (0.778) | 27.171 | < 0.001 |
| Putamen L - Cerebelum 4 5 L | -1.655 (0.783) | 1.567 (0.867) | -87.194 | < 0.001 |
| Putamen L - Cerebelum 7b R | -1.319 (0.79) | 0.202 (0.869) | -40.967 | < 0.001 |
| Putamen L - Cerebelum 8 L | -1.568 (0.876) | 0.644 (0.77) | -60.001 | < 0.001 |
| Putamen R - Thalamus L | 1.281 (0.765) | 0.317 (0.866) | 26.376 | < 0.001 |
| Putamen R - Thalamus R | 1.279 (0.755) | 0.378 (0.883) | 24.513 | < 0.001 |
| Putamen R - Heschl R | 1.327 (0.756) | -0.61 (0.856) | 53.615 | < 0.001 |
| Putamen R - Cerebelum 3 R | -1.378 (0.79) | 0.26 (0.881) | -43.773 | < 0.001 |
| Putamen R - Vermis 6 | -1.324 (0.756) | 0.325 (0.762) | -48.584 | < 0.001 |
| Pallidum L - Heschl R | 1.648 (0.829) | -0.054 (0.82) | 46.134 | < 0.001 |
| Pallidum L - Cerebelum 3 L | 1.29 (0.677) | 1.493 (0.955) | -5.484 | < 0.001 |
| Pallidum R - Cerebelum 3 R | -1.291 (0.871) | 0.695 (0.863) | -51.198 | < 0.001 |
| Pallidum R - Cerebelum 4 5 L | -1.306 (0.782) | 0.299 (0.967) | -40.781 | < 0.001 |
| Pallidum R - Vermis 10 | -1.431 (0.748) | -0.649 (0.812) | -22.416 | < 0.001 |
| Thalamus L - Heschl R | 2.084 (0.858) | -0.352 (0.922) | 61.163 | < 0.001 |
| Thalamus L - Cerebelum 10 R | -1.331 (0.778) | 0.167 (0.849) | -41.111 | < 0.001 |
| Thalamus R - Heschl R | 1.983 (0.837) | -0.538 (0.874) | 65.889 | < 0.001 |
| Thalamus R - Temporal Pole Mid R | 1.389 (0.756) | 0.026 (0.85) | 37.887 | < 0.001 |
| Heschl L - Vermis 4 5 | 1.87 (0.701) | 0.536 (0.906) | 36.846 | < 0.001 |
| Heschl R - Cerebelum 10 R | -1.653 (0.791) | 1.521 (1.005) | -78.514 | < 0.001 |
| Heschl R - Vermis 4 5 | 2.366 (0.839) | -0.187 (0.915) | 65.008 | < 0.001 |
| Temporal Sup L - Vermis 4 5 | 1.695 (0.825) | 0.35 (0.875) | 35.368 | < 0.001 |
| Temporal Sup R - Cerebelum 3 R | -1.464 (0.781) | -0.259 (0.844) | -33.131 | < 0.001 |
| Temporal Sup R - Vermis 4 5 | 1.343 (0.937) | 0.494 (0.919) | 20.441 | < 0.001 |
| Temporal Pole Sup L - Cerebelum 3 L | 1.382 (0.795) | 0.685 (0.912) | 18.223 | < 0.001 |
| Temporal Pole Sup L - Cerebelum 4 5 R | 1.481 (0.735) | 0.301 (0.898) | 32.137 | < 0.001 |
| Temporal Pole Sup R - Temporal Pole Mid L | 1.262 (0.764) | -0.519 (0.845) | 49.429 | < 0.001 |
| Temporal Pole Sup R - Cerebelum Crus1 R | 1.558 (0.88) | -0.469 (0.849) | 52.418 | < 0.001 |
| Temporal Pole Sup R - Cerebelum 4 5 R | 1.406 (0.757) | 0.716 (0.952) | 17.936 | < 0.001 |
| Temporal Pole Sup R - Vermis 4 5 | 1.522 (0.971) | -0.082 (0.831) | 39.661 | < 0.001 |
| Temporal Mid L - Cerebelum 3 L | 1.519 (0.807) | 0.561 (0.929) | 24.626 | < 0.001 |
| Temporal Mid L - Cerebelum 4 5 L | 1.452 (0.849) | 1.24 (0.901) | 5.424 | < 0.001 |
| Temporal Mid L - Cerebelum 4 5 R | 1.565 (0.806) | 1.029 (0.831) | 14.633 | < 0.001 |
| Temporal Mid L - Vermis 4 5 | 1.693 (0.879) | 1.056 (0.802) | 16.905 | < 0.001 |
| Temporal Mid L - Vermis 10 | -1.288 (0.736) | -0.659 (0.824) | -17.997 | < 0.001 |
| Temporal Mid R - Cerebelum Crus1 R | 1.312 (0.762) | 1.224 (0.871) | 2.414 | 0.022 |
| Temporal Mid R - Cerebelum 4 5 R | 1.941 (0.814) | 1.382 (0.771) | 15.771 | < 0.001 |
| Temporal Pole Mid L - Temporal Pole Mid R | 1.808 (0.709) | 0.22 (0.862) | 44.979 | < 0.001 |
| Temporal Pole Mid L - Cerebelum Crus1 L | 1.597 (0.845) | -0.537 (0.868) | 55.741 | < 0.001 |
| Temporal Pole Mid L - Cerebelum Crus2 L | 1.244 (0.82) | -0.121 (0.837) | 36.806 | < 0.001 |
| Temporal Pole Mid L - Cerebelum Crus2 R | 1.723 (0.897) | -0.006 (0.932) | 42.262 | < 0.001 |
| Temporal Pole Mid L - Cerebelum 6 R | 1.162 (0.84) | -0.313 (0.88) | 38.346 | < 0.001 |
| Temporal Pole Mid L - Cerebelum 7b L | 1.18 (0.924) | -0.261 (0.851) | 36.282 | < 0.001 |
| Temporal Pole Mid R - Temporal Inf L | 1.335 (0.682) | -0.349 (0.846) | 48.991 | < 0.001 |
| Temporal Pole Mid R - Cerebelum Crus2 R | 2.332 (0.871) | -0.047 (0.953) | 58.275 | < 0.001 |
| Temporal Pole Mid R - Cerebelum 6 R | 1.284 (0.808) | 0.105 (0.843) | 31.951 | < 0.001 |
| Temporal Pole Mid R - Cerebelum 7b R | 1.267 (0.93) | -0.194 (0.828) | 37.091 | < 0.001 |
| Temporal Pole Mid R - Cerebelum 10 L | 1.436 (0.826) | 0.252 (0.845) | 31.711 | < 0.001 |
| Temporal Pole Mid R - Cerebelum 10 R | 1.173 (0.868) | 0.238 (0.969) | 22.742 | < 0.001 |
| Temporal Inf L - Cerebelum Crus2 R | 1.254 (0.781) | -0.177 (0.901) | 37.968 | < 0.001 |
| Temporal Inf L - Cerebelum 3 L | 1.859 (0.832) | 0.739 (0.904) | 28.824 | < 0.001 |
| Temporal Inf L - Cerebelum 4 5 L | 1.584 (0.82) | 0.545 (0.891) | 27.129 | < 0.001 |
| Temporal Inf L - Cerebelum 4 5 R | 1.659 (0.751) | 0.287 (0.848) | 38.296 | < 0.001 |
| Temporal Inf L - Cerebelum 10 R | 1.351 (0.805) | -0.164 (0.874) | 40.321 | < 0.001 |
| Temporal Inf L - Vermis 3 | 1.312 (0.802) | 0.35 (0.907) | 25.119 | < 0.001 |
| Temporal Inf L - Vermis 4 5 | 1.637 (0.925) | 0.15 (0.83) | 37.837 | < 0.001 |
| Temporal Inf L - Vermis 6 | 1.559 (0.728) | 0.125 (0.818) | 41.413 | < 0.001 |
| Temporal Inf R - Cerebelum Crus2 R | 1.29 (0.771) | 0.551 (0.813) | 20.835 | < 0.001 |
| Cerebelum Crus1 L - Cerebelum 4 5 L | 1.935 (0.791) | 0.796 (0.954) | 29.073 | < 0.001 |
| Cerebelum Crus1 L - Cerebelum 4 5 R | 2.034 (0.801) | 0.949 (0.917) | 28.188 | < 0.001 |
| Cerebelum Crus1 L - Vermis 8 | 1.777 (0.744) | 0.612 (0.877) | 32.041 | < 0.001 |
| Cerebelum Crus1 R - Cerebelum 4 5 R | 1.823 (0.78) | 0.363 (0.899) | 38.771 | < 0.001 |
| Cerebelum Crus1 R - Vermis 8 | 1.91 (0.733) | -0.125 (0.863) | 56.834 | < 0.001 |
| Cerebelum Crus2 L - Cerebelum 4 5 L | 1.729 (0.755) | -0.015 (0.85) | 48.472 | < 0.001 |
| Cerebelum Crus2 L - Cerebelum 4 5 R | 1.357 (0.755) | 0.199 (0.86) | 32.007 | < 0.001 |
| Cerebelum Crus2 L - Cerebelum 7b L | 1.301 (0.776) | 0.421 (0.868) | 23.909 | < 0.001 |
| Cerebelum Crus2 L - Cerebelum 8 L | 1.935 (0.818) | 0.38 (0.923) | 39.875 | < 0.001 |
| Cerebelum Crus2 L - Cerebelum 9 L | 1.282 (0.805) | 0.134 (0.768) | 32.620 | < 0.001 |
| Cerebelum Crus2 L - Cerebelum 10 L | 1.378 (0.795) | 0.083 (0.924) | 33.609 | < 0.001 |
| Cerebelum Crus2 L - Vermis 8 | 2.269 (0.805) | 0.326 (0.914) | 50.450 | < 0.001 |
| Cerebelum Crus2 R - Cerebelum 4 5 L | 1.782 (0.816) | -0.444 (0.853) | 59.607 | < 0.001 |
| Cerebelum Crus2 R - Cerebelum 4 5 R | 2.014 (0.727) | -0.472 (0.819) | 71.793 | < 0.001 |
| Cerebelum Crus2 R - Cerebelum 7b L | 1.783 (0.788) | 0.209 (0.879) | 42.181 | < 0.001 |
| Cerebelum Crus2 R - Cerebelum 7b R | 1.71 (0.887) | -0.218 (0.916) | 47.815 | < 0.001 |
| Cerebelum Crus2 R - Cerebelum 8 L | 2.109 (0.832) | 0.33 (0.907) | 45.707 | < 0.001 |
| Cerebelum Crus2 R - Cerebelum 8 R | 1.936 (0.834) | 0.127 (0.797) | 49.594 | < 0.001 |
| Cerebelum Crus2 R - Cerebelum 9 R | 1.358 (0.817) | -0.454 (0.776) | 50.871 | < 0.001 |
| Cerebelum Crus2 R - Cerebelum 10 L | 1.674 (0.888) | 0.397 (0.957) | 30.934 | < 0.001 |
| Cerebelum Crus2 R - Cerebelum 10 R | 1.276 (0.847) | -0.002 (0.855) | 33.591 | < 0.001 |
| Cerebelum Crus2 R - Vermis 8 | 1.972 (0.826) | -0.37 (0.885) | 61.180 | < 0.001 |
| Cerebelum 3 L - Cerebelum 9 L | -1.187 (0.785) | 0.442 (0.802) | -45.885 | < 0.001 |
| Cerebelum 4 5 L - Cerebelum 10 L | 1.337 (0.858) | 0.32 (0.897) | 25.906 | < 0.001 |
| Cerebelum 4 5 L - Cerebelum 10 R | 1.632 (0.778) | -0.274 (0.823) | 53.241 | < 0.001 |
| Cerebelum 4 5 R - Cerebelum 6 L | 1.276 (0.689) | 0.595 (0.885) | 19.192 | < 0.001 |
| Cerebelum 4 5 R - Cerebelum 6 R | 1.615 (0.776) | 1.018 (0.831) | 16.595 | < 0.001 |
| Cerebelum 4 5 R - Vermis 1 2 | 1.328 (0.758) | -0.175 (1.017) | 37.496 | < 0.001 |
| Cerebelum 6 L - Vermis 8 | 1.365 (0.822) | 0.891 (0.827) | 12.856 | < 0.001 |
| Cerebelum 6 L - Vermis 9 | 1.392 (0.812) | 0.727 (0.799) | 18.467 | < 0.001 |
| Cerebelum 6 R - Vermis 8 | 1.445 (0.783) | 0.243 (0.832) | 33.243 | < 0.001 |
| Cerebelum 6 R - Vermis 9 | 1.395 (0.795) | 0.078 (0.804) | 36.813 | < 0.001 |
| Cerebelum 7b L - Cerebelum 7b R | 1.374 (0.788) | -0.569 (0.894) | 51.546 | < 0.001 |
| Cerebelum 7b L - Cerebelum 8 L | 1.919 (0.817) | 0.145 (0.808) | 48.822 | < 0.001 |
| Cerebelum 7b L - Cerebelum 8 R | 1.458 (0.849) | -0.683 (0.851) | 56.353 | < 0.001 |
| Cerebelum 7b L - Cerebelum 10 L | 2.126 (0.861) | 0.059 (0.969) | 50.424 | < 0.001 |
| Cerebelum 7b L - Cerebelum 10 R | 1.355 (0.771) | -0.095 (0.802) | 41.223 | < 0.001 |
| Cerebelum 7b L - Vermis 8 | 1.626 (0.91) | -0.068 (0.841) | 43.211 | < 0.001 |
| Cerebelum 7b R - Cerebelum 8 L | 1.458 (0.82) | -0.149 (0.898) | 41.799 | < 0.001 |
| Cerebelum 7b R - Cerebelum 8 R | 1.753 (0.811) | -0.146 (0.78) | 53.363 | < 0.001 |
| Cerebelum 7b R - Cerebelum 10 L | 1.829 (0.878) | -0.035 (0.964) | 45.204 | < 0.001 |
| Cerebelum 7b R - Cerebelum 10 R | 1.199 (0.842) | -0.365 (0.86) | 41.091 | < 0.001 |
| Cerebelum 7b R - Vermis 8 | 1.622 (0.808) | -0.774 (0.885) | 63.189 | < 0.001 |
| Cerebelum 8 L - Cerebelum 8 R | 1.333 (0.797) | -0.207 (0.897) | 40.605 | < 0.001 |
| Cerebelum 8 L - Cerebelum 9 L | 1.264 (0.747) | -0.159 (0.795) | 41.236 | < 0.001 |
| Cerebelum 8 L - Cerebelum 9 R | 1.465 (0.761) | 0.173 (0.804) | 36.888 | < 0.001 |
| Cerebelum 8 L - Cerebelum 10 L | 2.135 (0.846) | 0.086 (0.951) | 50.911 | < 0.001 |
| Cerebelum 8 L - Vermis 8 | 1.557 (0.808) | 0.151 (0.829) | 38.394 | < 0.001 |
| Cerebelum 8 R - Cerebelum 9 L | 1.283 (0.774) | -0.352 (0.817) | 45.946 | < 0.001 |
| Cerebelum 8 R - Cerebelum 9 R | 1.336 (0.783) | -0.179 (0.763) | 43.804 | < 0.001 |
| Cerebelum 8 R - Cerebelum 10 L | 1.592 (0.846) | -0.099 (0.922) | 42.723 | < 0.001 |
| Cerebelum 8 R - Vermis 8 | 1.363 (0.832) | 0.084 (0.855) | 33.909 | < 0.001 |
| Cerebelum 9 R - Vermis 8 | 1.383 (0.775) | 0.517 (0.859) | 23.664 | < 0.001 |
| Cerebelum 10 R - Vermis 4 5 | 1.399 (0.796) | -0.51 (0.859) | 51.525 | < 0.001 |
| Vermis 7 - Vermis 8 | 1.844 (0.828) | 0.544 (0.854) | 34.542 | < 0.001 |
| Vermis 7 - Vermis 10 | 1.316 (0.782) | -0.102 (0.917) | 37.208 | < 0.001 |
| Precentral L - Frontal Sup Medial R | 0.58 (0.817) | 1.193 (0.87) | -16.240 | < 0.001 |
| Precentral L - Cuneus L | -0.275 (0.839) | -1.253 (0.866) | 25.659 | < 0.001 |
| Precentral L - Occipital Sup L | -0.192 (0.864) | -1.21 (0.879) | 26.136 | < 0.001 |
| Precentral L - Occipital Mid L | -0.691 (0.889) | -1.35 (0.831) | 17.126 | < 0.001 |
| Precentral L - Fusiform R | -0.983 (0.737) | -1.274 (0.781) | 8.600 | < 0.001 |
| Precentral L - Cerebelum 8 L | -0.771 (0.872) | -1.37 (0.82) | 15.818 | < 0.001 |
| Precentral L - Vermis 8 | 0.647 (0.747) | -1.264 (0.826) | 54.254 | < 0.001 |
| Precentral R - Rolandic Oper R | -0.197 (0.907) | -1.84 (0.844) | 41.937 | < 0.001 |
| Precentral R - Calcarine R | -0.339 (0.798) | -1.307 (0.798) | 27.124 | < 0.001 |
| Precentral R - Cuneus L | -0.4 (0.774) | -1.238 (0.813) | 23.605 | < 0.001 |
| Precentral R - Cuneus R | -0.659 (0.816) | -1.258 (0.802) | 16.561 | < 0.001 |
| Precentral R - Occipital Sup L | -0.139 (0.79) | -1.332 (0.846) | 32.613 | < 0.001 |
| Precentral R - Occipital Sup R | -0.342 (0.826) | -1.586 (0.836) | 33.472 | < 0.001 |
| Precentral R - Occipital Mid L | -0.746 (0.796) | -1.352 (0.826) | 16.689 | < 0.001 |
| Precentral R - Occipital Mid R | -0.734 (0.884) | -1.427 (0.785) | 18.512 | < 0.001 |
| Precentral R - Fusiform L | -1.041 (0.777) | -1.188 (0.783) | 4.200 | < 0.001 |
| Precentral R - SupraMarginal L | -0.71 (0.848) | -1.144 (0.809) | 11.711 | < 0.001 |
| Precentral R - Temporal Sup R | -0.633 (0.838) | -1.146 (0.821) | 13.825 | < 0.001 |
| Precentral R - Vermis 10 | -0.475 (0.772) | -1.257 (0.786) | 22.430 | < 0.001 |
| Frontal Sup L - Frontal Mid L | 1.065 (0.928) | 1.159 (0.82) | -2.397 | 0.023 |
| Frontal Sup L - Insula R | 0.145 (0.774) | -1.079 (0.821) | 34.319 | < 0.001 |
| Frontal Sup R - Frontal Mid R | -0.258 (0.898) | 1.204 (0.779) | -38.864 | < 0.001 |
| Frontal Sup R - Vermis 10 | 0.156 (0.735) | -1.265 (0.829) | 40.552 | < 0.001 |
| Frontal Sup Orb L - Supp Motor Area L | -0.241 (0.709) | 1.246 (0.85) | -42.469 | < 0.001 |
| Frontal Sup Orb L - Supp Motor Area R | -1.108 (0.686) | 1.098 (0.833) | -64.658 | < 0.001 |
| Frontal Sup Orb L - ParaHippocampal L | 0.388 (0.832) | 1.249 (0.791) | -23.711 | < 0.001 |
| Frontal Sup Orb L - Vermis 6 | 0.46 (0.822) | 1.139 (0.884) | -17.794 | < 0.001 |
| Frontal Sup Orb L - Vermis 9 | -0.814 (0.728) | 1.463 (0.811) | -66.059 | < 0.001 |
| Frontal Sup Orb R - Frontal Inf Oper L | 0.609 (0.862) | 1.254 (0.769) | -17.649 | < 0.001 |
| Frontal Sup Orb R - Supp Motor Area L | -0.168 (0.736) | 1.284 (0.847) | -40.927 | < 0.001 |
| Frontal Sup Orb R - Hippocampus L | -0.399 (0.689) | 1.304 (0.86) | -48.873 | < 0.001 |
| Frontal Sup Orb R - Amygdala L | -0.625 (0.765) | 1.273 (1.063) | -45.838 | < 0.001 |
| Frontal Sup Orb R - Lingual L | 0.279 (0.801) | 1.148 (0.867) | -23.288 | < 0.001 |
| Frontal Sup Orb R - Caudate R | 0.303 (0.826) | 1.303 (0.85) | -26.687 | < 0.001 |
| Frontal Sup Orb R - Thalamus R | 0.088 (0.718) | 1.17 (0.761) | -32.683 | < 0.001 |
| Frontal Sup Orb R - Heschl L | -0.123 (0.869) | 1.514 (0.908) | -41.164 | < 0.001 |
| Frontal Sup Orb R - Cerebelum 4 5 L | 0.597 (0.839) | 1.234 (0.984) | -15.587 | < 0.001 |
| Frontal Sup Orb R - Cerebelum 4 5 R | 0.755 (0.778) | 1.199 (0.942) | -11.491 | < 0.001 |
| Frontal Mid L - Frontal Sup Medial L | 0.884 (0.856) | 1.35 (0.853) | -12.187 | < 0.001 |
| Frontal Mid L - Cerebelum Crus1 L | -0.594 (0.77) | -1.307 (0.9) | 19.044 | < 0.001 |
| Frontal Mid L - Cerebelum Crus2 L | -0.302 (0.739) | -1.338 (0.94) | 27.418 | < 0.001 |
| Frontal Mid L - Cerebelum 8 L | -0.893 (0.714) | -1.335 (0.777) | 13.244 | < 0.001 |
| Frontal Mid L - Cerebelum 10 R | -0.607 (0.764) | -1.102 (0.783) | 14.300 | < 0.001 |
| Frontal Mid L - Vermis 7 | -0.609 (0.768) | -1.299 (0.815) | 19.465 | < 0.001 |
| Frontal Mid L - Vermis 8 | -0.057 (0.66) | -1.722 (0.83) | 49.641 | < 0.001 |
| Frontal Mid L - Vermis 9 | -0.164 (0.836) | -1.351 (0.796) | 32.532 | < 0.001 |
| Frontal Mid R - Supp Motor Area R | 0.35 (0.764) | 1.385 (0.895) | -27.802 | < 0.001 |
| Frontal Mid R - Frontal Sup Medial L | 0.327 (0.821) | 1.535 (0.854) | -32.241 | < 0.001 |
| Frontal Mid R - Frontal Sup Medial R | 0.552 (0.914) | 1.499 (0.873) | -23.709 | < 0.001 |
| Frontal Mid R - Cingulum Ant L | 0.841 (0.816) | 1.096 (0.901) | -6.639 | < 0.001 |
| Frontal Mid R - Cingulum Ant R | 0.71 (0.776) | 1.449 (0.88) | -19.908 | < 0.001 |
| Frontal Mid Orb L - Olfactory L | 0.028 (0.736) | 1.062 (0.945) | -27.285 | < 0.001 |
| Frontal Mid Orb L - Frontal Sup Medial R | -0.269 (0.723) | 1.458 (0.901) | -47.283 | < 0.001 |
| Frontal Mid Orb L - ParaHippocampal L | 0.098 (0.801) | 1.557 (0.779) | -41.299 | < 0.001 |
| Frontal Mid Orb L - ParaHippocampal R | -0.22 (0.791) | 1.444 (0.822) | -46.135 | < 0.001 |
| Frontal Mid Orb L - Lingual L | -0.255 (0.77) | 1.276 (0.91) | -40.608 | < 0.001 |
| Frontal Mid Orb L - Cerebelum 4 5 L | 0.261 (0.803) | 1.391 (0.895) | -29.701 | < 0.001 |
| Frontal Mid Orb L - Vermis 1 2 | 0.169 (0.771) | 1.542 (0.915) | -36.297 | < 0.001 |
| Frontal Mid Orb R - Supp Motor Area R | 0.348 (0.696) | 1.211 (0.863) | -24.611 | < 0.001 |
| Frontal Mid Orb R - Frontal Sup Medial R | -0.545 (0.728) | 1.992 (0.894) | -69.620 | < 0.001 |
| Frontal Mid Orb R - ParaHippocampal L | -0.055 (0.836) | 1.733 (0.861) | -47.122 | < 0.001 |
| Frontal Mid Orb R - ParaHippocampal R | -0.214 (0.807) | 1.202 (0.849) | -38.235 | < 0.001 |
| Frontal Mid Orb R - Amygdala L | -0.245 (0.725) | 1.259 (0.984) | -38.927 | < 0.001 |
| Frontal Mid Orb R - Heschl L | 0.984 (0.79) | 1.274 (0.85) | -7.925 | < 0.001 |
| Frontal Mid Orb R - Cerebelum 4 5 L | 0.747 (0.797) | 1.496 (0.987) | -18.671 | < 0.001 |
| Frontal Mid Orb R - Cerebelum 6 L | 0.439 (0.811) | 1.479 (0.882) | -27.448 | < 0.001 |
| Frontal Mid Orb R - Vermis 1 2 | -0.176 (0.724) | 1.153 (0.865) | -37.273 | < 0.001 |
| Frontal Inf Oper L - Postcentral L | 0.094 (0.809) | 1.263 (0.919) | -30.195 | < 0.001 |
| Frontal Inf Oper L - Cerebelum 4 5 R | -0.252 (0.814) | -1.181 (0.827) | 25.309 | < 0.001 |
| Frontal Inf Oper L - Vermis 7 | 0.229 (0.885) | -1.194 (0.814) | 37.436 | < 0.001 |
| Frontal Inf Oper R - Frontal Sup Medial R | 0.032 (0.831) | 1.166 (0.849) | -30.215 | < 0.001 |
| Frontal Inf Oper R - Pallidum L | -0.789 (0.8) | -1.162 (0.845) | 10.130 | < 0.001 |
| Frontal Inf Tri L - Cingulum Post R | -0.27 (0.722) | 1.3 (0.847) | -44.614 | < 0.001 |
| Frontal Inf Tri L - Cerebelum 4 5 R | 0.202 (0.741) | -1.421 (0.847) | 45.603 | < 0.001 |
| Frontal Inf Tri L - Vermis 8 | 0.599 (0.712) | -1.108 (0.783) | 50.997 | < 0.001 |
| Frontal Inf Tri R - Fusiform R | -0.611 (0.696) | -1.174 (0.919) | 15.461 | < 0.001 |
| Frontal Inf Tri R - Pallidum L | -0.771 (0.877) | -1.187 (0.941) | 10.209 | < 0.001 |
| Frontal Inf Tri R - Temporal Pole Mid L | 0.458 (0.781) | 1.35 (0.967) | -22.697 | < 0.001 |
| Frontal Inf Tri R - Cerebelum 4 5 R | -0.149 (0.719) | -1.212 (0.797) | 31.316 | < 0.001 |
| Frontal Inf Tri R - Cerebelum 10 L | 0.735 (1.001) | -1.567 (0.909) | 53.849 | < 0.001 |
| Frontal Inf Tri R - Cerebelum 10 R | -0.581 (1.023) | -1.25 (0.921) | 15.372 | < 0.001 |
| Frontal Inf Tri R - Vermis 3 | -0.404 (0.788) | -1.245 (0.829) | 23.257 | < 0.001 |
| Frontal Inf Tri R - Vermis 10 | -0.473 (0.787) | -1.14 (0.781) | 19.025 | < 0.001 |
| Frontal Inf Orb L - ParaHippocampal L | -0.251 (0.818) | 1.143 (0.883) | -36.595 | < 0.001 |
| Frontal Inf Orb L - Vermis 3 | 0.814 (0.806) | 1.471 (0.923) | -16.945 | < 0.001 |
| Frontal Inf Orb R - Supp Motor Area R | -0.681 (0.728) | 1.286 (0.839) | -55.979 | < 0.001 |
| Frontal Inf Orb R - Hippocampus R | 0.217 (0.746) | 1.223 (0.843) | -28.246 | < 0.001 |
| Frontal Inf Orb R - ParaHippocampal L | 0.365 (0.818) | 1.384 (0.878) | -26.853 | < 0.001 |
| Frontal Inf Orb R - Cerebelum 6 L | -0.025 (0.758) | 1.425 (0.953) | -37.664 | < 0.001 |
| Frontal Inf Orb R - Cerebelum 6 R | -0.053 (0.735) | 1.169 (0.984) | -31.449 | < 0.001 |
| Frontal Inf Orb R - Vermis 6 | -0.228 (0.818) | 1.274 (0.99) | -37.004 | < 0.001 |
| Rolandic Oper L - Rolandic Oper R | 0.596 (0.821) | -1.204 (0.829) | 48.804 | < 0.001 |
| Rolandic Oper L - Cingulum Mid L | -0.287 (0.766) | -1.643 (0.779) | 39.282 | < 0.001 |
| Rolandic Oper L - Cingulum Mid R | 0.02 (0.797) | -1.17 (0.783) | 33.688 | < 0.001 |
| Rolandic Oper L - Lingual R | -0.145 (0.737) | -1.138 (0.8) | 28.893 | < 0.001 |
| Rolandic Oper L - Fusiform L | -0.558 (0.773) | -1.089 (0.742) | 15.662 | < 0.001 |
| Rolandic Oper L - Precuneus L | -0.597 (0.738) | -1.148 (0.748) | 16.587 | < 0.001 |
| Rolandic Oper L - Precuneus R | -0.501 (0.722) | -1.271 (0.776) | 22.955 | < 0.001 |
| Rolandic Oper L - Vermis 1 2 | -0.301 (0.684) | 1.388 (0.837) | -49.411 | < 0.001 |
| Rolandic Oper R - Insula L | -0.072 (0.761) | -1.374 (0.788) | 37.580 | < 0.001 |
| Rolandic Oper R - Insula R | -0.444 (0.761) | -1.279 (0.803) | 23.853 | < 0.001 |
| Rolandic Oper R - Cingulum Mid L | -0.038 (0.817) | -1.149 (0.809) | 30.554 | < 0.001 |
| Rolandic Oper R - Lingual R | 0.514 (0.801) | -1.152 (0.807) | 46.325 | < 0.001 |
| Rolandic Oper R - Postcentral L | -0.499 (0.899) | -1.121 (0.812) | 16.226 | < 0.001 |
| Rolandic Oper R - SupraMarginal R | 0.407 (0.789) | -1.263 (0.781) | 47.561 | < 0.001 |
| Rolandic Oper R - Paracentral Lobule R | 0.164 (0.792) | -1.44 (0.856) | 43.528 | < 0.001 |
| Rolandic Oper R - Temporal Sup L | -0.342 (0.892) | -1.37 (0.827) | 26.724 | < 0.001 |
| Rolandic Oper R - Temporal Sup R | -0.462 (0.903) | -1.473 (0.897) | 25.129 | < 0.001 |
| Rolandic Oper R - Cerebelum 4 5 R | -0.641 (0.757) | -1.246 (0.793) | 17.460 | < 0.001 |
| Rolandic Oper R - Cerebelum 8 L | 0.094 (0.736) | -1.345 (0.726) | 44.006 | < 0.001 |
| Rolandic Oper R - Cerebelum 10 R | -0.519 (0.8) | -1.06 (0.767) | 15.456 | < 0.001 |
| Supp Motor Area L - Calcarine R | -0.834 (0.798) | -1.2 (0.885) | 9.706 | < 0.001 |
| Supp Motor Area L - Cuneus L | 0.099 (0.796) | -1.143 (0.919) | 32.316 | < 0.001 |
| Supp Motor Area L - Lingual R | -0.935 (0.839) | -1.21 (0.92) | 6.984 | < 0.001 |
| Supp Motor Area L - Fusiform L | -0.508 (0.781) | -1.25 (0.858) | 20.219 | < 0.001 |
| Supp Motor Area R - Frontal Sup Medial L | -1.013 (0.708) | 1.182 (0.828) | -63.734 | < 0.001 |
| Supp Motor Area R - Rectus L | -0.199 (0.703) | 1.119 (0.787) | -39.449 | < 0.001 |
| Supp Motor Area R - Rectus R | -0.527 (0.805) | 1.338 (0.801) | -51.940 | < 0.001 |
| Supp Motor Area R - Insula L | -0.386 (0.789) | -1.324 (0.761) | 27.066 | < 0.001 |
| Supp Motor Area R - SupraMarginal L | -0.873 (0.835) | -1.108 (0.772) | 6.540 | < 0.001 |
| Supp Motor Area R - Paracentral Lobule L | 0.573 (0.87) | 1.143 (0.871) | -14.635 | < 0.001 |
| Olfactory L - Cerebelum 10 L | -0.225 (0.757) | 1.198 (0.829) | -40.055 | < 0.001 |
| Olfactory R - Heschl R | 0.104 (0.759) | 1.373 (0.815) | -36.038 | < 0.001 |
| Frontal Med Orb L - Vermis 1 2 | -0.328 (0.746) | 1.455 (0.842) | -50.100 | < 0.001 |
| Frontal Med Orb R - ParaHippocampal L | -1.233 (0.793) | 1.127 (0.922) | -61.362 | < 0.001 |
| Frontal Med Orb R - Parietal Sup L | 0.941 (0.808) | 1.464 (0.898) | -13.676 | < 0.001 |
| Frontal Med Orb R - Parietal Inf L | 1.08 (0.875) | 1.206 (0.803) | -3.350 | 0.001 |
| Frontal Med Orb R - Caudate L | -0.373 (0.797) | -1.216 (0.851) | 22.890 | < 0.001 |
| Frontal Med Orb R - Cerebelum 6 L | 0.149 (0.741) | 1.286 (0.839) | -32.134 | < 0.001 |
| Frontal Med Orb R - Cerebelum 8 R | -0.607 (0.748) | 1.497 (0.785) | -61.364 | < 0.001 |
| Frontal Med Orb R - Vermis 6 | -0.226 (0.712) | 1.542 (0.795) | -52.378 | < 0.001 |
| Rectus L - ParaHippocampal L | 0.587 (0.802) | 2.059 (0.946) | -37.546 | < 0.001 |
| Rectus L - ParaHippocampal R | 0.707 (0.828) | 1.268 (0.854) | -14.902 | < 0.001 |
| Rectus L - Lingual L | -0.253 (0.765) | 1.414 (0.844) | -46.290 | < 0.001 |
| Rectus L - Occipital Mid L | -0.815 (0.855) | 1.326 (0.756) | -59.345 | < 0.001 |
| Rectus L - Occipital Inf L | 0.839 (0.875) | 1.478 (0.893) | -16.161 | < 0.001 |
| Rectus L - Occipital Inf R | -0.084 (0.811) | 1.255 (0.958) | -33.723 | < 0.001 |
| Rectus L - Heschl L | 0.28 (0.91) | 1.172 (0.852) | -22.612 | < 0.001 |
| Rectus L - Temporal Inf R | 0.512 (0.764) | 1.307 (0.907) | -21.199 | < 0.001 |
| Rectus L - Cerebelum 6 L | 0.976 (0.812) | 1.416 (0.83) | -11.996 | < 0.001 |
| Rectus L - Cerebelum 6 R | 0.42 (0.811) | 1.253 (0.769) | -23.562 | < 0.001 |
| Rectus L - Cerebelum 8 L | -0.601 (0.839) | 1.095 (1.02) | -40.610 | < 0.001 |
| Rectus L - Cerebelum 8 R | -0.596 (0.785) | 1.167 (0.896) | -46.787 | < 0.001 |
| Rectus L - Cerebelum 10 L | 0.532 (0.768) | 1.349 (0.839) | -22.709 | < 0.001 |
| Rectus L - Vermis 6 | 0.645 (0.85) | 1.352 (0.824) | -18.897 | < 0.001 |
| Rectus L - Vermis 9 | 0.619 (0.778) | 1.133 (0.715) | -15.387 | < 0.001 |
| Rectus R - ParaHippocampal L | 0.533 (0.776) | 1.439 (0.984) | -22.859 | < 0.001 |
| Rectus R - ParaHippocampal R | 0.432 (0.827) | 1.822 (0.863) | -36.785 | < 0.001 |
| Rectus R - Occipital Inf R | 0.99 (0.793) | 1.381 (0.904) | -10.297 | < 0.001 |
| Rectus R - Fusiform R | 0.918 (0.821) | 1.196 (0.835) | -7.496 | < 0.001 |
| Rectus R - Heschl L | 0.547 (0.803) | 1.386 (0.868) | -22.432 | < 0.001 |
| Rectus R - Temporal Pole Sup L | 0.125 (0.829) | 1.123 (0.877) | -26.156 | < 0.001 |
| Rectus R - Temporal Inf L | 0.669 (0.827) | 1.422 (0.887) | -19.644 | < 0.001 |
| Rectus R - Temporal Inf R | 0.963 (0.783) | 1.335 (0.802) | -10.499 | < 0.001 |
| Rectus R - Cerebelum Crus1 L | 0.278 (0.828) | 1.146 (0.866) | -22.909 | < 0.001 |
| Rectus R - Cerebelum 3 L | 1.035 (0.825) | 1.318 (0.91) | -7.275 | < 0.001 |
| Rectus R - Vermis 8 | 0.305 (0.791) | 1.496 (0.799) | -33.488 | < 0.001 |
| Rectus R - Vermis 9 | 0.416 (0.758) | 1.172 (0.76) | -22.261 | < 0.001 |
| Insula L - Cingulum Ant L | -0.862 (0.782) | -1.195 (0.759) | 9.674 | < 0.001 |
| Insula L - Cingulum Mid L | -0.18 (0.78) | -1.217 (0.807) | 29.226 | < 0.001 |
| Insula L - Cingulum Mid R | -0.134 (0.806) | -1.343 (0.807) | 33.520 | < 0.001 |
| Insula L - Cerebelum Crus1 R | 0.383 (0.907) | -1.256 (0.789) | 43.092 | < 0.001 |
| Insula R - Cingulum Ant R | -0.332 (0.753) | -1.412 (0.752) | 32.110 | < 0.001 |
| Insula R - Cingulum Mid L | -1.128 (0.762) | -1.409 (0.793) | 8.076 | < 0.001 |
| Insula R - Occipital Inf L | -0.138 (0.807) | -1.118 (0.8) | 27.244 | < 0.001 |
| Insula R - Fusiform L | -0.751 (0.816) | -1.472 (0.777) | 20.241 | < 0.001 |
| Insula R - Fusiform R | -0.442 (0.856) | -1.26 (0.754) | 22.692 | < 0.001 |
| Insula R - Pallidum L | 0.727 (0.738) | -1.249 (0.882) | 54.338 | < 0.001 |
| Cingulum Ant R - Hippocampus L | -0.286 (0.665) | 1.13 (0.9) | -40.019 | < 0.001 |
| Cingulum Ant R - Cerebelum 8 L | 0.059 (0.784) | 1.251 (0.932) | -30.954 | < 0.001 |
| Cingulum Mid L - Cingulum Mid R | 0.227 (0.774) | -1.119 (0.873) | 36.511 | < 0.001 |
| Cingulum Mid L - Fusiform L | -0.992 (0.686) | -1.212 (0.777) | 6.706 | < 0.001 |
| Cingulum Mid L - Caudate L | -0.309 (0.753) | -1.218 (0.785) | 26.423 | < 0.001 |
| Cingulum Mid L - Thalamus L | -0.23 (0.726) | -1.233 (0.796) | 29.462 | < 0.001 |
| Cingulum Mid L - Heschl L | -0.481 (0.811) | -1.429 (0.732) | 27.421 | < 0.001 |
| Cingulum Mid L - Heschl R | 0.748 (0.869) | -1.919 (0.811) | 70.942 | < 0.001 |
| Cingulum Mid L - Cerebelum 8 L | -0.314 (0.755) | -1.384 (0.827) | 30.230 | < 0.001 |
| Cingulum Mid L - Vermis 8 | 0.21 (0.678) | -1.731 (0.818) | 57.767 | < 0.001 |
| Cingulum Mid R - Heschl R | 0.747 (0.847) | -1.596 (0.824) | 62.729 | < 0.001 |
| Cingulum Mid R - Temporal Sup R | 0.228 (0.894) | -1.24 (0.854) | 37.546 | < 0.001 |
| Cingulum Mid R - Cerebelum 4 5 L | -0.942 (0.745) | -1.218 (0.894) | 7.495 | < 0.001 |
| Cingulum Mid R - Cerebelum 8 L | -0.416 (0.778) | -1.672 (0.809) | 35.364 | < 0.001 |
| Cingulum Mid R - Vermis 8 | 0.019 (0.747) | -1.514 (0.795) | 44.426 | < 0.001 |
| Cingulum Post L - Cuneus R | 0.678 (0.744) | 1.235 (0.795) | -16.176 | < 0.001 |
| Cingulum Post L - SupraMarginal R | -0.285 (0.81) | 1.304 (0.785) | -44.563 | < 0.001 |
| Cingulum Post L - Cerebelum 10 R | -0.013 (0.844) | -1.098 (0.87) | 28.303 | < 0.001 |
| Cingulum Post R - Putamen R | 0.562 (0.754) | 1.282 (0.836) | -20.225 | < 0.001 |
| Cingulum Post R - Cerebelum Crus2 R | 0.801 (0.787) | -1.334 (0.827) | 59.115 | < 0.001 |
| Hippocampus L - Precuneus L | -0.39 (0.772) | -1.273 (0.77) | 25.604 | < 0.001 |
| Hippocampus R - Precuneus L | 0.289 (0.711) | -1.264 (0.755) | 47.318 | < 0.001 |
| Hippocampus R - Cerebelum 4 5 R | 0.928 (0.75) | 1.481 (0.849) | -15.443 | < 0.001 |
| Hippocampus R - Vermis 1 2 | -0.148 (0.732) | 1.156 (0.87) | -36.282 | < 0.001 |
| ParaHippocampal L - Pallidum L | 0.795 (0.789) | 1.127 (0.877) | -8.890 | < 0.001 |
| ParaHippocampal L - Temporal Sup L | -0.381 (0.712) | 1.192 (0.952) | -41.857 | < 0.001 |
| ParaHippocampal R - Cerebelum 10 L | 0.126 (0.82) | 1.709 (0.935) | -40.286 | < 0.001 |
| ParaHippocampal R - Vermis 4 5 | 0.83 (0.854) | 1.241 (0.832) | -10.901 | < 0.001 |
| ParaHippocampal R - Vermis 6 | 0.614 (0.756) | 1.32 (0.902) | -18.968 | < 0.001 |
| Amygdala L - Angular L | -0.528 (0.741) | 1.405 (0.942) | -50.988 | < 0.001 |
| Amygdala L - Cerebelum 9 R | -0.306 (0.704) | 1.198 (0.872) | -42.450 | < 0.001 |
| Amygdala L - Vermis 1 2 | -0.217 (0.76) | 1.13 (0.899) | -36.168 | < 0.001 |
| Amygdala R - Cuneus L | -0.406 (0.662) | -1.145 (0.751) | 23.344 | < 0.001 |
| Amygdala R - Cuneus R | -0.245 (0.702) | -1.112 (0.836) | 25.101 | < 0.001 |
| Amygdala R - Occipital Sup L | -0.375 (0.74) | -1.324 (0.886) | 25.974 | < 0.001 |
| Amygdala R - Pallidum L | -0.561 (0.82) | 1.049 (0.961) | -40.290 | < 0.001 |
| Amygdala R - Heschl L | 0.908 (0.837) | 1.23 (0.904) | -8.271 | < 0.001 |
| Amygdala R - Temporal Sup L | 0.318 (0.745) | 1.037 (0.932) | -19.053 | < 0.001 |
| Amygdala R - Temporal Inf R | -0.577 (0.661) | -1.36 (0.828) | 23.361 | < 0.001 |
| Calcarine L - Occipital Mid L | 0.536 (0.74) | 1.351 (0.873) | -22.526 | < 0.001 |
| Calcarine L - Occipital Mid R | 0.059 (0.759) | 1.229 (0.905) | -31.332 | < 0.001 |
| Calcarine L - Occipital Inf L | 0.209 (0.763) | 1.208 (0.935) | -26.199 | < 0.001 |
| Calcarine L - Postcentral L | -0.536 (0.838) | -1.16 (0.827) | 16.747 | < 0.001 |
| Calcarine L - Cerebelum 4 5 L | 1.017 (0.763) | 1.431 (0.982) | -10.521 | < 0.001 |
| Calcarine L - Cerebelum 6 L | 0.354 (0.795) | 1.225 (0.877) | -23.263 | < 0.001 |
| Calcarine L - Vermis 3 | 0.02 (0.755) | 1.517 (0.861) | -41.352 | < 0.001 |
| Calcarine L - Vermis 6 | 0.101 (0.824) | 1.258 (0.9) | -29.990 | < 0.001 |
| Calcarine L - Vermis 7 | -0.73 (0.804) | 1.086 (0.909) | -47.330 | < 0.001 |
| Calcarine R - Postcentral L | -0.264 (0.778) | -1.475 (0.772) | 34.955 | < 0.001 |
| Calcarine R - Postcentral R | 0.105 (0.785) | -1.16 (0.847) | 34.672 | < 0.001 |
| Calcarine R - Parietal Sup L | 0.301 (0.804) | -1.253 (0.839) | 42.287 | < 0.001 |
| Calcarine R - Cerebelum 4 5 L | 0.279 (0.685) | 1.194 (1.002) | -23.822 | < 0.001 |
| Calcarine R - Cerebelum 4 5 R | 0.968 (0.784) | 1.114 (0.88) | -3.895 | < 0.001 |
| Calcarine R - Vermis 3 | 0.891 (0.733) | 1.333 (0.855) | -12.399 | < 0.001 |
| Cuneus L - Postcentral L | -0.184 (0.793) | -1.615 (0.868) | 38.515 | < 0.001 |
| Cuneus L - Postcentral R | -0.257 (0.796) | -1.631 (0.9) | 36.161 | < 0.001 |
| Cuneus L - Parietal Sup L | 0.118 (0.78) | -1.783 (0.839) | 52.453 | < 0.001 |
| Cuneus L - Parietal Sup R | -0.307 (0.699) | -1.931 (0.819) | 47.673 | < 0.001 |
| Cuneus L - Angular L | 0.021 (0.732) | 1.401 (0.841) | -39.149 | < 0.001 |
| Cuneus L - Paracentral Lobule L | -0.434 (0.734) | -1.261 (0.892) | 22.660 | < 0.001 |
| Cuneus L - Paracentral Lobule R | -0.329 (0.771) | -1.347 (0.817) | 28.640 | < 0.001 |
| Cuneus L - Temporal Pole Sup R | 0.097 (0.827) | -1.352 (0.802) | 39.779 | < 0.001 |
| Cuneus L - Vermis 10 | -0.274 (0.814) | 1.516 (0.799) | -49.614 | < 0.001 |
| Cuneus R - Occipital Sup L | -0.103 (0.862) | -1.198 (0.876) | 28.158 | < 0.001 |
| Cuneus R - Postcentral R | -0.452 (0.804) | -1.368 (0.879) | 24.303 | < 0.001 |
| Cuneus R - Parietal Sup L | -0.233 (0.81) | -1.315 (0.816) | 29.751 | < 0.001 |
| Cuneus R - Parietal Sup R | -0.427 (0.783) | -1.487 (0.862) | 28.780 | < 0.001 |
| Cuneus R - Cerebelum 3 R | -0.533 (0.694) | 1.372 (1.037) | -48.272 | < 0.001 |
| Cuneus R - Cerebelum 4 5 L | 0.847 (0.7) | 1.083 (1.041) | -5.943 | < 0.001 |
| Cuneus R - Vermis 3 | -0.269 (0.73) | 1.471 (0.899) | -47.539 | < 0.001 |
| Cuneus R - Vermis 10 | -0.096 (0.803) | 1.572 (1.001) | -41.125 | < 0.001 |
| Lingual L - Vermis 3 | 0.279 (0.779) | 1.136 (0.968) | -21.819 | < 0.001 |
| Lingual L - Vermis 6 | 0.944 (0.771) | 1.514 (0.886) | -15.344 | < 0.001 |
| Lingual L - Vermis 9 | 0.652 (0.733) | 1.111 (0.812) | -13.276 | < 0.001 |
| Lingual R - Occipital Sup R | -0.031 (0.851) | -1.218 (0.847) | 31.261 | < 0.001 |
| Lingual R - Occipital Inf L | 0.291 (0.807) | 1.054 (0.917) | -19.758 | < 0.001 |
| Lingual R - Postcentral L | -0.43 (0.776) | -1.502 (0.788) | 30.667 | < 0.001 |
| Lingual R - Cerebelum Crus1 R | 0.861 (0.779) | 1.219 (0.844) | -9.854 | < 0.001 |
| Lingual R - Cerebelum 4 5 L | 1.038 (0.771) | 1.194 (0.975) | -3.979 | < 0.001 |
| Lingual R - Cerebelum 6 R | 0.764 (0.837) | 1.424 (0.831) | -17.676 | < 0.001 |
| Lingual R - Vermis 3 | 0.381 (0.75) | 1.213 (0.963) | -21.573 | < 0.001 |
| Lingual R - Vermis 6 | 0.473 (0.781) | 1.624 (0.931) | -29.944 | < 0.001 |
| Lingual R - Vermis 9 | 0.918 (0.76) | 1.246 (0.847) | -9.107 | < 0.001 |
| Lingual R - Vermis 10 | -0.105 (0.816) | 1.077 (1.001) | -28.962 | < 0.001 |
| Occipital Sup L - Postcentral R | -0.07 (0.798) | -1.264 (0.907) | 31.243 | < 0.001 |
| Occipital Sup L - Temporal Pole Sup L | -0.764 (0.78) | -1.555 (0.791) | 22.517 | < 0.001 |
| Occipital Sup L - Temporal Pole Mid R | 0.039 (0.696) | -1.409 (0.875) | 40.960 | < 0.001 |
| Occipital Sup L - Vermis 10 | -0.249 (0.83) | 1.13 (0.972) | -34.130 | < 0.001 |
| Occipital Sup R - Postcentral R | -0.231 (0.82) | -1.933 (0.869) | 45.038 | < 0.001 |
| Occipital Sup R - Parietal Sup L | -0.605 (0.847) | -1.263 (0.874) | 17.111 | < 0.001 |
| Occipital Sup R - Parietal Sup R | -0.979 (0.8) | -1.142 (0.862) | 4.385 | < 0.001 |
| Occipital Sup R - Paracentral Lobule R | -0.561 (0.822) | -1.301 (0.798) | 20.418 | < 0.001 |
| Occipital Sup R - Temporal Pole Sup L | -0.508 (0.78) | -1.196 (0.839) | 18.995 | < 0.001 |
| Occipital Sup R - Vermis 1 2 | 0.285 (0.895) | 1.164 (0.987) | -20.860 | < 0.001 |
| Occipital Sup R - Vermis 3 | 0.464 (0.703) | 1.224 (0.921) | -20.732 | < 0.001 |
| Occipital Sup R - Vermis 4 5 | 0.509 (0.833) | 1.126 (0.998) | -14.991 | < 0.001 |
| Occipital Mid L - Postcentral L | -0.645 (0.841) | -1.196 (0.858) | 14.497 | < 0.001 |
| Occipital Mid L - Postcentral R | -0.894 (0.848) | -1.544 (0.863) | 16.984 | < 0.001 |
| Occipital Mid L - Parietal Sup L | -0.226 (0.847) | -1.388 (0.911) | 29.539 | < 0.001 |
| Occipital Mid L - Parietal Sup R | -0.718 (0.8) | -1.354 (0.882) | 16.896 | < 0.001 |
| Occipital Mid L - Angular L | 0.868 (0.799) | 1.166 (0.844) | -8.125 | < 0.001 |
| Occipital Mid L - Cerebelum 3 R | 0.143 (0.72) | 1.369 (1.018) | -31.077 | < 0.001 |
| Occipital Mid L - Cerebelum 4 5 L | 0.598 (0.825) | 2.055 (1.052) | -34.497 | < 0.001 |
| Occipital Mid L - Cerebelum 4 5 R | 0.506 (0.834) | 1.99 (0.944) | -37.264 | < 0.001 |
| Occipital Mid L - Cerebelum 6 L | -0.209 (0.81) | 1.421 (0.93) | -41.808 | < 0.001 |
| Occipital Mid L - Vermis 3 | 0.37 (0.697) | 1.937 (0.871) | -44.441 | < 0.001 |
| Occipital Mid L - Vermis 6 | 0.398 (0.751) | 1.159 (0.91) | -20.367 | < 0.001 |
| Occipital Mid L - Vermis 10 | 0.862 (0.855) | 1.269 (0.961) | -9.990 | < 0.001 |
| Occipital Mid R - Postcentral R | -0.743 (0.885) | -1.696 (0.868) | 24.302 | < 0.001 |
| Occipital Mid R - Parietal Sup R | -1.135 (0.795) | -1.359 (0.877) | 6.000 | < 0.001 |
| Occipital Mid R - Heschl L | 0.005 (0.913) | 1.172 (0.881) | -29.072 | < 0.001 |
| Occipital Mid R - Cerebelum Crus1 R | 0.322 (0.802) | 1.114 (0.852) | -21.402 | < 0.001 |
| Occipital Mid R - Cerebelum 4 5 L | 0.403 (0.787) | 1.55 (1.065) | -27.396 | < 0.001 |
| Occipital Mid R - Cerebelum 4 5 R | 0.913 (0.797) | 1.499 (0.991) | -14.580 | < 0.001 |
| Occipital Mid R - Vermis 3 | 0.776 (0.739) | 1.29 (0.899) | -13.960 | < 0.001 |
| Occipital Mid R - Vermis 4 5 | 0.532 (0.848) | 1.442 (1.012) | -21.814 | < 0.001 |
| Occipital Inf L - Cerebelum Crus1 L | 0.646 (0.791) | 1.196 (0.878) | -14.724 | < 0.001 |
| Occipital Inf L - Cerebelum 6 L | 1.142 (0.803) | 1.365 (0.907) | -5.836 | < 0.001 |
| Occipital Inf L - Cerebelum 6 R | 1.067 (0.78) | 1.311 (0.883) | -6.554 | < 0.001 |
| Occipital Inf L - Vermis 3 | 0.673 (0.784) | 1.257 (0.925) | -15.233 | < 0.001 |
| Occipital Inf R - Temporal Mid R | 0.774 (0.799) | 1.185 (0.739) | -11.954 | < 0.001 |
| Occipital Inf R - Cerebelum 4 5 L | 0.594 (0.82) | 1.651 (1.003) | -25.775 | < 0.001 |
| Occipital Inf R - Cerebelum 4 5 R | 1.152 (0.759) | 1.458 (0.965) | -7.886 | < 0.001 |
| Occipital Inf R - Cerebelum 6 R | 0.313 (0.796) | 1.097 (0.855) | -21.228 | < 0.001 |
| Occipital Inf R - Vermis 4 5 | 0.735 (0.772) | 1.118 (0.922) | -10.075 | < 0.001 |
| Occipital Inf R - Vermis 10 | -0.474 (0.84) | 1.271 (1.061) | -40.778 | < 0.001 |
| Fusiform L - Postcentral L | -1.123 (0.752) | -1.462 (0.883) | 9.253 | < 0.001 |
| Fusiform L - Postcentral R | -0.877 (0.743) | -1.55 (0.799) | 19.498 | < 0.001 |
| Fusiform L - Parietal Sup L | -0.845 (0.799) | -1.366 (0.76) | 14.925 | < 0.001 |
| Fusiform L - Parietal Sup R | 0.056 (0.763) | -1.392 (0.799) | 41.449 | < 0.001 |
| Fusiform L - Cerebelum 8 L | 0.413 (0.689) | -1.336 (0.916) | 48.222 | < 0.001 |
| Fusiform L - Cerebelum 10 L | 0.357 (0.745) | 1.087 (1.015) | -18.320 | < 0.001 |
| Fusiform R - Postcentral R | -0.75 (0.698) | -1.253 (0.792) | 15.042 | < 0.001 |
| Fusiform R - Paracentral Lobule R | -1.206 (0.742) | -1.131 (0.77) | -2.198 | 0.039 |
| Fusiform R - Pallidum R | 0.518 (0.757) | -1.086 (0.852) | 44.517 | < 0.001 |
| Fusiform R - Cerebelum 3 L | 0.948 (0.832) | 1.376 (0.962) | -10.640 | < 0.001 |
| Fusiform R - Cerebelum 4 5 L | 1.136 (0.786) | 1.494 (0.942) | -9.236 | < 0.001 |
| Fusiform R - Cerebelum 6 R | 0.76 (0.798) | 1.169 (0.792) | -11.501 | < 0.001 |
| Fusiform R - Cerebelum 10 L | 0.78 (0.744) | 1.191 (0.977) | -10.579 | < 0.001 |
| Fusiform R - Vermis 3 | 0.919 (0.778) | 1.786 (0.846) | -23.878 | < 0.001 |
| Fusiform R - Vermis 4 5 | 0.982 (0.807) | 1.862 (0.883) | -23.261 | < 0.001 |
| Postcentral L - Pallidum R | 0.645 (0.763) | 1.258 (0.833) | -17.171 | < 0.001 |
| Postcentral L - Temporal Inf R | -0.843 (0.719) | -1.184 (0.825) | 9.865 | < 0.001 |
| Postcentral R - Parietal Inf R | 0.672 (0.807) | 1.549 (0.753) | -25.117 | < 0.001 |
| Parietal Sup L - Temporal Mid L | 0.153 (0.792) | -1.168 (0.779) | 37.596 | < 0.001 |
| Parietal Sup L - Vermis 10 | 0.498 (0.846) | -1.168 (0.814) | 44.881 | < 0.001 |
| Parietal Sup R - Putamen R | 0.821 (0.757) | 1.099 (1.01) | -6.978 | < 0.001 |
| Parietal Sup R - Temporal Pole Sup L | -0.28 (0.747) | -1.304 (0.847) | 28.671 | < 0.001 |
| Parietal Sup R - Temporal Inf R | -0.793 (0.745) | -1.127 (0.803) | 9.658 | < 0.001 |
| Parietal Sup R - Cerebelum 6 L | -0.026 (0.854) | -1.196 (0.849) | 30.727 | < 0.001 |
| Parietal Inf L - Angular L | 0.142 (0.829) | 1.137 (0.783) | -27.598 | < 0.001 |
| Parietal Inf L - Temporal Inf R | -1.06 (0.686) | -1.176 (0.815) | 3.453 | 0.001 |
| Parietal Inf L - Vermis 8 | 0.949 (0.788) | -1.347 (0.827) | 63.554 | < 0.001 |
| Parietal Inf R - Pallidum L | 0.008 (0.865) | -1.241 (0.872) | 32.177 | < 0.001 |
| SupraMarginal L - Temporal Mid R | -0.631 (0.718) | -1.128 (0.872) | 13.920 | < 0.001 |
| SupraMarginal L - Temporal Inf R | -0.707 (0.777) | -1.256 (0.837) | 15.215 | < 0.001 |
| SupraMarginal R - Heschl R | 0.523 (0.843) | -1.285 (0.81) | 48.912 | < 0.001 |
| SupraMarginal R - Temporal Sup L | -0.627 (0.757) | -1.125 (0.767) | 14.615 | < 0.001 |
| SupraMarginal R - Temporal Sup R | -0.523 (0.776) | -1.467 (0.822) | 26.419 | < 0.001 |
| SupraMarginal R - Temporal Mid L | 0.212 (0.707) | -1.106 (0.804) | 38.945 | < 0.001 |
| SupraMarginal R - Temporal Mid R | -0.735 (0.749) | -1.132 (0.848) | 11.109 | < 0.001 |
| Angular L - Precuneus R | 0.106 (0.869) | 1.143 (0.885) | -26.443 | < 0.001 |
| Angular L - Cerebelum Crus2 R | -0.607 (0.791) | -1.293 (0.846) | 18.722 | < 0.001 |
| Angular R - Temporal Pole Sup L | 0.278 (0.695) | -2.352 (0.819) | 77.391 | < 0.001 |
| Angular R - Temporal Pole Sup R | 0.994 (0.765) | -1.528 (0.864) | 69.083 | < 0.001 |
| Precuneus L - Putamen R | 0.096 (0.683) | 1.135 (0.922) | -28.630 | < 0.001 |
| Precuneus L - Vermis 10 | -0.029 (0.841) | -1.213 (0.797) | 32.336 | < 0.001 |
| Precuneus R - Temporal Pole Sup L | -0.114 (0.678) | -1.133 (0.897) | 28.644 | < 0.001 |
| Caudate R - Temporal Pole Mid L | 0.145 (0.831) | 1.213 (0.87) | -28.075 | < 0.001 |
| Caudate R - Cerebelum 8 L | -1.027 (0.859) | 1.082 (0.836) | -55.648 | < 0.001 |
| Caudate R - Cerebelum 8 R | -0.452 (0.841) | 1.649 (0.807) | -57.010 | < 0.001 |
| Putamen L - Putamen R | -0.456 (0.754) | -1.393 (0.877) | 25.647 | < 0.001 |
| Thalamus L - Cerebelum 4 5 L | -0.592 (0.748) | 1.254 (0.866) | -51.026 | < 0.001 |
| Heschl L - Temporal Sup L | 0.535 (0.833) | 1.129 (0.811) | -16.142 | < 0.001 |
| Temporal Sup L - Cerebelum 4 5 L | 0.403 (0.843) | 1.243 (0.934) | -21.094 | < 0.001 |
| Temporal Sup L - Cerebelum 6 L | 0.631 (0.758) | 1.377 (0.903) | -20.017 | < 0.001 |
| Temporal Sup R - Vermis 9 | 0.372 (0.759) | 1.258 (0.932) | -23.308 | < 0.001 |
| Temporal Pole Sup R - Temporal Mid L | 0.608 (0.69) | -1.186 (0.832) | 52.477 | < 0.001 |
| Temporal Pole Sup R - Cerebelum 3 R | 0.089 (0.683) | 1.159 (0.844) | -31.171 | < 0.001 |
| Temporal Mid R - Cerebelum 4 5 L | 0.617 (0.771) | 1.362 (0.874) | -20.219 | < 0.001 |
| Temporal Mid R - Vermis 6 | 0.91 (0.779) | 1.577 (0.824) | -18.608 | < 0.001 |
| Cerebelum Crus1 L - Cerebelum 6 L | 1.009 (0.745) | 1.16 (0.874) | -4.172 | < 0.001 |
| Cerebelum Crus1 R - Cerebelum 7b R | 0.167 (0.792) | -1.145 (0.824) | 36.290 | < 0.001 |
| Cerebelum 4 5 R - Vermis 4 5 | 0.925 (0.724) | 1.629 (0.91) | -19.143 | < 0.001 |
| Cerebelum 4 5 R - Vermis 7 | 0.333 (0.782) | 1.387 (0.898) | -27.984 | < 0.001 |
| Vermis 1 2 - Vermis 7 | -0.526 (0.768) | -1.186 (0.851) | 18.210 | < 0.001 |
| Vermis 3 - Vermis 6 | -0.316 (0.725) | 1.138 (0.87) | -40.607 | < 0.001 |
| Vermis 4 5 - Vermis 6 | 0.502 (0.801) | 1.127 (0.785) | -17.628 | < 0.001 |
| Vermis 4 5 - Vermis 7 | -0.159 (0.736) | 1.27 (0.906) | -38.730 | < 0.001 |
| Vermis 6 - Vermis 7 | 0.31 (0.842) | 1.438 (0.895) | -29.028 | < 0.001 |

**Supplementary Table 9**. Between-group comparison on regional frailty-related ROI-to-ROI connectivity effects between CU and AD. Mean (SD) t-values of the frailty–GMV association estimated separately in cognitively unimpaired (CU) and Alzheimer’s disease (AD) groups using repeated subsampling (n = 1000) and models adjusted for scanner effects.

| **Regions** | **CU** | **FTLD** | ***t*** | ***P*_FDR_** |
| --- | --- | --- | --- | --- |
| Precentral L - Lingual L | -1.645 (0.815) | -2.684 (0.218) | 38.942 | < 0.001 |
| Precentral L - Lingual R | -1.275 (0.849) | -2.85 (0.241) | 56.404 | < 0.001 |
| Precentral L - Fusiform L | -1.658 (0.835) | -2.467 (0.244) | 29.372 | < 0.001 |
| Precentral R - Occipital Inf R | -1.287 (0.784) | -1.418 (0.261) | 5.018 | < 0.001 |
| Frontal Sup Orb L - Cerebelum 3 L | 2.334 (0.847) | -0.003 (0.41) | 78.526 | < 0.001 |
| Frontal Sup Orb R - Frontal Inf Oper R | 1.194 (0.817) | 0.848 (0.259) | 12.779 | < 0.001 |
| Frontal Mid Orb L - Vermis 3 | 1.57 (0.872) | 1.209 (0.364) | 12.054 | < 0.001 |
| Frontal Mid Orb R - Cerebelum 3 L | 1.391 (0.772) | 1.171 (0.418) | 7.908 | < 0.001 |
| Frontal Inf Oper L - Temporal Inf L | -1.592 (0.786) | -2.855 (0.254) | 48.328 | < 0.001 |
| Frontal Med Orb R - Amygdala L | -1.295 (0.784) | 0.416 (0.389) | -61.804 | < 0.001 |
| Rectus L - Amygdala L | -1.398 (0.708) | -0.895 (0.268) | -21.010 | < 0.001 |
| Rectus L - Cerebelum 3 L | 1.293 (0.847) | -0.74 (0.475) | 66.200 | < 0.001 |
| Rectus L - Cerebelum 4 5 L | 1.464 (0.791) | -0.18 (0.323) | 60.827 | < 0.001 |
| Rectus R - Amygdala L | -1.906 (0.802) | -0.344 (0.335) | -56.811 | < 0.001 |
| Rectus R - Occipital Inf L | 1.947 (0.831) | 0.71 (0.299) | 44.313 | < 0.001 |
| Rectus R - Cerebelum 4 5 L | 1.493 (0.769) | 0.821 (0.352) | 25.143 | < 0.001 |
| Rectus R - Cerebelum 4 5 R | 1.62 (0.805) | 0.61 (0.333) | 36.658 | < 0.001 |
| Rectus R - Cerebelum 6 L | 1.494 (0.745) | 1.565 (0.296) | -2.792 | 0.008 |
| Cingulum Mid R - Fusiform L | -1.403 (0.708) | -2.25 (0.213) | 36.257 | < 0.001 |
| Cingulum Post L - Hippocampus L | 1.332 (0.761) | 0.94 (0.282) | 15.265 | < 0.001 |
| Hippocampus R - Vermis 4 5 | 1.315 (0.858) | 1.087 (0.291) | 7.948 | < 0.001 |
| ParaHippocampal R - Cerebelum 4 5 L | 1.308 (0.797) | 1.848 (0.328) | -19.836 | < 0.001 |
| ParaHippocampal R - Cerebelum 4 5 R | 1.661 (0.737) | 1.237 (0.276) | 17.041 | < 0.001 |
| Calcarine L - Vermis 4 5 | 1.315 (0.813) | 0.065 (0.265) | 46.224 | < 0.001 |
| Calcarine R - Vermis 4 5 | 1.708 (0.786) | 0.793 (0.236) | 35.227 | < 0.001 |
| Cuneus L - Fusiform R | -1.36 (0.772) | -2.175 (0.269) | 31.501 | < 0.001 |
| Cuneus L - Temporal Inf R | -1.554 (0.794) | -2.171 (0.221) | 23.663 | < 0.001 |
| Cuneus R - Fusiform L | -1.526 (0.856) | -1.991 (0.265) | 16.416 | < 0.001 |
| Cuneus R - Pallidum R | 1.472 (0.805) | 2.682 (0.291) | -44.704 | < 0.001 |
| Lingual L - Paracentral Lobule L | -1.247 (0.687) | -2.164 (0.224) | 40.142 | < 0.001 |
| Lingual L - Cerebelum 4 5 L | 1.704 (0.783) | 1.24 (0.331) | 17.296 | < 0.001 |
| Lingual L - Cerebelum 4 5 R | 1.494 (0.825) | 0.043 (0.261) | 53.072 | < 0.001 |
| Lingual L - Vermis 4 5 | 1.378 (0.835) | 0.63 (0.262) | 27.045 | < 0.001 |
| Lingual R - Paracentral Lobule L | -1.358 (0.756) | -3.159 (0.241) | 71.800 | < 0.001 |
| Lingual R - Paracentral Lobule R | -1.271 (0.77) | -1.926 (0.216) | 25.896 | < 0.001 |
| Lingual R - Cerebelum 4 5 R | 1.245 (0.83) | 0.695 (0.274) | 19.874 | < 0.001 |
| Occipital Sup L - Fusiform R | -1.259 (0.792) | -2.197 (0.252) | 35.692 | < 0.001 |
| Occipital Sup L - Temporal Inf R | -1.402 (0.792) | -1.895 (0.252) | 18.751 | < 0.001 |
| Occipital Mid L - Temporal Inf R | -1.471 (0.785) | -2.046 (0.246) | 22.091 | < 0.001 |
| Occipital Mid L - Vermis 4 5 | 1.369 (0.829) | -0.029 (0.282) | 50.496 | < 0.001 |
| Occipital Mid R - Temporal Inf R | -1.304 (0.72) | -2.232 (0.272) | 38.165 | < 0.001 |
| Occipital Inf L - Cerebelum 4 5 L | 2.035 (0.788) | 1.695 (0.328) | 12.589 | < 0.001 |
| Occipital Inf L - Cerebelum 4 5 R | 1.47 (0.776) | 1.196 (0.29) | 10.437 | < 0.001 |
| Occipital Inf L - Vermis 4 5 | 1.386 (0.75) | -0.216 (0.261) | 63.789 | < 0.001 |
| Occipital Inf L - Vermis 6 | 1.747 (0.747) | 1.222 (0.304) | 20.584 | < 0.001 |
| Occipital Inf R - Postcentral L | -1.251 (0.807) | -2.153 (0.263) | 33.615 | < 0.001 |
| Fusiform R - Cerebelum 4 5 R | 1.92 (0.834) | 0.764 (0.257) | 41.894 | < 0.001 |
| Fusiform R - Vermis 6 | 1.441 (0.784) | 0.666 (0.308) | 29.101 | < 0.001 |
| Postcentral L - Cerebelum Crus2 R | 1.314 (0.797) | 2.241 (0.278) | -34.749 | < 0.001 |
| Parietal Sup R - Caudate L | 1.878 (0.844) | 0.021 (0.338) | 64.567 | < 0.001 |
| Angular L - Temporal Pole Sup R | 1.452 (0.791) | 0.577 (0.24) | 33.452 | < 0.001 |
| Precuneus L - Cerebelum 7b R | -1.249 (0.816) | -1.923 (0.276) | 24.740 | < 0.001 |
| Putamen L - Cerebelum 4 5 L | -1.655 (0.783) | -1.018 (0.227) | -24.711 | < 0.001 |
| Pallidum L - Cerebelum 3 L | 1.29 (0.677) | -1.3 (0.248) | 113.687 | < 0.001 |
| Thalamus R - Heschl R | 1.983 (0.837) | 2.112 (0.275) | -4.637 | < 0.001 |
| Heschl R - Cerebelum 10 R | -1.653 (0.791) | 1.584 (0.257) | -123.118 | < 0.001 |
| Temporal Sup R - Vermis 4 5 | 1.343 (0.937) | 1.897 (0.315) | -17.748 | < 0.001 |
| Temporal Mid L - Vermis 4 5 | 1.693 (0.879) | 2.331 (0.319) | -21.573 | < 0.001 |
| Temporal Mid R - Cerebelum Crus1 R | 1.312 (0.762) | -1.183 (0.267) | 97.714 | < 0.001 |
| Temporal Mid R - Cerebelum 4 5 R | 1.941 (0.814) | 1.193 (0.299) | 27.288 | < 0.001 |
| Temporal Mid R - Vermis 4 5 | 1.367 (0.877) | 2.643 (0.275) | -43.907 | < 0.001 |
| Cerebelum 4 5 R - Vermis 1 2 | 1.328 (0.758) | 3.909 (0.302) | -100.016 | < 0.001 |
| Precentral L - Cuneus L | -0.275 (0.839) | -1.85 (0.246) | 56.985 | < 0.001 |
| Precentral L - Occipital Sup L | -0.192 (0.864) | -2.073 (0.264) | 65.872 | < 0.001 |
| Precentral L - Occipital Mid L | -0.691 (0.889) | -2.986 (0.303) | 77.317 | < 0.001 |
| Precentral L - Fusiform R | -0.983 (0.737) | -1.956 (0.226) | 39.975 | < 0.001 |
| Precentral L - Vermis 8 | 0.647 (0.747) | 2.401 (0.241) | -70.605 | < 0.001 |
| Precentral R - Rolandic Oper R | -0.197 (0.907) | -1.318 (0.252) | 37.671 | < 0.001 |
| Precentral R - Calcarine R | -0.339 (0.798) | -2.136 (0.198) | 69.067 | < 0.001 |
| Precentral R - Cuneus L | -0.4 (0.774) | -2.255 (0.219) | 72.913 | < 0.001 |
| Precentral R - Cuneus R | -0.659 (0.816) | -2.421 (0.241) | 65.476 | < 0.001 |
| Precentral R - Occipital Sup L | -0.139 (0.79) | -2.24 (0.225) | 80.935 | < 0.001 |
| Precentral R - Occipital Sup R | -0.342 (0.826) | -2.425 (0.232) | 76.781 | < 0.001 |
| Precentral R - Occipital Mid L | -0.746 (0.796) | -2.561 (0.212) | 69.646 | < 0.001 |
| Precentral R - Occipital Mid R | -0.734 (0.884) | -2.45 (0.252) | 58.995 | < 0.001 |
| Precentral R - Fusiform L | -1.041 (0.777) | -2.458 (0.223) | 55.393 | < 0.001 |
| Precentral R - SupraMarginal L | -0.71 (0.848) | -0.501 (0.231) | -7.519 | < 0.001 |
| Precentral R - Temporal Sup R | -0.633 (0.838) | -2.035 (0.267) | 50.409 | < 0.001 |
| Precentral R - Vermis 10 | -0.475 (0.772) | 0.758 (0.287) | -47.340 | < 0.001 |
| Frontal Sup L - Frontal Mid L | 1.065 (0.928) | -0.181 (0.257) | 40.918 | < 0.001 |
| Frontal Sup L - Insula R | 0.145 (0.774) | 0.33 (0.308) | -7.023 | < 0.001 |
| Frontal Sup R - Frontal Mid R | -0.258 (0.898) | -0.602 (0.272) | 11.593 | < 0.001 |
| Frontal Sup R - Vermis 10 | 0.156 (0.735) | 0.392 (0.32) | -9.328 | < 0.001 |
| Frontal Sup Orb L - Supp Motor Area L | -0.241 (0.709) | -0.567 (0.25) | 13.706 | < 0.001 |
| Frontal Sup Orb L - Supp Motor Area R | -1.108 (0.686) | -0.266 (0.275) | -36.036 | < 0.001 |
| Frontal Sup Orb L - Cerebelum 4 5 L | 1.096 (0.814) | 0.108 (0.344) | 35.377 | < 0.001 |
| Frontal Sup Orb L - Vermis 6 | 0.46 (0.822) | 0.096 (0.261) | 13.318 | < 0.001 |
| Frontal Sup Orb L - Vermis 9 | -0.814 (0.728) | 0.758 (0.228) | -65.153 | < 0.001 |
| Frontal Sup Orb R - Frontal Inf Oper L | 0.609 (0.862) | 0.539 (0.261) | 2.465 | 0.022 |
| Frontal Sup Orb R - Supp Motor Area L | -0.168 (0.736) | 0.326 (0.26) | -20.022 | < 0.001 |
| Frontal Sup Orb R - Hippocampus L | -0.399 (0.689) | 1.202 (0.323) | -66.531 | < 0.001 |
| Frontal Sup Orb R - Amygdala L | -0.625 (0.765) | 0.134 (0.264) | -29.686 | < 0.001 |
| Frontal Sup Orb R - Lingual L | 0.279 (0.801) | 1.195 (0.28) | -34.119 | < 0.001 |
| Frontal Sup Orb R - Thalamus R | 0.088 (0.718) | 0.182 (0.31) | -3.806 | < 0.001 |
| Frontal Sup Orb R - Heschl L | -0.123 (0.869) | 0.385 (0.273) | -17.639 | < 0.001 |
| Frontal Sup Orb R - Cerebelum 4 5 L | 0.597 (0.839) | 1.789 (0.319) | -41.987 | < 0.001 |
| Frontal Sup Orb R - Cerebelum 4 5 R | 0.755 (0.778) | 0.151 (0.317) | 22.753 | < 0.001 |
| Frontal Mid L - Frontal Sup Medial L | 0.884 (0.856) | 1.147 (0.253) | -9.304 | < 0.001 |
| Frontal Mid L - Frontal Sup Medial R | 1.109 (0.889) | 0.755 (0.268) | 12.050 | < 0.001 |
| Frontal Mid L - Cerebelum Crus1 L | -0.594 (0.77) | -0.931 (0.289) | 12.953 | < 0.001 |
| Frontal Mid L - Cerebelum Crus2 L | -0.302 (0.739) | -1.005 (0.284) | 28.100 | < 0.001 |
| Frontal Mid L - Cerebelum 8 L | -0.893 (0.714) | 0.124 (0.282) | -41.864 | < 0.001 |
| Frontal Mid L - Cerebelum 10 R | -0.607 (0.764) | 0.319 (0.288) | -35.868 | < 0.001 |
| Frontal Mid L - Vermis 7 | -0.609 (0.768) | -0.028 (0.274) | -22.550 | < 0.001 |
| Frontal Mid L - Vermis 8 | -0.057 (0.66) | 1.359 (0.256) | -63.276 | < 0.001 |
| Frontal Mid L - Vermis 9 | -0.164 (0.836) | 1.163 (0.25) | -48.089 | < 0.001 |
| Frontal Mid R - Supp Motor Area R | 0.35 (0.764) | -0.604 (0.289) | 36.946 | < 0.001 |
| Frontal Mid R - Frontal Sup Medial L | 0.327 (0.821) | 1.562 (0.233) | -45.761 | < 0.001 |
| Frontal Mid R - Frontal Sup Medial R | 0.552 (0.914) | 0.945 (0.278) | -13.013 | < 0.001 |
| Frontal Mid R - Cingulum Ant R | 0.71 (0.776) | 0.234 (0.275) | 18.300 | < 0.001 |
| Frontal Mid Orb L - Olfactory L | 0.028 (0.736) | -0.685 (0.26) | 28.895 | < 0.001 |
| Frontal Mid Orb L - Frontal Sup Medial R | -0.269 (0.723) | 2.04 (0.232) | -96.166 | < 0.001 |
| Frontal Mid Orb L - ParaHippocampal R | -0.22 (0.791) | 1.089 (0.251) | -49.863 | < 0.001 |
| Frontal Mid Orb L - Lingual L | -0.255 (0.77) | 0.351 (0.248) | -23.689 | < 0.001 |
| Frontal Mid Orb L - Cerebelum 4 5 L | 0.261 (0.803) | 0.541 (0.333) | -10.157 | < 0.001 |
| Frontal Mid Orb L - Vermis 1 2 | 0.169 (0.771) | 0.378 (0.35) | -7.794 | < 0.001 |
| Frontal Mid Orb R - Frontal Sup Medial R | -0.545 (0.728) | 1.275 (0.291) | -73.388 | < 0.001 |
| Frontal Mid Orb R - ParaHippocampal R | -0.214 (0.807) | 1.282 (0.287) | -55.255 | < 0.001 |
| Frontal Mid Orb R - Amygdala L | -0.245 (0.725) | -0.074 (0.277) | -6.958 | < 0.001 |
| Frontal Mid Orb R - Heschl L | 0.984 (0.79) | -0.415 (0.288) | 52.606 | < 0.001 |
| Frontal Mid Orb R - Cerebelum 4 5 L | 0.747 (0.797) | 1.685 (0.36) | -33.886 | < 0.001 |
| Frontal Mid Orb R - Cerebelum 6 L | 0.439 (0.811) | 0.168 (0.235) | 10.154 | < 0.001 |
| Frontal Mid Orb R - Vermis 1 2 | -0.176 (0.724) | 1.173 (0.347) | -53.142 | < 0.001 |
| Frontal Inf Oper L - Postcentral L | 0.094 (0.809) | -1.169 (0.24) | 47.363 | < 0.001 |
| Frontal Inf Oper L - Cerebelum 4 5 R | -0.252 (0.814) | -1.005 (0.238) | 28.076 | < 0.001 |
| Frontal Inf Oper L - Vermis 7 | 0.229 (0.885) | 0.351 (0.219) | -4.228 | < 0.001 |
| Frontal Inf Oper R - Frontal Sup Medial R | 0.032 (0.831) | -0.299 (0.238) | 12.102 | < 0.001 |
| Frontal Inf Oper R - Pallidum L | -0.789 (0.8) | 0.679 (0.321) | -53.865 | < 0.001 |
| Frontal Inf Tri L - Cingulum Post R | -0.27 (0.722) | -0.406 (0.226) | 5.681 | < 0.001 |
| Frontal Inf Tri L - Cerebelum 4 5 R | 0.202 (0.741) | -0.553 (0.25) | 30.555 | < 0.001 |
| Frontal Inf Tri L - Vermis 8 | 0.599 (0.712) | 0.277 (0.331) | 12.962 | < 0.001 |
| Frontal Inf Tri R - Fusiform R | -0.611 (0.696) | 0.299 (0.291) | -38.140 | < 0.001 |
| Frontal Inf Tri R - Pallidum L | -0.771 (0.877) | 0.147 (0.286) | -31.476 | < 0.001 |
| Frontal Inf Tri R - Temporal Pole Mid L | 0.458 (0.781) | -0.541 (0.278) | 38.123 | < 0.001 |
| Frontal Inf Tri R - Cerebelum 4 5 R | -0.149 (0.719) | -0.611 (0.25) | 19.204 | < 0.001 |
| Frontal Inf Tri R - Cerebelum 10 L | 0.735 (1.001) | -1.058 (0.192) | 55.626 | < 0.001 |
| Frontal Inf Tri R - Cerebelum 10 R | -0.581 (1.023) | 0.022 (0.225) | -18.191 | < 0.001 |
| Frontal Inf Tri R - Vermis 3 | -0.404 (0.788) | -0.521 (0.194) | 4.573 | < 0.001 |
| Frontal Inf Tri R - Vermis 10 | -0.473 (0.787) | -0.951 (0.208) | 18.586 | < 0.001 |
| Frontal Inf Orb L - ParaHippocampal L | -0.251 (0.818) | -1.056 (0.304) | 29.165 | < 0.001 |
| Frontal Inf Orb L - Vermis 3 | 0.814 (0.806) | 1.251 (0.368) | -15.579 | < 0.001 |
| Frontal Inf Orb R - Supp Motor Area R | -0.681 (0.728) | -0.242 (0.283) | -17.778 | < 0.001 |
| Frontal Inf Orb R - Hippocampus R | 0.217 (0.746) | 1.452 (0.245) | -49.738 | < 0.001 |
| Frontal Inf Orb R - ParaHippocampal L | 0.365 (0.818) | 0.706 (0.31) | -12.322 | < 0.001 |
| Frontal Inf Orb R - Cerebelum 6 L | -0.025 (0.758) | -0.103 (0.239) | 3.080 | 0.003 |
| Frontal Inf Orb R - Cerebelum 6 R | -0.053 (0.735) | 0.945 (0.266) | -40.377 | < 0.001 |
| Frontal Inf Orb R - Vermis 6 | -0.228 (0.818) | 0.61 (0.278) | -30.707 | < 0.001 |
| Rolandic Oper L - Rolandic Oper R | 0.596 (0.821) | -0.041 (0.256) | 23.412 | < 0.001 |
| Rolandic Oper L - Cingulum Mid L | -0.287 (0.766) | -0.928 (0.248) | 25.199 | < 0.001 |
| Rolandic Oper L - Cingulum Mid R | 0.02 (0.797) | -0.548 (0.226) | 21.704 | < 0.001 |
| Rolandic Oper L - Lingual R | -0.145 (0.737) | -0.588 (0.242) | 18.090 | < 0.001 |
| Rolandic Oper L - Fusiform L | -0.558 (0.773) | -1.438 (0.196) | 34.905 | < 0.001 |
| Rolandic Oper L - Precuneus L | -0.597 (0.738) | 1.374 (0.271) | -79.294 | < 0.001 |
| Rolandic Oper L - Precuneus R | -0.501 (0.722) | 1.5 (0.303) | -80.838 | < 0.001 |
| Rolandic Oper L - Vermis 1 2 | -0.301 (0.684) | 0.294 (0.322) | -24.872 | < 0.001 |
| Rolandic Oper R - Insula L | -0.072 (0.761) | 0.081 (0.295) | -5.941 | < 0.001 |
| Rolandic Oper R - Insula R | -0.444 (0.761) | 0.38 (0.327) | -31.477 | < 0.001 |
| Rolandic Oper R - Cingulum Mid L | -0.038 (0.817) | -2.793 (0.247) | 102.011 | < 0.001 |
| Rolandic Oper R - Lingual R | 0.514 (0.801) | -0.408 (0.243) | 34.811 | < 0.001 |
| Rolandic Oper R - Postcentral L | -0.499 (0.899) | -1.918 (0.249) | 48.096 | < 0.001 |
| Rolandic Oper R - SupraMarginal R | 0.407 (0.789) | -1.524 (0.279) | 72.976 | < 0.001 |
| Rolandic Oper R - Paracentral Lobule R | 0.164 (0.792) | -0.669 (0.246) | 31.771 | < 0.001 |
| Rolandic Oper R - Temporal Sup L | -0.342 (0.892) | -1.061 (0.25) | 24.544 | < 0.001 |
| Rolandic Oper R - Temporal Sup R | -0.462 (0.903) | -0.706 (0.242) | 8.256 | < 0.001 |
| Rolandic Oper R - Cerebelum 4 5 R | -0.641 (0.757) | 0.379 (0.315) | -39.337 | < 0.001 |
| Rolandic Oper R - Cerebelum 8 L | 0.094 (0.736) | -0.169 (0.218) | 10.834 | < 0.001 |
| Rolandic Oper R - Cerebelum 10 R | -0.519 (0.8) | 0.445 (0.294) | -35.761 | < 0.001 |
| Supp Motor Area L - Calcarine R | -0.834 (0.798) | -1.775 (0.243) | 35.646 | < 0.001 |
| Supp Motor Area L - Cuneus L | 0.099 (0.796) | -1.543 (0.247) | 62.310 | < 0.001 |
| Supp Motor Area L - Lingual R | -0.935 (0.839) | -1.359 (0.244) | 15.372 | < 0.001 |
| Supp Motor Area R - Frontal Sup Medial L | -1.013 (0.708) | 0.24 (0.253) | -52.730 | < 0.001 |
| Supp Motor Area R - Rectus L | -0.199 (0.703) | 0.394 (0.308) | -24.397 | < 0.001 |
| Supp Motor Area R - Rectus R | -0.527 (0.805) | 1.155 (0.279) | -62.470 | < 0.001 |
| Supp Motor Area R - Insula L | -0.386 (0.789) | -1.052 (0.291) | 25.051 | < 0.001 |
| Supp Motor Area R - SupraMarginal L | -0.873 (0.835) | -0.807 (0.228) | -2.419 | 0.025 |
| Supp Motor Area R - Paracentral Lobule L | 0.573 (0.87) | -1.144 (0.28) | 59.424 | < 0.001 |
| Olfactory L - Cerebelum 10 L | -0.225 (0.757) | -0.853 (0.314) | 24.243 | < 0.001 |
| Olfactory R - Heschl R | 0.104 (0.759) | -0.942 (0.268) | 41.104 | < 0.001 |
| Frontal Sup Medial L - Caudate L | -1.032 (0.762) | 0.958 (0.281) | -77.500 | < 0.001 |
| Frontal Med Orb L - Vermis 1 2 | -0.328 (0.746) | 2.286 (0.316) | -102.015 | < 0.001 |
| Frontal Med Orb R - ParaHippocampal L | -1.233 (0.793) | -0.296 (0.331) | -34.498 | < 0.001 |
| Frontal Med Orb R - Parietal Sup L | 0.941 (0.808) | -0.044 (0.29) | 36.307 | < 0.001 |
| Frontal Med Orb R - Parietal Inf L | 1.08 (0.875) | 0.29 (0.292) | 27.074 | < 0.001 |
| Frontal Med Orb R - Caudate L | -0.373 (0.797) | 0.601 (0.251) | -36.860 | < 0.001 |
| Frontal Med Orb R - Cerebelum 6 L | 0.149 (0.741) | -0.363 (0.195) | 21.138 | < 0.001 |
| Frontal Med Orb R - Vermis 6 | -0.226 (0.712) | 0.073 (0.261) | -12.454 | < 0.001 |
| Rectus L - ParaHippocampal L | 0.587 (0.802) | -0.296 (0.351) | 31.884 | < 0.001 |
| Rectus L - ParaHippocampal R | 0.707 (0.828) | -1.264 (0.358) | 69.101 | < 0.001 |
| Rectus L - Lingual L | -0.253 (0.765) | 1.383 (0.281) | -63.480 | < 0.001 |
| Rectus L - Occipital Mid L | -0.815 (0.855) | 0.968 (0.263) | -63.049 | < 0.001 |
| Rectus L - Occipital Inf L | 0.839 (0.875) | 0.368 (0.309) | 16.072 | < 0.001 |
| Rectus L - Occipital Inf R | -0.084 (0.811) | -0.764 (0.277) | 25.090 | < 0.001 |
| Rectus L - Heschl L | 0.28 (0.91) | 0.45 (0.268) | -5.667 | < 0.001 |
| Rectus L - Temporal Inf R | 0.512 (0.764) | -1.174 (0.28) | 65.473 | < 0.001 |
| Rectus L - Cerebelum 6 L | 0.976 (0.812) | 1.45 (0.265) | -17.560 | < 0.001 |
| Rectus L - Cerebelum 6 R | 0.42 (0.811) | 0.884 (0.253) | -17.281 | < 0.001 |
| Rectus L - Cerebelum 8 L | -0.601 (0.839) | -0.084 (0.261) | -18.640 | < 0.001 |
| Rectus L - Cerebelum 8 R | -0.596 (0.785) | 0.003 (0.269) | -22.793 | < 0.001 |
| Rectus L - Cerebelum 10 L | 0.532 (0.768) | -0.956 (0.269) | 57.818 | < 0.001 |
| Rectus L - Vermis 6 | 0.645 (0.85) | 0.358 (0.262) | 10.209 | < 0.001 |
| Rectus L - Vermis 9 | 0.619 (0.778) | 0.911 (0.268) | -11.245 | < 0.001 |
| Rectus R - ParaHippocampal L | 0.533 (0.776) | 0.357 (0.373) | 6.454 | < 0.001 |
| Rectus R - ParaHippocampal R | 0.432 (0.827) | 0.751 (0.373) | -11.134 | < 0.001 |
| Rectus R - Occipital Inf R | 0.99 (0.793) | 0.083 (0.28) | 34.099 | < 0.001 |
| Rectus R - Fusiform R | 0.918 (0.821) | 1.133 (0.285) | -7.818 | < 0.001 |
| Rectus R - Heschl L | 0.547 (0.803) | -0.13 (0.258) | 25.376 | < 0.001 |
| Rectus R - Temporal Pole Sup L | 0.125 (0.829) | -0.033 (0.31) | 5.617 | < 0.001 |
| Rectus R - Temporal Inf L | 0.669 (0.827) | -0.372 (0.256) | 38.024 | < 0.001 |
| Rectus R - Temporal Inf R | 0.963 (0.783) | -0.295 (0.246) | 48.494 | < 0.001 |
| Rectus R - Cerebelum Crus1 L | 0.278 (0.828) | 0.182 (0.265) | 3.494 | 0.001 |
| Rectus R - Cerebelum 3 L | 1.035 (0.825) | 0.224 (0.437) | 27.483 | < 0.001 |
| Rectus R - Vermis 8 | 0.305 (0.791) | -0.85 (0.245) | 44.096 | < 0.001 |
| Rectus R - Vermis 9 | 0.416 (0.758) | 1.136 (0.276) | -28.213 | < 0.001 |
| Insula L - Cingulum Ant L | -0.862 (0.782) | -1.286 (0.3) | 16.031 | < 0.001 |
| Insula L - Cingulum Mid L | -0.18 (0.78) | 0.458 (0.411) | -22.909 | < 0.001 |
| Insula L - Cingulum Mid R | -0.134 (0.806) | 0.032 (0.302) | -6.102 | < 0.001 |
| Insula L - Cerebelum Crus1 R | 0.383 (0.907) | 0.546 (0.251) | -5.488 | < 0.001 |
| Insula R - Cingulum Ant R | -0.332 (0.753) | 0.075 (0.27) | -16.088 | < 0.001 |
| Insula R - Cingulum Mid L | -1.128 (0.762) | 0.368 (0.317) | -57.385 | < 0.001 |
| Insula R - Calcarine L | -1.136 (0.72) | 0.753 (0.278) | -77.363 | < 0.001 |
| Insula R - Occipital Inf L | -0.138 (0.807) | 0.235 (0.291) | -13.785 | < 0.001 |
| Insula R - Fusiform L | -0.751 (0.816) | 0.909 (0.259) | -61.304 | < 0.001 |
| Insula R - Fusiform R | -0.442 (0.856) | 2.65 (0.421) | -102.511 | < 0.001 |
| Cingulum Ant R - Hippocampus L | -0.286 (0.665) | 1.736 (0.26) | -89.522 | < 0.001 |
| Cingulum Ant R - Cerebelum 8 L | 0.059 (0.784) | -0.371 (0.225) | 16.666 | < 0.001 |
| Cingulum Mid L - Cingulum Mid R | 0.227 (0.774) | -0.47 (0.271) | 26.916 | < 0.001 |
| Cingulum Mid L - Fusiform L | -0.992 (0.686) | -1.17 (0.269) | 7.625 | < 0.001 |
| Cingulum Mid L - Caudate L | -0.309 (0.753) | 0.611 (0.28) | -36.245 | < 0.001 |
| Cingulum Mid L - Thalamus L | -0.23 (0.726) | 0.777 (0.235) | -41.753 | < 0.001 |
| Cingulum Mid L - Heschl L | -0.481 (0.811) | -0.749 (0.268) | 9.929 | < 0.001 |
| Cingulum Mid L - Heschl R | 0.748 (0.869) | -1.557 (0.274) | 79.955 | < 0.001 |
| Cingulum Mid L - Cerebelum 8 L | -0.314 (0.755) | -1.334 (0.211) | 41.155 | < 0.001 |
| Cingulum Mid R - Heschl R | 0.747 (0.847) | -1.786 (0.238) | 91.074 | < 0.001 |
| Cingulum Mid R - Temporal Sup R | 0.228 (0.894) | -2.771 (0.231) | 102.693 | < 0.001 |
| Cingulum Mid R - Cerebelum 4 5 L | -0.942 (0.745) | -1.175 (0.217) | 9.502 | < 0.001 |
| Cingulum Mid R - Cerebelum 8 L | -0.416 (0.778) | -1.591 (0.216) | 46.019 | < 0.001 |
| Cingulum Mid R - Vermis 8 | 0.019 (0.747) | 0.512 (0.248) | -19.810 | < 0.001 |
| Cingulum Post L - Cuneus R | 0.678 (0.744) | 0.009 (0.276) | 26.680 | < 0.001 |
| Cingulum Post L - SupraMarginal R | -0.285 (0.81) | 0.05 (0.283) | -12.350 | < 0.001 |
| Cingulum Post L - Cerebelum 10 R | -0.013 (0.844) | -0.287 (0.238) | 9.894 | < 0.001 |
| Cingulum Post R - Putamen R | 0.562 (0.754) | 0.15 (0.23) | 16.503 | < 0.001 |
| Cingulum Post R - Cerebelum Crus2 R | 0.801 (0.787) | -0.947 (0.25) | 66.924 | < 0.001 |
| Hippocampus L - Precuneus L | -0.39 (0.772) | -0.935 (0.271) | 21.041 | < 0.001 |
| Hippocampus R - Precuneus L | 0.289 (0.711) | -0.785 (0.254) | 44.950 | < 0.001 |
| Hippocampus R - Cerebelum 4 5 R | 0.928 (0.75) | 0.243 (0.258) | 27.341 | < 0.001 |
| Hippocampus R - Vermis 1 2 | -0.148 (0.732) | -0.3 (0.288) | 6.122 | < 0.001 |
| ParaHippocampal L - Pallidum L | 0.795 (0.789) | -0.22 (0.232) | 39.010 | < 0.001 |
| ParaHippocampal L - Temporal Sup L | -0.381 (0.712) | -0.262 (0.267) | -4.972 | < 0.001 |
| ParaHippocampal R - Cerebelum 10 L | 0.126 (0.82) | -0.297 (0.269) | 15.480 | < 0.001 |
| ParaHippocampal R - Vermis 4 5 | 0.83 (0.854) | -0.079 (0.269) | 32.105 | < 0.001 |
| ParaHippocampal R - Vermis 6 | 0.614 (0.756) | 1.693 (0.284) | -42.266 | < 0.001 |
| Amygdala L - Angular L | -0.528 (0.741) | 1.965 (0.241) | -101.162 | < 0.001 |
| Amygdala L - Cerebelum 9 R | -0.306 (0.704) | -0.387 (0.227) | 3.450 | 0.001 |
| Amygdala L - Vermis 1 2 | -0.217 (0.76) | 1.384 (0.244) | -63.395 | < 0.001 |
| Amygdala R - Cuneus L | -0.406 (0.662) | 0.007 (0.269) | -18.273 | < 0.001 |
| Amygdala R - Cuneus R | -0.245 (0.702) | -0.161 (0.233) | -3.590 | 0.001 |
| Amygdala R - Occipital Sup L | -0.375 (0.74) | 0.673 (0.255) | -42.375 | < 0.001 |
| Amygdala R - Pallidum L | -0.561 (0.82) | 0.537 (0.266) | -40.246 | < 0.001 |
| Amygdala R - Heschl L | 0.908 (0.837) | -0.723 (0.228) | 59.469 | < 0.001 |
| Amygdala R - Temporal Sup L | 0.318 (0.745) | -0.515 (0.328) | 32.342 | < 0.001 |
| Amygdala R - Temporal Inf R | -0.577 (0.661) | -1.026 (0.281) | 19.766 | < 0.001 |
| Calcarine L - Occipital Mid L | 0.536 (0.74) | 1.239 (0.243) | -28.565 | < 0.001 |
| Calcarine L - Occipital Mid R | 0.059 (0.759) | 0.153 (0.247) | -3.730 | < 0.001 |
| Calcarine L - Occipital Inf L | 0.209 (0.763) | 0.593 (0.282) | -14.952 | < 0.001 |
| Calcarine L - Postcentral L | -0.536 (0.838) | -2.185 (0.223) | 60.122 | < 0.001 |
| Calcarine L - Cerebelum 4 5 L | 1.017 (0.763) | 0.027 (0.315) | 37.921 | < 0.001 |
| Calcarine L - Cerebelum 6 L | 0.354 (0.795) | -0.552 (0.258) | 34.281 | < 0.001 |
| Calcarine L - Vermis 3 | 0.02 (0.755) | 0.777 (0.291) | -29.596 | < 0.001 |
| Calcarine L - Vermis 7 | -0.73 (0.804) | -0.257 (0.247) | -17.779 | < 0.001 |
| Calcarine R - Postcentral L | -0.264 (0.778) | -2.565 (0.218) | 90.063 | < 0.001 |
| Calcarine R - Postcentral R | 0.105 (0.785) | -2.415 (0.207) | 98.174 | < 0.001 |
| Calcarine R - Parietal Sup L | 0.301 (0.804) | -1.274 (0.249) | 59.155 | < 0.001 |
| Calcarine R - Cerebelum 4 5 L | 0.279 (0.685) | -1.004 (0.279) | 54.891 | < 0.001 |
| Calcarine R - Cerebelum 4 5 R | 0.968 (0.784) | -0.278 (0.255) | 47.755 | < 0.001 |
| Calcarine R - Vermis 3 | 0.891 (0.733) | 1.385 (0.311) | -19.599 | < 0.001 |
| Cuneus L - Postcentral L | -0.184 (0.793) | -2.149 (0.226) | 75.411 | < 0.001 |
| Cuneus L - Postcentral R | -0.257 (0.796) | -2.149 (0.237) | 72.057 | < 0.001 |
| Cuneus L - Parietal Sup L | 0.118 (0.78) | -1.825 (0.276) | 74.283 | < 0.001 |
| Cuneus L - Parietal Sup R | -0.307 (0.699) | -2.2 (0.259) | 80.283 | < 0.001 |
| Cuneus L - Angular L | 0.021 (0.732) | 1.3 (0.31) | -50.863 | < 0.001 |
| Cuneus L - Paracentral Lobule L | -0.434 (0.734) | -1.651 (0.267) | 49.314 | < 0.001 |
| Cuneus L - Paracentral Lobule R | -0.329 (0.771) | -1.946 (0.232) | 63.485 | < 0.001 |
| Cuneus L - Temporal Pole Sup R | 0.097 (0.827) | -1.793 (0.241) | 69.425 | < 0.001 |
| Cuneus L - Vermis 10 | -0.274 (0.814) | -1.075 (0.219) | 30.019 | < 0.001 |
| Cuneus R - Occipital Sup L | -0.103 (0.862) | -1.487 (0.254) | 48.688 | < 0.001 |
| Cuneus R - Postcentral R | -0.452 (0.804) | -2.388 (0.243) | 72.900 | < 0.001 |
| Cuneus R - Parietal Sup L | -0.233 (0.81) | -1.622 (0.247) | 51.879 | < 0.001 |
| Cuneus R - Parietal Sup R | -0.427 (0.783) | -1.859 (0.254) | 55.018 | < 0.001 |
| Cuneus R - Cerebelum 3 R | -0.533 (0.694) | -1.601 (0.276) | 45.249 | < 0.001 |
| Cuneus R - Cerebelum 4 5 L | 0.847 (0.7) | -0.731 (0.251) | 67.133 | < 0.001 |
| Cuneus R - Vermis 3 | -0.269 (0.73) | -0.626 (0.248) | 14.615 | < 0.001 |
| Cuneus R - Vermis 4 5 | 1.158 (0.818) | -0.105 (0.228) | 47.046 | < 0.001 |
| Lingual L - Vermis 3 | 0.279 (0.779) | 1.292 (0.328) | -37.882 | < 0.001 |
| Lingual L - Vermis 6 | 0.944 (0.771) | 0.641 (0.284) | 11.677 | < 0.001 |
| Lingual L - Vermis 9 | 0.652 (0.733) | 0.486 (0.232) | 6.819 | < 0.001 |
| Lingual R - Occipital Sup R | -0.031 (0.851) | -0.444 (0.275) | 14.589 | < 0.001 |
| Lingual R - Occipital Inf L | 0.291 (0.807) | 0.073 (0.31) | 7.951 | < 0.001 |
| Lingual R - Postcentral L | -0.43 (0.776) | -2.879 (0.215) | 96.182 | < 0.001 |
| Lingual R - Cerebelum Crus1 R | 0.861 (0.779) | 0.114 (0.224) | 29.143 | < 0.001 |
| Lingual R - Cerebelum 4 5 L | 1.038 (0.771) | 1.254 (0.324) | -8.177 | < 0.001 |
| Lingual R - Cerebelum 6 R | 0.764 (0.837) | 0.573 (0.302) | 6.810 | < 0.001 |
| Lingual R - Vermis 3 | 0.381 (0.75) | 1.613 (0.354) | -46.994 | < 0.001 |
| Lingual R - Vermis 4 5 | 1.191 (0.815) | 0.75 (0.256) | 16.337 | < 0.001 |
| Lingual R - Vermis 6 | 0.473 (0.781) | 0.396 (0.309) | 2.894 | 0.006 |
| Lingual R - Vermis 9 | 0.918 (0.76) | 0.429 (0.241) | 19.392 | < 0.001 |
| Lingual R - Vermis 10 | -0.105 (0.816) | 0.848 (0.246) | -35.375 | < 0.001 |
| Occipital Sup L - Postcentral R | -0.07 (0.798) | -2.367 (0.233) | 87.378 | < 0.001 |
| Occipital Sup L - Temporal Pole Sup L | -0.764 (0.78) | -1.104 (0.219) | 13.276 | < 0.001 |
| Occipital Sup L - Temporal Pole Mid R | 0.039 (0.696) | -0.336 (0.254) | 16.027 | < 0.001 |
| Occipital Sup L - Vermis 10 | -0.249 (0.83) | -0.07 (0.243) | -6.574 | < 0.001 |
| Occipital Sup R - Postcentral R | -0.231 (0.82) | -2.018 (0.233) | 66.312 | < 0.001 |
| Occipital Sup R - Parietal Sup L | -0.605 (0.847) | -1.311 (0.247) | 25.326 | < 0.001 |
| Occipital Sup R - Paracentral Lobule R | -0.561 (0.822) | -1.612 (0.255) | 38.587 | < 0.001 |
| Occipital Sup R - Temporal Pole Sup L | -0.508 (0.78) | -1.071 (0.24) | 21.810 | < 0.001 |
| Occipital Sup R - Vermis 1 2 | 0.285 (0.895) | 1.207 (0.305) | -30.803 | < 0.001 |
| Occipital Sup R - Vermis 3 | 0.464 (0.703) | 0.398 (0.281) | 2.776 | 0.009 |
| Occipital Sup R - Vermis 4 5 | 0.509 (0.833) | -0.507 (0.245) | 37.002 | < 0.001 |
| Occipital Mid L - Postcentral L | -0.645 (0.841) | -3.347 (0.23) | 97.942 | < 0.001 |
| Occipital Mid L - Postcentral R | -0.894 (0.848) | -2.968 (0.213) | 75.033 | < 0.001 |
| Occipital Mid L - Parietal Sup L | -0.226 (0.847) | -2.577 (0.226) | 84.818 | < 0.001 |
| Occipital Mid L - Parietal Sup R | -0.718 (0.8) | -3.694 (0.248) | 112.400 | < 0.001 |
| Occipital Mid L - Angular L | 0.868 (0.799) | 1.344 (0.286) | -17.754 | < 0.001 |
| Occipital Mid L - Cerebelum 3 R | 0.143 (0.72) | -0.279 (0.231) | 17.675 | < 0.001 |
| Occipital Mid L - Cerebelum 4 5 L | 0.598 (0.825) | -0.145 (0.271) | 27.028 | < 0.001 |
| Occipital Mid L - Cerebelum 4 5 R | 0.506 (0.834) | 0.159 (0.233) | 12.692 | < 0.001 |
| Occipital Mid L - Cerebelum 6 L | -0.209 (0.81) | -0.334 (0.307) | 4.599 | < 0.001 |
| Occipital Mid L - Vermis 3 | 0.37 (0.697) | 0.671 (0.243) | -12.903 | < 0.001 |
| Occipital Mid L - Vermis 10 | 0.862 (0.855) | 0.074 (0.214) | 28.281 | < 0.001 |
| Occipital Mid R - Postcentral R | -0.743 (0.885) | -2.547 (0.237) | 62.243 | < 0.001 |
| Occipital Mid R - Parietal Sup R | -1.135 (0.795) | -2.214 (0.26) | 40.799 | < 0.001 |
| Occipital Mid R - Heschl L | 0.005 (0.913) | -0.69 (0.249) | 23.240 | < 0.001 |
| Occipital Mid R - Cerebelum Crus1 R | 0.322 (0.802) | -1.473 (0.283) | 66.712 | < 0.001 |
| Occipital Mid R - Cerebelum 4 5 L | 0.403 (0.787) | -0.977 (0.297) | 51.862 | < 0.001 |
| Occipital Mid R - Cerebelum 4 5 R | 0.913 (0.797) | -0.717 (0.236) | 62.025 | < 0.001 |
| Occipital Mid R - Vermis 3 | 0.776 (0.739) | -0.767 (0.205) | 63.660 | < 0.001 |
| Occipital Mid R - Vermis 4 5 | 0.532 (0.848) | -0.236 (0.212) | 27.788 | < 0.001 |
| Occipital Inf L - Cerebelum Crus1 L | 0.646 (0.791) | 0.027 (0.312) | 22.991 | < 0.001 |
| Occipital Inf L - Cerebelum 6 L | 1.142 (0.803) | 1.052 (0.353) | 3.233 | 0.002 |
| Occipital Inf L - Cerebelum 6 R | 1.067 (0.78) | 0.73 (0.28) | 12.852 | < 0.001 |
| Occipital Inf L - Vermis 3 | 0.673 (0.784) | 1.563 (0.325) | -33.154 | < 0.001 |
| Occipital Inf R - Temporal Mid R | 0.774 (0.799) | -0.351 (0.242) | 42.603 | < 0.001 |
| Occipital Inf R - Cerebelum 4 5 R | 1.152 (0.759) | 1.017 (0.333) | 5.136 | < 0.001 |
| Occipital Inf R - Cerebelum 6 R | 0.313 (0.796) | 1.362 (0.299) | -39.010 | < 0.001 |
| Occipital Inf R - Vermis 4 5 | 0.735 (0.772) | 0.067 (0.306) | 25.470 | < 0.001 |
| Occipital Inf R - Vermis 10 | -0.474 (0.84) | 1.757 (0.249) | -80.554 | < 0.001 |
| Fusiform L - Postcentral L | -1.123 (0.752) | -2.239 (0.226) | 44.982 | < 0.001 |
| Fusiform L - Postcentral R | -0.877 (0.743) | -1.982 (0.231) | 44.913 | < 0.001 |
| Fusiform L - Parietal Sup L | -0.845 (0.799) | -1.164 (0.226) | 12.156 | < 0.001 |
| Fusiform L - Parietal Sup R | 0.056 (0.763) | -1.088 (0.258) | 44.910 | < 0.001 |
| Fusiform L - Cerebelum 8 L | 0.413 (0.689) | -1.435 (0.315) | 77.118 | < 0.001 |
| Fusiform L - Cerebelum 10 L | 0.357 (0.745) | 1.406 (0.242) | -42.329 | < 0.001 |
| Fusiform R - Postcentral R | -0.75 (0.698) | -1.61 (0.24) | 36.846 | < 0.001 |
| Fusiform R - Paracentral Lobule R | -1.206 (0.742) | -0.496 (0.25) | -28.675 | < 0.001 |
| Fusiform R - Pallidum R | 0.518 (0.757) | 1.618 (0.293) | -42.875 | < 0.001 |
| Fusiform R - Cerebelum 3 L | 0.948 (0.832) | 0.677 (0.28) | 9.780 | < 0.001 |
| Fusiform R - Cerebelum 4 5 L | 1.136 (0.786) | -0.126 (0.284) | 47.757 | < 0.001 |
| Fusiform R - Cerebelum 10 L | 0.78 (0.744) | 1.508 (0.294) | -28.804 | < 0.001 |
| Fusiform R - Vermis 3 | 0.919 (0.778) | 1.175 (0.272) | -9.840 | < 0.001 |
| Fusiform R - Vermis 4 5 | 0.982 (0.807) | -0.359 (0.258) | 50.026 | < 0.001 |
| Postcentral L - Pallidum R | 0.645 (0.763) | 0.752 (0.226) | -4.257 | < 0.001 |
| Postcentral R - Parietal Inf R | 0.672 (0.807) | 1.948 (0.279) | -47.267 | < 0.001 |
| Parietal Sup L - Temporal Mid L | 0.153 (0.792) | -0.204 (0.226) | 13.721 | < 0.001 |
| Parietal Sup L - Vermis 10 | 0.498 (0.846) | 0.324 (0.266) | 6.189 | < 0.001 |
| Parietal Sup R - Putamen R | 0.821 (0.757) | 0.331 (0.259) | 19.344 | < 0.001 |
| Parietal Sup R - Temporal Pole Sup L | -0.28 (0.747) | -0.889 (0.234) | 24.609 | < 0.001 |
| Parietal Sup R - Temporal Inf R | -0.793 (0.745) | -1.38 (0.246) | 23.689 | < 0.001 |
| Parietal Sup R - Cerebelum 6 L | -0.026 (0.854) | 0.307 (0.283) | -11.692 | < 0.001 |
| Parietal Inf L - Angular L | 0.142 (0.829) | 0.447 (0.239) | -11.178 | < 0.001 |
| Parietal Inf L - Temporal Inf R | -1.06 (0.686) | -1.958 (0.245) | 38.996 | < 0.001 |
| Parietal Inf L - Vermis 8 | 0.949 (0.788) | 2.394 (0.284) | -54.514 | < 0.001 |
| Parietal Inf R - Pallidum L | 0.008 (0.865) | 2.317 (0.324) | -79.094 | < 0.001 |
| SupraMarginal L - Temporal Mid R | -0.631 (0.718) | 0.607 (0.265) | -51.139 | < 0.001 |
| SupraMarginal L - Temporal Inf R | -0.707 (0.777) | -0.593 (0.232) | -4.450 | < 0.001 |
| SupraMarginal R - Heschl R | 0.523 (0.843) | 0.004 (0.261) | 18.582 | < 0.001 |
| SupraMarginal R - Temporal Sup L | -0.627 (0.757) | 0.199 (0.25) | -32.739 | < 0.001 |
| SupraMarginal R - Temporal Mid L | 0.212 (0.707) | 1.515 (0.267) | -54.562 | < 0.001 |
| SupraMarginal R - Temporal Mid R | -0.735 (0.749) | 0.697 (0.286) | -56.499 | < 0.001 |
| Angular L - Precuneus R | 0.106 (0.869) | 1.457 (0.23) | -47.527 | < 0.001 |
| Angular L - Cerebelum Crus2 R | -0.607 (0.791) | -1.255 (0.324) | 23.988 | < 0.001 |
| Angular R - Temporal Pole Sup L | 0.278 (0.695) | 0.841 (0.253) | -24.042 | < 0.001 |
| Angular R - Temporal Pole Sup R | 0.994 (0.765) | 0.67 (0.248) | 12.750 | < 0.001 |
| Precuneus L - Putamen R | 0.096 (0.683) | -0.814 (0.215) | 40.172 | < 0.001 |
| Precuneus L - Vermis 10 | -0.029 (0.841) | -0.514 (0.246) | 17.526 | < 0.001 |
| Caudate R - Temporal Pole Mid L | 0.145 (0.831) | 2.076 (0.251) | -70.332 | < 0.001 |
| Caudate R - Cerebelum 8 L | -1.027 (0.859) | 1.077 (0.29) | -73.428 | < 0.001 |
| Caudate R - Cerebelum 8 R | -0.452 (0.841) | 1.406 (0.28) | -66.292 | < 0.001 |
| Putamen L - Putamen R | -0.456 (0.754) | -0.919 (0.291) | 18.131 | < 0.001 |
| Thalamus L - Cerebelum 4 5 L | -0.592 (0.748) | -0.16 (0.28) | -17.115 | < 0.001 |
| Heschl L - Temporal Sup L | 0.535 (0.833) | -0.07 (0.267) | 21.879 | < 0.001 |
| Temporal Sup L - Cerebelum 4 5 L | 0.403 (0.843) | 1.481 (0.249) | -38.761 | < 0.001 |
| Temporal Sup L - Cerebelum 6 L | 0.631 (0.758) | 0.829 (0.246) | -7.891 | < 0.001 |
| Temporal Sup R - Vermis 9 | 0.372 (0.759) | -0.09 (0.226) | 18.440 | < 0.001 |
| Temporal Pole Sup R - Temporal Mid L | 0.608 (0.69) | -0.395 (0.271) | 42.800 | < 0.001 |
| Temporal Mid R - Cerebelum 4 5 L | 0.617 (0.771) | 1.368 (0.335) | -28.224 | < 0.001 |
| Temporal Mid R - Vermis 6 | 0.91 (0.779) | 1.196 (0.292) | -10.857 | < 0.001 |
| Cerebelum Crus1 L - Cerebelum 6 L | 1.009 (0.745) | 0.943 (0.284) | 2.624 | 0.014 |
| Cerebelum Crus1 R - Cerebelum 7b R | 0.167 (0.792) | -0.656 (0.337) | 30.251 | < 0.001 |
| Cerebelum 4 5 R - Vermis 4 5 | 0.925 (0.724) | 1.128 (0.25) | -8.391 | < 0.001 |
| Cerebelum 4 5 R - Vermis 7 | 0.333 (0.782) | -1.138 (0.242) | 56.858 | < 0.001 |
| Vermis 1 2 - Vermis 7 | -0.526 (0.768) | 0.469 (0.336) | -37.549 | < 0.001 |
| Vermis 3 - Vermis 6 | -0.316 (0.725) | -0.223 (0.263) | -3.844 | < 0.001 |
| Vermis 4 5 - Vermis 6 | 0.502 (0.801) | -0.698 (0.242) | 45.359 | < 0.001 |
| Vermis 4 5 - Vermis 7 | -0.159 (0.736) | -0.987 (0.276) | 33.316 | < 0.001 |
| Vermis 6 - Vermis 7 | 0.31 (0.842) | -0.093 (0.318) | 14.145 | < 0.001 |
| Precentral L - Calcarine L | -0.448 (0.87) | -2.315 (0.208) | 65.996 | < 0.001 |
| Precentral L - Calcarine R | -0.507 (0.806) | -2.191 (0.241) | 63.258 | < 0.001 |
| Precentral L - Cuneus R | -0.554 (0.838) | -2.62 (0.242) | 74.896 | < 0.001 |
| Precentral L - Occipital Sup R | -0.621 (0.864) | -2.638 (0.23) | 71.305 | < 0.001 |
| Precentral L - Occipital Mid R | -0.49 (0.888) | -3.29 (0.289) | 94.823 | < 0.001 |
| Precentral L - Occipital Inf L | -0.86 (0.862) | -2.927 (0.266) | 72.437 | < 0.001 |
| Precentral L - Occipital Inf R | -0.949 (0.809) | -2.021 (0.274) | 39.658 | < 0.001 |
| Precentral L - Parietal Sup R | 0.722 (0.86) | -2.185 (0.311) | 100.520 | < 0.001 |
| Precentral L - Pallidum L | -0.917 (0.754) | 2.247 (0.295) | -123.640 | < 0.001 |
| Precentral R - Frontal Inf Oper L | -0.388 (0.817) | -2.862 (0.247) | 91.682 | < 0.001 |
| Precentral R - Frontal Inf Oper R | 0.204 (0.822) | -1.966 (0.262) | 79.524 | < 0.001 |
| Precentral R - Frontal Inf Tri L | -0.808 (0.762) | -2.738 (0.215) | 77.043 | < 0.001 |
| Precentral R - Frontal Inf Tri R | 0.224 (0.879) | -2.089 (0.199) | 81.182 | < 0.001 |
| Precentral R - Calcarine L | -0.345 (0.812) | -2.454 (0.207) | 79.596 | < 0.001 |
| Precentral R - Lingual L | -0.898 (0.745) | -2.168 (0.201) | 52.074 | < 0.001 |
| Precentral R - Lingual R | -0.739 (0.765) | -2.542 (0.209) | 71.900 | < 0.001 |
| Precentral R - Occipital Inf L | -0.543 (0.789) | -2.994 (0.221) | 94.627 | < 0.001 |
| Precentral R - Fusiform R | -1.051 (0.741) | -2.082 (0.229) | 42.006 | < 0.001 |
| Precentral R - Temporal Pole Sup R | -0.525 (0.771) | -2.04 (0.199) | 60.179 | < 0.001 |
| Precentral R - Cerebelum Crus1 L | 0.871 (0.879) | 1.912 (0.275) | -35.731 | < 0.001 |
| Precentral R - Cerebelum 9 L | 0.888 (0.811) | 2.279 (0.283) | -51.201 | < 0.001 |
| Precentral R - Cerebelum 10 R | -0.665 (0.902) | 1.907 (0.3) | -85.542 | < 0.001 |
| Precentral R - Vermis 8 | 0.782 (0.684) | 1.981 (0.257) | -51.883 | < 0.001 |
| Frontal Sup L - Cingulum Post L | -0.429 (0.754) | -2.444 (0.226) | 80.889 | < 0.001 |
| Frontal Sup L - Angular L | -0.858 (0.797) | -1.965 (0.251) | 41.868 | < 0.001 |
| Frontal Sup L - Vermis 1 2 | -0.127 (0.724) | -2.08 (0.237) | 81.126 | < 0.001 |
| Frontal Sup L - Vermis 9 | -0.444 (0.78) | 1.916 (0.249) | -91.169 | < 0.001 |
| Frontal Sup R - Cingulum Post L | -0.241 (0.693) | -3.007 (0.249) | 118.689 | < 0.001 |
| Frontal Sup R - Cingulum Post R | -0.373 (0.711) | -2.793 (0.256) | 101.345 | < 0.001 |
| Frontal Sup R - Hippocampus L | -0.515 (0.838) | 2.33 (0.294) | -101.267 | < 0.001 |
| Frontal Sup R - Calcarine L | -1.046 (0.756) | -1.925 (0.254) | 34.879 | < 0.001 |
| Frontal Sup R - Lingual L | -0.836 (0.723) | -1.936 (0.238) | 45.689 | < 0.001 |
| Frontal Sup R - Occipital Inf L | -0.501 (0.779) | -2.088 (0.209) | 62.182 | < 0.001 |
| Frontal Sup R - Parietal Inf R | -0.528 (0.819) | -1.933 (0.251) | 51.838 | < 0.001 |
| Frontal Sup R - Angular L | -0.657 (0.785) | -1.983 (0.218) | 51.504 | < 0.001 |
| Frontal Sup R - Angular R | -0.738 (0.844) | -2.332 (0.24) | 57.442 | < 0.001 |
| Frontal Sup R - Precuneus L | -0.702 (0.759) | -2.107 (0.243) | 55.773 | < 0.001 |
| Frontal Sup R - Precuneus R | -1.072 (0.767) | -1.92 (0.261) | 33.080 | < 0.001 |
| Frontal Sup R - Caudate L | 0.263 (0.811) | 2.251 (0.276) | -73.392 | < 0.001 |
| Frontal Sup R - Caudate R | -0.763 (0.744) | 2.066 (0.293) | -111.839 | < 0.001 |
| Frontal Sup R - Pallidum R | -0.214 (0.829) | 1.987 (0.38) | -76.331 | < 0.001 |
| Frontal Sup R - Cerebelum 4 5 R | 0.253 (0.714) | -2.135 (0.275) | 98.612 | < 0.001 |
| Frontal Sup Orb L - Frontal Mid Orb R | 0.595 (0.801) | 2.051 (0.276) | -54.382 | < 0.001 |
| Frontal Sup Orb L - Parietal Inf R | 0.31 (0.753) | 1.941 (0.315) | -63.165 | < 0.001 |
| Frontal Sup Orb L - Thalamus L | 0.189 (0.745) | 2.2 (0.34) | -77.644 | < 0.001 |
| Frontal Sup Orb R - Frontal Inf Tri L | -0.307 (0.806) | -2.187 (0.234) | 70.800 | < 0.001 |
| Frontal Sup Orb R - Vermis 10 | 0.185 (0.85) | 2.884 (0.316) | -94.160 | < 0.001 |
| Frontal Mid L - Cingulum Post L | -0.99 (0.732) | -2.777 (0.236) | 73.512 | < 0.001 |
| Frontal Mid L - Cingulum Post R | -0.754 (0.705) | -2.28 (0.205) | 65.746 | < 0.001 |
| Frontal Mid L - Calcarine L | -0.779 (0.762) | -1.947 (0.268) | 45.756 | < 0.001 |
| Frontal Mid L - Angular R | -0.627 (0.817) | -2.208 (0.241) | 58.712 | < 0.001 |
| Frontal Mid L - Heschl R | 0.501 (0.679) | 2.001 (0.397) | -60.365 | < 0.001 |
| Frontal Mid L - Cerebelum Crus1 R | -0.212 (0.754) | -2.098 (0.279) | 74.212 | < 0.001 |
| Frontal Mid L - Cerebelum 7b R | -1.097 (0.774) | -2.248 (0.218) | 45.272 | < 0.001 |
| Frontal Mid R - Cingulum Post R | -0.769 (0.698) | -2.195 (0.23) | 61.324 | < 0.001 |
| Frontal Mid R - Hippocampus L | 0.048 (0.792) | 1.903 (0.275) | -69.942 | < 0.001 |
| Frontal Mid R - Thalamus R | 0.218 (0.748) | 2.158 (0.427) | -71.213 | < 0.001 |
| Frontal Mid R - Cerebelum 4 5 R | 0.096 (0.69) | -1.986 (0.22) | 90.861 | < 0.001 |
| Frontal Mid Orb L - Thalamus L | 0.894 (0.712) | 2.192 (0.29) | -53.393 | < 0.001 |
| Frontal Mid Orb L - Temporal Pole Sup R | 1.058 (0.76) | 2.003 (0.255) | -37.289 | < 0.001 |
| Frontal Mid Orb R - Occipital Sup R | 0.201 (0.78) | 2.097 (0.284) | -72.220 | < 0.001 |
| Frontal Mid Orb R - Postcentral L | -0.462 (0.884) | 2.489 (0.291) | -100.260 | < 0.001 |
| Frontal Mid Orb R - Thalamus L | 0.645 (0.681) | 2.471 (0.269) | -78.903 | < 0.001 |
| Frontal Mid Orb R - Vermis 3 | 1.133 (0.776) | 2.363 (0.331) | -46.105 | < 0.001 |
| Frontal Inf Oper L - Frontal Inf Orb R | -0.231 (0.772) | -2.062 (0.223) | 72.100 | < 0.001 |
| Frontal Inf Oper L - Parietal Inf L | 0.181 (0.887) | -2.331 (0.252) | 86.219 | < 0.001 |
| Frontal Inf Oper L - Temporal Mid L | -0.878 (0.751) | -2.395 (0.299) | 59.339 | < 0.001 |
| Frontal Inf Oper L - Temporal Inf R | -1.115 (0.771) | -1.945 (0.259) | 32.279 | < 0.001 |
| Frontal Inf Oper L - Cerebelum Crus1 R | 0.095 (0.856) | -1.922 (0.226) | 72.045 | < 0.001 |
| Frontal Inf Oper L - Cerebelum 6 L | -0.54 (0.747) | -2.118 (0.247) | 63.404 | < 0.001 |
| Frontal Inf Oper R - Thalamus R | -0.379 (0.79) | 1.992 (0.31) | -88.399 | < 0.001 |
| Frontal Inf Tri L - Frontal Inf Orb R | -0.472 (0.811) | -2.208 (0.238) | 64.962 | < 0.001 |
| Frontal Inf Tri L - Frontal Med Orb R | 0.244 (0.779) | -2.163 (0.253) | 93.007 | < 0.001 |
| Frontal Inf Tri L - Parietal Inf L | -0.369 (0.843) | -2.125 (0.265) | 62.826 | < 0.001 |
| Frontal Inf Tri L - Putamen R | 0.477 (0.774) | -1.952 (0.254) | 94.360 | < 0.001 |
| Frontal Inf Tri L - Cerebelum 6 L | -0.598 (0.753) | -1.986 (0.216) | 56.023 | < 0.001 |
| Frontal Inf Tri R - Supp Motor Area L | 0.113 (0.885) | -2.217 (0.266) | 79.698 | < 0.001 |
| Frontal Inf Orb L - Vermis 4 5 | 1.148 (0.9) | 2.111 (0.362) | -31.384 | < 0.001 |
| Frontal Inf Orb R - Lingual R | 1.054 (0.784) | 2.178 (0.325) | -41.893 | < 0.001 |
| Frontal Inf Orb R - Thalamus L | 0.782 (0.744) | 1.908 (0.345) | -43.395 | < 0.001 |
| Frontal Inf Orb R - Thalamus R | 0.799 (0.762) | 2.055 (0.298) | -48.544 | < 0.001 |
| Rolandic Oper L - Supp Motor Area R | -0.437 (0.801) | -2.188 (0.249) | 65.970 | < 0.001 |
| Rolandic Oper L - Amygdala R | 0.949 (0.786) | -1.969 (0.209) | 113.379 | < 0.001 |
| Rolandic Oper L - Heschl L | 0.105 (0.863) | -2.264 (0.232) | 83.839 | < 0.001 |
| Rolandic Oper L - Temporal Pole Sup R | 0.294 (0.733) | -3.292 (0.185) | 150.011 | < 0.001 |
| Rolandic Oper R - Rectus R | -0.868 (0.704) | 2.115 (0.303) | -122.983 | < 0.001 |
| Rolandic Oper R - Cingulum Ant L | -0.655 (0.718) | -2.306 (0.219) | 69.584 | < 0.001 |
| Rolandic Oper R - Temporal Pole Sup R | 0.531 (0.863) | -2.381 (0.219) | 103.410 | < 0.001 |
| Rolandic Oper R - Vermis 3 | -0.685 (0.729) | 1.932 (0.274) | -106.263 | < 0.001 |
| Supp Motor Area L - Occipital Sup R | 0.287 (0.812) | -2.085 (0.218) | 89.154 | < 0.001 |
| Supp Motor Area L - Occipital Mid L | -0.013 (0.835) | -2.333 (0.234) | 84.644 | < 0.001 |
| Supp Motor Area L - Occipital Mid R | -0.034 (0.851) | -2.259 (0.249) | 79.385 | < 0.001 |
| Supp Motor Area L - Temporal Sup R | 0.001 (0.857) | -2.02 (0.235) | 71.910 | < 0.001 |
| Supp Motor Area R - Occipital Sup L | 0.183 (0.786) | -2.062 (0.233) | 86.648 | < 0.001 |
| Supp Motor Area R - SupraMarginal R | -0.807 (0.782) | -2.01 (0.262) | 46.149 | < 0.001 |
| Supp Motor Area R - Angular R | -0.471 (0.798) | -2.119 (0.221) | 62.926 | < 0.001 |
| Supp Motor Area R - Cerebelum Crus1 L | 0.079 (0.887) | 2.802 (0.295) | -92.118 | < 0.001 |
| Supp Motor Area R - Vermis 7 | -0.208 (0.747) | 2.227 (0.287) | -96.194 | < 0.001 |
| Olfactory L - Temporal Pole Sup L | 0.243 (0.764) | -1.933 (0.296) | 83.965 | < 0.001 |
| Olfactory L - Temporal Pole Sup R | 0.414 (0.679) | -2.591 (0.305) | 127.692 | < 0.001 |
| Olfactory L - Vermis 4 5 | 0.583 (0.778) | 3.014 (0.314) | -91.660 | < 0.001 |
| Frontal Sup Medial L - Cingulum Post L | -0.711 (0.731) | -2.592 (0.235) | 77.486 | < 0.001 |
| Frontal Sup Medial L - Cingulum Post R | -0.735 (0.699) | -2.164 (0.234) | 61.294 | < 0.001 |
| Frontal Sup Medial L - Pallidum R | -0.669 (0.735) | 2.531 (0.357) | -123.878 | < 0.001 |
| Frontal Sup Medial L - Vermis 8 | 0.219 (0.636) | 2.357 (0.267) | -97.997 | < 0.001 |
| Frontal Sup Medial L - Vermis 9 | -0.494 (0.722) | 2.985 (0.282) | -141.867 | < 0.001 |
| Frontal Sup Medial R - Temporal Mid R | -1.138 (0.756) | -2.433 (0.24) | 51.649 | < 0.001 |
| Frontal Sup Medial R - Cerebelum 10 L | 0.573 (0.838) | 2.064 (0.309) | -52.738 | < 0.001 |
| Frontal Sup Medial R - Vermis 3 | -0.558 (0.749) | 2.087 (0.289) | -104.194 | < 0.001 |
| Frontal Sup Medial R - Vermis 9 | -0.199 (0.845) | 2.208 (0.309) | -84.590 | < 0.001 |
| Frontal Med Orb L - Fusiform R | -0.477 (0.711) | -2.105 (0.209) | 69.501 | < 0.001 |
| Frontal Med Orb L - Temporal Pole Sup R | -0.057 (0.721) | -1.954 (0.312) | 76.360 | < 0.001 |
| Frontal Med Orb L - Cerebelum Crus2 L | -0.096 (0.796) | -2.276 (0.251) | 82.588 | < 0.001 |
| Frontal Med Orb R - Cingulum Post L | 0.293 (0.691) | -2.099 (0.404) | 94.563 | < 0.001 |
| Frontal Med Orb R - Cerebelum Crus2 L | -0.164 (0.842) | -2.023 (0.271) | 66.460 | < 0.001 |
| Rectus L - SupraMarginal L | 0.388 (0.821) | 1.91 (0.222) | -56.601 | < 0.001 |
| Rectus L - Temporal Pole Mid R | -0.429 (0.722) | -2.017 (0.278) | 64.917 | < 0.001 |
| Rectus L - Cerebelum 9 R | -0.215 (0.671) | 1.891 (0.371) | -86.898 | < 0.001 |
| Rectus L - Vermis 10 | 0.581 (0.893) | 2.383 (0.278) | -60.887 | < 0.001 |
| Rectus R - Insula L | 0.405 (0.868) | 3.812 (0.275) | -118.336 | < 0.001 |
| Rectus R - Insula R | 0.286 (0.851) | 3.769 (0.309) | -121.619 | < 0.001 |
| Rectus R - Lingual R | 0.097 (0.793) | 2.004 (0.309) | -70.860 | < 0.001 |
| Rectus R - Parietal Inf R | 0.795 (0.737) | 2.095 (0.322) | -51.155 | < 0.001 |
| Rectus R - SupraMarginal L | 0.029 (0.727) | 2.308 (0.314) | -90.975 | < 0.001 |
| Insula L - Cuneus L | -0.167 (0.786) | 2.08 (0.233) | -86.737 | < 0.001 |
| Insula L - Occipital Sup R | 0.132 (0.736) | 2.09 (0.318) | -77.250 | < 0.001 |
| Insula L - Fusiform R | 0.206 (0.779) | 2.116 (0.284) | -72.873 | < 0.001 |
| Insula L - Parietal Sup L | -0.352 (0.815) | 2.273 (0.317) | -94.954 | < 0.001 |
| Insula L - Parietal Sup R | -0.935 (0.796) | 2.335 (0.316) | -120.723 | < 0.001 |
| Insula L - Parietal Inf R | 0.462 (0.792) | 1.938 (0.424) | -51.977 | < 0.001 |
| Insula L - Cerebelum 6 R | -0.046 (0.801) | 2.67 (0.273) | -101.539 | < 0.001 |
| Insula R - Lingual R | -0.167 (0.788) | 2.469 (0.269) | -100.130 | < 0.001 |
| Cingulum Ant R - Temporal Sup L | -0.614 (0.762) | -2.129 (0.227) | 60.249 | < 0.001 |
| Cingulum Mid L - Calcarine L | 0.167 (0.735) | -2.602 (0.201) | 114.951 | < 0.001 |
| Cingulum Mid L - Cuneus R | -0.295 (0.768) | -2.319 (0.242) | 79.498 | < 0.001 |
| Cingulum Mid L - Lingual R | -0.44 (0.745) | -2.037 (0.248) | 64.309 | < 0.001 |
| Cingulum Mid L - Occipital Sup L | 0.496 (0.744) | -2.264 (0.208) | 113.037 | < 0.001 |
| Cingulum Mid L - Occipital Sup R | -0.038 (0.77) | -2.839 (0.193) | 111.617 | < 0.001 |
| Cingulum Mid L - Occipital Mid L | -0.422 (0.799) | -2.327 (0.22) | 72.663 | < 0.001 |
| Cingulum Mid L - Temporal Sup R | 0.102 (0.849) | -2.633 (0.238) | 98.114 | < 0.001 |
| Cingulum Mid L - Temporal Pole Sup R | -0.333 (0.806) | -2.176 (0.236) | 69.358 | < 0.001 |
| Cingulum Mid L - Cerebelum 4 5 R | -0.371 (0.759) | -1.997 (0.205) | 65.393 | < 0.001 |
| Cingulum Mid R - Hippocampus L | -0.002 (0.764) | 2.061 (0.284) | -80.009 | < 0.001 |
| Cingulum Mid R - Occipital Mid L | -0.342 (0.82) | -2.225 (0.22) | 70.165 | < 0.001 |
| Cingulum Mid R - SupraMarginal L | -0.267 (0.866) | -2.679 (0.231) | 85.079 | < 0.001 |
| Cingulum Mid R - Temporal Sup L | -0.859 (0.842) | -1.988 (0.22) | 41.038 | < 0.001 |
| Cingulum Mid R - Temporal Pole Sup L | 0.068 (0.749) | -2.027 (0.257) | 83.734 | < 0.001 |
| Cingulum Post L - Fusiform R | 0.145 (0.678) | -1.986 (0.223) | 94.415 | < 0.001 |
| Cingulum Post L - Cerebelum Crus1 R | -0.141 (0.75) | -2.743 (0.229) | 104.960 | < 0.001 |
| Cingulum Post L - Cerebelum Crus2 L | -0.611 (0.791) | -2.844 (0.251) | 85.053 | < 0.001 |
| Cingulum Post L - Cerebelum 8 R | -0.147 (0.73) | -2.355 (0.222) | 91.491 | < 0.001 |
| Cingulum Post L - Cerebelum 9 R | -0.695 (0.817) | -2.692 (0.285) | 73.025 | < 0.001 |
| Cingulum Post R - Temporal Pole Mid L | 0.249 (0.701) | 2.116 (0.331) | -76.150 | < 0.001 |
| Cingulum Post R - Cerebelum Crus1 R | 0.431 (0.796) | -1.969 (0.227) | 91.757 | < 0.001 |
| Hippocampus L - SupraMarginal L | -0.252 (0.771) | 2.344 (0.386) | -95.266 | < 0.001 |
| Hippocampus L - Vermis 1 2 | 0.565 (0.658) | 2.012 (0.325) | -62.309 | < 0.001 |
| Hippocampus L - Vermis 3 | 0.111 (0.663) | 2.144 (0.335) | -86.500 | < 0.001 |
| Hippocampus R - Amygdala L | 0.492 (0.811) | -2.332 (0.266) | 104.680 | < 0.001 |
| Hippocampus R - Parietal Inf R | 0.041 (0.872) | 2.005 (0.345) | -66.228 | < 0.001 |
| Hippocampus R - SupraMarginal L | 0.739 (0.829) | 2.289 (0.28) | -56.001 | < 0.001 |
| Hippocampus R - Angular R | -0.142 (0.743) | 2.352 (0.288) | -98.930 | < 0.001 |
| ParaHippocampal L - Temporal Pole Sup L | 1.058 (0.789) | -2.224 (0.275) | 124.216 | < 0.001 |
| ParaHippocampal R - Amygdala R | 0.47 (0.784) | -2.156 (0.252) | 100.889 | < 0.001 |
| ParaHippocampal R - Caudate R | 0.139 (0.834) | 2.059 (0.254) | -69.635 | < 0.001 |
| Amygdala L - Cerebelum 7b R | -0.61 (0.788) | 2.044 (0.263) | -101.025 | < 0.001 |
| Amygdala R - Temporal Pole Mid R | 0.234 (0.784) | -2.93 (0.226) | 122.647 | < 0.001 |
| Amygdala R - Vermis 9 | -0.324 (0.735) | 1.934 (0.286) | -90.523 | < 0.001 |
| Calcarine L - Postcentral R | -0.163 (0.833) | -2.254 (0.216) | 76.871 | < 0.001 |
| Calcarine L - Parietal Sup R | 0.176 (0.774) | -1.94 (0.24) | 82.526 | < 0.001 |
| Calcarine L - Precuneus L | -0.133 (0.711) | -3.098 (0.285) | 122.435 | < 0.001 |
| Calcarine L - Precuneus R | -0.054 (0.752) | -2.749 (0.268) | 106.700 | < 0.001 |
| Calcarine L - Paracentral Lobule L | -0.655 (0.742) | -2.435 (0.245) | 72.070 | < 0.001 |
| Calcarine L - Cerebelum 9 R | 0.37 (0.689) | -2.35 (0.284) | 115.429 | < 0.001 |
| Calcarine R - Precuneus L | 0.02 (0.752) | -2.033 (0.262) | 81.509 | < 0.001 |
| Calcarine R - Paracentral Lobule L | -0.744 (0.744) | -2.419 (0.223) | 68.155 | < 0.001 |
| Calcarine R - Paracentral Lobule R | -0.4 (0.775) | -2.099 (0.228) | 66.545 | < 0.001 |
| Cuneus L - Precuneus R | 0.463 (0.677) | -2.226 (0.301) | 114.850 | < 0.001 |
| Cuneus L - Pallidum L | -0.216 (0.663) | 2.367 (0.254) | -115.059 | < 0.001 |
| Cuneus L - Cerebelum Crus1 R | -0.466 (0.893) | -2.285 (0.29) | 61.228 | < 0.001 |
| Cuneus L - Cerebelum 9 R | 0.18 (0.725) | -3.124 (0.226) | 137.663 | < 0.001 |
| Cuneus R - Lingual L | -0.367 (0.819) | -2.143 (0.242) | 65.783 | < 0.001 |
| Cuneus R - Postcentral L | -0.355 (0.806) | -2.908 (0.224) | 96.477 | < 0.001 |
| Cuneus R - Paracentral Lobule R | -0.505 (0.818) | -2.173 (0.252) | 61.649 | < 0.001 |
| Lingual L - Postcentral L | -1.012 (0.808) | -2.74 (0.199) | 65.648 | < 0.001 |
| Lingual L - Postcentral R | -0.478 (0.764) | -2.2 (0.195) | 69.037 | < 0.001 |
| Lingual L - Parietal Sup L | -0.323 (0.804) | -1.939 (0.244) | 60.824 | < 0.001 |
| Lingual L - Parietal Sup R | -0.415 (0.745) | -2.178 (0.223) | 71.734 | < 0.001 |
| Lingual L - Precuneus L | -0.128 (0.699) | -2.301 (0.243) | 92.807 | < 0.001 |
| Lingual L - Precuneus R | 0.196 (0.766) | -2.785 (0.253) | 116.936 | < 0.001 |
| Lingual L - Thalamus R | 0.531 (0.894) | -1.976 (0.258) | 85.234 | < 0.001 |
| Lingual L - Cerebelum 8 R | 0.06 (0.797) | -2.105 (0.241) | 82.198 | < 0.001 |
| Lingual R - Postcentral R | -0.094 (0.791) | -2.155 (0.232) | 79.091 | < 0.001 |
| Lingual R - Precuneus L | -0.069 (0.757) | -1.966 (0.242) | 75.480 | < 0.001 |
| Lingual R - Precuneus R | 0.216 (0.791) | -2.251 (0.251) | 94.043 | < 0.001 |
| Occipital Sup L - Postcentral L | 0.377 (0.801) | -2.651 (0.204) | 115.857 | < 0.001 |
| Occipital Sup L - Parietal Sup R | -0.472 (0.737) | -2.045 (0.237) | 64.248 | < 0.001 |
| Occipital Sup R - Occipital Inf L | -0.385 (0.816) | -1.957 (0.244) | 58.377 | < 0.001 |
| Occipital Sup R - Fusiform L | -0.965 (0.829) | -3.035 (0.23) | 76.091 | < 0.001 |
| Occipital Sup R - Fusiform R | -1.015 (0.776) | -2.245 (0.295) | 46.859 | < 0.001 |
| Occipital Sup R - Postcentral L | -0.073 (0.781) | -2.088 (0.209) | 78.808 | < 0.001 |
| Occipital Sup R - Angular L | 0.928 (0.656) | 1.919 (0.279) | -43.963 | < 0.001 |
| Occipital Sup R - Temporal Inf L | -0.867 (0.766) | -2.528 (0.237) | 65.505 | < 0.001 |
| Occipital Mid L - Occipital Inf R | -0.167 (0.816) | -2.03 (0.252) | 69.006 | < 0.001 |
| Occipital Mid L - Paracentral Lobule L | -0.603 (0.785) | -2.217 (0.224) | 62.573 | < 0.001 |
| Occipital Mid L - Paracentral Lobule R | -0.861 (0.776) | -2.066 (0.222) | 47.226 | < 0.001 |
| Occipital Mid R - Occipital Inf R | -0.003 (0.855) | -2.01 (0.278) | 70.611 | < 0.001 |
| Occipital Mid R - Fusiform L | -0.823 (0.784) | -2.233 (0.227) | 54.635 | < 0.001 |
| Occipital Mid R - Postcentral L | -0.649 (0.862) | -2.152 (0.24) | 53.129 | < 0.001 |
| Occipital Mid R - Putamen R | 0.553 (0.784) | 1.926 (0.381) | -49.807 | < 0.001 |
| Occipital Mid R - Temporal Inf L | -0.569 (0.722) | -2.422 (0.223) | 77.587 | < 0.001 |
| Occipital Inf L - Postcentral L | -0.703 (0.758) | -2.356 (0.219) | 66.210 | < 0.001 |
| Occipital Inf L - Postcentral R | -0.345 (0.757) | -2.164 (0.225) | 72.879 | < 0.001 |
| Occipital Inf L - Paracentral Lobule L | -0.84 (0.741) | -1.944 (0.249) | 44.676 | < 0.001 |
| Occipital Inf L - Cerebelum 8 R | 0.661 (0.793) | -2.016 (0.238) | 102.228 | < 0.001 |
| Occipital Inf R - Caudate R | 0.145 (0.783) | 2.707 (0.315) | -96.005 | < 0.001 |
| Occipital Inf R - Cerebelum 3 L | 0.217 (0.792) | 1.929 (0.32) | -63.362 | < 0.001 |
| Occipital Inf R - Cerebelum 6 L | 0.581 (0.782) | 1.963 (0.282) | -52.621 | < 0.001 |
| Fusiform L - Fusiform R | 1.021 (0.869) | -2.136 (0.32) | 107.811 | < 0.001 |
| Fusiform L - Thalamus R | 0.672 (0.848) | -2.629 (0.21) | 119.493 | < 0.001 |
| Fusiform L - Temporal Pole Mid R | 0.873 (0.688) | -2.101 (0.277) | 126.834 | < 0.001 |
| Fusiform L - Cerebelum 8 R | 0.019 (0.745) | -2.099 (0.238) | 85.642 | < 0.001 |
| Fusiform R - Postcentral L | -0.982 (0.75) | -2.139 (0.25) | 46.263 | < 0.001 |
| Fusiform R - Cerebelum 8 L | -0.039 (0.822) | -2.084 (0.273) | 74.662 | < 0.001 |
| Postcentral L - Temporal Sup R | -0.745 (0.831) | -1.999 (0.252) | 45.641 | < 0.001 |
| Postcentral L - Cerebelum Crus2 L | 0.72 (0.736) | 2.269 (0.257) | -62.865 | < 0.001 |
| Postcentral R - Pallidum L | -1.103 (0.743) | 2.1 (0.278) | -127.719 | < 0.001 |
| Postcentral R - Temporal Sup R | -0.738 (0.826) | -2.325 (0.262) | 57.950 | < 0.001 |
| Postcentral R - Temporal Pole Sup L | -0.327 (0.791) | -2.3 (0.259) | 74.994 | < 0.001 |
| Parietal Sup L - Pallidum R | 0.708 (0.842) | 1.892 (0.253) | -42.565 | < 0.001 |
| Parietal Inf L - Vermis 3 | -0.312 (0.719) | 2.235 (0.381) | -99.013 | < 0.001 |
| Parietal Inf R - SupraMarginal R | 0.318 (0.769) | 1.967 (0.37) | -61.083 | < 0.001 |
| Parietal Inf R - Precuneus L | -0.093 (0.83) | 2.56 (0.286) | -95.622 | < 0.001 |
| Parietal Inf R - Thalamus R | 0.577 (0.802) | 1.922 (0.323) | -49.159 | < 0.001 |
| Parietal Inf R - Temporal Mid L | -0.116 (0.718) | 2.062 (0.325) | -87.445 | < 0.001 |
| Parietal Inf R - Cerebelum 4 5 L | -0.097 (0.89) | 1.913 (0.292) | -67.864 | < 0.001 |
| SupraMarginal L - Precuneus L | 0.176 (0.783) | 2.085 (0.261) | -73.158 | < 0.001 |
| SupraMarginal L - Cerebelum 4 5 L | -0.36 (0.922) | 2.467 (0.286) | -92.588 | < 0.001 |
| SupraMarginal R - Pallidum L | -0.744 (0.757) | 2.153 (0.37) | -108.755 | < 0.001 |
| Angular L - Pallidum R | -0.436 (0.798) | 2.033 (0.333) | -90.246 | < 0.001 |
| Angular L - Vermis 3 | -0.339 (0.732) | 2.404 (0.34) | -107.431 | < 0.001 |
| Angular R - Temporal Inf L | 0.346 (0.776) | -1.943 (0.259) | 88.460 | < 0.001 |
| Angular R - Temporal Inf R | 0.225 (0.727) | -2.133 (0.254) | 96.792 | < 0.001 |
| Angular R - Cerebelum Crus1 L | -0.61 (0.798) | -2.226 (0.247) | 61.185 | < 0.001 |
| Angular R - Cerebelum Crus1 R | 0.59 (0.826) | -2.239 (0.274) | 102.742 | < 0.001 |
| Angular R - Cerebelum 10 L | 0.605 (0.81) | 2.265 (0.196) | -63.007 | < 0.001 |
| Precuneus L - Pallidum L | -0.488 (0.767) | 2.757 (0.232) | -128.007 | < 0.001 |
| Precuneus L - Temporal Inf R | -0.377 (0.729) | -2.093 (0.236) | 70.800 | < 0.001 |
| Precuneus L - Cerebelum Crus1 R | -0.433 (0.793) | -1.926 (0.286) | 56.030 | < 0.001 |
| Precuneus L - Cerebelum 9 R | 0.374 (0.725) | -2.213 (0.254) | 106.443 | < 0.001 |
| Precuneus R - Pallidum L | 0.062 (0.795) | 3.275 (0.28) | -120.535 | < 0.001 |
| Precuneus R - Pallidum R | -0.439 (0.82) | 2.505 (0.245) | -108.812 | < 0.001 |
| Precuneus R - Cerebelum Crus1 R | 0.747 (0.804) | -2.353 (0.286) | 114.867 | < 0.001 |
| Precuneus R - Cerebelum 8 R | -0.835 (0.813) | -2.26 (0.263) | 52.735 | < 0.001 |
| Paracentral Lobule R - Pallidum L | -0.976 (0.784) | 2.19 (0.269) | -120.834 | < 0.001 |
| Caudate L - Temporal Pole Mid R | -0.209 (0.769) | 2.239 (0.289) | -94.179 | < 0.001 |
| Caudate L - Temporal Inf R | 1.065 (0.763) | 2.047 (0.413) | -35.770 | < 0.001 |
| Caudate L - Cerebelum 7b R | -0.553 (0.764) | 2.099 (0.336) | -100.470 | < 0.001 |
| Caudate L - Cerebelum 8 L | -0.341 (0.854) | 1.922 (0.268) | -79.880 | < 0.001 |
| Caudate L - Vermis 10 | -0.547 (0.803) | 2.361 (0.286) | -107.869 | < 0.001 |
| Caudate R - Putamen L | -0.016 (0.819) | -2.077 (0.282) | 75.255 | < 0.001 |
| Caudate R - Temporal Pole Mid R | -0.571 (0.752) | 2.352 (0.263) | -115.978 | < 0.001 |
| Caudate R - Vermis 10 | 0.055 (0.738) | 2.004 (0.28) | -78.116 | < 0.001 |
| Putamen L - Temporal Pole Sup R | -0.043 (0.68) | -2.097 (0.272) | 88.685 | < 0.001 |
| Putamen L - Cerebelum 10 L | 0.049 (0.766) | 2.18 (0.309) | -81.579 | < 0.001 |
| Pallidum L - Thalamus R | 0.411 (0.622) | 2.475 (0.246) | -97.560 | < 0.001 |
| Pallidum L - Temporal Sup R | 0.09 (0.743) | 2.136 (0.27) | -81.806 | < 0.001 |
| Pallidum L - Temporal Mid R | 0.528 (0.841) | 2.171 (0.263) | -58.973 | < 0.001 |
| Pallidum R - Temporal Sup R | -0.519 (0.845) | 2.143 (0.257) | -95.326 | < 0.001 |
| Thalamus R - Temporal Sup R | 0.796 (0.885) | 2.125 (0.284) | -45.244 | < 0.001 |
| Thalamus R - Cerebelum 6 L | 0.168 (0.786) | -2.189 (0.224) | 91.179 | < 0.001 |
| Thalamus R - Vermis 10 | -0.716 (0.754) | 2.075 (0.257) | -110.772 | < 0.001 |
| Heschl L - Cerebelum 3 L | 0.714 (0.78) | -2.468 (0.191) | 125.326 | < 0.001 |
| Temporal Sup L - Cerebelum Crus1 L | -0.086 (0.858) | 2.025 (0.278) | -74.014 | < 0.001 |
| Temporal Sup L - Cerebelum 6 R | 0.488 (0.768) | 1.936 (0.349) | -54.286 | < 0.001 |
| Temporal Sup L - Vermis 3 | 0.602 (0.784) | 1.907 (0.255) | -50.056 | < 0.001 |
| Temporal Sup R - Vermis 3 | -0.081 (0.83) | 2.163 (0.293) | -80.618 | < 0.001 |
| Temporal Pole Sup L - Cerebelum 8 L | 0.149 (0.745) | -2.154 (0.234) | 93.204 | < 0.001 |
| Temporal Pole Sup R - Cerebelum 7b R | 0.739 (0.89) | 1.98 (0.39) | -40.417 | < 0.001 |
| Temporal Pole Sup R - Vermis 3 | 0.419 (0.733) | 2.272 (0.309) | -73.693 | < 0.001 |
| Temporal Mid L - Vermis 3 | 0.565 (0.768) | 2.268 (0.304) | -65.193 | < 0.001 |
| Temporal Mid R - Vermis 3 | 1.1 (0.782) | 2.248 (0.31) | -43.135 | < 0.001 |
| Temporal Inf L - Cerebelum 7b R | 0.618 (0.805) | -2.041 (0.232) | 100.308 | < 0.001 |
| Temporal Inf L - Cerebelum 8 R | 0.082 (0.723) | -1.96 (0.236) | 84.931 | < 0.001 |
| Temporal Inf R - Cerebelum 8 L | -0.174 (0.757) | -2.342 (0.254) | 85.888 | < 0.001 |
| Temporal Inf R - Vermis 9 | -0.14 (0.742) | 1.907 (0.255) | -82.527 | < 0.001 |
| Cerebelum Crus1 L - Cerebelum 7b R | 0.249 (0.857) | -2.294 (0.273) | 89.344 | < 0.001 |
| Cerebelum Crus1 L - Cerebelum 10 L | 0.465 (0.695) | 1.918 (0.297) | -60.780 | < 0.001 |
| Cerebelum Crus1 R - Cerebelum Crus2 L | -0.137 (0.76) | -2.181 (0.287) | 79.588 | < 0.001 |
| Cerebelum 3 R - Vermis 3 | -0.004 (0.786) | 2.202 (0.279) | -83.630 | < 0.001 |
| Cerebelum 3 R - Vermis 4 5 | -0.504 (0.67) | 2.246 (0.296) | -118.720 | < 0.001 |
| Cerebelum 4 5 L - Cerebelum 9 R | -0.782 (0.787) | -2.153 (0.216) | 53.154 | < 0.001 |
| Cerebelum 6 L - Cerebelum 6 R | 0.612 (0.774) | -2.067 (0.283) | 102.782 | < 0.001 |
| Cerebelum 6 L - Cerebelum 7b R | 0.114 (0.857) | -2.221 (0.235) | 83.083 | < 0.001 |
| Cerebelum 6 L - Cerebelum 8 R | -0.051 (0.83) | -2.387 (0.219) | 86.021 | < 0.001 |
| Cerebelum 6 R - Cerebelum 8 R | -0.208 (0.776) | -2 (0.266) | 69.034 | < 0.001 |
| Cerebelum 7b R - Vermis 7 | 0.033 (0.83) | -2.09 (0.25) | 77.478 | < 0.001 |
| Cerebelum 8 R - Vermis 6 | -0.357 (0.8) | -2.219 (0.231) | 70.706 | < 0.001 |
| Cerebelum 8 R - Vermis 7 | 0.372 (0.816) | -2.063 (0.251) | 90.178 | < 0.001 |
| Cerebelum 9 L - Vermis 3 | -0.581 (0.751) | -1.959 (0.228) | 55.530 | < 0.001 |
| Cerebelum 9 R - Vermis 3 | -0.6 (0.808) | -2.118 (0.216) | 57.369 | < 0.001 |
| Vermis 1 2 - Vermis 3 | 1.011 (0.688) | 2.274 (0.277) | -53.840 | < 0.001 |

**Supplementary Table 10**. Between-group comparison on regional frailty-related ROI-to-ROI connectivity effects between CU and FTLD. Mean (SD) t-values of the frailty–GMV association estimated separately in cognitively unimpaired (CU) and frontotemporal lobar degeneration (FTLD) groups using repeated subsampling (n = 1000) and models adjusted for scanner effects.

| **Regions** | **AD** | **FTLD** | ***t*** | ***P*_FDR_** |
| --- | --- | --- | --- | --- |
| Precentral L - Lingual L | -1.215 (0.875) | -2.684 (0.218) | 51.496 | < 0.001 |
| Precentral L - Lingual R | -1.032 (0.844) | -2.85 (0.241) | 65.503 | < 0.001 |
| Precentral L - Fusiform L | -1.499 (0.858) | -2.467 (0.244) | 34.288 | < 0.001 |
| Precentral R - Occipital Inf R | -1.139 (0.788) | -1.418 (0.261) | 10.646 | < 0.001 |
| Frontal Sup Orb L - Cerebelum 3 L | 1.122 (0.898) | -0.003 (0.41) | 36.050 | < 0.001 |
| Frontal Sup Orb R - Frontal Inf Oper R | 1.198 (0.835) | 0.848 (0.259) | 12.644 | < 0.001 |
| Frontal Mid Orb R - Cerebelum 3 L | 1.358 (0.925) | 1.171 (0.418) | 5.820 | < 0.001 |
| Frontal Inf Oper L - Temporal Inf L | 0.445 (0.833) | -2.855 (0.254) | 119.870 | < 0.001 |
| Frontal Inf Tri L - Temporal Inf L | 0.352 (0.884) | -2.289 (0.24) | 91.218 | < 0.001 |
| Frontal Med Orb R - Amygdala L | 1.446 (0.929) | 0.416 (0.389) | 32.343 | < 0.001 |
| Rectus L - Amygdala L | 1.432 (0.968) | -0.895 (0.268) | 73.282 | < 0.001 |
| Rectus L - Cerebelum 3 L | 1.622 (0.866) | -0.74 (0.475) | 75.623 | < 0.001 |
| Rectus L - Cerebelum 4 5 L | 1.892 (0.858) | -0.18 (0.323) | 71.448 | < 0.001 |
| Rectus R - Amygdala L | 1.309 (0.882) | -0.344 (0.335) | 55.422 | < 0.001 |
| Rectus R - Occipital Inf L | 1.265 (0.877) | 0.71 (0.299) | 18.932 | < 0.001 |
| Rectus R - Cerebelum 4 5 L | 1.402 (0.8) | 0.821 (0.352) | 20.999 | < 0.001 |
| Rectus R - Cerebelum 4 5 R | 1.433 (0.829) | 0.61 (0.333) | 29.125 | < 0.001 |
| Rectus R - Cerebelum 6 L | 1.658 (0.895) | 1.565 (0.296) | 3.127 | 0.003 |
| Cingulum Mid R - Fusiform L | -1.111 (0.789) | -2.25 (0.213) | 44.073 | < 0.001 |
| Cingulum Post L - Hippocampus L | -1.339 (0.951) | 0.94 (0.282) | -72.660 | < 0.001 |
| Hippocampus R - Vermis 4 5 | 1.158 (0.887) | 1.087 (0.291) | 2.398 | 0.026 |
| ParaHippocampal R - Cerebelum 4 5 L | 1.327 (0.906) | 1.848 (0.328) | -17.102 | < 0.001 |
| ParaHippocampal R - Cerebelum 4 5 R | 1.371 (0.905) | 1.237 (0.276) | 4.478 | < 0.001 |
| Calcarine L - Vermis 4 5 | 1.286 (0.866) | 0.065 (0.265) | 42.627 | < 0.001 |
| Calcarine R - Vermis 4 5 | 1.405 (0.891) | 0.793 (0.236) | 20.966 | < 0.001 |
| Cuneus L - Fusiform R | -0.229 (0.842) | -2.175 (0.269) | 69.643 | < 0.001 |
| Cuneus L - Temporal Inf R | -1.019 (0.907) | -2.171 (0.221) | 39.003 | < 0.001 |
| Cuneus R - Fusiform L | -0.681 (0.828) | -1.991 (0.265) | 47.679 | < 0.001 |
| Cuneus R - Pallidum R | -0.133 (0.813) | 2.682 (0.291) | -103.102 | < 0.001 |
| Lingual L - Paracentral Lobule L | -0.491 (0.829) | -2.164 (0.224) | 61.613 | < 0.001 |
| Lingual L - Cerebelum 4 5 R | 1.102 (0.925) | 0.043 (0.261) | 34.861 | < 0.001 |
| Lingual L - Vermis 4 5 | 1.213 (0.96) | 0.63 (0.262) | 18.545 | < 0.001 |
| Lingual R - Paracentral Lobule L | -0.503 (0.934) | -3.159 (0.241) | 87.055 | < 0.001 |
| Lingual R - Paracentral Lobule R | -0.62 (0.812) | -1.926 (0.216) | 49.117 | < 0.001 |
| Lingual R - Cerebelum 4 5 R | 1.141 (0.897) | 0.695 (0.274) | 15.046 | < 0.001 |
| Occipital Sup L - Fusiform R | -0.802 (0.852) | -2.197 (0.252) | 49.650 | < 0.001 |
| Occipital Sup L - Temporal Inf R | -1.233 (0.906) | -1.895 (0.252) | 22.281 | < 0.001 |
| Occipital Mid L - Temporal Inf R | -1.164 (0.825) | -2.046 (0.246) | 32.426 | < 0.001 |
| Occipital Mid L - Vermis 4 5 | 2.015 (0.966) | -0.029 (0.282) | 64.243 | < 0.001 |
| Occipital Mid R - Temporal Inf R | -0.859 (0.855) | -2.232 (0.272) | 48.406 | < 0.001 |
| Occipital Inf L - Cerebelum 4 5 L | 1.589 (0.972) | 1.695 (0.328) | -3.274 | 0.002 |
| Occipital Inf L - Cerebelum 4 5 R | 1.589 (0.926) | 1.196 (0.29) | 12.793 | < 0.001 |
| Occipital Inf L - Vermis 4 5 | 1.141 (0.857) | -0.216 (0.261) | 47.875 | < 0.001 |
| Occipital Inf R - Postcentral L | -1.113 (0.786) | -2.153 (0.263) | 39.689 | < 0.001 |
| Fusiform R - Cerebelum 4 5 R | 1.643 (0.844) | 0.764 (0.257) | 31.506 | < 0.001 |
| Fusiform R - Vermis 6 | 1.29 (0.861) | 0.666 (0.308) | 21.560 | < 0.001 |
| Postcentral L - Cerebelum Crus2 R | -0.191 (0.768) | 2.241 (0.278) | -94.224 | < 0.001 |
| Parietal Sup R - Caudate L | 1.393 (0.863) | 0.021 (0.338) | 46.831 | < 0.001 |
| Angular L - Temporal Pole Sup R | -1.485 (0.821) | 0.577 (0.24) | -76.242 | < 0.001 |
| Precuneus L - Cerebelum 7b R | -0.039 (0.809) | -1.923 (0.276) | 69.712 | < 0.001 |
| Putamen L - Cerebelum 4 5 L | 1.567 (0.867) | -1.018 (0.227) | 91.173 | < 0.001 |
| Pallidum L - Cerebelum 3 L | 1.493 (0.955) | -1.3 (0.248) | 89.545 | < 0.001 |
| Thalamus R - Heschl R | -0.538 (0.874) | 2.112 (0.275) | -91.473 | < 0.001 |
| Temporal Sup R - Vermis 4 5 | 0.494 (0.919) | 1.897 (0.315) | -45.660 | < 0.001 |
| Temporal Mid L - Cerebelum 4 5 L | 1.24 (0.901) | 1.489 (0.317) | -8.260 | < 0.001 |
| Temporal Mid L - Vermis 4 5 | 1.056 (0.802) | 2.331 (0.319) | -46.664 | < 0.001 |
| Temporal Mid R - Cerebelum Crus1 R | 1.224 (0.871) | -1.183 (0.267) | 83.564 | < 0.001 |
| Temporal Mid R - Cerebelum 4 5 R | 1.382 (0.771) | 1.193 (0.299) | 7.236 | < 0.001 |
| Temporal Mid R - Vermis 4 5 | 1.365 (0.844) | 2.643 (0.275) | -45.532 | < 0.001 |
| Cerebelum 4 5 R - Vermis 1 2 | -0.175 (1.017) | 3.909 (0.302) | -121.802 | < 0.001 |
| Precentral L - Frontal Sup Medial R | 1.193 (0.87) | 0.614 (0.287) | 19.994 | < 0.001 |
| Precentral L - Cuneus L | -1.253 (0.866) | -1.85 (0.246) | 20.957 | < 0.001 |
| Precentral L - Occipital Sup L | -1.21 (0.879) | -2.073 (0.264) | 29.735 | < 0.001 |
| Precentral L - Occipital Mid L | -1.35 (0.831) | -2.986 (0.303) | 58.529 | < 0.001 |
| Precentral L - Fusiform R | -1.274 (0.781) | -1.956 (0.226) | 26.521 | < 0.001 |
| Precentral L - Cerebelum 8 L | -1.37 (0.82) | -0.74 (0.26) | -23.141 | < 0.001 |
| Precentral L - Vermis 8 | -1.264 (0.826) | 2.401 (0.241) | -134.678 | < 0.001 |
| Precentral R - Rolandic Oper R | -1.84 (0.844) | -1.318 (0.252) | -18.736 | < 0.001 |
| Precentral R - Calcarine R | -1.307 (0.798) | -2.136 (0.198) | 31.855 | < 0.001 |
| Precentral R - Cuneus L | -1.238 (0.813) | -2.255 (0.219) | 38.192 | < 0.001 |
| Precentral R - Cuneus R | -1.258 (0.802) | -2.421 (0.241) | 43.905 | < 0.001 |
| Precentral R - Occipital Sup L | -1.332 (0.846) | -2.24 (0.225) | 32.823 | < 0.001 |
| Precentral R - Occipital Sup R | -1.586 (0.836) | -2.425 (0.232) | 30.597 | < 0.001 |
| Precentral R - Occipital Mid L | -1.352 (0.826) | -2.561 (0.212) | 44.835 | < 0.001 |
| Precentral R - Occipital Mid R | -1.427 (0.785) | -2.45 (0.252) | 39.234 | < 0.001 |
| Precentral R - Fusiform L | -1.188 (0.783) | -2.458 (0.223) | 49.335 | < 0.001 |
| Precentral R - SupraMarginal L | -1.144 (0.809) | -0.501 (0.231) | -24.163 | < 0.001 |
| Precentral R - Temporal Sup R | -1.146 (0.821) | -2.035 (0.267) | 32.554 | < 0.001 |
| Precentral R - Vermis 10 | -1.257 (0.786) | 0.758 (0.287) | -76.133 | < 0.001 |
| Frontal Sup L - Frontal Mid L | 1.159 (0.82) | -0.181 (0.257) | 49.296 | < 0.001 |
| Frontal Sup L - Insula R | -1.079 (0.821) | 0.33 (0.308) | -50.838 | < 0.001 |
| Frontal Sup R - Frontal Mid R | 1.204 (0.779) | -0.602 (0.272) | 69.180 | < 0.001 |
| Frontal Sup R - Vermis 10 | -1.265 (0.829) | 0.392 (0.32) | -58.937 | < 0.001 |
| Frontal Sup Orb L - Supp Motor Area L | 1.246 (0.85) | -0.567 (0.25) | 64.663 | < 0.001 |
| Frontal Sup Orb L - Supp Motor Area R | 1.098 (0.833) | -0.266 (0.275) | 49.165 | < 0.001 |
| Frontal Sup Orb L - ParaHippocampal L | 1.249 (0.791) | 0.334 (0.266) | 34.689 | < 0.001 |
| Frontal Sup Orb L - Cerebelum 4 5 L | 1.171 (0.949) | 0.108 (0.344) | 33.294 | < 0.001 |
| Frontal Sup Orb L - Vermis 6 | 1.139 (0.884) | 0.096 (0.261) | 35.761 | < 0.001 |
| Frontal Sup Orb L - Vermis 9 | 1.463 (0.811) | 0.758 (0.228) | 26.462 | < 0.001 |
| Frontal Sup Orb R - Frontal Inf Oper L | 1.254 (0.769) | 0.539 (0.261) | 27.845 | < 0.001 |
| Frontal Sup Orb R - Supp Motor Area L | 1.284 (0.847) | 0.326 (0.26) | 34.186 | < 0.001 |
| Frontal Sup Orb R - Hippocampus L | 1.304 (0.86) | 1.202 (0.323) | 3.517 | 0.001 |
| Frontal Sup Orb R - Amygdala L | 1.273 (1.063) | 0.134 (0.264) | 32.884 | < 0.001 |
| Frontal Sup Orb R - Caudate R | 1.303 (0.85) | 0.348 (0.265) | 33.935 | < 0.001 |
| Frontal Sup Orb R - Thalamus R | 1.17 (0.761) | 0.182 (0.31) | 37.993 | < 0.001 |
| Frontal Sup Orb R - Heschl L | 1.514 (0.908) | 0.385 (0.273) | 37.610 | < 0.001 |
| Frontal Sup Orb R - Cerebelum 4 5 L | 1.234 (0.984) | 1.789 (0.319) | -16.948 | < 0.001 |
| Frontal Sup Orb R - Cerebelum 4 5 R | 1.199 (0.942) | 0.151 (0.317) | 33.348 | < 0.001 |
| Frontal Mid L - Frontal Sup Medial L | 1.35 (0.853) | 1.147 (0.253) | 7.222 | < 0.001 |
| Frontal Mid L - Frontal Sup Medial R | 1.089 (0.815) | 0.755 (0.268) | 12.325 | < 0.001 |
| Frontal Mid L - Cerebelum Crus1 L | -1.307 (0.9) | -0.931 (0.289) | -12.593 | < 0.001 |
| Frontal Mid L - Cerebelum Crus2 L | -1.338 (0.94) | -1.005 (0.284) | -10.729 | < 0.001 |
| Frontal Mid L - Cerebelum 8 L | -1.335 (0.777) | 0.124 (0.282) | -55.826 | < 0.001 |
| Frontal Mid L - Cerebelum 10 R | -1.102 (0.783) | 0.319 (0.288) | -53.809 | < 0.001 |
| Frontal Mid L - Vermis 7 | -1.299 (0.815) | -0.028 (0.274) | -46.751 | < 0.001 |
| Frontal Mid L - Vermis 8 | -1.722 (0.83) | 1.359 (0.256) | -112.142 | < 0.001 |
| Frontal Mid L - Vermis 9 | -1.351 (0.796) | 1.163 (0.25) | -95.334 | < 0.001 |
| Frontal Mid R - Supp Motor Area R | 1.385 (0.895) | -0.604 (0.289) | 66.878 | < 0.001 |
| Frontal Mid R - Frontal Sup Medial R | 1.499 (0.873) | 0.945 (0.278) | 19.136 | < 0.001 |
| Frontal Mid R - Cingulum Ant L | 1.096 (0.901) | 0.817 (0.249) | 9.438 | < 0.001 |
| Frontal Mid R - Cingulum Ant R | 1.449 (0.88) | 0.234 (0.275) | 41.687 | < 0.001 |
| Frontal Mid Orb L - Olfactory L | 1.062 (0.945) | -0.685 (0.26) | 56.338 | < 0.001 |
| Frontal Mid Orb L - Frontal Sup Medial R | 1.458 (0.901) | 2.04 (0.232) | -19.791 | < 0.001 |
| Frontal Mid Orb L - ParaHippocampal L | 1.557 (0.779) | 0.135 (0.248) | 55.010 | < 0.001 |
| Frontal Mid Orb L - ParaHippocampal R | 1.444 (0.822) | 1.089 (0.251) | 13.079 | < 0.001 |
| Frontal Mid Orb L - Lingual L | 1.276 (0.91) | 0.351 (0.248) | 31.000 | < 0.001 |
| Frontal Mid Orb L - Cerebelum 4 5 L | 1.391 (0.895) | 0.541 (0.333) | 28.146 | < 0.001 |
| Frontal Mid Orb L - Vermis 1 2 | 1.542 (0.915) | 0.378 (0.35) | 37.583 | < 0.001 |
| Frontal Mid Orb R - Supp Motor Area R | 1.211 (0.863) | 0.307 (0.268) | 31.651 | < 0.001 |
| Frontal Mid Orb R - Frontal Sup Medial R | 1.992 (0.894) | 1.275 (0.291) | 24.138 | < 0.001 |
| Frontal Mid Orb R - ParaHippocampal L | 1.733 (0.861) | -0.061 (0.326) | 61.634 | < 0.001 |
| Frontal Mid Orb R - ParaHippocampal R | 1.202 (0.849) | 1.282 (0.287) | -2.827 | 0.007 |
| Frontal Mid Orb R - Amygdala L | 1.259 (0.984) | -0.074 (0.277) | 41.266 | < 0.001 |
| Frontal Mid Orb R - Heschl L | 1.274 (0.85) | -0.415 (0.288) | 59.516 | < 0.001 |
| Frontal Mid Orb R - Cerebelum 4 5 L | 1.496 (0.987) | 1.685 (0.36) | -5.673 | < 0.001 |
| Frontal Mid Orb R - Cerebelum 6 L | 1.479 (0.882) | 0.168 (0.235) | 45.424 | < 0.001 |
| Frontal Inf Oper L - Postcentral L | 1.263 (0.919) | -1.169 (0.24) | 80.979 | < 0.001 |
| Frontal Inf Oper L - Cerebelum 4 5 R | -1.181 (0.827) | -1.005 (0.238) | -6.460 | < 0.001 |
| Frontal Inf Oper L - Vermis 7 | -1.194 (0.814) | 0.351 (0.219) | -57.958 | < 0.001 |
| Frontal Inf Oper R - Frontal Sup Medial R | 1.166 (0.849) | -0.299 (0.238) | 52.579 | < 0.001 |
| Frontal Inf Oper R - Pallidum L | -1.162 (0.845) | 0.679 (0.321) | -64.391 | < 0.001 |
| Frontal Inf Tri L - Cingulum Post R | 1.3 (0.847) | -0.406 (0.226) | 61.532 | < 0.001 |
| Frontal Inf Tri L - Cerebelum 4 5 R | -1.421 (0.847) | -0.553 (0.25) | -31.058 | < 0.001 |
| Frontal Inf Tri L - Vermis 8 | -1.108 (0.783) | 0.277 (0.331) | -51.511 | < 0.001 |
| Frontal Inf Tri R - Fusiform R | -1.174 (0.919) | 0.299 (0.291) | -48.335 | < 0.001 |
| Frontal Inf Tri R - Pallidum L | -1.187 (0.941) | 0.147 (0.286) | -42.863 | < 0.001 |
| Frontal Inf Tri R - Temporal Pole Mid L | 1.35 (0.967) | -0.541 (0.278) | 59.457 | < 0.001 |
| Frontal Inf Tri R - Cerebelum 4 5 R | -1.212 (0.797) | -0.611 (0.25) | -22.748 | < 0.001 |
| Frontal Inf Tri R - Cerebelum 10 L | -1.567 (0.909) | -1.058 (0.192) | -17.329 | < 0.001 |
| Frontal Inf Tri R - Cerebelum 10 R | -1.25 (0.921) | 0.022 (0.225) | -42.396 | < 0.001 |
| Frontal Inf Tri R - Vermis 3 | -1.245 (0.829) | -0.521 (0.194) | -26.883 | < 0.001 |
| Frontal Inf Tri R - Vermis 10 | -1.14 (0.781) | -0.951 (0.208) | -7.385 | < 0.001 |
| Frontal Inf Orb L - ParaHippocampal L | 1.143 (0.883) | -1.056 (0.304) | 74.426 | < 0.001 |
| Frontal Inf Orb L - Vermis 3 | 1.471 (0.923) | 1.251 (0.368) | 7.008 | < 0.001 |
| Frontal Inf Orb R - Supp Motor Area R | 1.286 (0.839) | -0.242 (0.283) | 54.528 | < 0.001 |
| Frontal Inf Orb R - Hippocampus R | 1.223 (0.843) | 1.452 (0.245) | -8.241 | < 0.001 |
| Frontal Inf Orb R - ParaHippocampal L | 1.384 (0.878) | 0.706 (0.31) | 23.033 | < 0.001 |
| Frontal Inf Orb R - Cerebelum 6 L | 1.425 (0.953) | -0.103 (0.239) | 49.186 | < 0.001 |
| Frontal Inf Orb R - Cerebelum 6 R | 1.169 (0.984) | 0.945 (0.266) | 6.921 | < 0.001 |
| Frontal Inf Orb R - Vermis 6 | 1.274 (0.99) | 0.61 (0.278) | 20.420 | < 0.001 |
| Rolandic Oper L - Rolandic Oper R | -1.204 (0.829) | -0.041 (0.256) | -42.425 | < 0.001 |
| Rolandic Oper L - Cingulum Mid L | -1.643 (0.779) | -0.928 (0.248) | -27.681 | < 0.001 |
| Rolandic Oper L - Cingulum Mid R | -1.17 (0.783) | -0.548 (0.226) | -24.115 | < 0.001 |
| Rolandic Oper L - Lingual R | -1.138 (0.8) | -0.588 (0.242) | -20.811 | < 0.001 |
| Rolandic Oper L - Fusiform L | -1.089 (0.742) | -1.438 (0.196) | 14.407 | < 0.001 |
| Rolandic Oper L - Precuneus L | -1.148 (0.748) | 1.374 (0.271) | -100.259 | < 0.001 |
| Rolandic Oper L - Precuneus R | -1.271 (0.776) | 1.5 (0.303) | -105.183 | < 0.001 |
| Rolandic Oper L - Vermis 1 2 | 1.388 (0.837) | 0.294 (0.322) | 38.580 | < 0.001 |
| Rolandic Oper R - Insula L | -1.374 (0.788) | 0.081 (0.295) | -54.695 | < 0.001 |
| Rolandic Oper R - Insula R | -1.279 (0.803) | 0.38 (0.327) | -60.523 | < 0.001 |
| Rolandic Oper R - Cingulum Mid L | -1.149 (0.809) | -2.793 (0.247) | 61.441 | < 0.001 |
| Rolandic Oper R - Lingual R | -1.152 (0.807) | -0.408 (0.243) | -27.927 | < 0.001 |
| Rolandic Oper R - Postcentral L | -1.121 (0.812) | -1.918 (0.249) | 29.659 | < 0.001 |
| Rolandic Oper R - SupraMarginal R | -1.263 (0.781) | -1.524 (0.279) | 9.953 | < 0.001 |
| Rolandic Oper R - Paracentral Lobule R | -1.44 (0.856) | -0.669 (0.246) | -27.412 | < 0.001 |
| Rolandic Oper R - Temporal Sup L | -1.37 (0.827) | -1.061 (0.25) | -11.306 | < 0.001 |
| Rolandic Oper R - Temporal Sup R | -1.473 (0.897) | -0.706 (0.242) | -26.118 | < 0.001 |
| Rolandic Oper R - Cerebelum 4 5 R | -1.246 (0.793) | 0.379 (0.315) | -60.224 | < 0.001 |
| Rolandic Oper R - Cerebelum 8 L | -1.345 (0.726) | -0.169 (0.218) | -49.051 | < 0.001 |
| Rolandic Oper R - Cerebelum 10 R | -1.06 (0.767) | 0.445 (0.294) | -57.971 | < 0.001 |
| Supp Motor Area L - Calcarine R | -1.2 (0.885) | -1.775 (0.243) | 19.807 | < 0.001 |
| Supp Motor Area L - Cuneus L | -1.143 (0.919) | -1.543 (0.247) | 13.277 | < 0.001 |
| Supp Motor Area L - Lingual R | -1.21 (0.92) | -1.359 (0.244) | 4.970 | < 0.001 |
| Supp Motor Area L - Fusiform L | -1.25 (0.858) | -0.453 (0.265) | -28.055 | < 0.001 |
| Supp Motor Area R - Frontal Sup Medial L | 1.182 (0.828) | 0.24 (0.253) | 34.407 | < 0.001 |
| Supp Motor Area R - Rectus L | 1.119 (0.787) | 0.394 (0.308) | 27.115 | < 0.001 |
| Supp Motor Area R - Rectus R | 1.338 (0.801) | 1.155 (0.279) | 6.824 | < 0.001 |
| Supp Motor Area R - Insula L | -1.324 (0.761) | -1.052 (0.291) | -10.559 | < 0.001 |
| Supp Motor Area R - SupraMarginal L | -1.108 (0.772) | -0.807 (0.228) | -11.838 | < 0.001 |
| Supp Motor Area R - Paracentral Lobule L | 1.143 (0.871) | -1.144 (0.28) | 78.997 | < 0.001 |
| Olfactory L - Cerebelum 10 L | 1.198 (0.829) | -0.853 (0.314) | 73.114 | < 0.001 |
| Olfactory R - Heschl R | 1.373 (0.815) | -0.942 (0.268) | 85.325 | < 0.001 |
| Frontal Sup Medial L - Caudate L | -1.08 (0.854) | 0.958 (0.281) | -71.712 | < 0.001 |
| Frontal Med Orb L - Vermis 1 2 | 1.455 (0.842) | 2.286 (0.316) | -29.225 | < 0.001 |
| Frontal Med Orb R - ParaHippocampal L | 1.127 (0.922) | -0.296 (0.331) | 45.920 | < 0.001 |
| Frontal Med Orb R - Parietal Sup L | 1.464 (0.898) | -0.044 (0.29) | 50.516 | < 0.001 |
| Frontal Med Orb R - Parietal Inf L | 1.206 (0.803) | 0.29 (0.292) | 33.903 | < 0.001 |
| Frontal Med Orb R - Caudate L | -1.216 (0.851) | 0.601 (0.251) | -64.795 | < 0.001 |
| Frontal Med Orb R - Cerebelum 6 L | 1.286 (0.839) | -0.363 (0.195) | 60.553 | < 0.001 |
| Frontal Med Orb R - Cerebelum 8 R | 1.497 (0.785) | -0.61 (0.259) | 80.571 | < 0.001 |
| Frontal Med Orb R - Vermis 6 | 1.542 (0.795) | 0.073 (0.261) | 55.523 | < 0.001 |
| Rectus L - ParaHippocampal L | 2.059 (0.946) | -0.296 (0.351) | 73.837 | < 0.001 |
| Rectus L - ParaHippocampal R | 1.268 (0.854) | -1.264 (0.358) | 86.495 | < 0.001 |
| Rectus L - Occipital Mid L | 1.326 (0.756) | 0.968 (0.263) | 14.155 | < 0.001 |
| Rectus L - Occipital Inf L | 1.478 (0.893) | 0.368 (0.309) | 37.168 | < 0.001 |
| Rectus L - Occipital Inf R | 1.255 (0.958) | -0.764 (0.277) | 64.026 | < 0.001 |
| Rectus L - Heschl L | 1.172 (0.852) | 0.45 (0.268) | 25.533 | < 0.001 |
| Rectus L - Temporal Inf R | 1.307 (0.907) | -1.174 (0.28) | 82.673 | < 0.001 |
| Rectus L - Cerebelum 6 R | 1.253 (0.769) | 0.884 (0.253) | 14.400 | < 0.001 |
| Rectus L - Cerebelum 8 L | 1.095 (1.02) | -0.084 (0.261) | 35.383 | < 0.001 |
| Rectus L - Cerebelum 8 R | 1.167 (0.896) | 0.003 (0.269) | 39.368 | < 0.001 |
| Rectus L - Cerebelum 10 L | 1.349 (0.839) | -0.956 (0.269) | 82.706 | < 0.001 |
| Rectus L - Vermis 6 | 1.352 (0.824) | 0.358 (0.262) | 36.362 | < 0.001 |
| Rectus L - Vermis 9 | 1.133 (0.715) | 0.911 (0.268) | 9.177 | < 0.001 |
| Rectus R - ParaHippocampal L | 1.439 (0.984) | 0.357 (0.373) | 32.500 | < 0.001 |
| Rectus R - ParaHippocampal R | 1.822 (0.863) | 0.751 (0.373) | 36.021 | < 0.001 |
| Rectus R - Occipital Inf R | 1.381 (0.904) | 0.083 (0.28) | 43.382 | < 0.001 |
| Rectus R - Fusiform R | 1.196 (0.835) | 1.133 (0.285) | 2.250 | 0.038 |
| Rectus R - Heschl L | 1.386 (0.868) | -0.13 (0.258) | 52.923 | < 0.001 |
| Rectus R - Temporal Pole Sup L | 1.123 (0.877) | -0.033 (0.31) | 39.277 | < 0.001 |
| Rectus R - Temporal Inf L | 1.422 (0.887) | -0.372 (0.256) | 61.475 | < 0.001 |
| Rectus R - Temporal Inf R | 1.335 (0.802) | -0.295 (0.246) | 61.462 | < 0.001 |
| Rectus R - Cerebelum Crus1 L | 1.146 (0.866) | 0.182 (0.265) | 33.676 | < 0.001 |
| Rectus R - Cerebelum 3 L | 1.318 (0.91) | 0.224 (0.437) | 34.285 | < 0.001 |
| Rectus R - Vermis 8 | 1.496 (0.799) | -0.85 (0.245) | 88.783 | < 0.001 |
| Insula L - Cingulum Ant L | -1.195 (0.759) | -1.286 (0.3) | 3.538 | 0.001 |
| Insula L - Cingulum Mid L | -1.217 (0.807) | 0.458 (0.411) | -58.536 | < 0.001 |
| Insula L - Cingulum Mid R | -1.343 (0.807) | 0.032 (0.302) | -50.442 | < 0.001 |
| Insula L - Cerebelum Crus1 R | -1.256 (0.789) | 0.546 (0.251) | -68.794 | < 0.001 |
| Insula R - Cingulum Ant R | -1.412 (0.752) | 0.075 (0.27) | -58.849 | < 0.001 |
| Insula R - Cingulum Mid L | -1.409 (0.793) | 0.368 (0.317) | -65.819 | < 0.001 |
| Insula R - Calcarine L | -1.126 (0.827) | 0.753 (0.278) | -68.091 | < 0.001 |
| Insula R - Occipital Inf L | -1.118 (0.8) | 0.235 (0.291) | -50.235 | < 0.001 |
| Insula R - Fusiform L | -1.472 (0.777) | 0.909 (0.259) | -91.975 | < 0.001 |
| Insula R - Fusiform R | -1.26 (0.754) | 2.65 (0.421) | -143.209 | < 0.001 |
| Insula R - Pallidum L | -1.249 (0.882) | 0.73 (0.289) | -67.425 | < 0.001 |
| Cingulum Ant R - Hippocampus L | 1.13 (0.9) | 1.736 (0.26) | -20.467 | < 0.001 |
| Cingulum Ant R - Cerebelum 8 L | 1.251 (0.932) | -0.371 (0.225) | 53.528 | < 0.001 |
| Cingulum Mid L - Cingulum Mid R | -1.119 (0.873) | -0.47 (0.271) | -22.448 | < 0.001 |
| Cingulum Mid L - Caudate L | -1.218 (0.785) | 0.611 (0.28) | -69.442 | < 0.001 |
| Cingulum Mid L - Thalamus L | -1.233 (0.796) | 0.777 (0.235) | -76.589 | < 0.001 |
| Cingulum Mid L - Heschl L | -1.429 (0.732) | -0.749 (0.268) | -27.552 | < 0.001 |
| Cingulum Mid L - Heschl R | -1.919 (0.811) | -1.557 (0.274) | -13.368 | < 0.001 |
| Cingulum Mid L - Vermis 8 | -1.731 (0.818) | 0.198 (0.265) | -70.947 | < 0.001 |
| Cingulum Mid R - Heschl R | -1.596 (0.824) | -1.786 (0.238) | 7.000 | < 0.001 |
| Cingulum Mid R - Temporal Sup R | -1.24 (0.854) | -2.771 (0.231) | 54.675 | < 0.001 |
| Cingulum Mid R - Cerebelum 8 L | -1.672 (0.809) | -1.591 (0.216) | -3.042 | 0.004 |
| Cingulum Mid R - Vermis 8 | -1.514 (0.795) | 0.512 (0.248) | -76.920 | < 0.001 |
| Cingulum Post L - Cuneus R | 1.235 (0.795) | 0.009 (0.276) | 46.070 | < 0.001 |
| Cingulum Post L - SupraMarginal R | 1.304 (0.785) | 0.05 (0.283) | 47.554 | < 0.001 |
| Cingulum Post L - Cerebelum 10 R | -1.098 (0.87) | -0.287 (0.238) | -28.417 | < 0.001 |
| Cingulum Post R - Putamen R | 1.282 (0.836) | 0.15 (0.23) | 41.265 | < 0.001 |
| Cingulum Post R - Cerebelum Crus2 R | -1.334 (0.827) | -0.947 (0.25) | -14.154 | < 0.001 |
| Hippocampus L - Precuneus L | -1.273 (0.77) | -0.935 (0.271) | -13.116 | < 0.001 |
| Hippocampus R - Precuneus L | -1.264 (0.755) | -0.785 (0.254) | -18.988 | < 0.001 |
| Hippocampus R - Cerebelum 4 5 R | 1.481 (0.849) | 0.243 (0.258) | 44.152 | < 0.001 |
| Hippocampus R - Vermis 1 2 | 1.156 (0.87) | -0.3 (0.288) | 50.264 | < 0.001 |
| ParaHippocampal L - Pallidum L | 1.127 (0.877) | -0.22 (0.232) | 46.919 | < 0.001 |
| ParaHippocampal L - Temporal Sup L | 1.192 (0.952) | -0.262 (0.267) | 46.502 | < 0.001 |
| ParaHippocampal R - Cerebelum 10 L | 1.709 (0.935) | -0.297 (0.269) | 65.227 | < 0.001 |
| ParaHippocampal R - Vermis 4 5 | 1.241 (0.832) | -0.079 (0.269) | 47.695 | < 0.001 |
| ParaHippocampal R - Vermis 6 | 1.32 (0.902) | 1.693 (0.284) | -12.502 | < 0.001 |
| Amygdala L - Angular L | 1.405 (0.942) | 1.965 (0.241) | -18.202 | < 0.001 |
| Amygdala L - Cerebelum 9 R | 1.198 (0.872) | -0.387 (0.227) | 55.612 | < 0.001 |
| Amygdala L - Vermis 1 2 | 1.13 (0.899) | 1.384 (0.244) | -8.621 | < 0.001 |
| Amygdala R - Cuneus L | -1.145 (0.751) | 0.007 (0.269) | -45.668 | < 0.001 |
| Amygdala R - Cuneus R | -1.112 (0.836) | -0.161 (0.233) | -34.623 | < 0.001 |
| Amygdala R - Occipital Sup L | -1.324 (0.886) | 0.673 (0.255) | -68.502 | < 0.001 |
| Amygdala R - Pallidum L | 1.049 (0.961) | 0.537 (0.266) | 16.254 | < 0.001 |
| Amygdala R - Heschl L | 1.23 (0.904) | -0.723 (0.228) | 66.250 | < 0.001 |
| Amygdala R - Temporal Sup L | 1.037 (0.932) | -0.515 (0.328) | 49.657 | < 0.001 |
| Amygdala R - Temporal Inf R | -1.36 (0.828) | -1.026 (0.281) | -12.068 | < 0.001 |
| Calcarine L - Occipital Mid L | 1.351 (0.873) | 1.239 (0.243) | 3.898 | < 0.001 |
| Calcarine L - Occipital Mid R | 1.229 (0.905) | 0.153 (0.247) | 36.285 | < 0.001 |
| Calcarine L - Occipital Inf L | 1.208 (0.935) | 0.593 (0.282) | 19.922 | < 0.001 |
| Calcarine L - Postcentral L | -1.16 (0.827) | -2.185 (0.223) | 37.828 | < 0.001 |
| Calcarine L - Cerebelum 4 5 L | 1.431 (0.982) | 0.027 (0.315) | 43.043 | < 0.001 |
| Calcarine L - Cerebelum 6 L | 1.225 (0.877) | -0.552 (0.258) | 61.482 | < 0.001 |
| Calcarine L - Vermis 3 | 1.517 (0.861) | 0.777 (0.291) | 25.746 | < 0.001 |
| Calcarine L - Vermis 6 | 1.258 (0.9) | 0.043 (0.236) | 41.313 | < 0.001 |
| Calcarine L - Vermis 7 | 1.086 (0.909) | -0.257 (0.247) | 45.102 | < 0.001 |
| Calcarine R - Postcentral L | -1.475 (0.772) | -2.565 (0.218) | 42.988 | < 0.001 |
| Calcarine R - Postcentral R | -1.16 (0.847) | -2.415 (0.207) | 45.529 | < 0.001 |
| Calcarine R - Cerebelum 4 5 L | 1.194 (1.002) | -1.004 (0.279) | 66.811 | < 0.001 |
| Calcarine R - Cerebelum 4 5 R | 1.114 (0.88) | -0.278 (0.255) | 48.029 | < 0.001 |
| Cuneus L - Postcentral L | -1.615 (0.868) | -2.149 (0.226) | 18.832 | < 0.001 |
| Cuneus L - Postcentral R | -1.631 (0.9) | -2.149 (0.237) | 17.595 | < 0.001 |
| Cuneus L - Parietal Sup R | -1.931 (0.819) | -2.2 (0.259) | 9.925 | < 0.001 |
| Cuneus L - Angular L | 1.401 (0.841) | 1.3 (0.31) | 3.567 | 0.001 |
| Cuneus L - Paracentral Lobule L | -1.261 (0.892) | -1.651 (0.267) | 13.238 | < 0.001 |
| Cuneus L - Paracentral Lobule R | -1.347 (0.817) | -1.946 (0.232) | 22.289 | < 0.001 |
| Cuneus L - Temporal Pole Sup R | -1.352 (0.802) | -1.793 (0.241) | 16.676 | < 0.001 |
| Cuneus L - Vermis 10 | 1.516 (0.799) | -1.075 (0.219) | 98.855 | < 0.001 |
| Cuneus R - Occipital Sup L | -1.198 (0.876) | -1.487 (0.254) | 10.028 | < 0.001 |
| Cuneus R - Postcentral R | -1.368 (0.879) | -2.388 (0.243) | 35.393 | < 0.001 |
| Cuneus R - Parietal Sup L | -1.315 (0.816) | -1.622 (0.247) | 11.387 | < 0.001 |
| Cuneus R - Parietal Sup R | -1.487 (0.862) | -1.859 (0.254) | 13.107 | < 0.001 |
| Cuneus R - Cerebelum 3 R | 1.372 (1.037) | -1.601 (0.276) | 87.623 | < 0.001 |
| Cuneus R - Cerebelum 4 5 L | 1.083 (1.041) | -0.731 (0.251) | 53.574 | < 0.001 |
| Cuneus R - Vermis 3 | 1.471 (0.899) | -0.626 (0.248) | 71.093 | < 0.001 |
| Cuneus R - Vermis 4 5 | 1.229 (0.977) | -0.105 (0.228) | 42.045 | < 0.001 |
| Cuneus R - Vermis 10 | 1.572 (1.001) | -0.099 (0.214) | 51.636 | < 0.001 |
| Lingual L - Vermis 3 | 1.136 (0.968) | 1.292 (0.328) | -4.804 | < 0.001 |
| Lingual L - Vermis 6 | 1.514 (0.886) | 0.641 (0.284) | 29.684 | < 0.001 |
| Lingual L - Vermis 9 | 1.111 (0.812) | 0.486 (0.232) | 23.420 | < 0.001 |
| Lingual R - Occipital Sup R | -1.218 (0.847) | -0.444 (0.275) | -27.500 | < 0.001 |
| Lingual R - Occipital Inf L | 1.054 (0.917) | 0.073 (0.31) | 32.028 | < 0.001 |
| Lingual R - Postcentral L | -1.502 (0.788) | -2.879 (0.215) | 53.324 | < 0.001 |
| Lingual R - Cerebelum Crus1 R | 1.219 (0.844) | 0.114 (0.224) | 40.006 | < 0.001 |
| Lingual R - Cerebelum 6 R | 1.424 (0.831) | 0.573 (0.302) | 30.431 | < 0.001 |
| Lingual R - Vermis 3 | 1.213 (0.963) | 1.613 (0.354) | -12.336 | < 0.001 |
| Lingual R - Vermis 4 5 | 1.23 (0.972) | 0.75 (0.256) | 15.100 | < 0.001 |
| Lingual R - Vermis 6 | 1.624 (0.931) | 0.396 (0.309) | 39.567 | < 0.001 |
| Lingual R - Vermis 9 | 1.246 (0.847) | 0.429 (0.241) | 29.328 | < 0.001 |
| Lingual R - Vermis 10 | 1.077 (1.001) | 0.848 (0.246) | 7.042 | < 0.001 |
| Occipital Sup L - Postcentral R | -1.264 (0.907) | -2.367 (0.233) | 37.281 | < 0.001 |
| Occipital Sup L - Temporal Pole Sup L | -1.555 (0.791) | -1.104 (0.219) | -17.374 | < 0.001 |
| Occipital Sup L - Temporal Pole Mid R | -1.409 (0.875) | -0.336 (0.254) | -37.242 | < 0.001 |
| Occipital Sup L - Vermis 10 | 1.13 (0.972) | -0.07 (0.243) | 37.861 | < 0.001 |
| Occipital Sup R - Postcentral R | -1.933 (0.869) | -2.018 (0.233) | 3.009 | 0.004 |
| Occipital Sup R - Parietal Sup R | -1.142 (0.862) | -1.014 (0.29) | -4.454 | < 0.001 |
| Occipital Sup R - Paracentral Lobule R | -1.301 (0.798) | -1.612 (0.255) | 11.721 | < 0.001 |
| Occipital Sup R - Temporal Pole Sup L | -1.196 (0.839) | -1.071 (0.24) | -4.533 | < 0.001 |
| Occipital Sup R - Vermis 3 | 1.224 (0.921) | 0.398 (0.281) | 27.133 | < 0.001 |
| Occipital Sup R - Vermis 4 5 | 1.126 (0.998) | -0.507 (0.245) | 50.240 | < 0.001 |
| Occipital Mid L - Postcentral L | -1.196 (0.858) | -3.347 (0.23) | 76.551 | < 0.001 |
| Occipital Mid L - Postcentral R | -1.544 (0.863) | -2.968 (0.213) | 50.671 | < 0.001 |
| Occipital Mid L - Parietal Sup L | -1.388 (0.911) | -2.577 (0.226) | 40.013 | < 0.001 |
| Occipital Mid L - Parietal Sup R | -1.354 (0.882) | -3.694 (0.248) | 80.772 | < 0.001 |
| Occipital Mid L - Angular L | 1.166 (0.844) | 1.344 (0.286) | -6.314 | < 0.001 |
| Occipital Mid L - Cerebelum 3 R | 1.369 (1.018) | -0.279 (0.231) | 49.911 | < 0.001 |
| Occipital Mid L - Cerebelum 4 5 L | 2.055 (1.052) | -0.145 (0.271) | 64.061 | < 0.001 |
| Occipital Mid L - Cerebelum 4 5 R | 1.99 (0.944) | 0.159 (0.233) | 59.565 | < 0.001 |
| Occipital Mid L - Cerebelum 6 L | 1.421 (0.93) | -0.334 (0.307) | 56.705 | < 0.001 |
| Occipital Mid L - Vermis 3 | 1.937 (0.871) | 0.671 (0.243) | 44.286 | < 0.001 |
| Occipital Mid L - Vermis 6 | 1.159 (0.91) | 0.428 (0.244) | 24.517 | < 0.001 |
| Occipital Mid L - Vermis 10 | 1.269 (0.961) | 0.074 (0.214) | 38.370 | < 0.001 |
| Occipital Mid R - Postcentral R | -1.696 (0.868) | -2.547 (0.237) | 29.904 | < 0.001 |
| Occipital Mid R - Parietal Sup R | -1.359 (0.877) | -2.214 (0.26) | 29.564 | < 0.001 |
| Occipital Mid R - Heschl L | 1.172 (0.881) | -0.69 (0.249) | 64.328 | < 0.001 |
| Occipital Mid R - Cerebelum Crus1 R | 1.114 (0.852) | -1.473 (0.283) | 91.090 | < 0.001 |
| Occipital Mid R - Cerebelum 4 5 L | 1.55 (1.065) | -0.977 (0.297) | 72.295 | < 0.001 |
| Occipital Mid R - Cerebelum 4 5 R | 1.499 (0.991) | -0.717 (0.236) | 68.793 | < 0.001 |
| Occipital Mid R - Vermis 3 | 1.29 (0.899) | -0.767 (0.205) | 70.512 | < 0.001 |
| Occipital Mid R - Vermis 4 5 | 1.442 (1.012) | -0.236 (0.212) | 51.332 | < 0.001 |
| Occipital Inf L - Cerebelum Crus1 L | 1.196 (0.878) | 0.027 (0.312) | 39.663 | < 0.001 |
| Occipital Inf L - Cerebelum 6 L | 1.365 (0.907) | 1.052 (0.353) | 10.180 | < 0.001 |
| Occipital Inf L - Cerebelum 6 R | 1.311 (0.883) | 0.73 (0.28) | 19.831 | < 0.001 |
| Occipital Inf L - Vermis 3 | 1.257 (0.925) | 1.563 (0.325) | -9.860 | < 0.001 |
| Occipital Inf R - Temporal Mid R | 1.185 (0.739) | -0.351 (0.242) | 62.478 | < 0.001 |
| Occipital Inf R - Cerebelum 4 5 L | 1.651 (1.003) | 0.617 (0.337) | 30.894 | < 0.001 |
| Occipital Inf R - Cerebelum 4 5 R | 1.458 (0.965) | 1.017 (0.333) | 13.656 | < 0.001 |
| Occipital Inf R - Cerebelum 6 R | 1.097 (0.855) | 1.362 (0.299) | -9.258 | < 0.001 |
| Occipital Inf R - Vermis 4 5 | 1.118 (0.922) | 0.067 (0.306) | 34.219 | < 0.001 |
| Occipital Inf R - Vermis 10 | 1.271 (1.061) | 1.757 (0.249) | -14.119 | < 0.001 |
| Fusiform L - Postcentral L | -1.462 (0.883) | -2.239 (0.226) | 26.981 | < 0.001 |
| Fusiform L - Postcentral R | -1.55 (0.799) | -1.982 (0.231) | 16.441 | < 0.001 |
| Fusiform L - Parietal Sup L | -1.366 (0.76) | -1.164 (0.226) | -8.026 | < 0.001 |
| Fusiform L - Parietal Sup R | -1.392 (0.799) | -1.088 (0.258) | -11.462 | < 0.001 |
| Fusiform L - Cerebelum 8 L | -1.336 (0.916) | -1.435 (0.315) | 3.251 | 0.002 |
| Fusiform L - Cerebelum 10 L | 1.087 (1.015) | 1.406 (0.242) | -9.682 | < 0.001 |
| Fusiform R - Postcentral R | -1.253 (0.792) | -1.61 (0.24) | 13.675 | < 0.001 |
| Fusiform R - Paracentral Lobule R | -1.131 (0.77) | -0.496 (0.25) | -24.828 | < 0.001 |
| Fusiform R - Pallidum R | -1.086 (0.852) | 1.618 (0.293) | -94.932 | < 0.001 |
| Fusiform R - Cerebelum 3 L | 1.376 (0.962) | 0.677 (0.28) | 22.068 | < 0.001 |
| Fusiform R - Cerebelum 4 5 L | 1.494 (0.942) | -0.126 (0.284) | 52.051 | < 0.001 |
| Fusiform R - Cerebelum 6 R | 1.169 (0.792) | 0.723 (0.337) | 16.388 | < 0.001 |
| Fusiform R - Cerebelum 10 L | 1.191 (0.977) | 1.508 (0.294) | -9.853 | < 0.001 |
| Fusiform R - Vermis 3 | 1.786 (0.846) | 1.175 (0.272) | 21.756 | < 0.001 |
| Fusiform R - Vermis 4 5 | 1.862 (0.883) | -0.359 (0.258) | 76.346 | < 0.001 |
| Postcentral L - Pallidum R | 1.258 (0.833) | 0.752 (0.226) | 18.549 | < 0.001 |
| Postcentral L - Temporal Inf R | -1.184 (0.825) | -0.865 (0.26) | -11.672 | < 0.001 |
| Postcentral R - Parietal Inf R | 1.549 (0.753) | 1.948 (0.279) | -15.742 | < 0.001 |
| Parietal Sup L - Temporal Mid L | -1.168 (0.779) | -0.204 (0.226) | -37.553 | < 0.001 |
| Parietal Sup L - Vermis 10 | -1.168 (0.814) | 0.324 (0.266) | -55.103 | < 0.001 |
| Parietal Sup R - Putamen R | 1.099 (1.01) | 0.331 (0.259) | 23.284 | < 0.001 |
| Parietal Sup R - Temporal Pole Sup L | -1.304 (0.847) | -0.889 (0.234) | -14.929 | < 0.001 |
| Parietal Sup R - Temporal Inf R | -1.127 (0.803) | -1.38 (0.246) | 9.539 | < 0.001 |
| Parietal Sup R - Cerebelum 6 L | -1.196 (0.849) | 0.307 (0.283) | -53.125 | < 0.001 |
| Parietal Inf L - Angular L | 1.137 (0.783) | 0.447 (0.239) | 26.664 | < 0.001 |
| Parietal Inf L - Temporal Inf R | -1.176 (0.815) | -1.958 (0.245) | 29.060 | < 0.001 |
| Parietal Inf L - Vermis 8 | -1.347 (0.827) | 2.394 (0.284) | -135.289 | < 0.001 |
| Parietal Inf R - Pallidum L | -1.241 (0.872) | 2.317 (0.324) | -121.018 | < 0.001 |
| SupraMarginal L - Temporal Mid R | -1.128 (0.872) | 0.607 (0.265) | -60.236 | < 0.001 |
| SupraMarginal L - Temporal Inf R | -1.256 (0.837) | -0.593 (0.232) | -24.170 | < 0.001 |
| SupraMarginal R - Heschl R | -1.285 (0.81) | 0.004 (0.261) | -47.907 | < 0.001 |
| SupraMarginal R - Temporal Sup L | -1.125 (0.767) | 0.199 (0.25) | -51.885 | < 0.001 |
| SupraMarginal R - Temporal Sup R | -1.467 (0.822) | -0.567 (0.274) | -32.835 | < 0.001 |
| SupraMarginal R - Temporal Mid L | -1.106 (0.804) | 1.515 (0.267) | -97.854 | < 0.001 |
| SupraMarginal R - Temporal Mid R | -1.132 (0.848) | 0.697 (0.286) | -64.697 | < 0.001 |
| Angular L - Precuneus R | 1.143 (0.885) | 1.457 (0.23) | -10.868 | < 0.001 |
| Angular R - Temporal Pole Sup L | -2.352 (0.819) | 0.841 (0.253) | -117.727 | < 0.001 |
| Angular R - Temporal Pole Sup R | -1.528 (0.864) | 0.67 (0.248) | -77.277 | < 0.001 |
| Precuneus L - Putamen R | 1.135 (0.922) | -0.814 (0.215) | 65.107 | < 0.001 |
| Precuneus L - Vermis 10 | -1.213 (0.797) | -0.514 (0.246) | -26.511 | < 0.001 |
| Precuneus R - Temporal Pole Sup L | -1.133 (0.897) | -0.134 (0.254) | -33.869 | < 0.001 |
| Caudate R - Temporal Pole Mid L | 1.213 (0.87) | 2.076 (0.251) | -30.148 | < 0.001 |
| Caudate R - Cerebelum 8 R | 1.649 (0.807) | 1.406 (0.28) | 9.001 | < 0.001 |
| Putamen L - Putamen R | -1.393 (0.877) | -0.919 (0.291) | -16.237 | < 0.001 |
| Thalamus L - Cerebelum 4 5 L | 1.254 (0.866) | -0.16 (0.28) | 49.128 | < 0.001 |
| Heschl L - Temporal Sup L | 1.129 (0.811) | -0.07 (0.267) | 44.408 | < 0.001 |
| Temporal Sup L - Cerebelum 4 5 L | 1.243 (0.934) | 1.481 (0.249) | -7.806 | < 0.001 |
| Temporal Sup L - Cerebelum 6 L | 1.377 (0.903) | 0.829 (0.246) | 18.491 | < 0.001 |
| Temporal Sup R - Vermis 9 | 1.258 (0.932) | -0.09 (0.226) | 44.463 | < 0.001 |
| Temporal Pole Sup R - Temporal Mid L | -1.186 (0.832) | -0.395 (0.271) | -28.573 | < 0.001 |
| Temporal Pole Sup R - Cerebelum 3 R | 1.159 (0.844) | 0.049 (0.307) | 39.100 | < 0.001 |
| Temporal Mid R - Vermis 6 | 1.577 (0.824) | 1.196 (0.292) | 13.803 | < 0.001 |
| Cerebelum Crus1 L - Cerebelum 6 L | 1.16 (0.874) | 0.943 (0.284) | 7.491 | < 0.001 |
| Cerebelum Crus1 R - Cerebelum 7b R | -1.145 (0.824) | -0.656 (0.337) | -17.353 | < 0.001 |
| Cerebelum 4 5 R - Vermis 4 5 | 1.629 (0.91) | 1.128 (0.25) | 16.776 | < 0.001 |
| Cerebelum 4 5 R - Vermis 7 | 1.387 (0.898) | -1.138 (0.242) | 85.849 | < 0.001 |
| Vermis 1 2 - Vermis 7 | -1.186 (0.851) | 0.469 (0.336) | -57.183 | < 0.001 |
| Vermis 3 - Vermis 6 | 1.138 (0.87) | -0.223 (0.263) | 47.329 | < 0.001 |
| Vermis 4 5 - Vermis 6 | 1.127 (0.785) | -0.698 (0.242) | 70.279 | < 0.001 |
| Vermis 4 5 - Vermis 7 | 1.27 (0.906) | -0.987 (0.276) | 75.382 | < 0.001 |
| Vermis 6 - Vermis 7 | 1.438 (0.895) | -0.093 (0.318) | 50.984 | < 0.001 |
| Precentral L - Calcarine L | -0.905 (0.836) | -2.315 (0.208) | 51.743 | < 0.001 |
| Precentral L - Calcarine R | -0.92 (0.828) | -2.191 (0.241) | 46.627 | < 0.001 |
| Precentral L - Cuneus R | -0.503 (0.851) | -2.62 (0.242) | 75.737 | < 0.001 |
| Precentral L - Occipital Sup R | -1.078 (0.879) | -2.638 (0.23) | 54.310 | < 0.001 |
| Precentral L - Occipital Mid R | -0.704 (0.84) | -3.29 (0.289) | 92.088 | < 0.001 |
| Precentral L - Occipital Inf L | -0.921 (0.77) | -2.927 (0.266) | 77.855 | < 0.001 |
| Precentral L - Occipital Inf R | -0.518 (0.839) | -2.021 (0.274) | 53.854 | < 0.001 |
| Precentral L - Parietal Sup R | -0.106 (0.859) | -2.185 (0.311) | 71.979 | < 0.001 |
| Precentral L - Pallidum L | 0.102 (0.823) | 2.247 (0.295) | -77.603 | < 0.001 |
| Precentral R - Frontal Inf Oper L | 0.214 (0.827) | -2.862 (0.247) | 112.721 | < 0.001 |
| Precentral R - Frontal Inf Oper R | 0.346 (0.892) | -1.966 (0.262) | 78.632 | < 0.001 |
| Precentral R - Frontal Inf Tri L | -0.274 (0.817) | -2.738 (0.215) | 92.198 | < 0.001 |
| Precentral R - Frontal Inf Tri R | 0.27 (0.87) | -2.089 (0.199) | 83.598 | < 0.001 |
| Precentral R - Calcarine L | -1.059 (0.833) | -2.454 (0.207) | 51.400 | < 0.001 |
| Precentral R - Lingual L | -0.909 (0.818) | -2.168 (0.201) | 47.276 | < 0.001 |
| Precentral R - Lingual R | -1 (0.85) | -2.542 (0.209) | 55.689 | < 0.001 |
| Precentral R - Occipital Inf L | -0.863 (0.768) | -2.994 (0.221) | 84.332 | < 0.001 |
| Precentral R - Fusiform R | -0.845 (0.8) | -2.082 (0.229) | 46.992 | < 0.001 |
| Precentral R - Temporal Pole Sup R | -0.484 (0.803) | -2.04 (0.199) | 59.528 | < 0.001 |
| Precentral R - Cerebelum Crus1 L | -0.652 (0.806) | 1.912 (0.275) | -95.260 | < 0.001 |
| Precentral R - Cerebelum 9 L | -0.175 (0.822) | 2.279 (0.283) | -89.282 | < 0.001 |
| Precentral R - Cerebelum 10 R | -0.459 (0.874) | 1.907 (0.3) | -80.915 | < 0.001 |
| Precentral R - Vermis 8 | -0.526 (0.808) | 1.981 (0.257) | -93.510 | < 0.001 |
| Frontal Sup L - Cingulum Post L | -0.552 (0.892) | -2.444 (0.226) | 64.998 | < 0.001 |
| Frontal Sup L - Angular L | -0.419 (0.907) | -1.965 (0.251) | 51.916 | < 0.001 |
| Frontal Sup L - Vermis 1 2 | -0.49 (0.764) | -2.08 (0.237) | 62.879 | < 0.001 |
| Frontal Sup L - Vermis 9 | -0.86 (0.775) | 1.916 (0.249) | -107.843 | < 0.001 |
| Frontal Sup R - Cingulum Post L | -0.447 (0.853) | -3.007 (0.249) | 91.081 | < 0.001 |
| Frontal Sup R - Cingulum Post R | -0.099 (0.832) | -2.793 (0.256) | 97.896 | < 0.001 |
| Frontal Sup R - Hippocampus L | 0.21 (0.791) | 2.33 (0.294) | -79.462 | < 0.001 |
| Frontal Sup R - Calcarine L | -0.707 (0.921) | -1.925 (0.254) | 40.318 | < 0.001 |
| Frontal Sup R - Lingual L | -0.004 (0.896) | -1.936 (0.238) | 65.933 | < 0.001 |
| Frontal Sup R - Occipital Inf L | -0.085 (0.881) | -2.088 (0.209) | 69.968 | < 0.001 |
| Frontal Sup R - Parietal Inf R | 0.01 (0.888) | -1.933 (0.251) | 66.594 | < 0.001 |
| Frontal Sup R - Angular L | -0.216 (0.835) | -1.983 (0.218) | 64.735 | < 0.001 |
| Frontal Sup R - Angular R | -0.589 (0.806) | -2.332 (0.24) | 65.527 | < 0.001 |
| Frontal Sup R - Precuneus L | -0.042 (0.899) | -2.107 (0.243) | 70.112 | < 0.001 |
| Frontal Sup R - Precuneus R | 0.318 (0.839) | -1.92 (0.261) | 80.514 | < 0.001 |
| Frontal Sup R - Caudate L | 0.334 (0.849) | 2.251 (0.276) | -67.902 | < 0.001 |
| Frontal Sup R - Caudate R | 0.127 (0.894) | 2.066 (0.293) | -65.189 | < 0.001 |
| Frontal Sup R - Pallidum R | -0.101 (0.793) | 1.987 (0.38) | -75.120 | < 0.001 |
| Frontal Sup R - Cerebelum 4 5 R | -0.16 (0.889) | -2.135 (0.275) | 67.097 | < 0.001 |
| Frontal Sup Orb L - Frontal Mid Orb R | 0.923 (0.854) | 2.051 (0.276) | -39.743 | < 0.001 |
| Frontal Sup Orb L - Parietal Inf R | 0.42 (0.791) | 1.941 (0.315) | -56.503 | < 0.001 |
| Frontal Sup Orb L - Thalamus L | 0.63 (0.812) | 2.2 (0.34) | -56.380 | < 0.001 |
| Frontal Sup Orb R - Frontal Inf Tri L | 0.571 (0.857) | -2.187 (0.234) | 98.147 | < 0.001 |
| Frontal Sup Orb R - Vermis 10 | 0.004 (0.737) | 2.884 (0.316) | -113.564 | < 0.001 |
| Frontal Mid L - Cingulum Post L | 0.139 (0.894) | -2.777 (0.236) | 99.721 | < 0.001 |
| Frontal Mid L - Cingulum Post R | 0.323 (0.9) | -2.28 (0.205) | 89.187 | < 0.001 |
| Frontal Mid L - Calcarine L | -0.369 (0.889) | -1.947 (0.268) | 53.723 | < 0.001 |
| Frontal Mid L - Angular R | 0.158 (0.874) | -2.208 (0.241) | 82.531 | < 0.001 |
| Frontal Mid L - Heschl R | -0.35 (0.861) | 2.001 (0.397) | -78.414 | < 0.001 |
| Frontal Mid L - Cerebelum Crus1 R | -0.62 (0.963) | -2.098 (0.279) | 46.588 | < 0.001 |
| Frontal Mid L - Cerebelum 7b R | -0.735 (0.864) | -2.248 (0.218) | 53.709 | < 0.001 |
| Frontal Mid R - Cingulum Post R | 0.377 (0.872) | -2.195 (0.23) | 90.152 | < 0.001 |
| Frontal Mid R - Hippocampus L | 0.498 (0.805) | 1.903 (0.275) | -52.231 | < 0.001 |
| Frontal Mid R - Thalamus R | -0.062 (0.858) | 2.158 (0.427) | -73.291 | < 0.001 |
| Frontal Mid R - Cerebelum 4 5 R | -0.356 (0.797) | -1.986 (0.22) | 62.318 | < 0.001 |
| Frontal Mid Orb L - Thalamus L | 0.229 (0.857) | 2.192 (0.29) | -68.651 | < 0.001 |
| Frontal Mid Orb L - Temporal Pole Sup R | 0.16 (0.918) | 2.003 (0.255) | -61.207 | < 0.001 |
| Frontal Mid Orb R - Occipital Sup R | -0.351 (0.868) | 2.097 (0.284) | -84.803 | < 0.001 |
| Frontal Mid Orb R - Postcentral L | 0.225 (0.853) | 2.489 (0.291) | -79.441 | < 0.001 |
| Frontal Mid Orb R - Thalamus L | 0.561 (0.878) | 2.471 (0.269) | -65.777 | < 0.001 |
| Frontal Mid Orb R - Vermis 3 | 0.791 (0.943) | 2.363 (0.331) | -49.725 | < 0.001 |
| Frontal Inf Oper L - Frontal Inf Orb R | -0.445 (0.856) | -2.062 (0.223) | 57.807 | < 0.001 |
| Frontal Inf Oper L - Parietal Inf L | 0.394 (0.824) | -2.331 (0.252) | 100.041 | < 0.001 |
| Frontal Inf Oper L - Temporal Mid L | -0.342 (0.778) | -2.395 (0.299) | 77.884 | < 0.001 |
| Frontal Inf Oper L - Temporal Inf R | -0.301 (0.938) | -1.945 (0.259) | 53.463 | < 0.001 |
| Frontal Inf Oper L - Cerebelum Crus1 R | -0.016 (0.854) | -1.922 (0.226) | 68.203 | < 0.001 |
| Frontal Inf Oper L - Cerebelum 6 L | -0.059 (0.824) | -2.118 (0.247) | 75.659 | < 0.001 |
| Frontal Inf Oper R - Thalamus R | -0.302 (0.797) | 1.992 (0.31) | -84.859 | < 0.001 |
| Frontal Inf Tri L - Frontal Inf Orb R | -0.118 (0.859) | -2.208 (0.238) | 74.184 | < 0.001 |
| Frontal Inf Tri L - Frontal Med Orb R | 0.106 (0.837) | -2.163 (0.253) | 82.046 | < 0.001 |
| Frontal Inf Tri L - Parietal Inf L | -0.042 (0.897) | -2.125 (0.265) | 70.392 | < 0.001 |
| Frontal Inf Tri L - Putamen R | 0.027 (0.897) | -1.952 (0.254) | 67.117 | < 0.001 |
| Frontal Inf Tri L - Cerebelum 6 L | 0.083 (0.884) | -1.986 (0.216) | 71.903 | < 0.001 |
| Frontal Inf Tri R - Supp Motor Area L | -0.128 (0.737) | -2.217 (0.266) | 84.311 | < 0.001 |
| Frontal Inf Orb L - Vermis 4 5 | 0.124 (0.81) | 2.111 (0.362) | -70.772 | < 0.001 |
| Frontal Inf Orb R - Lingual R | 0.409 (0.863) | 2.178 (0.325) | -60.673 | < 0.001 |
| Frontal Inf Orb R - Thalamus L | 0.034 (0.881) | 1.908 (0.345) | -62.662 | < 0.001 |
| Frontal Inf Orb R - Thalamus R | 0.203 (0.883) | 2.055 (0.298) | -62.856 | < 0.001 |
| Rolandic Oper L - Supp Motor Area R | -0.736 (0.819) | -2.188 (0.249) | 53.622 | < 0.001 |
| Rolandic Oper L - Amygdala R | 0.534 (0.905) | -1.969 (0.209) | 85.235 | < 0.001 |
| Rolandic Oper L - Heschl L | 0.663 (0.797) | -2.264 (0.232) | 111.519 | < 0.001 |
| Rolandic Oper L - Temporal Pole Sup R | -0.834 (0.776) | -3.292 (0.185) | 97.476 | < 0.001 |
| Rolandic Oper R - Rectus R | 0.63 (0.888) | 2.115 (0.303) | -50.033 | < 0.001 |
| Rolandic Oper R - Cingulum Ant L | -0.166 (0.759) | -2.306 (0.219) | 85.644 | < 0.001 |
| Rolandic Oper R - Temporal Pole Sup R | -0.303 (0.801) | -2.381 (0.219) | 79.154 | < 0.001 |
| Rolandic Oper R - Vermis 3 | -0.261 (0.85) | 1.932 (0.274) | -77.660 | < 0.001 |
| Supp Motor Area L - Occipital Sup R | -0.702 (0.884) | -2.085 (0.218) | 48.007 | < 0.001 |
| Supp Motor Area L - Occipital Mid L | -0.992 (0.899) | -2.333 (0.234) | 45.634 | < 0.001 |
| Supp Motor Area L - Occipital Mid R | -0.938 (0.913) | -2.259 (0.249) | 44.164 | < 0.001 |
| Supp Motor Area L - Temporal Sup R | -0.178 (0.802) | -2.02 (0.235) | 69.708 | < 0.001 |
| Supp Motor Area R - Occipital Sup L | -0.859 (0.898) | -2.062 (0.233) | 41.018 | < 0.001 |
| Supp Motor Area R - SupraMarginal R | -0.821 (0.881) | -2.01 (0.262) | 40.919 | < 0.001 |
| Supp Motor Area R - Angular R | -0.414 (0.819) | -2.119 (0.221) | 63.583 | < 0.001 |
| Supp Motor Area R - Cerebelum Crus1 L | 0.486 (0.864) | 2.802 (0.295) | -80.208 | < 0.001 |
| Supp Motor Area R - Vermis 7 | -0.171 (0.847) | 2.227 (0.287) | -84.827 | < 0.001 |
| Olfactory L - Temporal Pole Sup L | 0.242 (0.843) | -1.933 (0.296) | 76.977 | < 0.001 |
| Olfactory L - Temporal Pole Sup R | -0.547 (0.787) | -2.591 (0.305) | 76.579 | < 0.001 |
| Olfactory L - Vermis 4 5 | 0.329 (0.864) | 3.014 (0.314) | -92.405 | < 0.001 |
| Frontal Sup Medial L - Cingulum Post L | -0.353 (0.85) | -2.592 (0.235) | 80.291 | < 0.001 |
| Frontal Sup Medial L - Cingulum Post R | -0.336 (0.844) | -2.164 (0.234) | 65.969 | < 0.001 |
| Frontal Sup Medial L - Pallidum R | -0.743 (0.896) | 2.531 (0.357) | -107.334 | < 0.001 |
| Frontal Sup Medial L - Vermis 8 | -0.407 (0.846) | 2.357 (0.267) | -98.555 | < 0.001 |
| Frontal Sup Medial L - Vermis 9 | -0.617 (0.772) | 2.985 (0.282) | -138.595 | < 0.001 |
| Frontal Sup Medial R - Temporal Mid R | -0.84 (0.882) | -2.433 (0.24) | 55.122 | < 0.001 |
| Frontal Sup Medial R - Cerebelum 10 L | 0.314 (0.927) | 2.064 (0.309) | -56.620 | < 0.001 |
| Frontal Sup Medial R - Vermis 3 | 0.045 (0.819) | 2.087 (0.289) | -74.356 | < 0.001 |
| Frontal Sup Medial R - Vermis 9 | -0.131 (0.757) | 2.208 (0.309) | -90.489 | < 0.001 |
| Frontal Med Orb L - Fusiform R | -0.353 (0.921) | -2.105 (0.209) | 58.679 | < 0.001 |
| Frontal Med Orb L - Temporal Pole Sup R | 0.078 (0.931) | -1.954 (0.312) | 65.404 | < 0.001 |
| Frontal Med Orb L - Cerebelum Crus2 L | -0.277 (0.828) | -2.276 (0.251) | 73.040 | < 0.001 |
| Frontal Med Orb R - Cingulum Post L | -0.654 (0.885) | -2.099 (0.404) | 46.976 | < 0.001 |
| Frontal Med Orb R - Cerebelum Crus2 L | -0.273 (0.795) | -2.023 (0.271) | 65.854 | < 0.001 |
| Rectus L - SupraMarginal L | 0.411 (0.763) | 1.91 (0.222) | -59.658 | < 0.001 |
| Rectus L - Temporal Pole Mid R | 0.76 (0.969) | -2.017 (0.278) | 87.089 | < 0.001 |
| Rectus L - Cerebelum 9 R | 0.82 (0.854) | 1.891 (0.371) | -36.407 | < 0.001 |
| Rectus L - Vermis 10 | 0.604 (0.769) | 2.383 (0.278) | -68.771 | < 0.001 |
| Rectus R - Insula L | -0.161 (0.844) | 3.812 (0.275) | -141.507 | < 0.001 |
| Rectus R - Insula R | -0.657 (0.825) | 3.769 (0.309) | -158.953 | < 0.001 |
| Rectus R - Lingual R | 1.074 (0.856) | 2.004 (0.309) | -32.310 | < 0.001 |
| Rectus R - Parietal Inf R | 0.664 (0.879) | 2.095 (0.322) | -48.358 | < 0.001 |
| Rectus R - SupraMarginal L | 0.459 (0.788) | 2.308 (0.314) | -68.886 | < 0.001 |
| Insula L - Cuneus L | -0.156 (0.795) | 2.08 (0.233) | -85.392 | < 0.001 |
| Insula L - Occipital Sup R | 0.121 (0.927) | 2.09 (0.318) | -63.543 | < 0.001 |
| Insula L - Fusiform R | -0.684 (0.79) | 2.116 (0.284) | -105.451 | < 0.001 |
| Insula L - Parietal Sup L | -0.165 (0.819) | 2.273 (0.317) | -87.769 | < 0.001 |
| Insula L - Parietal Sup R | -0.01 (0.821) | 2.335 (0.316) | -84.269 | < 0.001 |
| Insula L - Parietal Inf R | 0.419 (0.93) | 1.938 (0.424) | -47.018 | < 0.001 |
| Insula L - Cerebelum 6 R | -0.347 (0.787) | 2.67 (0.273) | -114.576 | < 0.001 |
| Insula R - Lingual R | -0.313 (0.849) | 2.469 (0.269) | -98.802 | < 0.001 |
| Cingulum Ant R - Temporal Sup L | 0.07 (0.759) | -2.129 (0.227) | 87.755 | < 0.001 |
| Cingulum Mid L - Calcarine L | -0.347 (0.882) | -2.602 (0.201) | 78.798 | < 0.001 |
| Cingulum Mid L - Cuneus R | 0.014 (0.923) | -2.319 (0.242) | 77.330 | < 0.001 |
| Cingulum Mid L - Lingual R | -0.448 (0.912) | -2.037 (0.248) | 53.160 | < 0.001 |
| Cingulum Mid L - Occipital Sup L | 0.192 (0.957) | -2.264 (0.208) | 79.354 | < 0.001 |
| Cingulum Mid L - Occipital Sup R | 0.245 (0.86) | -2.839 (0.193) | 110.681 | < 0.001 |
| Cingulum Mid L - Occipital Mid L | -0.424 (0.878) | -2.327 (0.22) | 66.496 | < 0.001 |
| Cingulum Mid L - Temporal Sup R | -0.788 (0.845) | -2.633 (0.238) | 66.437 | < 0.001 |
| Cingulum Mid L - Temporal Pole Sup R | -0.726 (0.836) | -2.176 (0.236) | 52.814 | < 0.001 |
| Cingulum Mid L - Cerebelum 4 5 R | -0.797 (0.893) | -1.997 (0.205) | 41.408 | < 0.001 |
| Cingulum Mid R - Hippocampus L | -0.317 (0.917) | 2.061 (0.284) | -78.302 | < 0.001 |
| Cingulum Mid R - Occipital Mid L | -0.13 (0.845) | -2.225 (0.22) | 75.829 | < 0.001 |
| Cingulum Mid R - SupraMarginal L | -0.546 (0.795) | -2.679 (0.231) | 81.484 | < 0.001 |
| Cingulum Mid R - Temporal Sup L | -0.815 (0.793) | -1.988 (0.22) | 45.089 | < 0.001 |
| Cingulum Mid R - Temporal Pole Sup L | -0.476 (0.798) | -2.027 (0.257) | 58.493 | < 0.001 |
| Cingulum Post L - Fusiform R | 0.288 (0.838) | -1.986 (0.223) | 82.947 | < 0.001 |
| Cingulum Post L - Cerebelum Crus1 R | -0.212 (0.832) | -2.743 (0.229) | 92.692 | < 0.001 |
| Cingulum Post L - Cerebelum Crus2 L | -1.134 (0.834) | -2.844 (0.251) | 62.087 | < 0.001 |
| Cingulum Post L - Cerebelum 8 R | 0.27 (0.871) | -2.355 (0.222) | 92.342 | < 0.001 |
| Cingulum Post L - Cerebelum 9 R | -0.593 (0.838) | -2.692 (0.285) | 75.002 | < 0.001 |
| Cingulum Post R - Temporal Pole Mid L | 0.904 (0.932) | 2.116 (0.331) | -38.746 | < 0.001 |
| Cingulum Post R - Cerebelum Crus1 R | 0.036 (0.894) | -1.969 (0.227) | 68.729 | < 0.001 |
| Hippocampus L - SupraMarginal L | 0.422 (0.993) | 2.344 (0.386) | -57.020 | < 0.001 |
| Hippocampus L - Vermis 1 2 | 0.785 (0.875) | 2.012 (0.325) | -41.561 | < 0.001 |
| Hippocampus L - Vermis 3 | 0.106 (0.898) | 2.144 (0.335) | -67.212 | < 0.001 |
| Hippocampus R - Amygdala L | -0.053 (0.834) | -2.332 (0.266) | 82.314 | < 0.001 |
| Hippocampus R - Parietal Inf R | 0.26 (0.881) | 2.005 (0.345) | -58.343 | < 0.001 |
| Hippocampus R - SupraMarginal L | -0.082 (0.821) | 2.289 (0.28) | -86.472 | < 0.001 |
| Hippocampus R - Angular R | 0.248 (0.859) | 2.352 (0.288) | -73.405 | < 0.001 |
| ParaHippocampal L - Temporal Pole Sup L | -0.544 (0.875) | -2.224 (0.275) | 57.911 | < 0.001 |
| ParaHippocampal R - Amygdala R | -0.697 (0.819) | -2.156 (0.252) | 53.839 | < 0.001 |
| ParaHippocampal R - Caudate R | 1.038 (0.839) | 2.059 (0.254) | -36.851 | < 0.001 |
| Amygdala L - Cerebelum 7b R | 0.27 (0.797) | 2.044 (0.263) | -66.867 | < 0.001 |
| Amygdala R - Temporal Pole Mid R | -0.563 (0.807) | -2.93 (0.226) | 89.331 | < 0.001 |
| Amygdala R - Vermis 9 | 0.518 (0.825) | 1.934 (0.286) | -51.282 | < 0.001 |
| Calcarine L - Postcentral R | -0.788 (0.871) | -2.254 (0.216) | 51.670 | < 0.001 |
| Calcarine L - Parietal Sup R | -0.626 (0.848) | -1.94 (0.24) | 47.096 | < 0.001 |
| Calcarine L - Precuneus L | -0.497 (0.885) | -3.098 (0.285) | 88.506 | < 0.001 |
| Calcarine L - Precuneus R | -0.759 (0.905) | -2.749 (0.268) | 66.683 | < 0.001 |
| Calcarine L - Paracentral Lobule L | -0.814 (0.917) | -2.435 (0.245) | 54.008 | < 0.001 |
| Calcarine L - Cerebelum 9 R | 0.403 (0.76) | -2.35 (0.284) | 107.277 | < 0.001 |
| Calcarine R - Precuneus L | -0.823 (0.873) | -2.033 (0.262) | 41.978 | < 0.001 |
| Calcarine R - Paracentral Lobule L | -0.991 (0.866) | -2.419 (0.223) | 50.482 | < 0.001 |
| Calcarine R - Paracentral Lobule R | -0.74 (0.805) | -2.099 (0.228) | 51.386 | < 0.001 |
| Cuneus L - Precuneus R | 0.118 (0.733) | -2.226 (0.301) | 93.595 | < 0.001 |
| Cuneus L - Pallidum L | 0.067 (0.75) | 2.367 (0.254) | -91.804 | < 0.001 |
| Cuneus L - Cerebelum Crus1 R | -0.181 (0.813) | -2.285 (0.29) | 77.062 | < 0.001 |
| Cuneus L - Cerebelum 9 R | 0.514 (0.785) | -3.124 (0.226) | 140.912 | < 0.001 |
| Cuneus R - Lingual L | -0.708 (0.837) | -2.143 (0.242) | 52.097 | < 0.001 |
| Cuneus R - Postcentral L | -0.922 (0.821) | -2.908 (0.224) | 73.783 | < 0.001 |
| Cuneus R - Paracentral Lobule R | -0.773 (0.8) | -2.173 (0.252) | 52.812 | < 0.001 |
| Lingual L - Postcentral L | -1.084 (0.85) | -2.74 (0.199) | 59.978 | < 0.001 |
| Lingual L - Postcentral R | -1.094 (0.819) | -2.2 (0.195) | 41.535 | < 0.001 |
| Lingual L - Parietal Sup L | -1.182 (0.844) | -1.939 (0.244) | 27.224 | < 0.001 |
| Lingual L - Parietal Sup R | -0.725 (0.81) | -2.178 (0.223) | 54.698 | < 0.001 |
| Lingual L - Precuneus L | -0.297 (0.855) | -2.301 (0.243) | 71.320 | < 0.001 |
| Lingual L - Precuneus R | -0.884 (0.847) | -2.785 (0.253) | 68.064 | < 0.001 |
| Lingual L - Thalamus R | -0.008 (0.884) | -1.976 (0.258) | 67.607 | < 0.001 |
| Lingual L - Cerebelum 8 R | -0.387 (0.783) | -2.105 (0.241) | 66.319 | < 0.001 |
| Lingual R - Postcentral R | -0.827 (0.817) | -2.155 (0.232) | 49.434 | < 0.001 |
| Lingual R - Precuneus L | -0.424 (0.847) | -1.966 (0.242) | 55.371 | < 0.001 |
| Lingual R - Precuneus R | -0.645 (0.813) | -2.251 (0.251) | 59.681 | < 0.001 |
| Occipital Sup L - Postcentral L | -0.9 (0.883) | -2.651 (0.204) | 61.127 | < 0.001 |
| Occipital Sup L - Parietal Sup R | -0.886 (0.819) | -2.045 (0.237) | 43.010 | < 0.001 |
| Occipital Sup R - Occipital Inf L | -0.971 (0.855) | -1.957 (0.244) | 35.062 | < 0.001 |
| Occipital Sup R - Fusiform L | -0.831 (0.868) | -3.035 (0.23) | 77.568 | < 0.001 |
| Occipital Sup R - Fusiform R | -0.938 (0.886) | -2.245 (0.295) | 44.240 | < 0.001 |
| Occipital Sup R - Postcentral L | -0.978 (0.882) | -2.088 (0.209) | 38.746 | < 0.001 |
| Occipital Sup R - Angular L | 1.086 (0.837) | 1.919 (0.279) | -29.828 | < 0.001 |
| Occipital Sup R - Temporal Inf L | -0.696 (0.804) | -2.528 (0.237) | 69.102 | < 0.001 |
| Occipital Mid L - Occipital Inf R | -0.222 (0.83) | -2.03 (0.252) | 65.876 | < 0.001 |
| Occipital Mid L - Paracentral Lobule L | -0.623 (0.843) | -2.217 (0.224) | 57.814 | < 0.001 |
| Occipital Mid L - Paracentral Lobule R | -0.856 (0.794) | -2.066 (0.222) | 46.422 | < 0.001 |
| Occipital Mid R - Occipital Inf R | 0.158 (0.83) | -2.01 (0.278) | 78.376 | < 0.001 |
| Occipital Mid R - Fusiform L | -0.562 (0.923) | -2.233 (0.227) | 55.590 | < 0.001 |
| Occipital Mid R - Postcentral L | -0.862 (0.802) | -2.152 (0.24) | 48.759 | < 0.001 |
| Occipital Mid R - Putamen R | 0.317 (0.832) | 1.926 (0.381) | -55.618 | < 0.001 |
| Occipital Mid R - Temporal Inf L | -0.514 (0.84) | -2.422 (0.223) | 69.383 | < 0.001 |
| Occipital Inf L - Postcentral L | -0.919 (0.779) | -2.356 (0.219) | 56.120 | < 0.001 |
| Occipital Inf L - Postcentral R | -0.838 (0.782) | -2.164 (0.225) | 51.512 | < 0.001 |
| Occipital Inf L - Paracentral Lobule L | -0.585 (0.779) | -1.944 (0.249) | 52.538 | < 0.001 |
| Occipital Inf L - Cerebelum 8 R | -0.013 (0.853) | -2.016 (0.238) | 71.508 | < 0.001 |
| Occipital Inf R - Caudate R | 0.376 (0.938) | 2.707 (0.315) | -74.457 | < 0.001 |
| Occipital Inf R - Cerebelum 3 L | 0.65 (0.865) | 1.929 (0.32) | -43.867 | < 0.001 |
| Occipital Inf R - Cerebelum 6 L | 0.89 (0.903) | 1.963 (0.282) | -35.888 | < 0.001 |
| Fusiform L - Fusiform R | -1.009 (0.9) | -2.136 (0.32) | 37.318 | < 0.001 |
| Fusiform L - Thalamus R | 0.377 (0.91) | -2.629 (0.21) | 101.826 | < 0.001 |
| Fusiform L - Temporal Pole Mid R | -0.487 (0.816) | -2.101 (0.277) | 59.198 | < 0.001 |
| Fusiform L - Cerebelum 8 R | -1.131 (0.882) | -2.099 (0.238) | 33.515 | < 0.001 |
| Fusiform R - Postcentral L | -1.018 (0.766) | -2.139 (0.25) | 43.968 | < 0.001 |
| Fusiform R - Cerebelum 8 L | -0.331 (0.889) | -2.084 (0.273) | 59.634 | < 0.001 |
| Postcentral L - Temporal Sup R | -0.761 (0.76) | -1.999 (0.252) | 48.848 | < 0.001 |
| Postcentral L - Cerebelum Crus2 L | -0.152 (0.804) | 2.269 (0.257) | -90.737 | < 0.001 |
| Postcentral R - Pallidum L | -0.333 (0.849) | 2.1 (0.278) | -86.114 | < 0.001 |
| Postcentral R - Temporal Sup R | -0.827 (0.872) | -2.325 (0.262) | 52.006 | < 0.001 |
| Postcentral R - Temporal Pole Sup L | -0.9 (0.837) | -2.3 (0.259) | 50.563 | < 0.001 |
| Parietal Sup L - Pallidum R | -0.267 (0.835) | 1.892 (0.253) | -78.251 | < 0.001 |
| Parietal Inf L - Vermis 3 | 0.342 (1.018) | 2.235 (0.381) | -55.046 | < 0.001 |
| Parietal Inf R - SupraMarginal R | 0.764 (0.835) | 1.967 (0.37) | -41.664 | < 0.001 |
| Parietal Inf R - Precuneus L | 0.783 (0.761) | 2.56 (0.286) | -69.120 | < 0.001 |
| Parietal Inf R - Thalamus R | -0.776 (0.807) | 1.922 (0.323) | -98.142 | < 0.001 |
| Parietal Inf R - Temporal Mid L | -0.87 (0.892) | 2.062 (0.325) | -97.646 | < 0.001 |
| Parietal Inf R - Cerebelum 4 5 L | -0.392 (0.851) | 1.913 (0.292) | -81.002 | < 0.001 |
| SupraMarginal L - Precuneus L | 0.296 (0.808) | 2.085 (0.261) | -66.633 | < 0.001 |
| SupraMarginal L - Cerebelum 4 5 L | -0.488 (0.849) | 2.467 (0.286) | -104.331 | < 0.001 |
| SupraMarginal R - Pallidum L | -0.854 (0.852) | 2.153 (0.37) | -102.381 | < 0.001 |
| Angular L - Pallidum R | 0.135 (1.04) | 2.033 (0.333) | -54.962 | < 0.001 |
| Angular L - Vermis 3 | 0.141 (0.838) | 2.404 (0.34) | -79.165 | < 0.001 |
| Angular R - Temporal Inf L | -0.168 (0.894) | -1.943 (0.259) | 60.303 | < 0.001 |
| Angular R - Temporal Inf R | -0.591 (0.944) | -2.133 (0.254) | 49.873 | < 0.001 |
| Angular R - Cerebelum Crus1 L | -0.494 (0.888) | -2.226 (0.247) | 59.392 | < 0.001 |
| Angular R - Cerebelum Crus1 R | 0.131 (0.929) | -2.239 (0.274) | 77.401 | < 0.001 |
| Angular R - Cerebelum 10 L | -0.204 (0.878) | 2.265 (0.196) | -86.807 | < 0.001 |
| Precuneus L - Pallidum L | 0.878 (0.836) | 2.757 (0.232) | -68.458 | < 0.001 |
| Precuneus L - Temporal Inf R | -0.199 (0.836) | -2.093 (0.236) | 68.935 | < 0.001 |
| Precuneus L - Cerebelum Crus1 R | -0.311 (0.841) | -1.926 (0.286) | 57.520 | < 0.001 |
| Precuneus L - Cerebelum 9 R | 0.085 (0.773) | -2.213 (0.254) | 89.339 | < 0.001 |
| Precuneus R - Pallidum L | 0.596 (0.821) | 3.275 (0.28) | -97.631 | < 0.001 |
| Precuneus R - Pallidum R | -0.499 (0.86) | 2.505 (0.245) | -106.260 | < 0.001 |
| Precuneus R - Cerebelum Crus1 R | -0.101 (0.853) | -2.353 (0.286) | 79.184 | < 0.001 |
| Precuneus R - Cerebelum 8 R | -0.142 (0.752) | -2.26 (0.263) | 84.106 | < 0.001 |
| Paracentral Lobule R - Pallidum L | 0.125 (0.828) | 2.19 (0.269) | -74.964 | < 0.001 |
| Caudate L - Temporal Pole Mid R | 0.333 (0.89) | 2.239 (0.289) | -64.392 | < 0.001 |
| Caudate L - Temporal Inf R | 0.623 (0.838) | 2.047 (0.413) | -48.181 | < 0.001 |
| Caudate L - Cerebelum 7b R | 0.183 (0.855) | 2.099 (0.336) | -65.958 | < 0.001 |
| Caudate L - Cerebelum 8 L | 0.709 (0.964) | 1.922 (0.268) | -38.335 | < 0.001 |
| Caudate L - Vermis 10 | 0.104 (0.851) | 2.361 (0.286) | -79.479 | < 0.001 |
| Caudate R - Putamen L | 0.209 (0.786) | -2.077 (0.282) | 86.634 | < 0.001 |
| Caudate R - Temporal Pole Mid R | 1.014 (0.857) | 2.352 (0.263) | -47.176 | < 0.001 |
| Caudate R - Vermis 10 | 0.137 (0.958) | 2.004 (0.28) | -59.191 | < 0.001 |
| Putamen L - Temporal Pole Sup R | 0.127 (0.96) | -2.097 (0.272) | 70.540 | < 0.001 |
| Putamen L - Cerebelum 10 L | 0.17 (0.915) | 2.18 (0.309) | -65.825 | < 0.001 |
| Pallidum L - Thalamus R | -0.28 (0.816) | 2.475 (0.246) | -102.150 | < 0.001 |
| Pallidum L - Temporal Sup R | -0.377 (0.857) | 2.136 (0.27) | -88.395 | < 0.001 |
| Pallidum L - Temporal Mid R | -0.708 (0.893) | 2.171 (0.263) | -97.777 | < 0.001 |
| Pallidum R - Temporal Sup R | -0.506 (0.93) | 2.143 (0.257) | -86.862 | < 0.001 |
| Thalamus R - Temporal Sup R | 0.062 (0.778) | 2.125 (0.284) | -78.780 | < 0.001 |
| Thalamus R - Cerebelum 6 L | 0.479 (0.836) | -2.189 (0.224) | 97.495 | < 0.001 |
| Thalamus R - Vermis 10 | -0.74 (0.956) | 2.075 (0.257) | -89.884 | < 0.001 |
| Heschl L - Cerebelum 3 L | 0.348 (0.851) | -2.468 (0.191) | 102.147 | < 0.001 |
| Temporal Sup L - Cerebelum Crus1 L | 1.01 (0.994) | 2.025 (0.278) | -31.112 | < 0.001 |
| Temporal Sup L - Cerebelum 6 R | 0.873 (0.89) | 1.936 (0.349) | -35.169 | < 0.001 |
| Temporal Sup L - Vermis 3 | 0.436 (0.871) | 1.907 (0.255) | -51.306 | < 0.001 |
| Temporal Sup R - Vermis 3 | 0.855 (1.07) | 2.163 (0.293) | -37.277 | < 0.001 |
| Temporal Pole Sup L - Cerebelum 8 L | -0.3 (0.861) | -2.154 (0.234) | 65.646 | < 0.001 |
| Temporal Pole Sup R - Cerebelum 7b R | 0.132 (0.868) | 1.98 (0.39) | -61.392 | < 0.001 |
| Temporal Pole Sup R - Vermis 3 | 0.089 (0.95) | 2.272 (0.309) | -69.102 | < 0.001 |
| Temporal Mid L - Vermis 3 | 0.931 (0.91) | 2.268 (0.304) | -44.062 | < 0.001 |
| Temporal Mid R - Vermis 3 | 0.455 (0.849) | 2.248 (0.31) | -62.769 | < 0.001 |
| Temporal Inf L - Cerebelum 7b R | -0.442 (0.874) | -2.041 (0.232) | 55.914 | < 0.001 |
| Temporal Inf L - Cerebelum 8 R | -1.1 (0.826) | -1.96 (0.236) | 31.632 | < 0.001 |
| Temporal Inf R - Cerebelum 8 L | -0.719 (0.845) | -2.342 (0.254) | 58.167 | < 0.001 |
| Temporal Inf R - Vermis 9 | 0.076 (0.849) | 1.907 (0.255) | -65.365 | < 0.001 |
| Cerebelum Crus1 L - Cerebelum 7b R | -0.55 (0.851) | -2.294 (0.273) | 61.685 | < 0.001 |
| Cerebelum Crus1 L - Cerebelum 10 L | 0.081 (0.835) | 1.918 (0.297) | -65.556 | < 0.001 |
| Cerebelum Crus1 R - Cerebelum Crus2 L | -0.719 (0.85) | -2.181 (0.287) | 51.527 | < 0.001 |
| Cerebelum 3 R - Vermis 3 | 0.602 (0.867) | 2.202 (0.279) | -55.573 | < 0.001 |
| Cerebelum 3 R - Vermis 4 5 | -0.091 (1.012) | 2.246 (0.296) | -70.088 | < 0.001 |
| Cerebelum 4 5 L - Cerebelum 9 R | -0.196 (0.862) | -2.153 (0.216) | 69.642 | < 0.001 |
| Cerebelum 6 L - Cerebelum 6 R | -0.094 (0.899) | -2.067 (0.283) | 66.176 | < 0.001 |
| Cerebelum 6 L - Cerebelum 7b R | -0.416 (0.815) | -2.221 (0.235) | 67.295 | < 0.001 |
| Cerebelum 6 L - Cerebelum 8 R | -0.443 (0.811) | -2.387 (0.219) | 73.211 | < 0.001 |
| Cerebelum 6 R - Cerebelum 8 R | -0.159 (0.776) | -2 (0.266) | 70.963 | < 0.001 |
| Cerebelum 7b R - Vermis 7 | -0.787 (0.849) | -2.09 (0.25) | 46.554 | < 0.001 |
| Cerebelum 8 R - Vermis 6 | -0.5 (0.826) | -2.219 (0.231) | 63.402 | < 0.001 |
| Cerebelum 8 R - Vermis 7 | -0.063 (0.837) | -2.063 (0.251) | 72.326 | < 0.001 |
| Cerebelum 9 L - Vermis 3 | -0.382 (0.756) | -1.959 (0.228) | 63.153 | < 0.001 |
| Cerebelum 9 R - Vermis 3 | -0.736 (0.845) | -2.118 (0.216) | 50.146 | < 0.001 |
| Vermis 1 2 - Vermis 3 | -0.577 (0.83) | 2.274 (0.277) | -103.011 | < 0.001 |

**Supplementary Table 11**. Between-group comparison on regional frailty-related ROI-to-ROI connectivity effects between AD and FTLD. Mean (SD) t-values of the frailty–GMV association estimated separately in Alzheimer’s disease (AD) and frontotemporal lobar degeneration (FTLD) groups using repeated subsampling (n = 1000) and models adjusted for scanner effects.

|  | **AD vs CU** | **FTLD vs CU** | **AD vs FTLD** |
| --- | --- | --- | --- |
| Frailty index excluding CDR | | | |
| Accuracy | 0.77 | 0.73 | 0.6575 |
| Sensitivity (Recall) | 0.83 | 0.68 | 0.5385 |
| Specificity | 0.71 | 0.78 | 0.2632 |
| Precision | 0.74 | 0.75 | 0.7963 |
| F1 Score | 0.78 | 0.72 | 0.3125 |
| Frailty index excluding MMSE | | | |
| Accuracy | 0.77 | 0.78 | 0.59 |
| Sensitivity (Recall) | 0.76 | 0.72 | 0.45 |
| Specificity | 0.77 | 0.79 | 0.64 |
| Precision | 0.67 | 0.42 | 0.30 |
| F1 Score | 0.71 | 0.53 | 0.36 |
| Frailty index excluding NPI-Q, GAD and GDS-7 | | | |
| Accuracy | 0.75 | 0.76 | 0.57 |
| Sensitivity (Recall) | 0.76 | 0.75 | 0.41 |
| Specificity | 0.75 | 0.76 | 0.62 |
| Precision | 0.64 | 0.39 | 0.27 |
| F1 Score | 0.70 | 0.52 | 0.33 |
| Frailty index excluding Pfeffer | | | |
| Accuracy | 0.74 | 0.77 | 0.66 |
| Sensitivity (Recall) | 0.75 | 0.72 | 0.30 |
| Specificity | 0.74 | 0.78 | 0.78 |
| Precision | 0.63 | 0.40 | 0.33 |
| F1 Score | 0.69 | 0.52 | 0.32 |

**Supplementary Table 12.** Frailty model performance metrics for diagnostic classification excluding variables potentially inducing circularity.

| **Region** | **Model** | | **Frailty** | |
| --- | --- | --- | --- | --- |
|  | **R^2^_adj_** | ***P*_FDR_** | ***t*** | ***P*_FDR_** |
| Frontal Sup L | 0.220 | < 0.001 | -2.718 | 0.007 |
| Frontal Sup Orb L | 0.154 | < 0.001 | -2.659 | 0.008 |
| Frontal Sup Orb R | 0.144 | < 0.001 | -2.963 | 0.003 |
| Frontal Mid L | 0.236 | < 0.001 | -3.038 | 0.003 |
| Frontal Mid Orb R | 0.160 | < 0.001 | -2.897 | 0.004 |
| Frontal Inf Tri L | 0.141 | < 0.001 | -2.127 | 0.034 |
| Frontal Inf Orb L | 0.114 | < 0.001 | -3.014 | 0.003 |
| Frontal Inf Orb R | 0.125 | < 0.001 | -2.660 | 0.008 |
| Supp Motor Area L | 0.128 | < 0.001 | -3.098 | 0.002 |
| Frontal Sup Med L | 0.173 | < 0.001 | -3.214 | 0.001 |
| Frontal Med Orb L | 0.128 | < 0.001 | -2.247 | 0.025 |
| Rectus L | 0.105 | < 0.001 | -2.211 | 0.027 |
| Rectus R | 0.093 | < 0.001 | -2.108 | 0.036 |
| Insula L | 0.052 | < 0.001 | -3.064 | 0.002 |
| Insula R | 0.145 | < 0.001 | -4.581 | < 0.001 |
| Cingulum Mid L | 0.113 | < 0.001 | -2.404 | 0.017 |
| Hippocampus L | 0.196 | < 0.001 | -2.430 | 0.015 |
| Hippocampus R | 0.262 | < 0.001 | -2.102 | 0.036 |
| ParaHippocampal R | 0.089 | < 0.001 | -2.850 | 0.005 |
| Amygdala L | 0.140 | < 0.001 | -3.210 | 0.001 |
| Amygdala R | 0.192 | < 0.001 | -4.199 | < 0.001 |
| Calcarine L | 0.317 | < 0.001 | -2.625 | 0.009 |
| Calcarine R | 0.204 | < 0.001 | -2.458 | 0.014 |
| Cuneus L | 0.312 | < 0.001 | -2.600 | 0.010 |
| Cuneus R | 0.248 | < 0.001 | -2.611 | 0.009 |
| Lingual L | 0.183 | < 0.001 | -3.633 | < 0.001 |
| Lingual R | 0.190 | < 0.001 | -3.171 | 0.002 |
| Occipital Sup L | 0.164 | < 0.001 | -2.005 | 0.046 |
| Occipital Sup R | 0.148 | < 0.001 | -2.417 | 0.016 |
| Occipital Mid L | 0.230 | < 0.001 | -2.789 | 0.005 |
| Occipital Mid R | 0.195 | < 0.001 | -3.280 | 0.001 |
| Occipital Inf L | 0.083 | < 0.001 | -2.159 | 0.031 |
| Fusiform L | 0.130 | < 0.001 | -3.011 | 0.003 |
| Fusiform R | 0.182 | < 0.001 | -3.401 | 0.001 |
| Postcentral L | 0.191 | < 0.001 | -2.340 | 0.020 |
| Parietal Inf L | 0.165 | < 0.001 | -3.342 | 0.001 |
| Parietal Inf R | 0.187 | < 0.001 | -2.052 | 0.041 |
| SupraMarginal R | 0.138 | < 0.001 | -4.139 | < 0.001 |
| Angular R | 0.178 | < 0.001 | -2.325 | 0.020 |
| Putamen R | 0.190 | < 0.001 | -2.703 | 0.007 |
| Thalamus L | 0.267 | < 0.001 | -2.310 | 0.021 |
| Thalamus R | 0.226 | < 0.001 | -2.609 | 0.009 |
| Heschl R | 0.149 | < 0.001 | -3.043 | 0.002 |
| Temporal Sup L | 0.107 | < 0.001 | -2.077 | 0.038 |
| Temporal Sup R | 0.126 | < 0.001 | -4.246 | < 0.001 |
| Temporal Pole Sup L | 0.107 | < 0.001 | -3.230 | 0.001 |
| Temporal Pole Sup R | 0.054 | < 0.001 | -2.411 | 0.016 |
| Temporal Mid L | 0.190 | < 0.001 | -4.381 | < 0.001 |
| Temporal Mid R | 0.213 | < 0.001 | -2.878 | 0.004 |
| Temporal Pol Mid L | 0.024 | 0.014 | -2.853 | 0.005 |
| Temporal Pol Mid R | 0.072 | < 0.001 | -3.456 | 0.001 |
| Temporal Inf L | 0.130 | < 0.001 | -2.325 | 0.020 |
| Temporal Inf R | 0.261 | < 0.001 | -3.424 | 0.001 |
| Cerebellum Crus1 L | 0.073 | < 0.001 | -2.815 | 0.005 |
| Cerebellum Crus1 R | 0.080 | < 0.001 | -3.546 | < 0.001 |
| Cerebellum Crus2 R | 0.049 | < 0.001 | -2.724 | 0.007 |
| Cerebellum 3 R | 0.089 | < 0.001 | -3.210 | 0.001 |
| Cerebellum 4 5 L | 0.122 | < 0.001 | -3.218 | 0.001 |
| Cerebellum 4 5 R | 0.120 | < 0.001 | -4.354 | < 0.001 |
| Cerebellum 6 L | 0.129 | < 0.001 | -4.006 | < 0.001 |
| Cerebellum 6 R | 0.153 | < 0.001 | -4.092 | < 0.001 |
| Cerebellum 7b L | 0.040 | 0.001 | -2.209 | 0.028 |
| Cerebellum 7b R | 0.059 | < 0.001 | -3.418 | 0.001 |
| Cerebellum 8 L | 0.067 | < 0.001 | -2.193 | 0.029 |
| Cerebellum 8 R | 0.068 | < 0.001 | -2.829 | 0.005 |
| Cerebellum 10 L | 0.042 | < 0.001 | -2.179 | 0.030 |
| Cerebellum 10 R | 0.042 | 0.001 | -2.406 | 0.017 |
| Vermis 4 5 | 0.102 | < 0.001 | -2.695 | 0.007 |
| Vermis 6 | 0.065 | < 0.001 | -2.592 | 0.010 |
| Vermis 7 | 0.127 | < 0.001 | -2.423 | 0.016 |
| Vermis 9 | 0.165 | < 0.001 | -2.279 | 0.023 |

**Supplementary Table 13**. Frailty on gray matter volume for CU adjusting for scanner type and data quality metrics. *P*_FDR_<0.05. Regions are presented using the AAL atlas.

| **Region** | **Model** | | **Frailty** | |
| --- | --- | --- | --- | --- |
|  | **R^2^_adj_** | ***P*_FDR_** | ***t*** | ***P*_FDR_** |
| Precentral L | 0.203 | < 0.001 | -4.138 | < 0.001 |
| Precentral R | 0.306 | < 0.001 | -5.068 | < 0.001 |
| Frontal Sup L | 0.094 | < 0.001 | -3.712 | < 0.001 |
| Frontal Sup R | 0.142 | < 0.001 | -4.765 | < 0.001 |
| Frontal Sup Orb L | 0.168 | < 0.001 | -4.856 | < 0.001 |
| Frontal Sup Orb R | 0.122 | < 0.001 | -3.041 | 0.003 |
| Frontal Mid L | 0.158 | < 0.001 | -4.321 | < 0.001 |
| Frontal Mid R | 0.121 | < 0.001 | -3.810 | < 0.001 |
| Frontal Mid Orb L | 0.092 | < 0.001 | -3.608 | < 0.001 |
| Frontal Mid Orb R | 0.078 | < 0.001 | -3.071 | 0.002 |
| Frontal Inf Oper L | 0.093 | < 0.001 | -3.442 | 0.001 |
| Frontal Inf Oper R | 0.093 | < 0.001 | -4.490 | < 0.001 |
| Frontal Inf Tri L | 0.075 | < 0.001 | -3.697 | < 0.001 |
| Frontal Inf Tri R | 0.099 | < 0.001 | -3.823 | < 0.001 |
| Frontal Inf Orb L | 0.072 | < 0.001 | -3.021 | 0.003 |
| Frontal Inf Orb R | 0.119 | < 0.001 | -3.925 | < 0.001 |
| Rolandic Oper L | 0.130 | < 0.001 | -4.990 | < 0.001 |
| Rolandic Oper R | 0.128 | < 0.001 | -3.351 | 0.001 |
| Supp Motor Area L | 0.127 | < 0.001 | -3.455 | 0.001 |
| Supp Motor Area R | 0.148 | < 0.001 | -3.849 | < 0.001 |
| Olfactory L | 0.236 | < 0.001 | -5.134 | < 0.001 |
| Olfactory R | 0.207 | < 0.001 | -3.414 | 0.001 |
| Frontal Sup Med L | 0.161 | < 0.001 | -4.053 | < 0.001 |
| Frontal Sup Med R | 0.094 | < 0.001 | -3.238 | 0.001 |
| Frontal Med Orb L | 0.096 | < 0.001 | -3.160 | 0.002 |
| Frontal Med Orb R | 0.091 | < 0.001 | -3.243 | 0.001 |
| Rectus L | 0.209 | < 0.001 | -3.888 | < 0.001 |
| Rectus R | 0.248 | < 0.001 | -5.711 | < 0.001 |
| Insula L | 0.110 | < 0.001 | -4.502 | < 0.001 |
| Insula R | 0.194 | < 0.001 | -6.032 | < 0.001 |
| Cingulum Ant L | 0.113 | < 0.001 | -3.081 | 0.002 |
| Cingulum Mid L | 0.178 | < 0.001 | -4.245 | < 0.001 |
| Cingulum Mid R | 0.175 | < 0.001 | -5.268 | < 0.001 |
| Cingulum Post L | 0.178 | < 0.001 | -2.731 | 0.007 |
| Cingulum Post R | 0.079 | < 0.001 | -2.950 | 0.003 |
| Hippocampus L | 0.235 | < 0.001 | -5.717 | < 0.001 |
| Hippocampus R | 0.267 | < 0.001 | -6.297 | < 0.001 |
| ParaHippocampal L | 0.227 | < 0.001 | -5.103 | < 0.001 |
| ParaHippocampal R | 0.203 | < 0.001 | -5.502 | < 0.001 |
| Amygdala L | 0.165 | < 0.001 | -4.650 | < 0.001 |
| Amygdala R | 0.170 | < 0.001 | -5.909 | < 0.001 |
| Calcarine L | 0.294 | < 0.001 | -3.174 | 0.002 |
| Calcarine R | 0.174 | < 0.001 | -4.489 | < 0.001 |
| Cuneus L | 0.258 | < 0.001 | -2.885 | 0.004 |
| Cuneus R | 0.271 | < 0.001 | -4.848 | < 0.001 |
| Lingual L | 0.253 | < 0.001 | -3.950 | < 0.001 |
| Lingual R | 0.265 | < 0.001 | -4.845 | < 0.001 |
| Occipital Sup L | 0.174 | < 0.001 | -4.341 | < 0.001 |
| Occipital Sup R | 0.237 | < 0.001 | -5.972 | < 0.001 |
| Occipital Mid L | 0.198 | < 0.001 | -4.471 | < 0.001 |
| Occipital Mid R | 0.227 | < 0.001 | -5.695 | < 0.001 |
| Occipital Inf L | 0.116 | < 0.001 | -4.766 | < 0.001 |
| Occipital Inf R | 0.191 | < 0.001 | -4.901 | < 0.001 |
| Fusiform L | 0.257 | < 0.001 | -5.699 | < 0.001 |
| Fusiform R | 0.290 | < 0.001 | -5.963 | < 0.001 |
| Postcentral L | 0.256 | < 0.001 | -4.377 | < 0.001 |
| Postcentral R | 0.237 | < 0.001 | -3.444 | 0.001 |
| Parietal Sup L | 0.169 | < 0.001 | -3.305 | 0.001 |
| Parietal Sup R | 0.224 | < 0.001 | -5.333 | < 0.001 |
| Parietal Inf L | 0.174 | < 0.001 | -4.373 | < 0.001 |
| Parietal Inf R | 0.175 | < 0.001 | -3.592 | < 0.001 |
| SupraMarginal L | 0.091 | < 0.001 | -4.452 | < 0.001 |
| SupraMarginal R | 0.150 | < 0.001 | -4.800 | < 0.001 |
| Angular L | 0.118 | < 0.001 | -4.450 | < 0.001 |
| Angular R | 0.161 | < 0.001 | -4.777 | < 0.001 |
| Precuneus L | 0.214 | < 0.001 | -3.741 | < 0.001 |
| Precuneus R | 0.218 | < 0.001 | -4.057 | < 0.001 |
| Caudate L | 0.131 | < 0.001 | -2.534 | 0.012 |
| Putamen L | 0.114 | < 0.001 | -2.865 | 0.004 |
| Putamen R | 0.097 | < 0.001 | -2.402 | 0.017 |
| Thalamus L | 0.225 | < 0.001 | -3.713 | < 0.001 |
| Thalamus R | 0.277 | < 0.001 | -4.292 | < 0.001 |
| Heschl L | 0.181 | < 0.001 | -4.930 | < 0.001 |
| Heschl R | 0.170 | < 0.001 | -3.299 | 0.001 |
| Temporal Sup L | 0.154 | < 0.001 | -5.937 | < 0.001 |
| Temporal Sup R | 0.167 | < 0.001 | -5.512 | < 0.001 |
| Temporal Pole Sup L | 0.156 | < 0.001 | -3.699 | < 0.001 |
| Temporal Pole Sup R | 0.113 | < 0.001 | -3.462 | 0.001 |
| Temporal Mid L | 0.176 | < 0.001 | -5.737 | < 0.001 |
| Temporal Mid R | 0.183 | < 0.001 | -5.345 | < 0.001 |
| Temporal Pol Mid L | 0.125 | < 0.001 | -4.063 | < 0.001 |
| Temporal Pol Mid R | 0.155 | < 0.001 | -4.150 | < 0.001 |
| Temporal Inf L | 0.241 | < 0.001 | -5.383 | < 0.001 |
| Temporal Inf R | 0.266 | < 0.001 | -6.483 | < 0.001 |
| Cerebellum Crus2 L | 0.061 | < 0.001 | -3.525 | < 0.001 |
| Cerebellum Crus2 R | 0.058 | < 0.001 | -2.798 | 0.005 |
| Cerebellum 6 L | 0.133 | < 0.001 | -2.126 | 0.034 |
| Cerebellum 6 R | 0.124 | < 0.001 | -2.159 | 0.031 |
| Cerebellum 7b L | 0.053 | 0.001 | -3.509 | 0.001 |
| Cerebellum 7b R | 0.080 | < 0.001 | -3.012 | 0.003 |
| Cerebellum 8 L | 0.097 | < 0.001 | -2.421 | 0.016 |
| Cerebellum 8 R | 0.110 | < 0.001 | -2.712 | 0.007 |

**Supplementary Table 14**. Frailty on gray matter volume for AD adjusting for scanner type and data quality metrics. *P*_FDR_<0.05. Regions are presented using the AAL atlas.

| **Region** | **Model** | | | **Frailty** | | |
| --- | --- | --- | --- | --- | --- | --- |
|  | **R^2^_adj_** | ***P*_FDR_** | ***t*** | | ***P*_FDR_** |  |
| Frontal Sup L | 0.189 | < 0.001 | -4.290 | | < 0.001 |  |
| Frontal Sup R | 0.068 | 0.029 | -3.005 | | 0.003 |  |
| Frontal Sup Orb L | 0.163 | < 0.001 | -3.136 | | 0.002 |  |
| Frontal Mid L | 0.171 | < 0.001 | -4.407 | | < 0.001 |  |
| Frontal Mid R | 0.089 | 0.010 | -2.549 | | 0.012 |  |
| Frontal Mid Orb L | 0.124 | 0.002 | -3.092 | | 0.002 |  |
| Frontal Inf Oper L | 0.096 | 0.007 | -3.767 | | < 0.001 |  |
| Frontal Inf Tri L | 0.107 | 0.004 | -3.880 | | < 0.001 |  |
| Frontal Inf Orb L | 0.135 | 0.001 | -4.227 | | < 0.001 |  |
| Rolandic Oper L | 0.163 | < 0.001 | -3.206 | | 0.002 |  |
| Rolandic Oper R | 0.081 | 0.015 | -2.982 | | 0.003 |  |
| Frontal Sup Med L | 0.161 | < 0.001 | -3.901 | | < 0.001 |  |
| Frontal Med Orb L | 0.113 | 0.003 | -2.102 | | 0.037 |  |
| Rectus L | 0.075 | 0.021 | -2.395 | | 0.018 |  |
| Insula L | 0.062 | 0.038 | -2.260 | | 0.025 |  |
| Insula R | 0.074 | 0.021 | -2.834 | | 0.005 |  |
| Cingulum Ant L | 0.089 | 0.010 | -2.775 | | 0.006 |  |
| Cingulum Mid L | 0.094 | 0.008 | -3.365 | | 0.001 |  |
| Cingulum Mid R | 0.083 | 0.014 | -2.494 | | 0.014 |  |
| Cingulum Post L | 0.152 | < 0.001 | -2.931 | | 0.004 |  |
| Occipital Mid R | 0.168 | < 0.001 | -2.264 | | 0.025 |  |
| Occipital Inf R | 0.192 | < 0.001 | -2.534 | | 0.012 |  |
| Parietal Sup R | 0.064 | 0.035 | -2.125 | | 0.035 |  |
| SupraMarginal L | 0.100 | 0.006 | -3.602 | | < 0.001 |  |
| Precuneus R | 0.070 | 0.026 | -2.300 | | 0.023 |  |
| Caudate L | 0.143 | 0.001 | -3.170 | | 0.002 |  |
| Putamen L | 0.122 | 0.002 | -2.006 | | 0.047 |  |
| Heschl L | 0.169 | < 0.001 | -2.394 | | 0.018 |  |
| Temporal Sup L | 0.162 | < 0.001 | -2.817 | | 0.005 |  |
| Temporal Sup R | 0.092 | 0.009 | -2.074 | | 0.040 |  |
| Temporal Mid L | 0.132 | 0.001 | -2.463 | | 0.015 |  |

**Supplementary Table 15**. Frailty on gray matter volume for FTLD adjusting for scanner type and data quality metrics. *P*_FDR_<0.05. Regions are presented using the AAL atlas.

| **Regions** | **Model** | | **Frailty** | |
| --- | --- | --- | --- | --- |
|  | **R^2^_adj_** | ***P*_FDR_** | ***t*** | ***P*_FDR_** |
| Precentral L - Rolandic Oper L | 0.154 | < 0.001 | -1.974 | 0.049 |
| Precentral L - Cingulum Mid R | 0.162 | < 0.001 | 2.052 | 0.041 |
| Precentral L - Lingual L | 0.179 | < 0.001 | -2.787 | 0.006 |
| Precentral L - Lingual R | 0.200 | < 0.001 | -2.180 | 0.030 |
| Precentral L - Fusiform L | 0.245 | < 0.001 | -2.706 | 0.007 |
| Precentral L - Temporal Pole Mid R | 0.234 | < 0.001 | -2.209 | 0.028 |
| Precentral L - Temporal Inf L | 0.294 | < 0.001 | -2.163 | 0.031 |
| Precentral L - Cerebellum 3 R | 0.063 | 0.004 | -2.047 | 0.042 |
| Precentral R - Occipital Inf R | 0.136 | < 0.001 | -2.070 | 0.040 |
| Precentral R - Parietal Sup R | 0.150 | < 0.001 | 2.191 | 0.029 |
| Precentral R - Cerebellum Crus2 L | 0.064 | 0.004 | 2.384 | 0.018 |
| Precentral R - Cerebellum Crus2 R | 0.074 | 0.002 | 2.410 | 0.017 |
| Frontal Sup L - Frontal Sup R | 0.389 | < 0.001 | 1.997 | 0.047 |
| Frontal Sup L - Frontal Sup Orb L | 0.168 | < 0.001 | -2.051 | 0.041 |
| Frontal Sup L - Temporal Inf R | 0.251 | < 0.001 | -2.392 | 0.018 |
| Frontal Sup L - Cerebellum 7b R | 0.076 | 0.001 | -2.132 | 0.034 |
| Frontal Sup R - Frontal Sup Orb L | 0.157 | < 0.001 | -2.712 | 0.007 |
| Frontal Sup R - Olfactory R | 0.126 | < 0.001 | -2.333 | 0.020 |
| Frontal Sup R - Temporal Inf R | 0.306 | < 0.001 | -2.754 | 0.006 |
| Frontal Sup R - Cerebellum 6 R | 0.086 | 0.001 | -2.012 | 0.045 |
| Frontal Sup R - Cerebellum 7b R | 0.100 | < 0.001 | -2.009 | 0.046 |
| Frontal Sup Orb L - Frontal Sup Medial L | 0.120 | < 0.001 | -2.170 | 0.031 |
| Frontal Sup Orb L - Frontal Med Orb L | 0.274 | < 0.001 | -1.974 | 0.050 |
| Frontal Sup Orb L - Cingulum Ant L | 0.172 | < 0.001 | -3.183 | 0.002 |
| Frontal Sup Orb L - Cingulum Ant R | 0.209 | < 0.001 | -2.282 | 0.023 |
| Frontal Sup Orb L - Amygdala L | 0.130 | < 0.001 | -2.225 | 0.027 |
| Frontal Sup Orb L - Cuneus L | 0.070 | 0.002 | -2.634 | 0.009 |
| Frontal Sup Orb L - Angular L | 0.119 | < 0.001 | -2.202 | 0.029 |
| Frontal Sup Orb L - Cerebellum 3 L | 0.082 | 0.001 | 3.771 | < 0.001 |
| Frontal Sup Orb L - Vermis 3 | 0.054 | 0.008 | 1.975 | 0.049 |
| Frontal Sup Orb R - Cingulum Ant L | 0.110 | < 0.001 | -2.736 | 0.007 |
| Frontal Sup Orb R - Cingulum Ant R | 0.178 | < 0.001 | -2.259 | 0.025 |
| Frontal Sup Orb R - Cerebellum 3 L | 0.058 | 0.006 | 2.482 | 0.014 |
| Frontal Mid L - Precuneus L | 0.184 | < 0.001 | -2.111 | 0.036 |
| Frontal Mid L - Temporal Mid R | 0.079 | 0.001 | -2.038 | 0.043 |
| Frontal Mid L - Temporal Inf L | 0.322 | < 0.001 | -2.187 | 0.030 |
| Frontal Mid L - Temporal Inf R | 0.264 | < 0.001 | -2.262 | 0.025 |
| Frontal Mid R - Lingual L | 0.049 | 0.013 | -2.075 | 0.039 |
| Frontal Mid R - Fusiform L | 0.119 | < 0.001 | -2.599 | 0.010 |
| Frontal Mid R - Temporal Inf R | 0.304 | < 0.001 | -2.695 | 0.008 |
| Frontal Mid R - Cerebellum 6 R | 0.088 | < 0.001 | -2.145 | 0.033 |
| Frontal Mid Orb L - Cerebellum 3 L | 0.077 | 0.001 | 2.930 | 0.004 |
| Frontal Mid Orb L - Vermis 3 | 0.101 | < 0.001 | 2.407 | 0.017 |
| Frontal Mid Orb L - Vermis 4 5 | 0.098 | < 0.001 | 1.988 | 0.048 |
| Frontal Mid Orb R - Occipital Inf L | 0.085 | 0.001 | 2.307 | 0.022 |
| Frontal Mid Orb R - Cerebellum 3 L | 0.042 | 0.023 | 2.015 | 0.045 |
| Frontal Inf Oper L - Pallidum L | 0.126 | < 0.001 | -3.453 | 0.001 |
| Frontal Inf Oper L - Temporal Inf L | 0.122 | < 0.001 | -2.572 | 0.011 |
| Frontal Inf Oper R - Temporal Inf R | 0.179 | < 0.001 | -2.085 | 0.038 |
| Frontal Inf Oper R - Cerebellum Crus1 R | 0.093 | < 0.001 | 2.206 | 0.028 |
| Frontal Inf Oper R - Cerebellum Crus2 R | 0.059 | 0.005 | 2.610 | 0.010 |
| Frontal Inf Tri L - Frontal Inf Orb L | 0.273 | < 0.001 | -2.416 | 0.016 |
| Frontal Inf Tri L - Occipital Inf L | 0.092 | < 0.001 | -2.274 | 0.024 |
| Frontal Inf Tri L - Occipital Inf R | 0.058 | 0.006 | -2.240 | 0.026 |
| Frontal Inf Tri L - Fusiform L | 0.057 | 0.007 | -2.262 | 0.025 |
| Frontal Inf Tri L - Fusiform R | 0.108 | < 0.001 | -2.588 | 0.010 |
| Frontal Inf Tri L - Precuneus R | 0.036 | 0.038 | -2.176 | 0.031 |
| Frontal Inf Tri L - Temporal Mid L | 0.200 | < 0.001 | -1.977 | 0.049 |
| Frontal Inf Tri L - Temporal Inf L | 0.129 | < 0.001 | -3.616 | < 0.001 |
| Frontal Inf Tri L - Temporal Inf R | 0.160 | < 0.001 | -2.829 | 0.005 |
| Frontal Inf Tri L - Cerebellum 6 R | 0.048 | 0.014 | -2.173 | 0.031 |
| Frontal Inf Tri R - Lingual R | 0.060 | 0.005 | -2.016 | 0.045 |
| Frontal Inf Tri R - Fusiform L | 0.049 | 0.013 | -2.168 | 0.031 |
| Frontal Inf Tri R - Temporal Inf R | 0.160 | < 0.001 | -2.898 | 0.004 |
| Frontal Inf Tri R - Cerebellum 6 L | 0.111 | < 0.001 | -2.083 | 0.038 |
| Frontal Inf Tri R - Cerebellum 6 R | 0.065 | 0.003 | -2.047 | 0.042 |
| Frontal Inf Orb L - Supp Motor Area R | 0.107 | < 0.001 | -2.483 | 0.014 |
| Frontal Inf Orb L - Pallidum L | 0.168 | < 0.001 | 2.497 | 0.013 |
| Frontal Inf Orb L - Heschl L | 0.075 | 0.001 | 2.248 | 0.025 |
| Frontal Inf Orb L - Heschl R | 0.157 | < 0.001 | 2.109 | 0.036 |
| Frontal Inf Orb R - Frontal Sup Medial R | 0.140 | < 0.001 | -2.385 | 0.018 |
| Frontal Inf Orb R - Fusiform R | 0.173 | < 0.001 | 2.435 | 0.016 |
| Frontal Inf Orb R - Heschl L | 0.079 | 0.001 | 2.531 | 0.012 |
| Frontal Inf Orb R - Heschl R | 0.135 | < 0.001 | 2.204 | 0.028 |
| Frontal Inf Orb R - Cerebellum 3 L | 0.044 | 0.019 | 1.972 | 0.050 |
| Frontal Inf Orb R - Cerebellum 4 5 R | 0.124 | < 0.001 | 2.167 | 0.031 |
| Frontal Inf Orb R - Vermis 3 | 0.066 | 0.003 | 2.791 | 0.006 |
| Rolandic Oper L - Temporal Pole Mid L | 0.103 | < 0.001 | -2.286 | 0.023 |
| Rolandic Oper L - Cerebellum 8 L | 0.109 | < 0.001 | 2.376 | 0.018 |
| Rolandic Oper L - Cerebellum 9 R | 0.040 | 0.028 | 2.163 | 0.031 |
| Rolandic Oper R - Parietal Inf R | 0.182 | < 0.001 | 2.669 | 0.008 |
| Rolandic Oper R - Angular L | 0.109 | < 0.001 | 2.183 | 0.030 |
| Rolandic Oper R - Angular R | 0.089 | < 0.001 | 4.090 | < 0.001 |
| Rolandic Oper R - Thalamus R | 0.142 | < 0.001 | 2.011 | 0.045 |
| Supp Motor Area L - Postcentral L | 0.274 | < 0.001 | -2.302 | 0.022 |
| Supp Motor Area L - Pallidum L | 0.051 | 0.011 | -2.287 | 0.023 |
| Supp Motor Area L - Temporal Pole Sup L | 0.148 | < 0.001 | -2.001 | 0.047 |
| Supp Motor Area L - Cerebellum 3 L | 0.061 | 0.005 | -2.528 | 0.012 |
| Supp Motor Area L - Cerebellum 3 R | 0.094 | < 0.001 | -2.487 | 0.014 |
| Supp Motor Area L - Cerebellum 4 5 L | 0.093 | < 0.001 | -2.272 | 0.024 |
| Supp Motor Area L - Cerebellum 6 R | 0.133 | < 0.001 | -2.492 | 0.013 |
| Supp Motor Area L - Vermis 3 | 0.071 | 0.002 | -2.994 | 0.003 |
| Supp Motor Area L - Vermis 6 | 0.113 | < 0.001 | -2.720 | 0.007 |
| Supp Motor Area R - Cingulum Ant L | 0.086 | 0.001 | -2.256 | 0.025 |
| Supp Motor Area R - Lingual L | 0.196 | < 0.001 | -2.218 | 0.027 |
| Supp Motor Area R - Lingual R | 0.183 | < 0.001 | -2.122 | 0.035 |
| Supp Motor Area R - Fusiform L | 0.258 | < 0.001 | -2.027 | 0.044 |
| Supp Motor Area R - Fusiform R | 0.249 | < 0.001 | -3.086 | 0.002 |
| Supp Motor Area R - Temporal Pole Sup L | 0.169 | < 0.001 | -2.095 | 0.037 |
| Supp Motor Area R - Temporal Inf R | 0.263 | < 0.001 | -2.376 | 0.018 |
| Supp Motor Area R - Cerebellum 10 R | 0.094 | < 0.001 | -2.281 | 0.023 |
| Supp Motor Area R - Vermis 3 | 0.113 | < 0.001 | -2.936 | 0.004 |
| Olfactory L - Parietal Inf R | 0.149 | < 0.001 | 2.500 | 0.013 |
| Olfactory L - Caudate R | 0.097 | < 0.001 | 2.516 | 0.013 |
| Olfactory L - Cerebellum 7b L | 0.067 | 0.003 | 2.227 | 0.027 |
| Olfactory R - Frontal Sup Medial R | 0.111 | < 0.001 | -2.577 | 0.011 |
| Olfactory R - Cingulum Ant L | 0.121 | < 0.001 | -2.442 | 0.015 |
| Olfactory R - Cingulum Mid L | 0.084 | 0.001 | -2.001 | 0.046 |
| Olfactory R - Parietal Inf L | 0.050 | 0.012 | -2.116 | 0.035 |
| Olfactory R - Cerebellum 10 R | 0.092 | < 0.001 | -2.211 | 0.028 |
| Frontal Sup Medial L - Rectus L | 0.176 | < 0.001 | -2.827 | 0.005 |
| Frontal Sup Medial L - Rectus R | 0.172 | < 0.001 | -3.171 | 0.002 |
| Frontal Sup Medial L - Calcarine L | 0.164 | < 0.001 | -2.000 | 0.047 |
| Frontal Sup Medial L - Occipital Inf R | 0.040 | 0.028 | -2.071 | 0.039 |
| Frontal Sup Medial L - Precuneus L | 0.174 | < 0.001 | -2.533 | 0.012 |
| Frontal Sup Medial L - Precuneus R | 0.073 | 0.002 | -2.631 | 0.009 |
| Frontal Sup Medial L - Temporal Mid L | 0.175 | < 0.001 | -2.354 | 0.019 |
| Frontal Sup Medial L - Temporal Mid R | 0.117 | < 0.001 | -2.723 | 0.007 |
| Frontal Sup Medial L - Temporal Inf L | 0.188 | < 0.001 | -2.925 | 0.004 |
| Frontal Sup Medial L - Temporal Inf R | 0.175 | < 0.001 | -2.627 | 0.009 |
| Frontal Sup Medial L - Cerebellum Crus2 R | 0.152 | < 0.001 | -2.768 | 0.006 |
| Frontal Sup Medial R - Rectus R | 0.221 | < 0.001 | -2.245 | 0.026 |
| Frontal Sup Medial R - Amygdala L | 0.142 | < 0.001 | -2.342 | 0.020 |
| Frontal Sup Medial R - Cerebellum 7b L | 0.092 | < 0.001 | 2.159 | 0.032 |
| Frontal Med Orb L - ParaHippocampal L | 0.113 | < 0.001 | -2.406 | 0.017 |
| Frontal Med Orb L - Amygdala L | 0.068 | 0.002 | -2.439 | 0.015 |
| Frontal Med Orb R - Cingulum Ant L | 0.274 | < 0.001 | -2.614 | 0.009 |
| Frontal Med Orb R - ParaHippocampal L | 0.097 | < 0.001 | -2.022 | 0.044 |
| Frontal Med Orb R - Amygdala L | 0.088 | < 0.001 | -2.293 | 0.023 |
| Frontal Med Orb R - Parietal Inf R | 0.043 | 0.022 | 2.198 | 0.029 |
| Rectus L - Cingulum Ant L | 0.170 | < 0.001 | -2.107 | 0.036 |
| Rectus L - Amygdala L | 0.200 | < 0.001 | -2.291 | 0.023 |
| Rectus L - Cuneus L | 0.080 | 0.001 | -2.514 | 0.013 |
| Rectus L - Occipital Sup L | 0.053 | 0.010 | -2.307 | 0.022 |
| Rectus L - Occipital Sup R | 0.068 | 0.003 | -2.099 | 0.037 |
| Rectus L - Parietal Inf R | 0.192 | < 0.001 | 2.062 | 0.040 |
| Rectus L - Temporal Sup R | 0.124 | < 0.001 | -2.712 | 0.007 |
| Rectus L - Cerebellum 3 L | 0.035 | 0.042 | 1.996 | 0.047 |
| Rectus L - Cerebellum 4 5 L | 0.058 | 0.006 | 2.309 | 0.022 |
| Rectus L - Cerebellum 4 5 R | 0.073 | 0.002 | 2.871 | 0.004 |
| Rectus L - Vermis 4 5 | 0.104 | < 0.001 | 2.497 | 0.013 |
| Rectus R - Cingulum Ant L | 0.185 | < 0.001 | -2.304 | 0.022 |
| Rectus R - Cingulum Ant R | 0.279 | < 0.001 | -3.008 | 0.003 |
| Rectus R - Amygdala L | 0.216 | < 0.001 | -3.281 | 0.001 |
| Rectus R - Occipital Inf L | 0.161 | < 0.001 | 3.042 | 0.003 |
| Rectus R - Temporal Sup R | 0.091 | < 0.001 | -2.333 | 0.020 |
| Rectus R - Cerebellum 3 R | 0.059 | 0.005 | 2.194 | 0.029 |
| Rectus R - Cerebellum 4 5 L | 0.048 | 0.014 | 2.434 | 0.016 |
| Rectus R - Cerebellum 4 5 R | 0.046 | 0.016 | 2.586 | 0.010 |
| Rectus R - Cerebellum 6 L | 0.051 | 0.011 | 2.353 | 0.019 |
| Rectus R - Cerebellum 6 R | 0.080 | 0.001 | 2.007 | 0.046 |
| Rectus R - Vermis 4 5 | 0.112 | < 0.001 | 2.268 | 0.024 |
| Insula R - Calcarine L | 0.074 | 0.001 | -2.073 | 0.039 |
| Insula R - Pallidum R | 0.122 | < 0.001 | 2.353 | 0.019 |
| Insula R - Thalamus R | 0.114 | < 0.001 | 2.154 | 0.032 |
| Cingulum Ant L - ParaHippocampal L | 0.045 | 0.018 | -2.722 | 0.007 |
| Cingulum Ant L - Amygdala L | 0.092 | < 0.001 | -2.185 | 0.030 |
| Cingulum Ant L - Temporal Sup L | 0.135 | < 0.001 | -2.025 | 0.044 |
| Cingulum Ant L - Temporal Pole Sup R | 0.092 | < 0.001 | -2.127 | 0.034 |
| Cingulum Ant L - Cerebellum 3 R | 0.050 | 0.012 | -2.067 | 0.040 |
| Cingulum Ant L - Cerebellum 4 5 L | 0.033 | 0.049 | -1.972 | 0.050 |
| Cingulum Ant L - Vermis 6 | 0.060 | 0.005 | -2.309 | 0.022 |
| Cingulum Ant R - ParaHippocampal L | 0.068 | 0.002 | -2.215 | 0.028 |
| Cingulum Ant R - Amygdala L | 0.126 | < 0.001 | -3.374 | 0.001 |
| Cingulum Ant R - Temporal Pole Sup L | 0.079 | 0.001 | -2.063 | 0.040 |
| Cingulum Ant R - Temporal Pole Mid R | 0.093 | < 0.001 | -2.583 | 0.010 |
| Cingulum Mid L - Cerebellum 4 5 L | 0.089 | < 0.001 | -2.005 | 0.046 |
| Cingulum Mid L - Vermis 3 | 0.074 | 0.001 | -2.219 | 0.027 |
| Cingulum Mid L - Vermis 6 | 0.046 | 0.017 | -2.385 | 0.018 |
| Cingulum Mid L - Vermis 7 | 0.108 | < 0.001 | -2.215 | 0.028 |
| Cingulum Mid R - Fusiform L | 0.190 | < 0.001 | -2.352 | 0.019 |
| Cingulum Mid R - Fusiform R | 0.214 | < 0.001 | -2.414 | 0.017 |
| Cingulum Mid R - Cerebellum Crus2 R | 0.151 | < 0.001 | 2.283 | 0.023 |
| Cingulum Mid R - Cerebellum 10 R | 0.090 | < 0.001 | -2.057 | 0.041 |
| Cingulum Mid R - Vermis 3 | 0.095 | < 0.001 | -2.304 | 0.022 |
| Cingulum Mid R - Vermis 6 | 0.057 | 0.007 | -2.267 | 0.024 |
| Cingulum Mid R - Vermis 7 | 0.115 | < 0.001 | -2.208 | 0.028 |
| Cingulum Post L - Hippocampus L | 0.151 | < 0.001 | 2.129 | 0.034 |
| Cingulum Post L - Vermis 4 5 | 0.177 | < 0.001 | 2.219 | 0.027 |
| Cingulum Post R - ParaHippocampal R | 0.081 | 0.001 | 2.191 | 0.029 |
| Cingulum Post R - Heschl R | 0.113 | < 0.001 | 2.365 | 0.019 |
| Hippocampus L - Postcentral L | 0.089 | < 0.001 | -2.572 | 0.011 |
| Hippocampus L - Angular L | 0.150 | < 0.001 | 2.119 | 0.035 |
| Hippocampus L - Cerebellum 4 5 L | 0.130 | < 0.001 | 2.601 | 0.010 |
| Hippocampus L - Cerebellum 6 R | 0.074 | 0.001 | 1.992 | 0.048 |
| Hippocampus R - Temporal Mid L | 0.195 | < 0.001 | 2.595 | 0.010 |
| Hippocampus R - Temporal Mid R | 0.220 | < 0.001 | 2.522 | 0.012 |
| ParaHippocampal L - ParaHippocampal R | 0.360 | < 0.001 | 2.352 | 0.019 |
| ParaHippocampal L - Fusiform R | 0.220 | < 0.001 | 2.889 | 0.004 |
| ParaHippocampal L - Parietal Inf R | 0.075 | 0.001 | 2.352 | 0.019 |
| ParaHippocampal L - Temporal Pole Mid R | 0.157 | < 0.001 | 2.140 | 0.033 |
| ParaHippocampal L - Temporal Inf R | 0.177 | < 0.001 | 2.206 | 0.028 |
| ParaHippocampal L - Cerebellum Crus2 R | 0.046 | 0.016 | 2.333 | 0.020 |
| ParaHippocampal L - Cerebellum 6 R | 0.124 | < 0.001 | 2.485 | 0.014 |
| ParaHippocampal R - Fusiform L | 0.325 | < 0.001 | 3.213 | 0.001 |
| ParaHippocampal R - Fusiform R | 0.302 | < 0.001 | 3.015 | 0.003 |
| ParaHippocampal R - Thalamus R | 0.115 | < 0.001 | 2.057 | 0.041 |
| ParaHippocampal R - Temporal Mid L | 0.085 | 0.001 | 2.398 | 0.017 |
| ParaHippocampal R - Temporal Mid R | 0.169 | < 0.001 | 2.344 | 0.020 |
| ParaHippocampal R - Temporal Pole Mid L | 0.249 | < 0.001 | 2.314 | 0.022 |
| ParaHippocampal R - Temporal Inf L | 0.314 | < 0.001 | 3.238 | 0.001 |
| ParaHippocampal R - Temporal Inf R | 0.274 | < 0.001 | 2.964 | 0.003 |
| ParaHippocampal R - Cerebellum 3 L | 0.155 | < 0.001 | 2.151 | 0.032 |
| ParaHippocampal R - Cerebellum 4 5 L | 0.117 | < 0.001 | 2.303 | 0.022 |
| ParaHippocampal R - Cerebellum 4 5 R | 0.148 | < 0.001 | 2.706 | 0.007 |
| ParaHippocampal R - Cerebellum 6 R | 0.096 | < 0.001 | 2.139 | 0.033 |
| Amygdala L - Temporal Pole Sup L | 0.225 | < 0.001 | -2.078 | 0.039 |
| Amygdala L - Vermis 6 | 0.040 | 0.027 | -2.131 | 0.034 |
| Amygdala L - Vermis 10 | 0.100 | < 0.001 | 1.987 | 0.048 |
| Amygdala R - Cerebellum 7b R | 0.096 | < 0.001 | -2.021 | 0.044 |
| Calcarine L - Heschl R | 0.108 | < 0.001 | 2.399 | 0.017 |
| Calcarine R - Pallidum L | 0.129 | < 0.001 | 2.799 | 0.006 |
| Calcarine R - Thalamus R | 0.137 | < 0.001 | 2.240 | 0.026 |
| Calcarine R - Heschl R | 0.155 | < 0.001 | 2.859 | 0.005 |
| Calcarine R - Vermis 4 5 | 0.234 | < 0.001 | 2.596 | 0.010 |
| Cuneus L - Occipital Inf R | 0.101 | < 0.001 | -2.831 | 0.005 |
| Cuneus L - Fusiform L | 0.140 | < 0.001 | -2.434 | 0.016 |
| Cuneus L - Fusiform R | 0.182 | < 0.001 | -2.202 | 0.029 |
| Cuneus L - Temporal Inf L | 0.112 | < 0.001 | -2.698 | 0.007 |
| Cuneus L - Temporal Inf R | 0.123 | < 0.001 | -2.505 | 0.013 |
| Cuneus L - Cerebellum 3 L | 0.042 | 0.023 | -2.151 | 0.032 |
| Cuneus L - Vermis 7 | 0.131 | < 0.001 | -2.673 | 0.008 |
| Cuneus R - Fusiform L | 0.198 | < 0.001 | -2.355 | 0.019 |
| Cuneus R - Pallidum R | 0.101 | < 0.001 | 2.203 | 0.029 |
| Cuneus R - Thalamus R | 0.187 | < 0.001 | 2.929 | 0.004 |
| Cuneus R - Temporal Inf L | 0.098 | < 0.001 | -2.152 | 0.032 |
| Cuneus R - Temporal Inf R | 0.113 | < 0.001 | -2.378 | 0.018 |
| Lingual L - Paracentral Lobule L | 0.233 | < 0.001 | -2.036 | 0.043 |
| Lingual L - Heschl R | 0.057 | 0.007 | 2.177 | 0.030 |
| Lingual L - Cerebellum 4 5 L | 0.198 | < 0.001 | 2.541 | 0.012 |
| Lingual L - Cerebellum 4 5 R | 0.208 | < 0.001 | 2.241 | 0.026 |
| Lingual L - Vermis 8 | 0.107 | < 0.001 | 2.290 | 0.023 |
| Lingual R - Paracentral Lobule L | 0.256 | < 0.001 | -2.235 | 0.026 |
| Lingual R - Paracentral Lobule R | 0.242 | < 0.001 | -2.048 | 0.042 |
| Lingual R - Heschl R | 0.059 | 0.006 | 2.636 | 0.009 |
| Lingual R - Vermis 8 | 0.106 | < 0.001 | 2.368 | 0.019 |
| Occipital Sup L - Fusiform R | 0.251 | < 0.001 | -2.037 | 0.043 |
| Occipital Sup L - Angular R | 0.102 | < 0.001 | 2.226 | 0.027 |
| Occipital Sup L - Pallidum R | 0.135 | < 0.001 | 1.981 | 0.049 |
| Occipital Sup L - Thalamus R | 0.111 | < 0.001 | 2.386 | 0.018 |
| Occipital Sup L - Temporal Inf R | 0.098 | < 0.001 | -2.375 | 0.018 |
| Occipital Sup R - Pallidum R | 0.160 | < 0.001 | 2.176 | 0.030 |
| Occipital Sup R - Thalamus R | 0.150 | < 0.001 | 2.317 | 0.021 |
| Occipital Mid L - Caudate L | 0.074 | 0.002 | 2.239 | 0.026 |
| Occipital Mid L - Pallidum L | 0.064 | 0.003 | 2.200 | 0.029 |
| Occipital Mid L - Thalamus R | 0.126 | < 0.001 | 2.196 | 0.029 |
| Occipital Mid L - Heschl R | 0.064 | 0.004 | 2.089 | 0.038 |
| Occipital Mid L - Temporal Inf R | 0.192 | < 0.001 | -2.441 | 0.015 |
| Occipital Mid L - Vermis 4 5 | 0.181 | < 0.001 | 2.061 | 0.040 |
| Occipital Mid R - Thalamus R | 0.163 | < 0.001 | 2.309 | 0.022 |
| Occipital Mid R - Heschl R | 0.074 | 0.001 | 2.033 | 0.043 |
| Occipital Mid R - Temporal Inf R | 0.120 | < 0.001 | -2.123 | 0.035 |
| Occipital Inf L - Cerebellum Crus2 R | 0.135 | < 0.001 | 2.219 | 0.027 |
| Occipital Inf L - Cerebellum 4 5 L | 0.193 | < 0.001 | 3.139 | 0.002 |
| Occipital Inf L - Cerebellum 4 5 R | 0.112 | < 0.001 | 2.151 | 0.032 |
| Occipital Inf L - Vermis 4 5 | 0.134 | < 0.001 | 2.016 | 0.045 |
| Occipital Inf L - Vermis 6 | 0.098 | < 0.001 | 2.555 | 0.011 |
| Occipital Inf L - Vermis 8 | 0.121 | < 0.001 | 2.365 | 0.019 |
| Occipital Inf R - Postcentral L | 0.145 | < 0.001 | -2.119 | 0.035 |
| Occipital Inf R - Paracentral Lobule R | 0.176 | < 0.001 | -2.344 | 0.020 |
| Fusiform L - Paracentral Lobule L | 0.199 | < 0.001 | -2.365 | 0.019 |
| Fusiform L - Cerebellum Crus2 L | 0.235 | < 0.001 | 2.241 | 0.026 |
| Fusiform L - Cerebellum Crus2 R | 0.167 | < 0.001 | 2.537 | 0.012 |
| Fusiform L - Cerebellum 4 5 L | 0.269 | < 0.001 | 2.138 | 0.034 |
| Fusiform L - Cerebellum 4 5 R | 0.235 | < 0.001 | 2.812 | 0.005 |
| Fusiform L - Cerebellum 6 L | 0.323 | < 0.001 | 2.205 | 0.028 |
| Fusiform L - Cerebellum 6 R | 0.277 | < 0.001 | 1.997 | 0.047 |
| Fusiform L - Vermis 4 5 | 0.237 | < 0.001 | 2.739 | 0.007 |
| Fusiform L - Vermis 6 | 0.177 | < 0.001 | 2.862 | 0.005 |
| Fusiform L - Vermis 8 | 0.148 | < 0.001 | 2.403 | 0.017 |
| Fusiform R - Paracentral Lobule L | 0.233 | < 0.001 | -2.465 | 0.014 |
| Fusiform R - Paracentral Lobule R | 0.284 | < 0.001 | -2.042 | 0.042 |
| Fusiform R - Temporal Pole Sup R | 0.175 | < 0.001 | 2.290 | 0.023 |
| Fusiform R - Cerebellum Crus2 R | 0.285 | < 0.001 | 2.157 | 0.032 |
| Fusiform R - Cerebellum 4 5 R | 0.274 | < 0.001 | 2.979 | 0.003 |
| Fusiform R - Vermis 6 | 0.197 | < 0.001 | 2.161 | 0.032 |
| Fusiform R - Vermis 8 | 0.160 | < 0.001 | 2.502 | 0.013 |
| Postcentral L - Pallidum L | 0.076 | 0.001 | -2.247 | 0.026 |
| Postcentral L - Temporal Pole Mid L | 0.145 | < 0.001 | -2.086 | 0.038 |
| Postcentral L - Cerebellum Crus2 R | 0.087 | < 0.001 | 2.024 | 0.044 |
| Postcentral L - Cerebellum 9 L | 0.080 | 0.001 | 2.115 | 0.035 |
| Postcentral R - Parietal Sup R | 0.222 | < 0.001 | 2.149 | 0.033 |
| Parietal Sup L - Caudate L | 0.130 | < 0.001 | 3.594 | < 0.001 |
| Parietal Sup L - Caudate R | 0.113 | < 0.001 | 2.868 | 0.004 |
| Parietal Sup L - Heschl R | 0.097 | < 0.001 | 2.999 | 0.003 |
| Parietal Sup L - Temporal Sup L | 0.096 | < 0.001 | 2.085 | 0.038 |
| Parietal Sup R - Caudate L | 0.129 | < 0.001 | 2.883 | 0.004 |
| Parietal Sup R - Caudate R | 0.084 | 0.001 | 2.340 | 0.020 |
| Parietal Sup R - Heschl R | 0.085 | 0.001 | 2.334 | 0.020 |
| Parietal Sup R - Vermis 8 | 0.062 | 0.004 | 2.872 | 0.004 |
| Parietal Inf L - Heschl R | 0.047 | 0.015 | 2.194 | 0.029 |
| Parietal Inf L - Cerebellum 6 R | 0.160 | < 0.001 | -2.520 | 0.012 |
| Parietal Inf L - Vermis 1 2 | 0.047 | 0.015 | -2.314 | 0.021 |
| Parietal Inf R - Heschl R | 0.093 | < 0.001 | 3.104 | 0.002 |
| Parietal Inf R - Vermis 1 2 | 0.108 | < 0.001 | -2.204 | 0.028 |
| SupraMarginal R - Cerebellum 3 L | 0.067 | 0.003 | -1.990 | 0.048 |
| Angular L - Temporal Pole Sup R | 0.057 | 0.007 | 2.158 | 0.032 |
| Angular R - Heschl R | 0.038 | 0.031 | 2.111 | 0.036 |
| Angular R - Vermis 7 | 0.050 | 0.012 | -2.044 | 0.042 |
| Precuneus L - Cerebellum 7b R | 0.071 | 0.002 | -2.065 | 0.040 |
| Precuneus R - Vermis 8 | 0.081 | 0.001 | 2.071 | 0.039 |
| Paracentral Lobule L - Pallidum L | 0.036 | 0.036 | -1.971 | 0.050 |
| Paracentral Lobule L - Temporal Inf R | 0.144 | < 0.001 | -2.379 | 0.018 |
| Paracentral Lobule L - Cerebellum 10 L | 0.108 | < 0.001 | 2.128 | 0.034 |
| Caudate L - Heschl R | 0.121 | < 0.001 | 2.643 | 0.009 |
| Caudate L - Temporal Sup L | 0.126 | < 0.001 | 2.309 | 0.022 |
| Caudate L - Cerebellum 4 5 R | 0.037 | 0.036 | 2.407 | 0.017 |
| Caudate R - Heschl R | 0.146 | < 0.001 | 2.455 | 0.015 |
| Caudate R - Cerebellum 7b L | 0.084 | 0.001 | -2.140 | 0.033 |
| Caudate R - Cerebellum 10 R | 0.067 | 0.003 | -2.276 | 0.024 |
| Putamen L - Thalamus L | 0.274 | < 0.001 | 2.026 | 0.044 |
| Putamen L - Cerebellum 4 5 L | 0.127 | < 0.001 | -2.678 | 0.008 |
| Putamen L - Cerebellum 7b L | 0.099 | < 0.001 | -2.018 | 0.045 |
| Putamen L - Cerebellum 7b R | 0.205 | < 0.001 | -2.049 | 0.042 |
| Putamen L - Cerebellum 8 L | 0.068 | 0.002 | -2.433 | 0.016 |
| Putamen R - Heschl R | 0.194 | < 0.001 | 2.075 | 0.039 |
| Putamen R - Cerebellum 3 R | 0.075 | 0.001 | -2.162 | 0.032 |
| Putamen R - Vermis 6 | 0.104 | < 0.001 | -2.057 | 0.041 |
| Pallidum L - Heschl R | 0.103 | < 0.001 | 2.542 | 0.012 |
| Pallidum L - Cerebellum 3 L | 0.096 | < 0.001 | 1.972 | 0.050 |
| Pallidum R - Cerebellum 3 R | 0.061 | 0.004 | -2.137 | 0.034 |
| Pallidum R - Cerebellum 4 5 L | 0.096 | < 0.001 | -2.184 | 0.030 |
| Pallidum R - Vermis 10 | 0.057 | 0.007 | -2.310 | 0.022 |
| Thalamus L - Heschl R | 0.156 | < 0.001 | 3.219 | 0.001 |
| Thalamus L - Cerebellum 10 R | 0.036 | 0.037 | -2.119 | 0.035 |
| Thalamus R - Heschl R | 0.154 | < 0.001 | 3.159 | 0.002 |
| Thalamus R - Temporal Pole Mid R | 0.218 | < 0.001 | 2.084 | 0.038 |
| Heschl L - Vermis 4 5 | 0.082 | 0.001 | 2.955 | 0.003 |
| Heschl R - Cerebellum 10 R | 0.092 | < 0.001 | -3.076 | 0.002 |
| Heschl R - Vermis 4 5 | 0.134 | < 0.001 | 3.507 | 0.001 |
| Temporal Sup L - Vermis 4 5 | 0.112 | < 0.001 | 2.581 | 0.010 |
| Temporal Sup R - Cerebellum 3 R | 0.131 | < 0.001 | -2.596 | 0.010 |
| Temporal Sup R - Vermis 4 5 | 0.187 | < 0.001 | 2.000 | 0.047 |
| Temporal Pole Sup L - Cerebellum 3 L | 0.134 | < 0.001 | 2.199 | 0.029 |
| Temporal Pole Sup L - Cerebellum 4 5 R | 0.099 | < 0.001 | 2.257 | 0.025 |
| Temporal Pole Sup R - Cerebellum Crus1 R | 0.099 | < 0.001 | 2.255 | 0.025 |
| Temporal Pole Sup R - Cerebellum 4 5 R | 0.103 | < 0.001 | 2.129 | 0.034 |
| Temporal Pole Sup R - Vermis 4 5 | 0.129 | < 0.001 | 2.213 | 0.028 |
| Temporal Mid L - Cerebellum 3 L | 0.154 | < 0.001 | 2.180 | 0.030 |
| Temporal Mid L - Cerebellum 4 5 L | 0.157 | < 0.001 | 2.248 | 0.025 |
| Temporal Mid L - Cerebellum 4 5 R | 0.093 | < 0.001 | 2.215 | 0.028 |
| Temporal Mid L - Vermis 4 5 | 0.212 | < 0.001 | 2.574 | 0.011 |
| Temporal Mid L - Vermis 10 | 0.088 | < 0.001 | -2.105 | 0.036 |
| Temporal Mid R - Cerebellum 4 5 R | 0.142 | < 0.001 | 2.897 | 0.004 |
| Temporal Mid R - Vermis 4 5 | 0.277 | < 0.001 | 2.004 | 0.046 |
| Temporal Pole Mid L - Temporal Pole Mid R | 0.452 | < 0.001 | 2.694 | 0.008 |
| Temporal Pole Mid L - Cerebellum Crus1 L | 0.175 | < 0.001 | 2.540 | 0.012 |
| Temporal Pole Mid L - Cerebellum Crus2 L | 0.099 | < 0.001 | 2.023 | 0.044 |
| Temporal Pole Mid L - Cerebellum Crus2 R | 0.111 | < 0.001 | 2.826 | 0.005 |
| Temporal Pole Mid R - Cerebellum Crus2 R | 0.144 | < 0.001 | 3.861 | < 0.001 |
| Temporal Pole Mid R - Cerebellum 6 R | 0.084 | 0.001 | 2.029 | 0.044 |
| Temporal Pole Mid R - Cerebellum 10 L | 0.044 | 0.019 | 2.139 | 0.033 |
| Temporal Inf L - Cerebellum Crus2 R | 0.221 | < 0.001 | 2.063 | 0.040 |
| Temporal Inf L - Cerebellum 3 L | 0.098 | < 0.001 | 2.757 | 0.006 |
| Temporal Inf L - Cerebellum 4 5 L | 0.174 | < 0.001 | 2.447 | 0.015 |
| Temporal Inf L - Cerebellum 4 5 R | 0.151 | < 0.001 | 2.484 | 0.014 |
| Temporal Inf L - Cerebellum 10 R | 0.154 | < 0.001 | 2.013 | 0.045 |
| Temporal Inf L - Vermis 4 5 | 0.157 | < 0.001 | 2.585 | 0.010 |
| Temporal Inf L - Vermis 6 | 0.104 | < 0.001 | 2.463 | 0.014 |
| Temporal Inf R - Cerebellum Crus2 R | 0.191 | < 0.001 | 2.049 | 0.041 |
| Cerebellum Crus1 L - Cerebellum 4 5 L | 0.201 | < 0.001 | 2.889 | 0.004 |
| Cerebellum Crus1 L - Cerebellum 4 5 R | 0.142 | < 0.001 | 3.042 | 0.003 |
| Cerebellum Crus1 L - Vermis 8 | 0.147 | < 0.001 | 2.754 | 0.006 |
| Cerebellum Crus1 R - Cerebellum 4 5 R | 0.119 | < 0.001 | 2.743 | 0.007 |
| Cerebellum Crus1 R - Vermis 8 | 0.146 | < 0.001 | 2.994 | 0.003 |
| Cerebellum Crus2 L - Cerebellum 4 5 L | 0.193 | < 0.001 | 2.432 | 0.016 |
| Cerebellum Crus2 L - Cerebellum 8 L | 0.272 | < 0.001 | 2.924 | 0.004 |
| Cerebellum Crus2 L - Cerebellum 10 L | 0.163 | < 0.001 | 2.073 | 0.039 |
| Cerebellum Crus2 L - Vermis 8 | 0.214 | < 0.001 | 3.588 | < 0.001 |
| Cerebellum Crus2 R - Cerebellum 4 5 L | 0.174 | < 0.001 | 2.545 | 0.012 |
| Cerebellum Crus2 R - Cerebellum 4 5 R | 0.247 | < 0.001 | 2.870 | 0.004 |
| Cerebellum Crus2 R - Cerebellum 7b L | 0.136 | < 0.001 | 2.704 | 0.007 |
| Cerebellum Crus2 R - Cerebellum 7b R | 0.401 | < 0.001 | 2.525 | 0.012 |
| Cerebellum Crus2 R - Cerebellum 8 L | 0.204 | < 0.001 | 3.153 | 0.002 |
| Cerebellum Crus2 R - Cerebellum 8 R | 0.235 | < 0.001 | 2.828 | 0.005 |
| Cerebellum Crus2 R - Cerebellum 10 L | 0.100 | < 0.001 | 2.524 | 0.012 |
| Cerebellum Crus2 R - Vermis 8 | 0.222 | < 0.001 | 3.078 | 0.002 |
| Cerebellum 3 L - Cerebellum 9 L | 0.177 | < 0.001 | -2.074 | 0.039 |
| Cerebellum 4 5 L - Cerebellum 10 R | 0.073 | 0.002 | 2.433 | 0.016 |
| Cerebellum 4 5 R - Cerebellum 6 R | 0.280 | < 0.001 | 2.442 | 0.015 |
| Cerebellum 4 5 R - Vermis 1 2 | 0.118 | < 0.001 | 2.133 | 0.034 |
| Cerebellum 6 L - Vermis 8 | 0.246 | < 0.001 | 2.220 | 0.027 |
| Cerebellum 6 L - Vermis 9 | 0.185 | < 0.001 | 2.212 | 0.028 |
| Cerebellum 6 R - Vermis 8 | 0.229 | < 0.001 | 2.266 | 0.024 |
| Cerebellum 6 R - Vermis 9 | 0.176 | < 0.001 | 2.109 | 0.036 |
| Cerebellum 7b L - Cerebellum 7b R | 0.279 | < 0.001 | 2.000 | 0.047 |
| Cerebellum 7b L - Cerebellum 8 L | 0.495 | < 0.001 | 2.962 | 0.003 |
| Cerebellum 7b L - Cerebellum 8 R | 0.393 | < 0.001 | 2.206 | 0.028 |
| Cerebellum 7b L - Cerebellum 10 L | 0.092 | < 0.001 | 3.348 | 0.001 |
| Cerebellum 7b L - Cerebellum 10 R | 0.122 | < 0.001 | 1.993 | 0.047 |
| Cerebellum 7b L - Vermis 8 | 0.208 | < 0.001 | 2.533 | 0.012 |
| Cerebellum 7b R - Cerebellum 8 L | 0.264 | < 0.001 | 2.141 | 0.033 |
| Cerebellum 7b R - Cerebellum 8 R | 0.400 | < 0.001 | 2.515 | 0.013 |
| Cerebellum 7b R - Cerebellum 10 L | 0.091 | < 0.001 | 2.758 | 0.006 |
| Cerebellum 7b R - Vermis 8 | 0.167 | < 0.001 | 2.562 | 0.011 |
| Cerebellum 8 L - Cerebellum 9 R | 0.242 | < 0.001 | 2.126 | 0.034 |
| Cerebellum 8 L - Cerebellum 10 L | 0.142 | < 0.001 | 3.233 | 0.001 |
| Cerebellum 8 L - Vermis 8 | 0.283 | < 0.001 | 2.364 | 0.019 |
| Cerebellum 8 R - Cerebellum 10 L | 0.090 | < 0.001 | 2.343 | 0.020 |
| Cerebellum 8 R - Vermis 8 | 0.236 | < 0.001 | 2.085 | 0.038 |
| Cerebellum 9 R - Vermis 8 | 0.145 | < 0.001 | 2.114 | 0.036 |
| Cerebellum 10 R - Vermis 4 5 | 0.047 | 0.015 | 2.116 | 0.035 |
| Vermis 7 - Vermis 8 | 0.249 | < 0.001 | 2.827 | 0.005 |

**Supplementary Table 16**. Frailty on ROI-to-ROI functional connectivity for CU adjusting for scanner type and data quality metrics. *P*_FDR_<0.05. Regions are presented using the AAL atlas.

| **Regions** | **Model** | | **Frailty** | |
| --- | --- | --- | --- | --- |
|  | **R^2^_adj_** | ***P*_FDR_** | ***t*** | ***P*_FDR_** |
| Precentral L - Frontal Mid R | 0.113 | < 0.001 | 1.972 | 0.049 |
| Precentral L - Frontal Mid Orb R | 0.125 | < 0.001 | 2.147 | 0.033 |
| Precentral L - Frontal Sup Medial R | 0.131 | < 0.001 | 2.103 | 0.036 |
| Precentral L - Cuneus L | 0.146 | < 0.001 | -2.085 | 0.038 |
| Precentral L - Lingual L | 0.073 | < 0.001 | -2.014 | 0.045 |
| Precentral L - Occipital Sup L | 0.059 | 0.002 | -2.030 | 0.043 |
| Precentral L - Occipital Mid L | 0.040 | 0.016 | -2.411 | 0.017 |
| Precentral L - Fusiform L | 0.192 | < 0.001 | -2.360 | 0.019 |
| Precentral L - Fusiform R | 0.179 | < 0.001 | -1.979 | 0.049 |
| Precentral L - Cerebellum 8 L | 0.087 | < 0.001 | -2.067 | 0.040 |
| Precentral L - Vermis 8 | 0.113 | < 0.001 | -1.994 | 0.047 |
| Precentral R - Rolandic Oper R | 0.091 | < 0.001 | -3.136 | 0.002 |
| Precentral R - Cingulum Ant L | 0.065 | 0.001 | 1.999 | 0.047 |
| Precentral R - Calcarine R | 0.031 | 0.036 | -2.214 | 0.028 |
| Precentral R - Cuneus R | 0.047 | 0.007 | -2.091 | 0.037 |
| Precentral R - Occipital Sup L | 0.051 | 0.005 | -2.208 | 0.028 |
| Precentral R - Occipital Sup R | 0.067 | 0.001 | -2.599 | 0.010 |
| Precentral R - Occipital Mid L | 0.030 | 0.040 | -2.355 | 0.019 |
| Precentral R - Occipital Mid R | 0.037 | 0.021 | -2.338 | 0.020 |
| Precentral R - Vermis 10 | 0.176 | < 0.001 | -2.079 | 0.038 |
| Frontal Sup L - Frontal Mid L | 0.242 | < 0.001 | 1.985 | 0.048 |
| Frontal Sup R - Frontal Mid R | 0.224 | < 0.001 | 2.021 | 0.044 |
| Frontal Sup R - Vermis 10 | 0.179 | < 0.001 | -2.150 | 0.032 |
| Frontal Sup Orb L - Supp Motor Area L | 0.163 | < 0.001 | 2.258 | 0.025 |
| Frontal Sup Orb L - Supp Motor Area R | 0.178 | < 0.001 | 2.136 | 0.033 |
| Frontal Sup Orb L - ParaHippocampal L | 0.112 | < 0.001 | 2.228 | 0.027 |
| Frontal Sup Orb L - Cerebellum 4 5 L | 0.208 | < 0.001 | 2.183 | 0.030 |
| Frontal Sup Orb L - Vermis 6 | 0.094 | < 0.001 | 2.210 | 0.028 |
| Frontal Sup Orb L - Vermis 9 | 0.085 | < 0.001 | 2.619 | 0.009 |
| Frontal Sup Orb R - Frontal Inf Oper L | 0.103 | < 0.001 | 2.259 | 0.025 |
| Frontal Sup Orb R - Frontal Inf Oper R | 0.109 | < 0.001 | 2.066 | 0.040 |
| Frontal Sup Orb R - Supp Motor Area L | 0.203 | < 0.001 | 2.242 | 0.026 |
| Frontal Sup Orb R - Supp Motor Area R | 0.182 | < 0.001 | 2.004 | 0.046 |
| Frontal Sup Orb R - Hippocampus L | 0.249 | < 0.001 | 2.352 | 0.019 |
| Frontal Sup Orb R - Amygdala L | 0.172 | < 0.001 | 2.339 | 0.020 |
| Frontal Sup Orb R - Lingual L | 0.210 | < 0.001 | 2.051 | 0.041 |
| Frontal Sup Orb R - Occipital Inf L | 0.145 | < 0.001 | 2.091 | 0.037 |
| Frontal Sup Orb R - Caudate R | 0.131 | < 0.001 | 2.113 | 0.035 |
| Frontal Sup Orb R - Thalamus R | 0.121 | < 0.001 | 2.030 | 0.043 |
| Frontal Sup Orb R - Heschl L | 0.105 | < 0.001 | 2.608 | 0.010 |
| Frontal Sup Orb R - Temporal Sup L | 0.162 | < 0.001 | 1.969 | 0.050 |
| Frontal Sup Orb R - Cerebellum 4 5 L | 0.241 | < 0.001 | 2.353 | 0.019 |
| Frontal Sup Orb R - Cerebellum 4 5 R | 0.224 | < 0.001 | 2.332 | 0.020 |
| Frontal Mid L - Frontal Sup Medial L | 0.195 | < 0.001 | 2.376 | 0.018 |
| Frontal Mid L - Cerebellum Crus1 L | 0.294 | < 0.001 | -2.034 | 0.043 |
| Frontal Mid L - Cerebellum 8 L | 0.130 | < 0.001 | -2.184 | 0.030 |
| Frontal Mid L - Vermis 7 | 0.106 | < 0.001 | -2.014 | 0.045 |
| Frontal Mid L - Vermis 8 | 0.136 | < 0.001 | -2.805 | 0.005 |
| Frontal Mid L - Vermis 9 | 0.152 | < 0.001 | -2.225 | 0.027 |
| Frontal Mid R - Supp Motor Area L | 0.096 | < 0.001 | 2.037 | 0.043 |
| Frontal Mid R - Supp Motor Area R | 0.111 | < 0.001 | 2.513 | 0.013 |
| Frontal Mid R - Frontal Sup Medial L | 0.140 | < 0.001 | 2.657 | 0.008 |
| Frontal Mid R - Frontal Sup Medial R | 0.157 | < 0.001 | 2.436 | 0.015 |
| Frontal Mid R - Cingulum Ant R | 0.093 | < 0.001 | 2.330 | 0.020 |
| Frontal Mid Orb L - Olfactory L | 0.091 | < 0.001 | 2.002 | 0.046 |
| Frontal Mid Orb L - Frontal Sup Medial R | 0.072 | 0.001 | 2.454 | 0.015 |
| Frontal Mid Orb L - ParaHippocampal L | 0.129 | < 0.001 | 2.824 | 0.005 |
| Frontal Mid Orb L - ParaHippocampal R | 0.129 | < 0.001 | 2.610 | 0.010 |
| Frontal Mid Orb L - Lingual L | 0.174 | < 0.001 | 2.268 | 0.024 |
| Frontal Mid Orb L - Cerebellum 4 5 L | 0.207 | < 0.001 | 2.601 | 0.010 |
| Frontal Mid Orb L - Vermis 1 2 | 0.218 | < 0.001 | 2.853 | 0.005 |
| Frontal Mid Orb L - Vermis 3 | 0.140 | < 0.001 | 2.339 | 0.020 |
| Frontal Mid Orb R - Supp Motor Area R | 0.156 | < 0.001 | 2.271 | 0.024 |
| Frontal Mid Orb R - Frontal Sup Medial R | 0.127 | < 0.001 | 3.608 | < 0.001 |
| Frontal Mid Orb R - ParaHippocampal L | 0.094 | < 0.001 | 3.127 | 0.002 |
| Frontal Mid Orb R - ParaHippocampal R | 0.086 | < 0.001 | 2.138 | 0.033 |
| Frontal Mid Orb R - Amygdala L | 0.223 | < 0.001 | 2.249 | 0.025 |
| Frontal Mid Orb R - Heschl L | 0.061 | 0.002 | 2.362 | 0.019 |
| Frontal Mid Orb R - Cerebellum 3 L | 0.187 | < 0.001 | 2.550 | 0.011 |
| Frontal Mid Orb R - Cerebellum 4 5 L | 0.246 | < 0.001 | 2.790 | 0.006 |
| Frontal Mid Orb R - Cerebellum 4 5 R | 0.204 | < 0.001 | 2.023 | 0.044 |
| Frontal Mid Orb R - Cerebellum 6 L | 0.112 | < 0.001 | 2.807 | 0.005 |
| Frontal Mid Orb R - Vermis 1 2 | 0.263 | < 0.001 | 2.073 | 0.039 |
| Frontal Inf Oper L - Postcentral L | 0.055 | 0.003 | 2.315 | 0.021 |
| Frontal Inf Oper L - Cerebellum 4 5 R | 0.109 | < 0.001 | -2.010 | 0.045 |
| Frontal Inf Oper R - Frontal Sup Medial R | 0.143 | < 0.001 | 2.032 | 0.043 |
| Frontal Inf Oper R - Pallidum L | 0.090 | < 0.001 | -2.128 | 0.034 |
| Frontal Inf Tri L - Cingulum Post R | 0.142 | < 0.001 | 2.335 | 0.020 |
| Frontal Inf Tri L - Cerebellum 4 5 R | 0.105 | < 0.001 | -2.468 | 0.014 |
| Frontal Inf Tri R - Temporal Pole Mid L | 0.149 | < 0.001 | 2.437 | 0.015 |
| Frontal Inf Tri R - Cerebellum 10 L | 0.120 | < 0.001 | -2.605 | 0.010 |
| Frontal Inf Tri R - Cerebellum 10 R | 0.175 | < 0.001 | -2.094 | 0.037 |
| Frontal Inf Tri R - Vermis 3 | 0.118 | < 0.001 | -2.045 | 0.042 |
| Frontal Inf Orb L - ParaHippocampal L | 0.113 | < 0.001 | 2.039 | 0.042 |
| Frontal Inf Orb L - Vermis 3 | 0.088 | < 0.001 | 2.715 | 0.007 |
| Frontal Inf Orb L - Vermis 6 | 0.110 | < 0.001 | 2.014 | 0.045 |
| Frontal Inf Orb R - Supp Motor Area R | 0.145 | < 0.001 | 2.508 | 0.013 |
| Frontal Inf Orb R - Hippocampus R | 0.334 | < 0.001 | 2.090 | 0.037 |
| Frontal Inf Orb R - ParaHippocampal L | 0.090 | < 0.001 | 2.515 | 0.012 |
| Frontal Inf Orb R - Fusiform L | 0.085 | < 0.001 | 2.023 | 0.044 |
| Frontal Inf Orb R - Cerebellum 4 5 L | 0.223 | < 0.001 | 1.991 | 0.047 |
| Frontal Inf Orb R - Cerebellum 6 L | 0.116 | < 0.001 | 2.661 | 0.008 |
| Frontal Inf Orb R - Cerebellum 6 R | 0.088 | < 0.001 | 2.106 | 0.036 |
| Frontal Inf Orb R - Vermis 6 | 0.146 | < 0.001 | 2.357 | 0.019 |
| Rolandic Oper L - Cingulum Mid L | 0.047 | 0.007 | -2.906 | 0.004 |
| Rolandic Oper L - Cingulum Mid R | 0.055 | 0.003 | -2.051 | 0.041 |
| Rolandic Oper L - Lingual R | 0.049 | 0.006 | -2.064 | 0.040 |
| Rolandic Oper L - Fusiform L | 0.065 | 0.001 | -2.068 | 0.039 |
| Rolandic Oper L - Precuneus L | 0.106 | < 0.001 | -2.041 | 0.042 |
| Rolandic Oper L - Precuneus R | 0.107 | < 0.001 | -2.156 | 0.032 |
| Rolandic Oper L - Vermis 1 2 | 0.135 | < 0.001 | 2.319 | 0.021 |
| Rolandic Oper R - Insula L | 0.125 | < 0.001 | -2.317 | 0.021 |
| Rolandic Oper R - Insula R | 0.053 | 0.004 | -2.239 | 0.026 |
| Rolandic Oper R - Lingual R | 0.059 | 0.002 | -1.990 | 0.047 |
| Rolandic Oper R - SupraMarginal R | 0.161 | < 0.001 | -2.160 | 0.032 |
| Rolandic Oper R - Paracentral Lobule R | 0.062 | 0.001 | -2.449 | 0.015 |
| Rolandic Oper R - Temporal Sup L | 0.102 | < 0.001 | -2.062 | 0.040 |
| Rolandic Oper R - Temporal Sup R | 0.176 | < 0.001 | -2.471 | 0.014 |
| Rolandic Oper R - Cerebellum 4 5 R | 0.039 | 0.017 | -2.128 | 0.034 |
| Rolandic Oper R - Cerebellum 8 L | 0.073 | < 0.001 | -2.182 | 0.030 |
| Supp Motor Area R - Frontal Sup Medial L | 0.097 | < 0.001 | 2.126 | 0.034 |
| Supp Motor Area R - Rectus L | 0.227 | < 0.001 | 2.040 | 0.042 |
| Supp Motor Area R - Rectus R | 0.211 | < 0.001 | 2.424 | 0.016 |
| Supp Motor Area R - Insula L | 0.046 | 0.008 | -2.281 | 0.023 |
| Olfactory L - Cerebellum 10 L | 0.124 | < 0.001 | 2.374 | 0.018 |
| Olfactory R - Heschl R | 0.046 | 0.008 | 2.316 | 0.021 |
| Olfactory R - Cerebellum 6 R | 0.130 | < 0.001 | 2.123 | 0.035 |
| Frontal Sup Medial L - Cerebellum 9 R | 0.086 | < 0.001 | 2.006 | 0.046 |
| Frontal Med Orb L - Vermis 1 2 | 0.152 | < 0.001 | 2.379 | 0.018 |
| Frontal Med Orb R - ParaHippocampal L | 0.048 | 0.007 | 2.086 | 0.038 |
| Frontal Med Orb R - Amygdala L | 0.105 | < 0.001 | 2.535 | 0.012 |
| Frontal Med Orb R - Parietal Sup L | 0.199 | < 0.001 | 2.444 | 0.015 |
| Frontal Med Orb R - Parietal Inf L | 0.190 | < 0.001 | 2.083 | 0.038 |
| Frontal Med Orb R - Caudate L | 0.084 | < 0.001 | -2.075 | 0.039 |
| Frontal Med Orb R - Cerebellum 6 L | 0.114 | < 0.001 | 2.294 | 0.023 |
| Frontal Med Orb R - Cerebellum 8 R | 0.195 | < 0.001 | 2.598 | 0.010 |
| Frontal Med Orb R - Vermis 6 | 0.167 | < 0.001 | 2.938 | 0.004 |
| Rectus L - ParaHippocampal L | 0.133 | < 0.001 | 3.595 | < 0.001 |
| Rectus L - ParaHippocampal R | 0.173 | < 0.001 | 2.215 | 0.028 |
| Rectus L - Amygdala L | 0.143 | < 0.001 | 2.565 | 0.011 |
| Rectus L - Lingual L | 0.200 | < 0.001 | 2.709 | 0.007 |
| Rectus L - Occipital Mid L | 0.160 | < 0.001 | 2.338 | 0.020 |
| Rectus L - Occipital Inf L | 0.233 | < 0.001 | 2.781 | 0.006 |
| Rectus L - Occipital Inf R | 0.186 | < 0.001 | 2.315 | 0.021 |
| Rectus L - Heschl L | 0.077 | < 0.001 | 2.004 | 0.046 |
| Rectus L - Temporal Inf R | 0.145 | < 0.001 | 2.441 | 0.015 |
| Rectus L - Cerebellum Crus2 L | 0.116 | < 0.001 | 2.094 | 0.037 |
| Rectus L - Cerebellum Crus2 R | 0.116 | < 0.001 | 2.000 | 0.046 |
| Rectus L - Cerebellum 3 L | 0.221 | < 0.001 | 2.780 | 0.006 |
| Rectus L - Cerebellum 4 5 L | 0.217 | < 0.001 | 3.439 | 0.001 |
| Rectus L - Cerebellum 6 L | 0.201 | < 0.001 | 2.454 | 0.015 |
| Rectus L - Cerebellum 6 R | 0.199 | < 0.001 | 2.475 | 0.014 |
| Rectus L - Cerebellum 8 R | 0.165 | < 0.001 | 2.112 | 0.036 |
| Rectus L - Cerebellum 10 L | 0.144 | < 0.001 | 2.283 | 0.023 |
| Rectus L - Vermis 6 | 0.180 | < 0.001 | 2.527 | 0.012 |
| Rectus R - ParaHippocampal L | 0.107 | < 0.001 | 2.499 | 0.013 |
| Rectus R - ParaHippocampal R | 0.175 | < 0.001 | 3.294 | 0.001 |
| Rectus R - Amygdala L | 0.108 | < 0.001 | 2.572 | 0.011 |
| Rectus R - Occipital Inf L | 0.174 | < 0.001 | 2.286 | 0.023 |
| Rectus R - Occipital Inf R | 0.183 | < 0.001 | 2.554 | 0.011 |
| Rectus R - Fusiform R | 0.161 | < 0.001 | 2.172 | 0.031 |
| Rectus R - Heschl L | 0.084 | < 0.001 | 2.321 | 0.021 |
| Rectus R - Temporal Pole Sup L | 0.214 | < 0.001 | 1.997 | 0.047 |
| Rectus R - Temporal Inf L | 0.101 | < 0.001 | 2.548 | 0.011 |
| Rectus R - Temporal Inf R | 0.143 | < 0.001 | 2.462 | 0.014 |
| Rectus R - Cerebellum Crus1 L | 0.095 | < 0.001 | 2.059 | 0.040 |
| Rectus R - Cerebellum 3 L | 0.167 | < 0.001 | 2.208 | 0.028 |
| Rectus R - Cerebellum 4 5 L | 0.205 | < 0.001 | 2.485 | 0.013 |
| Rectus R - Cerebellum 4 5 R | 0.216 | < 0.001 | 2.604 | 0.010 |
| Rectus R - Cerebellum 6 L | 0.216 | < 0.001 | 2.886 | 0.004 |
| Rectus R - Vermis 6 | 0.194 | < 0.001 | 2.033 | 0.043 |
| Rectus R - Vermis 8 | 0.068 | 0.001 | 2.683 | 0.008 |
| Rectus R - Vermis 9 | 0.155 | < 0.001 | 2.009 | 0.045 |
| Insula L - Cingulum Ant L | 0.071 | 0.001 | -2.119 | 0.035 |
| Insula L - Cingulum Mid L | 0.037 | 0.021 | -2.007 | 0.046 |
| Insula L - Cingulum Mid R | 0.078 | < 0.001 | -2.270 | 0.024 |
| Insula L - Cerebellum Crus1 R | 0.247 | < 0.001 | -2.103 | 0.036 |
| Insula R - Cingulum Ant R | 0.109 | < 0.001 | -2.631 | 0.009 |
| Insula R - Cingulum Mid L | 0.050 | 0.006 | -2.342 | 0.020 |
| Insula R - Calcarine L | 0.105 | < 0.001 | -2.208 | 0.028 |
| Insula R - Occipital Inf L | 0.102 | < 0.001 | -2.044 | 0.042 |
| Insula R - Fusiform L | 0.126 | < 0.001 | -2.505 | 0.013 |
| Insula R - Fusiform R | 0.163 | < 0.001 | -2.148 | 0.033 |
| Insula R - Pallidum L | 0.159 | < 0.001 | -2.086 | 0.038 |
| Insula R - Vermis 6 | 0.052 | 0.004 | -2.082 | 0.038 |
| Cingulum Ant L - Fusiform R | 0.193 | < 0.001 | 2.014 | 0.045 |
| Cingulum Ant L - SupraMarginal R | 0.154 | < 0.001 | 2.202 | 0.028 |
| Cingulum Ant R - Hippocampus L | 0.182 | < 0.001 | 2.061 | 0.040 |
| Cingulum Ant R - Cerebellum 8 L | 0.163 | < 0.001 | 2.191 | 0.029 |
| Cingulum Mid L - Fusiform L | 0.075 | < 0.001 | -2.007 | 0.046 |
| Cingulum Mid L - Caudate L | 0.144 | < 0.001 | -2.067 | 0.040 |
| Cingulum Mid L - Heschl L | 0.092 | < 0.001 | -2.558 | 0.011 |
| Cingulum Mid L - Heschl R | 0.093 | < 0.001 | -3.179 | 0.002 |
| Cingulum Mid L - Cerebellum 8 L | 0.125 | < 0.001 | -2.278 | 0.023 |
| Cingulum Mid L - Vermis 8 | 0.151 | < 0.001 | -2.868 | 0.004 |
| Cingulum Mid R - Heschl R | 0.081 | < 0.001 | -2.578 | 0.010 |
| Cingulum Mid R - Cerebellum 4 5 L | 0.043 | 0.011 | -1.997 | 0.047 |
| Cingulum Mid R - Cerebellum 8 L | 0.140 | < 0.001 | -2.697 | 0.007 |
| Cingulum Mid R - Vermis 8 | 0.139 | < 0.001 | -2.493 | 0.013 |
| Cingulum Post L - Hippocampus L | 0.119 | < 0.001 | -2.142 | 0.033 |
| Cingulum Post L - Cuneus R | 0.169 | < 0.001 | 2.033 | 0.043 |
| Cingulum Post L - Occipital Mid R | 0.113 | < 0.001 | 1.995 | 0.047 |
| Cingulum Post L - SupraMarginal R | 0.099 | < 0.001 | 2.218 | 0.027 |
| Cingulum Post R - Putamen R | 0.229 | < 0.001 | 2.266 | 0.024 |
| Cingulum Post R - Cerebellum Crus2 R | 0.089 | < 0.001 | -2.183 | 0.030 |
| Hippocampus L - Precuneus L | 0.122 | < 0.001 | -2.200 | 0.029 |
| Hippocampus R - Precuneus L | 0.137 | < 0.001 | -2.431 | 0.016 |
| Hippocampus R - Cerebellum 4 5 R | 0.171 | < 0.001 | 2.510 | 0.013 |
| Hippocampus R - Vermis 1 2 | 0.090 | < 0.001 | 2.102 | 0.036 |
| ParaHippocampal L - Temporal Sup L | 0.145 | < 0.001 | 2.031 | 0.043 |
| ParaHippocampal R - Cerebellum 4 5 L | 0.110 | < 0.001 | 2.388 | 0.018 |
| ParaHippocampal R - Cerebellum 4 5 R | 0.109 | < 0.001 | 2.475 | 0.014 |
| ParaHippocampal R - Cerebellum 10 L | 0.073 | < 0.001 | 2.832 | 0.005 |
| ParaHippocampal R - Vermis 4 5 | 0.166 | < 0.001 | 2.307 | 0.022 |
| ParaHippocampal R - Vermis 6 | 0.114 | < 0.001 | 2.271 | 0.024 |
| Amygdala L - Angular L | 0.182 | < 0.001 | 2.421 | 0.016 |
| Amygdala L - Vermis 1 2 | 0.099 | < 0.001 | 2.276 | 0.024 |
| Amygdala R - Cuneus L | 0.051 | 0.005 | -2.032 | 0.043 |
| Amygdala R - Occipital Sup L | 0.059 | 0.002 | -2.360 | 0.019 |
| Amygdala R - Heschl L | 0.101 | < 0.001 | 2.091 | 0.037 |
| Amygdala R - Temporal Inf R | 0.092 | < 0.001 | -2.329 | 0.021 |
| Calcarine L - Occipital Mid L | 0.186 | < 0.001 | 2.268 | 0.024 |
| Calcarine L - Occipital Mid R | 0.143 | < 0.001 | 2.139 | 0.033 |
| Calcarine L - Occipital Inf L | 0.113 | < 0.001 | 2.038 | 0.042 |
| Calcarine L - Postcentral L | 0.086 | < 0.001 | -2.033 | 0.043 |
| Calcarine L - Cerebellum 4 5 L | 0.166 | < 0.001 | 2.465 | 0.014 |
| Calcarine L - Cerebellum 6 L | 0.221 | < 0.001 | 1.976 | 0.049 |
| Calcarine L - Vermis 3 | 0.044 | 0.010 | 2.589 | 0.010 |
| Calcarine L - Vermis 4 5 | 0.191 | < 0.001 | 1.984 | 0.048 |
| Calcarine L - Vermis 6 | 0.140 | < 0.001 | 2.093 | 0.037 |
| Calcarine R - Postcentral L | 0.044 | 0.011 | -2.524 | 0.012 |
| Calcarine R - Postcentral R | 0.059 | 0.002 | -2.053 | 0.041 |
| Calcarine R - Parietal Sup L | 0.043 | 0.011 | -2.084 | 0.038 |
| Calcarine R - Cerebellum 4 5 L | 0.202 | < 0.001 | 2.178 | 0.030 |
| Calcarine R - Cerebellum 10 R | 0.172 | < 0.001 | -1.983 | 0.048 |
| Calcarine R - Vermis 3 | 0.113 | < 0.001 | 2.122 | 0.035 |
| Calcarine R - Vermis 4 5 | 0.165 | < 0.001 | 2.296 | 0.022 |
| Cuneus L - Postcentral L | 0.106 | < 0.001 | -2.631 | 0.009 |
| Cuneus L - Postcentral R | 0.081 | < 0.001 | -2.729 | 0.007 |
| Cuneus L - Parietal Sup L | 0.116 | < 0.001 | -3.237 | 0.001 |
| Cuneus L - Parietal Sup R | 0.112 | < 0.001 | -3.541 | < 0.001 |
| Cuneus L - Angular L | 0.143 | < 0.001 | 2.260 | 0.025 |
| Cuneus L - Paracentral Lobule L | 0.184 | < 0.001 | -2.113 | 0.035 |
| Cuneus L - Paracentral Lobule R | 0.173 | < 0.001 | -2.326 | 0.021 |
| Cuneus L - Temporal Pole Sup R | 0.101 | < 0.001 | -2.347 | 0.020 |
| Cuneus L - Vermis 10 | 0.129 | < 0.001 | 2.571 | 0.011 |
| Cuneus R - Postcentral R | 0.038 | 0.019 | -2.315 | 0.021 |
| Cuneus R - Parietal Sup L | 0.043 | 0.011 | -2.218 | 0.027 |
| Cuneus R - Cerebellum 3 R | 0.125 | < 0.001 | 2.587 | 0.010 |
| Cuneus R - Vermis 3 | 0.122 | < 0.001 | 2.578 | 0.010 |
| Cuneus R - Vermis 4 5 | 0.119 | < 0.001 | 2.092 | 0.037 |
| Cuneus R - Vermis 10 | 0.159 | < 0.001 | 2.841 | 0.005 |
| Lingual L - Cerebellum 4 5 L | 0.192 | < 0.001 | 2.114 | 0.035 |
| Lingual L - Vermis 6 | 0.217 | < 0.001 | 2.503 | 0.013 |
| Lingual R - Occipital Sup R | 0.161 | < 0.001 | -2.158 | 0.032 |
| Lingual R - Postcentral L | 0.055 | 0.003 | -2.456 | 0.015 |
| Lingual R - Cerebellum 4 5 L | 0.159 | < 0.001 | 2.074 | 0.039 |
| Lingual R - Cerebellum 6 R | 0.356 | < 0.001 | 2.311 | 0.022 |
| Lingual R - Vermis 3 | 0.065 | 0.001 | 2.056 | 0.041 |
| Lingual R - Vermis 4 5 | 0.208 | < 0.001 | 2.007 | 0.046 |
| Lingual R - Vermis 6 | 0.208 | < 0.001 | 2.767 | 0.006 |
| Lingual R - Vermis 9 | 0.115 | < 0.001 | 2.169 | 0.031 |
| Occipital Sup L - Postcentral R | 0.039 | 0.017 | -2.138 | 0.033 |
| Occipital Sup L - Temporal Pole Sup L | 0.095 | < 0.001 | -2.772 | 0.006 |
| Occipital Sup L - Temporal Pole Mid R | 0.092 | < 0.001 | -2.488 | 0.013 |
| Occipital Sup L - Temporal Inf R | 0.097 | < 0.001 | -2.080 | 0.038 |
| Occipital Sup R - Postcentral R | 0.064 | 0.001 | -3.317 | 0.001 |
| Occipital Sup R - Parietal Sup L | 0.090 | < 0.001 | -2.111 | 0.036 |
| Occipital Sup R - Paracentral Lobule R | 0.094 | < 0.001 | -2.163 | 0.031 |
| Occipital Sup R - Temporal Pole Sup L | 0.102 | < 0.001 | -2.131 | 0.034 |
| Occipital Sup R - Temporal Pole Mid L | 0.077 | < 0.001 | -1.968 | 0.050 |
| Occipital Sup R - Vermis 1 2 | 0.232 | < 0.001 | 1.974 | 0.049 |
| Occipital Sup R - Vermis 3 | 0.193 | < 0.001 | 2.101 | 0.037 |
| Occipital Mid L - Parietal Sup L | 0.090 | < 0.001 | -2.441 | 0.015 |
| Occipital Mid L - Parietal Sup R | 0.087 | < 0.001 | -2.384 | 0.018 |
| Occipital Mid L - Temporal Inf R | 0.120 | < 0.001 | -2.082 | 0.038 |
| Occipital Mid L - Cerebellum 3 R | 0.186 | < 0.001 | 2.582 | 0.010 |
| Occipital Mid L - Cerebellum 4 5 L | 0.278 | < 0.001 | 3.624 | < 0.001 |
| Occipital Mid L - Cerebellum 4 5 R | 0.190 | < 0.001 | 3.577 | < 0.001 |
| Occipital Mid L - Cerebellum 6 L | 0.236 | < 0.001 | 2.249 | 0.025 |
| Occipital Mid L - Vermis 3 | 0.129 | < 0.001 | 3.473 | 0.001 |
| Occipital Mid L - Vermis 4 5 | 0.206 | < 0.001 | 3.446 | 0.001 |
| Occipital Mid L - Vermis 10 | 0.171 | < 0.001 | 2.142 | 0.033 |
| Occipital Mid R - Postcentral R | 0.034 | 0.027 | -2.900 | 0.004 |
| Occipital Mid R - Parietal Sup R | 0.110 | < 0.001 | -2.344 | 0.020 |
| Occipital Mid R - Cerebellum 4 5 L | 0.167 | < 0.001 | 2.679 | 0.008 |
| Occipital Mid R - Cerebellum 4 5 R | 0.136 | < 0.001 | 2.726 | 0.007 |
| Occipital Mid R - Vermis 3 | 0.131 | < 0.001 | 2.153 | 0.032 |
| Occipital Mid R - Vermis 4 5 | 0.125 | < 0.001 | 2.441 | 0.015 |
| Occipital Inf L - Cerebellum 4 5 L | 0.241 | < 0.001 | 2.727 | 0.007 |
| Occipital Inf L - Cerebellum 4 5 R | 0.153 | < 0.001 | 2.809 | 0.005 |
| Occipital Inf L - Cerebellum 6 L | 0.221 | < 0.001 | 2.300 | 0.022 |
| Occipital Inf L - Cerebellum 6 R | 0.215 | < 0.001 | 2.308 | 0.022 |
| Occipital Inf L - Vermis 3 | 0.100 | < 0.001 | 2.155 | 0.032 |
| Occipital Inf L - Vermis 6 | 0.130 | < 0.001 | 2.027 | 0.044 |
| Occipital Inf R - Cerebellum 4 5 L | 0.157 | < 0.001 | 2.899 | 0.004 |
| Occipital Inf R - Cerebellum 4 5 R | 0.176 | < 0.001 | 2.617 | 0.009 |
| Occipital Inf R - Cerebellum 6 R | 0.264 | < 0.001 | 1.999 | 0.047 |
| Occipital Inf R - Vermis 10 | 0.168 | < 0.001 | 2.219 | 0.027 |
| Fusiform L - Postcentral L | 0.130 | < 0.001 | -2.317 | 0.021 |
| Fusiform L - Postcentral R | 0.127 | < 0.001 | -2.552 | 0.011 |
| Fusiform L - Parietal Sup L | 0.129 | < 0.001 | -2.118 | 0.035 |
| Fusiform L - Parietal Sup R | 0.106 | < 0.001 | -2.160 | 0.032 |
| Fusiform L - Cerebellum 8 L | 0.225 | < 0.001 | -2.403 | 0.017 |
| Fusiform L - Cerebellum 8 R | 0.281 | < 0.001 | -2.002 | 0.046 |
| Fusiform R - Postcentral R | 0.124 | < 0.001 | -2.048 | 0.041 |
| Fusiform R - Cerebellum 3 L | 0.126 | < 0.001 | 2.415 | 0.016 |
| Fusiform R - Cerebellum 4 5 L | 0.193 | < 0.001 | 2.453 | 0.015 |
| Fusiform R - Cerebellum 4 5 R | 0.160 | < 0.001 | 2.721 | 0.007 |
| Fusiform R - Cerebellum 10 L | 0.196 | < 0.001 | 2.035 | 0.043 |
| Fusiform R - Vermis 3 | 0.142 | < 0.001 | 3.085 | 0.002 |
| Fusiform R - Vermis 4 5 | 0.198 | < 0.001 | 3.046 | 0.003 |
| Fusiform R - Vermis 6 | 0.167 | < 0.001 | 2.040 | 0.042 |
| Postcentral L - Pallidum R | 0.053 | 0.004 | 2.362 | 0.019 |
| Postcentral R - Parietal Inf R | 0.176 | < 0.001 | 2.360 | 0.019 |
| Parietal Sup L - Temporal Mid L | 0.075 | < 0.001 | -2.023 | 0.044 |
| Parietal Sup L - Vermis 10 | 0.185 | < 0.001 | -2.133 | 0.034 |
| Parietal Sup R - Caudate L | 0.150 | < 0.001 | 2.466 | 0.014 |
| Parietal Sup R - Putamen R | 0.094 | < 0.001 | 2.284 | 0.023 |
| Parietal Sup R - Temporal Pole Sup L | 0.109 | < 0.001 | -2.216 | 0.027 |
| Parietal Inf L - Vermis 8 | 0.087 | < 0.001 | -2.259 | 0.025 |
| Parietal Inf R - Pallidum L | 0.088 | < 0.001 | -2.089 | 0.038 |
| SupraMarginal L - Temporal Mid R | 0.037 | 0.022 | -2.037 | 0.043 |
| SupraMarginal L - Temporal Inf R | 0.098 | < 0.001 | -2.091 | 0.037 |
| SupraMarginal R - Heschl R | 0.052 | 0.004 | -2.260 | 0.025 |
| SupraMarginal R - Temporal Sup R | 0.222 | < 0.001 | -2.776 | 0.006 |
| SupraMarginal R - Temporal Mid R | 0.143 | < 0.001 | -2.133 | 0.034 |
| Angular L - Putamen R | 0.238 | < 0.001 | 1.983 | 0.048 |
| Angular L - Temporal Pole Sup R | 0.127 | < 0.001 | -2.604 | 0.010 |
| Angular L - Cerebellum Crus2 R | 0.101 | < 0.001 | -1.990 | 0.048 |
| Angular R - Temporal Pole Sup L | 0.131 | < 0.001 | -4.048 | < 0.001 |
| Angular R - Temporal Pole Sup R | 0.123 | < 0.001 | -2.698 | 0.007 |
| Precuneus L - Putamen R | 0.179 | < 0.001 | 2.188 | 0.029 |
| Precuneus L - Vermis 10 | 0.141 | < 0.001 | -2.202 | 0.028 |
| Precuneus R - Putamen R | 0.111 | < 0.001 | 2.122 | 0.035 |
| Precuneus R - Temporal Pole Sup L | 0.108 | < 0.001 | -1.974 | 0.049 |
| Paracentral Lobule R - Putamen R | 0.085 | < 0.001 | 2.061 | 0.040 |
| Paracentral Lobule R - Cerebellum 7b R | 0.077 | < 0.001 | 2.085 | 0.038 |
| Caudate R - Temporal Pole Mid L | 0.161 | < 0.001 | 2.068 | 0.040 |
| Caudate R - Cerebellum 8 L | 0.174 | < 0.001 | 2.044 | 0.042 |
| Caudate R - Cerebellum 8 R | 0.270 | < 0.001 | 3.011 | 0.003 |
| Putamen L - Putamen R | 0.370 | < 0.001 | -2.230 | 0.027 |
| Putamen L - Cerebellum 4 5 L | 0.172 | < 0.001 | 2.840 | 0.005 |
| Pallidum L - Cerebellum 3 L | 0.121 | < 0.001 | 2.451 | 0.015 |
| Thalamus L - Cerebellum 4 5 L | 0.162 | < 0.001 | 2.087 | 0.038 |
| Heschl R - Cerebellum 10 R | 0.120 | < 0.001 | 2.620 | 0.009 |
| Temporal Sup L - Cerebellum 4 5 L | 0.119 | < 0.001 | 2.035 | 0.043 |
| Temporal Sup L - Cerebellum 6 L | 0.106 | < 0.001 | 2.420 | 0.016 |
| Temporal Sup R - Vermis 9 | 0.055 | 0.003 | 2.205 | 0.028 |
| Temporal Pole Sup R - Temporal Mid L | 0.141 | < 0.001 | -2.113 | 0.035 |
| Temporal Pole Sup R - Cerebellum 3 R | 0.074 | < 0.001 | 2.014 | 0.045 |
| Temporal Mid L - Cerebellum 4 5 L | 0.212 | < 0.001 | 2.007 | 0.046 |
| Temporal Mid R - Cerebellum Crus1 R | 0.134 | < 0.001 | 2.072 | 0.039 |
| Temporal Mid R - Cerebellum 4 5 L | 0.159 | < 0.001 | 2.393 | 0.017 |
| Temporal Mid R - Cerebellum 4 5 R | 0.105 | < 0.001 | 2.384 | 0.018 |
| Temporal Mid R - Vermis 4 5 | 0.159 | < 0.001 | 2.290 | 0.023 |
| Temporal Mid R - Vermis 6 | 0.125 | < 0.001 | 2.708 | 0.007 |
| Temporal Pole Mid L - Vermis 7 | 0.069 | 0.001 | -1.994 | 0.047 |
| Temporal Inf L - Cerebellum 8 L | 0.170 | < 0.001 | -1.987 | 0.048 |
| Cerebellum Crus1 R - Cerebellum 7b R | 0.176 | < 0.001 | -1.982 | 0.048 |
| Cerebellum 4 5 R - Vermis 4 5 | 0.164 | < 0.001 | 2.694 | 0.007 |
| Cerebellum 4 5 R - Vermis 7 | 0.117 | < 0.001 | 2.262 | 0.024 |
| Cerebellum 9 L - Cerebellum 10 R | 0.095 | < 0.001 | -2.112 | 0.036 |
| Cerebellum 10 L - Cerebellum 10 R | 0.125 | < 0.001 | -1.977 | 0.049 |
| Vermis 1 2 - Vermis 7 | 0.168 | < 0.001 | -2.310 | 0.022 |
| Vermis 4 5 - Vermis 7 | 0.173 | < 0.001 | 2.132 | 0.034 |
| Vermis 6 - Vermis 7 | 0.161 | < 0.001 | 2.409 | 0.017 |

**Supplementary Table 17**. Frailty on ROI-to-ROI functional connectivity for AD adjusting for scanner type and data quality metrics. *P*_FDR_<0.05. Regions are presented using the AAL atlas.

| **Regions** | **Model** | | **Frailty** | |
| --- | --- | --- | --- | --- |
|  | **R^2^_adj_** | ***P*_FDR_** | ***t*** | ***P*_FDR_** |
| Precentral L - Calcarine L | 0.145 | 0.008 | -2.335 | 0.022 |
| Precentral L - Cuneus R | 0.167 | 0.003 | -2.660 | 0.009 |
| Precentral L - Lingual L | 0.201 | 0.001 | -2.735 | 0.007 |
| Precentral L - Lingual R | 0.200 | 0.001 | -2.939 | 0.004 |
| Precentral L - Occipital Sup L | 0.178 | 0.002 | -2.109 | 0.038 |
| Precentral L - Occipital Sup R | 0.180 | 0.002 | -2.681 | 0.009 |
| Precentral L - Occipital Mid L | 0.224 | 0.001 | -3.060 | 0.003 |
| Precentral L - Occipital Mid R | 0.147 | 0.007 | -3.354 | 0.001 |
| Precentral L - Occipital Inf L | 0.190 | 0.002 | -3.039 | 0.003 |
| Precentral L - Occipital Inf R | 0.094 | 0.038 | -2.069 | 0.041 |
| Precentral L - Fusiform L | 0.188 | 0.002 | -2.504 | 0.014 |
| Precentral L - Fusiform R | 0.223 | 0.001 | -1.996 | 0.049 |
| Precentral L - Parietal Sup R | 0.088 | 0.045 | -2.187 | 0.031 |
| Precentral L - Pallidum L | 0.241 | < 0.001 | 2.277 | 0.025 |
| Precentral L - Vermis 8 | 0.153 | 0.006 | 2.696 | 0.008 |
| Precentral L - Vermis 9 | 0.112 | 0.021 | 1.999 | 0.048 |
| Precentral R - Frontal Inf Oper L | 0.118 | 0.017 | -2.913 | 0.004 |
| Precentral R - Frontal Inf Oper R | 0.154 | 0.005 | -2.025 | 0.046 |
| Precentral R - Frontal Inf Tri L | 0.094 | 0.037 | -2.782 | 0.006 |
| Precentral R - Calcarine L | 0.130 | 0.012 | -2.475 | 0.015 |
| Precentral R - Calcarine R | 0.133 | 0.011 | -2.127 | 0.036 |
| Precentral R - Cuneus L | 0.179 | 0.002 | -2.289 | 0.024 |
| Precentral R - Cuneus R | 0.183 | 0.002 | -2.472 | 0.015 |
| Precentral R - Lingual L | 0.168 | 0.003 | -2.207 | 0.030 |
| Precentral R - Lingual R | 0.187 | 0.002 | -2.633 | 0.010 |
| Precentral R - Occipital Sup L | 0.212 | 0.001 | -2.280 | 0.025 |
| Precentral R - Occipital Sup R | 0.164 | 0.004 | -2.448 | 0.016 |
| Precentral R - Occipital Mid L | 0.198 | 0.001 | -2.616 | 0.010 |
| Precentral R - Occipital Mid R | 0.103 | 0.028 | -2.465 | 0.015 |
| Precentral R - Occipital Inf L | 0.194 | 0.001 | -3.116 | 0.002 |
| Precentral R - Fusiform L | 0.163 | 0.004 | -2.520 | 0.013 |
| Precentral R - Fusiform R | 0.156 | 0.005 | -2.108 | 0.038 |
| Precentral R - Temporal Sup R | 0.120 | 0.016 | -2.051 | 0.043 |
| Precentral R - Cerebellum Crus1 L | 0.134 | 0.011 | 2.047 | 0.043 |
| Precentral R - Cerebellum 9 L | 0.157 | 0.005 | 2.382 | 0.019 |
| Precentral R - Cerebellum 10 R | 0.266 | < 0.001 | 2.048 | 0.043 |
| Precentral R - Vermis 8 | 0.094 | 0.037 | 2.217 | 0.029 |
| Frontal Sup L - Cingulum Post L | 0.166 | 0.004 | -2.485 | 0.015 |
| Frontal Sup L - Angular L | 0.239 | < 0.001 | -2.051 | 0.043 |
| Frontal Sup L - Vermis 1 2 | 0.257 | < 0.001 | -2.083 | 0.040 |
| Frontal Sup R - Cingulum Post L | 0.233 | < 0.001 | -3.204 | 0.002 |
| Frontal Sup R - Cingulum Post R | 0.261 | < 0.001 | -3.004 | 0.003 |
| Frontal Sup R - Hippocampus L | 0.282 | < 0.001 | 2.509 | 0.014 |
| Frontal Sup R - Occipital Inf L | 0.110 | 0.022 | -2.138 | 0.035 |
| Frontal Sup R - Angular L | 0.138 | 0.009 | -2.008 | 0.047 |
| Frontal Sup R - Angular R | 0.237 | < 0.001 | -2.496 | 0.014 |
| Frontal Sup R - Precuneus L | 0.127 | 0.013 | -2.161 | 0.033 |
| Frontal Sup R - Caudate L | 0.121 | 0.016 | 2.361 | 0.020 |
| Frontal Sup R - Caudate R | 0.096 | 0.035 | 2.159 | 0.033 |
| Frontal Sup R - Pallidum R | 0.198 | 0.001 | 2.007 | 0.048 |
| Frontal Sup R - Cerebellum 4 5 R | 0.093 | 0.039 | -2.166 | 0.033 |
| Frontal Sup Orb L - Frontal Mid Orb R | 0.217 | 0.001 | 2.162 | 0.033 |
| Frontal Sup Orb L - Parietal Inf R | 0.205 | 0.001 | 2.080 | 0.040 |
| Frontal Sup Orb L - Thalamus L | 0.220 | 0.001 | 2.316 | 0.023 |
| Frontal Sup Orb R - Frontal Inf Tri L | 0.228 | < 0.001 | -2.227 | 0.028 |
| Frontal Sup Orb R - Lingual R | 0.339 | < 0.001 | 2.042 | 0.044 |
| Frontal Sup Orb R - Vermis 10 | 0.418 | < 0.001 | 3.139 | 0.002 |
| Frontal Mid L - Cingulum Post L | 0.225 | < 0.001 | -2.850 | 0.005 |
| Frontal Mid L - Cingulum Post R | 0.151 | 0.006 | -2.303 | 0.023 |
| Frontal Mid L - Angular R | 0.098 | 0.033 | -2.212 | 0.029 |
| Frontal Mid L - Heschl R | 0.085 | 0.049 | 2.155 | 0.034 |
| Frontal Mid L - Cerebellum Crus1 R | 0.246 | < 0.001 | -2.261 | 0.026 |
| Frontal Mid L - Cerebellum 7b R | 0.147 | 0.007 | -2.250 | 0.027 |
| Frontal Mid R - Cingulum Post R | 0.182 | 0.002 | -2.182 | 0.032 |
| Frontal Mid R - Hippocampus L | 0.153 | 0.006 | 2.025 | 0.046 |
| Frontal Mid R - Thalamus R | 0.124 | 0.015 | 2.440 | 0.016 |
| Frontal Mid Orb L - Frontal Sup Medial R | 0.216 | 0.001 | 2.090 | 0.039 |
| Frontal Mid Orb L - Thalamus L | 0.340 | < 0.001 | 2.224 | 0.028 |
| Frontal Mid Orb L - Temporal Pole Sup R | 0.224 | 0.001 | 2.040 | 0.044 |
| Frontal Mid Orb R - Occipital Sup R | 0.221 | 0.001 | 2.147 | 0.034 |
| Frontal Mid Orb R - Postcentral L | 0.228 | < 0.001 | 2.707 | 0.008 |
| Frontal Mid Orb R - Thalamus L | 0.218 | 0.001 | 2.587 | 0.011 |
| Frontal Mid Orb R - Vermis 3 | 0.197 | 0.001 | 2.536 | 0.013 |
| Frontal Inf Oper L - Frontal Inf Orb R | 0.228 | < 0.001 | -2.138 | 0.035 |
| Frontal Inf Oper L - Parietal Inf L | 0.135 | 0.010 | -2.418 | 0.017 |
| Frontal Inf Oper L - Temporal Mid L | 0.248 | < 0.001 | -2.420 | 0.017 |
| Frontal Inf Oper L - Temporal Inf L | 0.211 | 0.001 | -2.901 | 0.005 |
| Frontal Inf Oper L - Cerebellum 6 L | 0.138 | 0.009 | -2.168 | 0.033 |
| Frontal Inf Tri L - Frontal Inf Orb R | 0.221 | 0.001 | -2.249 | 0.027 |
| Frontal Inf Tri L - Frontal Med Orb R | 0.205 | 0.001 | -2.279 | 0.025 |
| Frontal Inf Tri L - Parietal Inf L | 0.136 | 0.010 | -2.191 | 0.031 |
| Frontal Inf Tri L - Putamen R | 0.118 | 0.017 | -2.066 | 0.041 |
| Frontal Inf Tri L - Temporal Inf L | 0.171 | 0.003 | -2.333 | 0.022 |
| Frontal Inf Tri R - Thalamus R | 0.138 | 0.009 | 2.089 | 0.039 |
| Frontal Inf Orb L - Vermis 4 5 | 0.195 | 0.001 | 2.214 | 0.029 |
| Frontal Inf Orb R - Lingual R | 0.216 | 0.001 | 2.411 | 0.018 |
| Frontal Inf Orb R - Thalamus L | 0.176 | 0.003 | 2.076 | 0.041 |
| Frontal Inf Orb R - Thalamus R | 0.181 | 0.002 | 2.188 | 0.031 |
| Rolandic Oper L - Supp Motor Area R | 0.115 | 0.019 | -2.224 | 0.028 |
| Rolandic Oper L - Amygdala R | 0.142 | 0.008 | -2.047 | 0.043 |
| Rolandic Oper L - Heschl L | 0.183 | 0.002 | -2.492 | 0.014 |
| Rolandic Oper L - Temporal Pole Sup R | 0.095 | 0.037 | -3.391 | 0.001 |
| Rolandic Oper R - Rectus R | 0.380 | < 0.001 | 2.093 | 0.039 |
| Rolandic Oper R - Cingulum Ant L | 0.097 | 0.034 | -2.345 | 0.021 |
| Rolandic Oper R - Cingulum Mid L | 0.197 | 0.001 | -2.819 | 0.006 |
| Rolandic Oper R - Temporal Pole Sup R | 0.151 | 0.006 | -2.448 | 0.016 |
| Supp Motor Area L - Occipital Sup R | 0.166 | 0.004 | -2.084 | 0.040 |
| Supp Motor Area L - Occipital Mid L | 0.173 | 0.003 | -2.405 | 0.018 |
| Supp Motor Area L - Occipital Mid R | 0.089 | 0.043 | -2.259 | 0.026 |
| Supp Motor Area L - Temporal Sup R | 0.125 | 0.014 | -2.001 | 0.048 |
| Supp Motor Area R - Occipital Sup L | 0.215 | 0.001 | -2.090 | 0.039 |
| Supp Motor Area R - SupraMarginal R | 0.111 | 0.022 | -2.064 | 0.042 |
| Supp Motor Area R - Cerebellum Crus1 L | 0.240 | < 0.001 | 2.865 | 0.005 |
| Supp Motor Area R - Vermis 7 | 0.257 | < 0.001 | 2.434 | 0.017 |
| Olfactory L - Vermis 4 5 | 0.210 | 0.001 | 3.113 | 0.002 |
| Frontal Sup Medial L - Cingulum Post L | 0.251 | < 0.001 | -2.763 | 0.007 |
| Frontal Sup Medial L - Cingulum Post R | 0.214 | 0.001 | -2.226 | 0.028 |
| Frontal Sup Medial L - Pallidum R | 0.188 | 0.002 | 2.579 | 0.011 |
| Frontal Sup Medial L - Vermis 9 | 0.203 | 0.001 | 3.101 | 0.003 |
| Frontal Sup Medial R - Temporal Mid R | 0.154 | 0.005 | -2.439 | 0.017 |
| Frontal Sup Medial R - Cerebellum 10 L | 0.248 | < 0.001 | 2.200 | 0.030 |
| Frontal Sup Medial R - Vermis 3 | 0.193 | 0.001 | 2.133 | 0.035 |
| Frontal Sup Medial R - Vermis 9 | 0.272 | < 0.001 | 2.319 | 0.022 |
| Frontal Med Orb L - Fusiform R | 0.238 | < 0.001 | -2.146 | 0.034 |
| Frontal Med Orb L - Temporal Pole Sup R | 0.166 | 0.004 | -2.009 | 0.047 |
| Frontal Med Orb L - Cerebellum Crus2 L | 0.183 | 0.002 | -2.392 | 0.019 |
| Frontal Med Orb L - Vermis 1 2 | 0.295 | < 0.001 | 2.407 | 0.018 |
| Frontal Med Orb R - Cingulum Post L | 0.207 | 0.001 | -2.255 | 0.026 |
| Frontal Med Orb R - Cerebellum Crus2 L | 0.179 | 0.002 | -2.057 | 0.042 |
| Rectus L - SupraMarginal L | 0.267 | < 0.001 | 1.998 | 0.048 |
| Rectus L - Temporal Pole Mid R | 0.156 | 0.005 | -2.086 | 0.040 |
| Rectus L - Cerebellum 9 R | 0.379 | < 0.001 | 2.125 | 0.036 |
| Rectus L - Vermis 10 | 0.211 | 0.001 | 2.443 | 0.016 |
| Rectus R - Insula L | 0.378 | < 0.001 | 3.902 | < 0.001 |
| Rectus R - Insula R | 0.323 | < 0.001 | 3.863 | < 0.001 |
| Rectus R - Lingual R | 0.377 | < 0.001 | 2.092 | 0.039 |
| Rectus R - Parietal Inf R | 0.294 | < 0.001 | 2.212 | 0.029 |
| Rectus R - SupraMarginal L | 0.243 | < 0.001 | 2.355 | 0.021 |
| Rectus R - Vermis 10 | 0.257 | < 0.001 | 2.006 | 0.048 |
| Insula L - Cuneus L | 0.296 | < 0.001 | 2.146 | 0.034 |
| Insula L - Occipital Sup R | 0.309 | < 0.001 | 2.143 | 0.035 |
| Insula L - Fusiform R | 0.283 | < 0.001 | 2.155 | 0.034 |
| Insula L - Parietal Sup L | 0.235 | < 0.001 | 2.293 | 0.024 |
| Insula L - Parietal Sup R | 0.212 | 0.001 | 2.392 | 0.019 |
| Insula L - Heschl L | 0.108 | 0.025 | -1.993 | 0.049 |
| Insula L - Cerebellum 6 R | 0.173 | 0.003 | 2.749 | 0.007 |
| Insula R - Lingual R | 0.234 | < 0.001 | 2.569 | 0.012 |
| Insula R - Occipital Inf R | 0.220 | 0.001 | 2.056 | 0.042 |
| Insula R - Fusiform R | 0.232 | < 0.001 | 2.781 | 0.007 |
| Cingulum Ant R - Temporal Sup L | 0.191 | 0.002 | -2.117 | 0.037 |
| Cingulum Mid L - Calcarine L | 0.207 | 0.001 | -2.665 | 0.009 |
| Cingulum Mid L - Cuneus R | 0.141 | 0.008 | -2.320 | 0.022 |
| Cingulum Mid L - Lingual R | 0.154 | 0.005 | -2.066 | 0.042 |
| Cingulum Mid L - Occipital Sup L | 0.139 | 0.009 | -2.280 | 0.025 |
| Cingulum Mid L - Occipital Sup R | 0.220 | 0.001 | -2.878 | 0.005 |
| Cingulum Mid L - Occipital Mid L | 0.092 | 0.040 | -2.340 | 0.021 |
| Cingulum Mid L - Temporal Sup R | 0.248 | < 0.001 | -2.636 | 0.010 |
| Cingulum Mid L - Temporal Pole Sup R | 0.188 | 0.002 | -2.169 | 0.032 |
| Cingulum Mid R - Hippocampus L | 0.189 | 0.002 | 2.149 | 0.034 |
| Cingulum Mid R - Occipital Mid L | 0.162 | 0.004 | -2.239 | 0.027 |
| Cingulum Mid R - Fusiform L | 0.160 | 0.005 | -2.260 | 0.026 |
| Cingulum Mid R - SupraMarginal L | 0.178 | 0.002 | -2.698 | 0.008 |
| Cingulum Mid R - Temporal Sup R | 0.214 | 0.001 | -2.779 | 0.007 |
| Cingulum Mid R - Temporal Pole Sup L | 0.145 | 0.007 | -2.092 | 0.039 |
| Cingulum Post L - Cerebellum Crus1 R | 0.302 | < 0.001 | -2.846 | 0.005 |
| Cingulum Post L - Cerebellum Crus2 L | 0.234 | < 0.001 | -3.010 | 0.003 |
| Cingulum Post L - Cerebellum 8 R | 0.302 | < 0.001 | -2.498 | 0.014 |
| Cingulum Post L - Cerebellum 9 R | 0.283 | < 0.001 | -2.800 | 0.006 |
| Cingulum Post R - Temporal Pole Mid L | 0.155 | 0.005 | 2.145 | 0.034 |
| Cingulum Post R - Cerebellum Crus1 R | 0.271 | < 0.001 | -1.987 | 0.050 |
| Hippocampus L - SupraMarginal L | 0.210 | 0.001 | 2.453 | 0.016 |
| Hippocampus L - Vermis 1 2 | 0.213 | 0.001 | 2.118 | 0.037 |
| Hippocampus R - Amygdala L | 0.106 | 0.026 | -2.459 | 0.016 |
| Hippocampus R - Parietal Inf R | 0.186 | 0.002 | 2.096 | 0.039 |
| Hippocampus R - SupraMarginal L | 0.280 | < 0.001 | 2.530 | 0.013 |
| Hippocampus R - Angular R | 0.202 | 0.001 | 2.468 | 0.015 |
| ParaHippocampal R - Amygdala R | 0.134 | 0.010 | -2.366 | 0.020 |
| ParaHippocampal R - Caudate R | 0.242 | < 0.001 | 2.119 | 0.037 |
| ParaHippocampal R - Cerebellum 4 5 L | 0.252 | < 0.001 | 2.097 | 0.039 |
| Amygdala L - Angular L | 0.145 | 0.007 | 2.037 | 0.044 |
| Amygdala L - Cerebellum 7b R | 0.171 | 0.003 | 2.097 | 0.039 |
| Amygdala R - Temporal Pole Mid R | 0.197 | 0.001 | -3.240 | 0.002 |
| Amygdala R - Vermis 9 | 0.127 | 0.013 | 2.090 | 0.039 |
| Calcarine L - Postcentral L | 0.178 | 0.002 | -2.184 | 0.031 |
| Calcarine L - Postcentral R | 0.116 | 0.019 | -2.260 | 0.026 |
| Calcarine L - Precuneus L | 0.198 | 0.001 | -3.200 | 0.002 |
| Calcarine L - Precuneus R | 0.172 | 0.003 | -2.815 | 0.006 |
| Calcarine L - Paracentral Lobule L | 0.201 | 0.001 | -2.461 | 0.016 |
| Calcarine L - Pallidum L | 0.108 | 0.024 | 2.015 | 0.047 |
| Calcarine L - Cerebellum 9 R | 0.106 | 0.025 | -2.507 | 0.014 |
| Calcarine R - Postcentral L | 0.160 | 0.004 | -2.582 | 0.011 |
| Calcarine R - Postcentral R | 0.134 | 0.011 | -2.435 | 0.017 |
| Calcarine R - Precuneus L | 0.172 | 0.003 | -2.178 | 0.032 |
| Calcarine R - Paracentral Lobule L | 0.136 | 0.010 | -2.409 | 0.018 |
| Calcarine R - Paracentral Lobule R | 0.122 | 0.015 | -2.077 | 0.040 |
| Cuneus L - Fusiform R | 0.189 | 0.002 | -2.211 | 0.029 |
| Cuneus L - Postcentral L | 0.141 | 0.008 | -2.205 | 0.030 |
| Cuneus L - Postcentral R | 0.101 | 0.030 | -2.200 | 0.030 |
| Cuneus L - Parietal Sup R | 0.104 | 0.028 | -2.201 | 0.030 |
| Cuneus L - Precuneus R | 0.275 | < 0.001 | -2.286 | 0.024 |
| Cuneus L - Pallidum L | 0.205 | 0.001 | 2.518 | 0.013 |
| Cuneus L - Temporal Inf R | 0.108 | 0.025 | -2.216 | 0.029 |
| Cuneus L - Cerebellum Crus1 R | 0.168 | 0.003 | -2.348 | 0.021 |
| Cuneus L - Cerebellum 9 R | 0.195 | 0.001 | -3.288 | 0.001 |
| Cuneus R - Lingual L | 0.217 | 0.001 | -2.178 | 0.032 |
| Cuneus R - Postcentral L | 0.182 | 0.002 | -2.997 | 0.003 |
| Cuneus R - Postcentral R | 0.119 | 0.017 | -2.447 | 0.016 |
| Cuneus R - Paracentral Lobule R | 0.203 | 0.001 | -2.165 | 0.033 |
| Cuneus R - Pallidum R | 0.264 | < 0.001 | 2.740 | 0.007 |
| Lingual L - Postcentral L | 0.170 | 0.003 | -2.798 | 0.006 |
| Lingual L - Postcentral R | 0.173 | 0.003 | -2.214 | 0.029 |
| Lingual L - Parietal Sup R | 0.200 | 0.001 | -2.233 | 0.028 |
| Lingual L - Precuneus L | 0.171 | 0.003 | -2.400 | 0.018 |
| Lingual L - Precuneus R | 0.141 | 0.009 | -2.838 | 0.006 |
| Lingual L - Paracentral Lobule L | 0.197 | 0.001 | -2.202 | 0.030 |
| Lingual L - Thalamus R | 0.168 | 0.003 | -2.035 | 0.045 |
| Lingual L - Cerebellum 8 R | 0.191 | 0.002 | -2.202 | 0.030 |
| Lingual R - Postcentral L | 0.178 | 0.002 | -2.957 | 0.004 |
| Lingual R - Postcentral R | 0.131 | 0.011 | -2.171 | 0.032 |
| Lingual R - Precuneus L | 0.125 | 0.014 | -1.999 | 0.048 |
| Lingual R - Precuneus R | 0.146 | 0.007 | -2.258 | 0.026 |
| Lingual R - Paracentral Lobule L | 0.240 | < 0.001 | -3.303 | 0.001 |
| Occipital Sup L - Fusiform R | 0.164 | 0.004 | -2.230 | 0.028 |
| Occipital Sup L - Postcentral L | 0.238 | < 0.001 | -2.697 | 0.008 |
| Occipital Sup L - Postcentral R | 0.210 | 0.001 | -2.398 | 0.018 |
| Occipital Sup L - Parietal Sup R | 0.155 | 0.005 | -2.046 | 0.043 |
| Occipital Sup R - Fusiform L | 0.186 | 0.002 | -3.107 | 0.002 |
| Occipital Sup R - Fusiform R | 0.191 | 0.002 | -2.302 | 0.023 |
| Occipital Sup R - Postcentral L | 0.175 | 0.003 | -2.117 | 0.037 |
| Occipital Sup R - Postcentral R | 0.133 | 0.011 | -2.026 | 0.045 |
| Occipital Sup R - Temporal Inf L | 0.095 | 0.036 | -2.549 | 0.012 |
| Occipital Mid L - Postcentral L | 0.262 | < 0.001 | -3.433 | 0.001 |
| Occipital Mid L - Postcentral R | 0.221 | 0.001 | -3.058 | 0.003 |
| Occipital Mid L - Parietal Sup L | 0.290 | < 0.001 | -2.674 | 0.009 |
| Occipital Mid L - Parietal Sup R | 0.288 | < 0.001 | -3.838 | < 0.001 |
| Occipital Mid L - Paracentral Lobule L | 0.168 | 0.003 | -2.255 | 0.026 |
| Occipital Mid L - Paracentral Lobule R | 0.159 | 0.005 | -2.111 | 0.037 |
| Occipital Mid R - Fusiform L | 0.155 | 0.005 | -2.304 | 0.023 |
| Occipital Mid R - Postcentral L | 0.150 | 0.006 | -2.206 | 0.030 |
| Occipital Mid R - Postcentral R | 0.152 | 0.006 | -2.582 | 0.011 |
| Occipital Mid R - Parietal Sup R | 0.257 | < 0.001 | -2.248 | 0.027 |
| Occipital Mid R - Putamen R | 0.116 | 0.019 | 2.040 | 0.044 |
| Occipital Mid R - Temporal Inf L | 0.112 | 0.021 | -2.498 | 0.014 |
| Occipital Mid R - Temporal Inf R | 0.105 | 0.027 | -2.234 | 0.028 |
| Occipital Inf L - Postcentral L | 0.170 | 0.003 | -2.406 | 0.018 |
| Occipital Inf L - Postcentral R | 0.147 | 0.007 | -2.223 | 0.029 |
| Occipital Inf L - Cerebellum 8 R | 0.170 | 0.003 | -2.101 | 0.038 |
| Occipital Inf R - Postcentral L | 0.141 | 0.008 | -2.169 | 0.033 |
| Occipital Inf R - Caudate R | 0.168 | 0.003 | 2.748 | 0.007 |
| Occipital Inf R - Cerebellum 6 L | 0.121 | 0.016 | 2.079 | 0.040 |
| Fusiform L - Fusiform R | 0.456 | < 0.001 | -2.207 | 0.030 |
| Fusiform L - Postcentral L | 0.164 | 0.004 | -2.258 | 0.026 |
| Fusiform L - Postcentral R | 0.104 | 0.027 | -2.011 | 0.047 |
| Fusiform L - Thalamus R | 0.253 | < 0.001 | -2.777 | 0.007 |
| Fusiform R - Postcentral L | 0.147 | 0.007 | -2.174 | 0.032 |
| Fusiform R - Cerebellum 8 L | 0.132 | 0.011 | -2.194 | 0.031 |
| Postcentral L - Temporal Sup R | 0.193 | 0.001 | -1.995 | 0.049 |
| Postcentral L - Cerebellum Crus1 R | 0.192 | 0.002 | 1.986 | 0.050 |
| Postcentral L - Cerebellum Crus2 L | 0.220 | 0.001 | 2.421 | 0.017 |
| Postcentral L - Cerebellum Crus2 R | 0.112 | 0.021 | 2.378 | 0.019 |
| Postcentral R - Parietal Inf R | 0.148 | 0.007 | 2.054 | 0.043 |
| Postcentral R - Pallidum L | 0.170 | 0.003 | 2.111 | 0.037 |
| Postcentral R - Temporal Sup R | 0.087 | 0.047 | -2.339 | 0.021 |
| Parietal Inf L - Temporal Inf R | 0.140 | 0.009 | -1.996 | 0.049 |
| Parietal Inf L - Vermis 3 | 0.144 | 0.008 | 2.399 | 0.018 |
| Parietal Inf L - Vermis 8 | 0.103 | 0.028 | 2.469 | 0.015 |
| Parietal Inf R - SupraMarginal R | 0.177 | 0.003 | 2.097 | 0.039 |
| Parietal Inf R - Precuneus L | 0.119 | 0.017 | 2.642 | 0.010 |
| Parietal Inf R - Pallidum L | 0.284 | < 0.001 | 2.438 | 0.017 |
| Parietal Inf R - Thalamus R | 0.137 | 0.010 | 2.039 | 0.044 |
| Parietal Inf R - Temporal Mid L | 0.247 | < 0.001 | 2.179 | 0.032 |
| Parietal Inf R - Cerebellum 4 5 L | 0.173 | 0.003 | 2.023 | 0.046 |
| SupraMarginal L - Precuneus L | 0.136 | 0.010 | 2.207 | 0.030 |
| SupraMarginal L - Cerebellum 4 5 L | 0.236 | < 0.001 | 2.634 | 0.010 |
| SupraMarginal R - Pallidum L | 0.100 | 0.031 | 2.192 | 0.031 |
| Angular L - Pallidum R | 0.246 | < 0.001 | 2.081 | 0.040 |
| Angular L - Vermis 3 | 0.134 | 0.010 | 2.515 | 0.014 |
| Angular R - Temporal Inf R | 0.173 | 0.003 | -2.150 | 0.034 |
| Angular R - Cerebellum Crus1 L | 0.224 | 0.001 | -2.362 | 0.020 |
| Angular R - Cerebellum Crus1 R | 0.188 | 0.002 | -2.346 | 0.021 |
| Angular R - Cerebellum 6 R | 0.121 | 0.016 | -2.027 | 0.045 |
| Angular R - Cerebellum 10 L | 0.320 | < 0.001 | 2.435 | 0.017 |
| Precuneus L - Pallidum L | 0.149 | 0.007 | 2.816 | 0.006 |
| Precuneus L - Temporal Inf R | 0.097 | 0.034 | -2.156 | 0.034 |
| Precuneus L - Cerebellum 7b R | 0.142 | 0.008 | -2.051 | 0.043 |
| Precuneus L - Cerebellum 9 R | 0.366 | < 0.001 | -2.487 | 0.015 |
| Precuneus R - Pallidum L | 0.217 | 0.001 | 3.333 | 0.001 |
| Precuneus R - Pallidum R | 0.420 | < 0.001 | 2.578 | 0.011 |
| Precuneus R - Cerebellum Crus1 R | 0.159 | 0.005 | -2.427 | 0.017 |
| Precuneus R - Cerebellum 4 5 L | 0.141 | 0.008 | -1.991 | 0.049 |
| Precuneus R - Cerebellum 8 R | 0.087 | 0.046 | -2.364 | 0.020 |
| Paracentral Lobule R - Pallidum L | 0.175 | 0.003 | 2.197 | 0.030 |
| Caudate L - Temporal Pole Mid R | 0.180 | 0.002 | 2.334 | 0.022 |
| Caudate L - Temporal Inf R | 0.171 | 0.003 | 2.183 | 0.031 |
| Caudate L - Cerebellum 7b R | 0.265 | < 0.001 | 2.173 | 0.032 |
| Caudate L - Cerebellum 8 L | 0.166 | 0.004 | 2.018 | 0.046 |
| Caudate L - Vermis 10 | 0.177 | 0.003 | 2.461 | 0.016 |
| Caudate R - Putamen L | 0.209 | 0.001 | -2.112 | 0.037 |
| Caudate R - Temporal Pole Mid L | 0.162 | 0.004 | 2.112 | 0.037 |
| Caudate R - Temporal Pole Mid R | 0.251 | < 0.001 | 2.441 | 0.016 |
| Caudate R - Vermis 10 | 0.156 | 0.005 | 2.055 | 0.043 |
| Putamen L - Temporal Pole Sup R | 0.212 | 0.001 | -2.205 | 0.030 |
| Putamen L - Cerebellum 10 L | 0.349 | < 0.001 | 2.484 | 0.015 |
| Pallidum L - Thalamus R | 0.228 | < 0.001 | 2.525 | 0.013 |
| Pallidum L - Temporal Sup L | 0.110 | 0.023 | 1.985 | 0.050 |
| Pallidum L - Temporal Sup R | 0.248 | < 0.001 | 2.356 | 0.020 |
| Pallidum L - Temporal Mid R | 0.131 | 0.011 | 2.409 | 0.018 |
| Pallidum R - Temporal Sup R | 0.122 | 0.016 | 2.206 | 0.030 |
| Thalamus R - Heschl R | 0.197 | 0.001 | 2.196 | 0.030 |
| Thalamus R - Temporal Sup R | 0.165 | 0.004 | 2.272 | 0.025 |
| Thalamus R - Cerebellum 6 L | 0.212 | 0.001 | -2.264 | 0.026 |
| Thalamus R - Vermis 10 | 0.268 | < 0.001 | 2.199 | 0.030 |
| Heschl L - Cerebellum 3 L | 0.089 | 0.043 | -2.523 | 0.013 |
| Temporal Sup L - Cerebellum Crus1 L | 0.086 | 0.048 | 2.092 | 0.039 |
| Temporal Sup L - Cerebellum 6 R | 0.133 | 0.011 | 2.065 | 0.042 |
| Temporal Sup L - Vermis 3 | 0.103 | 0.028 | 2.016 | 0.047 |
| Temporal Sup R - Vermis 3 | 0.224 | 0.001 | 2.244 | 0.027 |
| Temporal Sup R - Vermis 4 5 | 0.238 | < 0.001 | 2.111 | 0.037 |
| Temporal Pole Sup L - Cerebellum 8 L | 0.114 | 0.020 | -2.139 | 0.035 |
| Temporal Pole Sup R - Cerebellum 7b R | 0.129 | 0.012 | 2.071 | 0.041 |
| Temporal Pole Sup R - Vermis 3 | 0.174 | 0.003 | 2.439 | 0.017 |
| Temporal Mid L - Vermis 3 | 0.161 | 0.004 | 2.425 | 0.017 |
| Temporal Mid L - Vermis 4 5 | 0.254 | < 0.001 | 2.538 | 0.013 |
| Temporal Mid R - Vermis 3 | 0.223 | 0.001 | 2.370 | 0.020 |
| Temporal Mid R - Vermis 4 5 | 0.316 | < 0.001 | 2.982 | 0.004 |
| Temporal Inf L - Cerebellum 7b R | 0.089 | 0.043 | -2.215 | 0.029 |
| Temporal Inf L - Cerebellum 8 R | 0.119 | 0.017 | -2.026 | 0.046 |
| Temporal Inf R - Cerebellum 8 L | 0.198 | 0.001 | -2.475 | 0.015 |
| Cerebellum Crus1 L - Cerebellum 7b R | 0.132 | 0.011 | -2.432 | 0.017 |
| Cerebellum Crus1 R - Cerebellum Crus2 L | 0.198 | 0.001 | -2.323 | 0.022 |
| Cerebellum 3 R - Vermis 3 | 0.122 | 0.015 | 2.228 | 0.028 |
| Cerebellum 3 R - Vermis 4 5 | 0.242 | < 0.001 | 2.492 | 0.014 |
| Cerebellum 4 5 L - Cerebellum 9 R | 0.208 | 0.001 | -2.406 | 0.018 |
| Cerebellum 4 5 R - Vermis 1 2 | 0.220 | 0.001 | 4.066 | < 0.001 |
| Cerebellum 6 L - Cerebellum 6 R | 0.439 | < 0.001 | -2.111 | 0.037 |
| Cerebellum 6 L - Cerebellum 8 R | 0.255 | < 0.001 | -2.513 | 0.014 |
| Cerebellum 6 R - Cerebellum 8 R | 0.284 | < 0.001 | -1.989 | 0.050 |
| Cerebellum 7b R - Vermis 7 | 0.189 | 0.002 | -2.181 | 0.032 |
| Cerebellum 8 R - Vermis 6 | 0.198 | 0.001 | -2.239 | 0.027 |
| Cerebellum 8 R - Vermis 7 | 0.218 | 0.001 | -2.095 | 0.039 |
| Cerebellum 9 L - Vermis 3 | 0.164 | 0.004 | -2.132 | 0.035 |
| Cerebellum 9 R - Vermis 3 | 0.236 | < 0.001 | -2.425 | 0.017 |

**Supplementary Table 18**. Frailty on ROI-to-ROI functional connectivity for FTLD adjusting for scanner type and data quality metrics. *P*_FDR_<0.05. Regions are presented using the AAL atlas.

| **Group** | **Variable** | **Statistics** |
| --- | --- | --- |
| CU | MRI-SNR | *r* = -0.1416, *P*_FDR_ = 0.230 |
|  | fMRI-SNR | *r* = -0.1379, *P*_FDR_ = 0.221 |
|  | fMRI-temporal SNR | *r* = -0.1091, *P*_FDR_ = 0.276 |
| AD | MRI-SNR | *r* = -0.0569, *P*_FDR_ = 0.570 |
|  | fMRI-SNR | *r* = 0.0268, *P*_FDR_ = 0.745 |
|  | fMRI-temporal SNR | *r* = -0.0267, *P*_FDR_ = 0.745 |
| FTLD | MRI-SNR | *r* = -0.1283, *P*_FDR_ = 0.276 |
|  | fMRI-SNR | *r* = 0.0753, *P*_FDR_ = 0.713 |
|  | fMRI-temporal SNR | *r* = -0.0343, *P*_FDR_ = 0.745 |

**Supplementary Table 19.** Associations between frailty and MRI/fMRI data quality metrics. SNR: signal-to-noise ratio.

| **Regions** | **Model** | | **Frailty** | |
| --- | --- | --- | --- | --- |
|  | **R^2^_adj_** | ***P*_FDR_** | ***t*** | ***P*_FDR_** |
| Precentral L | 0.191 | < 0.001 | -3.732 | < 0.001 |
| Precentral R | 0.296 | < 0.001 | -4.386 | < 0.001 |
| Frontal Sup Orb L | 0.168 | < 0.001 | -2.439 | 0.015 |
| Frontal Mid L | 0.161 | < 0.001 | -2.300 | 0.022 |
| Frontal Mid R | 0.112 | < 0.001 | -2.226 | 0.027 |
| Frontal Mid Orb L | 0.091 | < 0.001 | -1.967 | 0.050 |
| Frontal Inf Oper L | 0.081 | < 0.001 | -2.364 | 0.019 |
| Frontal Inf Oper R | 0.085 | < 0.001 | -2.973 | 0.003 |
| Frontal Inf Tri R | 0.086 | < 0.001 | -2.350 | 0.019 |
| Rolandic Oper L | 0.120 | < 0.001 | -2.932 | 0.004 |
| Supp Motor Area R | 0.150 | < 0.001 | -2.288 | 0.023 |
| Olfactory L | 0.218 | < 0.001 | -2.453 | 0.015 |
| Rectus R | 0.245 | < 0.001 | -3.436 | 0.001 |
| Insula R | 0.194 | < 0.001 | -2.659 | 0.008 |
| Cingulum Mid R | 0.190 | < 0.001 | -2.114 | 0.035 |
| Hippocampus L | 0.220 | < 0.001 | -2.574 | 0.010 |
| Hippocampus R | 0.249 | < 0.001 | -3.069 | 0.002 |
| ParaHippocampal L | 0.246 | < 0.001 | -2.137 | 0.033 |
| Amygdala R | 0.184 | < 0.001 | -2.738 | 0.006 |
| Calcarine R | 0.166 | < 0.001 | -2.566 | 0.011 |
| Occipital Sup L | 0.181 | < 0.001 | -2.283 | 0.023 |
| Occipital Sup R | 0.241 | < 0.001 | -3.345 | 0.001 |
| Occipital Mid L | 0.207 | < 0.001 | -1.989 | 0.047 |
| Occipital Mid R | 0.228 | < 0.001 | -3.010 | 0.003 |
| Occipital Inf R | 0.186 | < 0.001 | -2.599 | 0.010 |
| Fusiform L | 0.285 | < 0.001 | -2.044 | 0.042 |
| Postcentral L | 0.239 | < 0.001 | -3.852 | < 0.001 |
| Postcentral R | 0.234 | < 0.001 | -1.980 | 0.048 |
| Parietal Sup R | 0.237 | < 0.001 | -2.699 | 0.007 |
| SupraMarginal R | 0.158 | < 0.001 | -2.373 | 0.018 |
| Thalamus L | 0.225 | < 0.001 | -2.840 | 0.005 |
| Thalamus R | 0.277 | < 0.001 | -3.043 | 0.003 |
| Heschl L | 0.155 | < 0.001 | -2.880 | 0.004 |
| Temporal Sup L | 0.162 | < 0.001 | -3.146 | 0.002 |
| Temporal Sup R | 0.177 | < 0.001 | -2.552 | 0.011 |
| Temporal Mid L | 0.189 | < 0.001 | -2.473 | 0.014 |
| Temporal Inf R | 0.313 | < 0.001 | -1.982 | 0.048 |
| Cerebelum Crus2 L | 0.062 | < 0.001 | -2.417 | 0.016 |
| Cerebelum Crus2 R | 0.057 | < 0.001 | -2.191 | 0.029 |
| Cerebelum 3 L | 0.056 | < 0.001 | -2.251 | 0.025 |
| Cerebelum 7b L | 0.053 | 0.001 | -2.798 | 0.005 |
[truncated: 51,753 more chars]
